# Assessment of the impacts of artificial intelligence (AI) on intercultural communication among postgraduate students in a multicultural university environment

**DOI:** 10.1038/s41598-024-63276-5

**Published:** 2024-06-15

**Authors:** Abdul Qahar Sarwari, Muhammad Naeem Javed, Hamedi Mohd Adnan, Mohammad Nubli Abdul Wahab

**Affiliations:** 1 Department of Media and Communication Studies, Faculty of Arts and Social Sciences, University of Malaya (UM), 50603 Kuala Lumpur, Malaysia; 2 Center for Human Sciences (CHS), University Malaysia Pahang Al-Sultan Abdullah (UMPSA), Lebuhraya Tun Razak, 26300 Gambang, Kuantan, Pahang Malaysia; 3 Department of Media and Communication Studies, Emerson University Multan, Multan, Pakistan

**Keywords:** Artificial intelligence (AI), ChatGPT, Intercultural communication, Mediated communication, Modern communication technologies, Evolution, Neuroscience, Mathematics and computing

## Abstract

Artificial intelligence (AI) broadly influences different aspects of human life, especially human communication. One of the main concerns of the broad use of AI in daily interactions among different people could be whether it helps them interact easily or complicates their interactions. To answer the mentioned question, this study assessed the impacts of AI on intercultural communication among postgraduate students in a multicultural university environment. A newly developed survey instrument was used to conduct this study. The participants of this study were 115 postgraduate students from nine different countries. The descriptive statistics, reliability analysis, and Bivariate correlation tests of the 29th version of IBM-SPSS software were used to analyze the quantitative data, and inductive coding and conceptual content analysis were used to code and analyze the qualitative data. Based on descriptive results, the vast majority (93%) of the participants already used and experienced AI in their daily lives, and the majority of them believed that AI and AI technologies connect different cultures, reduce language and cultural barriers, and help people of different cultures to interact and be connected. Based on the results from the correlation test, there were strong positive correlations between AI attitudes and AI benefits, and also between AI regulation and AI benefits.

## Introduction

Communication is an essential part of human life that enables people to interact, share information, establish relationships, be connected, establish roles and values, and so on. From different types of human communication, intercultural communication is among the main requirements of modern life in the current globalized world with ever-growing multicultural environments and organizations deeply influenced by modern technologies. Intercultural communication is interactions between individuals from diverse cultures, and as a figurative and mutual process, engages meaning attribution between people from different cultures^1,2^. Different cultures have dissimilar values, languages, and patterns, and research on intercultural communication enables individuals to answer questions regarding people who have dissimilar cultural practices^1^.

Similar to other aspects of human communication, intercultural communication can be conducted directly or indirectly through the application of modern technologies. The application of communication technologies in daily interactions among people from different cultures has quite a long history. So far, AI and AI-powered technologies have been broadly used in human communication^3^, and the current fast growth and massive use of AI profoundly influenced different aspects of human life, including human communication^4–6^. Currently, AI and AI technologies have increased the speed of communication and information acquisition^3,7^, offer some innovative ways of interaction^8^, and enable people, especially lonely individuals to be connected^9^. Moreover, AI and AI technologies profoundly challenged and changed traditional communicative norms and theories^5,10^.

Scholars define AI as a technology that enables machines to act intelligently^11^. AI is “the science and engineering of making intelligent machines”^12^. The term 'artificial intelligence' was coined in 1956 at the Dartmouth Conference^13^. Moreover, after more than six decades since the emergence of AI as a new academic discipline, its fast growth has driven a massive increase in interest in the field, and it has received considerable attention from researchers, business interests, governments, and the public in the twenty-first century^8,14^.

It is noticeable that not only AI and AI technologies are essential requirements of modern communication and profoundly influence different aspects of communication, but communication is an essential requirement for theoretical and practical aspects of AI and AI technologies as well. Communication is essential for both the theoretical and practical aspects of AI, and it is the role of communication that enables science to have definitive tests and experimental evidence^13^.

Moreover, through the rapid growth of AI and AI-powered machines, the role of machines changed from mediators of communication between humans to communicators and partners of humans in human–machine communication. Thus, human-AI communication, in which people can interact with AI-powered machines through social bots and virtual agents, cannot neatly fit into existing communication theories and paradigms that focus on human–human interactions^10^. However, to effectively apply AI and AI technologies in their interactions and interact with the ever-growing intelligent machines, people need to gain and improve some essential skills, such as positive attitudes towards AI-powered machines, and language and technical skills. Because of the spread of AI in more areas and aspects of human life, improving AI skills is essential and will be more significant in the future^4^. Also, the fast development of AI asks individuals to know how to utilize it effectively^15^. Moreover, language skills, machine-learning knowledge, and awareness of the main benefits and problematic aspects of using AI-powered systems are essential for interactions with AI-powered systems^16^.

However, the fast growth and massive use of AI and AI-powered technologies in human communication profoundly challenged the existing norms and theories in the field of communication. The growth of AI profoundly changed human life^5^, and the current communication theories cannot cover human–machine communication precisely^10^. Assessment of the use and impacts of AI and AI technologies in different aspects of human communication, especially intercultural communication in modern collegiate environments, could answer some questions regarding the role of AI and AI technologies in human communication. Even though, AI has been extensively used in communication, but the probable social outcomes of using AI in communication still need to be explored^3^.

Moreover, people use AI and AI-related technologies in different aspects of human communication, including intercultural communication. Intercultural communication is an interesting and, at the same time, challenging type of communication, and could be influenced by AI and AI technologies like other types of human communication. Nowadays, people use AI and AI-powered technologies to overcome language, time, and space barriers when interacting with individuals from different cultures. However, because of the lack of evidence and information in the literature, it is unclear whether applying AI and AI technologies in daily interactions among individuals from different cultures in multicultural environments makes intercultural communication more accessible or complicated. Thus, to answer the mentioned concern and question, this study, through the application of a mixed-methods survey, assessed the impacts of AI and AI technologies on interactions among individuals from different cultures in a multicultural university environment.

## Literature review

In the current globalized world, which is deeply affected by modern technologies, intercultural communication is an essential requirement of modern life. In the ever-growing multicultural surroundings, communication among people from dissimilar cultures is an important part of their personal and professional lives^17^. Intercultural communication, which is a relatively new discipline and was pioneered by Hall^18^ in the late 1950s, has been assessed and studied by various researchers and from various perspectives^19,20^. In the ever-growing concept of globalization in the world, the skill of performing interactions and being connected to people from various backgrounds is considered an essential competence, both for the national and international contexts^21^. Currently, people use AI and AI-powered technologies to overcome language barriers and get information from different languages and cultures without the mediation of human interpreters. Based on the reviewed literature, AI and AI technologies help and enable people to conduct direct or indirect intercultural communication with different people, and the mentioned ever-growing modern technologies fundamentally influenced the ways people interact with one another^3,5–7^.

Moreover, boundaries in the modern world cannot imply permanent lines, leastwise in cultural, economic, and linguistic intelligence^21^. Modern technologies enabled people from different backgrounds to overcome time and space and interact with one another. As an essential part and requirement of modern life, communication has been deeply affected by ever-growing modern technologies, especially artificial intelligence (AI) and AI technologies. Based on the published literature, people have broadly used AI and AI technologies in their daily interactions, and AI has profoundly influenced human communication^3,5^. As an industrial revolution, AI can change how we perform, relate to others, and know ourselves^11^. AI as the fastest and the most important technology, has various benefits and different aspects of individual and social lives of human societies^22,23^. The term 'artificial intelligence' was produced in 1956 at the Dartmouth Conference, and before that, its primary aspect appeared in 1950 in Turing's “Game of Imitation”^13^. Currently, AI is applicable in every aspect of human life^4^, and AI can be defined as a technology that is created to allow 'computers and machines' to 'function intelligently'^10^.

According to the reviewed works, AI and AI technologies influenced different aspects of human communication and offer new opportunities. AI technologies offer more innovative functionalities supporting human-like interactions than computer software^8^. AI can increase information acquisition opportunities and expand human relationships^7^, and algorithmic replies could increase communication speed^3^. Moreover, the use of AI has become essential in different areas, especially in the field of education^24^.

Based on the literature, to apply AI and AI technologies in their daily interactions effectively, people need to gain, improve, and use some particular skills, such as language skills, technical skills, awareness about helpful and challenging aspects of AI utilization, and helpful attitudes toward AI technologies as inevitable requirements of modern life and modern communication. Because of the spread of AI in more areas and aspects of human life, improving AI skills is essential and will be more significant in the future^4^. Attitudes towards robots, digital literacy, and users' literacy are the main requirements of AI literacy^5^. The fast development of AI asks individuals to know how to utilize it effectively to enhance their success^15^. Moreover, awareness of the benefits and challenges of AI and positive attitudes towards AI technologies are among AI skills^16^.

The reviewed works indicate that AI and communication have mutual effects on each other, as AI and AI technologies deeply influence communication and are essential requirements of modern communication; communication is an essential requirement of theoretical and practical aspects and developments of AI and AI technologies. Communication is the main requirement for AI's practical and theoretical aspects, as communication enables scientists and users to test, define, and use it^13^.

Based on the literature, people broadly use AI and AI technologies daily. However, so far, the impact of the massive use of mentioned technology on social and cultural aspects of human life remains unexplored. However, despite concerns regarding the probable negative impacts of AI on human life, its outcomes on societies still need to be explored^3^. To apply AI and AI technologies effectively, people need to gain skills and know the primary outcomes and challenges of AI and AI technologies. Because of the spread of AI in human life, AI skills are essential requirements^4^, and awareness of the benefits and challenges of AI and positive attitudes towards AI technologies are among AI skills^16^.

However, besides some helpful effects of AI on human communication, it needs to be clarified whether AI and AI technologies make intercultural interactions among people of different cultures easier or more complicated. Thus, assessing the mentioned issue may add helpful information to the literature and answer probable questions regarding the probable outcomes of AI and AI technologies in intercultural communication.

## Methodology

The current study applied a mixed methods survey, which includes quantitative structured items and qualitative open-ended questions, to assess the impacts of AI and AI-powered technologies on interactions among individuals from different cultures in a multicultural university environment. The mixed method design can involve multiple structures of data collection procedures as researchers apply mixed methods to comprise different data sets to answer research questions^25^. Mixed methods included quantitative and qualitative data in the same survey to strengthen quantitative findings by results from the open-ended questions and vice versa. The mixed method design differs from the mixed methodology^26^. Moreover, for the research design, data collection procedures, data analysis, and data report, all research regulations of University of Malaya (UM) were considered and applied, and the study was approved by the ethics committee of the Department of Media and Communication Studies of University of Malaya (UM). A letter of informed consent was obtained from all subjects and/or their legal guardian(s) before the data collection procedure. Research instruments used in the current study, the participants, and the data analysis procedures of the study are described below:

### Participants

The current study had 115 participants from postgraduate students of University of Malaya (UM), the top public university in Malaysia, and the participants were from nine different countries. Participation in the current study was voluntary, and the participants participated after reading the consent letter and based on their willingness. The population of this study was about 1,000 regular and active international postgraduate students of the mentioned university. Even Though the actual number of the university's postgraduate students is high, after the Covid-19 pandemic, students primarily work from their home countries and home towns; during the data collection procedure, about 1000 active and regular postgraduate students were on campus, and the target population was the mentioned number of students. The convenience data collection method was used to collect the survey data from regular and active international postgraduate students who were staying and studying at the university campus. Of all 115 participants, 46 (40%) were male, and 69 (60%) of them female. Of them, 65 (56.5%) were Master's students, and 50 (43.5%) were PhD students. Of all 115 participants, 107 (93%) reported that they already experienced the use of AI, especially ChatGPT, and eight (7%) of them had yet to use AI and AI technologies.

As the survey instrument included 15 structured items and six open-ended questions, of all 115 participants, 105 shared their points of view and answers for all or some open-ended questions. All 105 respondents who answered the open-ended questions numbered P1 for Participant 1, P2 for Participant 2, …, and P105 for Participant 105 in the questionnaires filled by each participant. Moreover, all questionnaires filled by all 115 participants, including 105 respondents of the open-ended questions and the original result sheets of SPSS from different tests applied for this study are scanned and arranged in a single PDF file to share as a “Supplementary file”. Table 1 below indicates the demographic information of the participants.

**Table 1:** Demographic information of the participants.

Variable	Frequency	Percentage
Gender		
Male	46	40
Female	69	60
Age category		
22–27	46	40
28–33	43	37.4
34–39	15	13
40 +	11	9.6
Level of education		
Master	65	56.5
PhD	50	43.5
AI/ChatGPT experience		
Yes	107	93
No	8	7
Country		
Malaysian	49	42.6
China	49	42.6
Indonesia	5	4.3
Pakistan	5	4.3
Iran	2	1.7
Palestine	2	1.7
Jordan	1	0.9
Nigeria	1	0.9
UK	1	0.9

### Instruments

The AI and Mediated Intercultural Communication Questionnaire (AIMICQ) survey instrument was developed by the current study's primary author and with the help of the existing literature and ChatGPT, was used to collect the quantitative and qualitative data. The instrument includes 15 structured items and six (6) open-ended questions. The initial instrument included 30 items, and to reduce the number of items and dimensions and group them under their related dimensions and factors, we did a comprehensibility check with 19 participants, and also factor analysis under the dimension reduction analysis of SPSS was used. The number of items has been reduced to 15 items based on the comprehensibility check and through the use of factor analysis for the final quantitative instrument. Factor analysis enables researchers to shorten a group of multifaceted items and variables through the use of statistical procedures^27^. The existing literature strongly supports the use of factor analysis for construct validity^28^. Exploratory factor analysis is mostly used in instrument development of educational research, especially to measure variables that cannot be measured directly^29^.

The quantitative (structured) items were designed based on a Likert scale with five options per item, from strongly agree to strongly disagree. Likert^30^ introduced a broadly applicable five-category scale to measure attitudes. Based on the literature and factor analysis, the quantitative items are grouped under four attributes: “Attitudes towards AI” with three items, “AI benefits” with five items, “AI challenges” with four items, and “AI regulation” with three items. For the current work, the attitudes towards AI attribute refers to the personal attitudes of individuals, especially the participants towards the importance of AI and AI-related technologies, AI benefits attribute refers to the effects and outcomes of the application of AI and AI-related technologies in daily interactions among the participants, AI challenges attribute refers to the technical and ethical challenges and barriers of the use of AI and AI-related technologies, and AI regulation attribute refers to the probable professional and ethical rules and regulations regarding the application of AI and AI-powered technologies in university environments.

A pilot test was conducted through the participation of 19 participants to check the instrument's reliability. Based on the reliability test of the SPSS software, the Cronbach's Alpha score of the quantitative instrument from the pilot study was 0.804. Also, Cronbach's Alpha score for the quantitative items from the final survey, which includes 115 participants, is 0.769. A pilot test helps the researcher verify participant flow, data turnout, the procedure's effectiveness, and the participants' willingness^31^. To have the direct and detailed views of the participants, a package of six interview questions based on the literature and with the help of ChatGPT was designed and included at the end of the quantitative questionnaire. The reliability of open-ended questions was assessed based on the previous works. The critical perspectives of the interview instruments are replication and transparency^32^.

Moreover, to check the content validity of the instrument through expert validation, the instruments were sent to three experts from three interrelated areas (communication, language, and humanities technology) who are Associate Professors of two different Malaysian public universities, and two of them shared their comments and their comments were considered in the instrument revision. Researchers can ensure the content validity of their questionnaires through discussion with some experts from the related fields^32^. Based on the results from factor analysis, all 15 items of the manuscript are grouped under four attributes. Table 2 below includes attributes of the study and the number of items for each attribute.

**Table 2:** Study attributes and the number of items for each attribute.

Attribute	Number of items
AI attitudes	3
AI benefits	5
AI challenges	4
AI regulation	3

### Data analysis

Before the analysis and based on the results from the exploratory factor analysis, all 15 items of the quantitative instruments were grouped under four attributes using the Transform test of the SPSS. The mentioned attributes are Attitudes towards AI, AI benefits, AI challenges, and AI regulation. The descriptive statistics, reliability analysis, and Bivariate correlation tests of the 29th version of IBM-SPSS software were used to analyze the quantitative data. The descriptive test finds basic statistical information, such as frequencies, percentages, Mean scores, and Std. Deviation scores. The reliability analysis of the scale test of the SPSS was used to find out Cronbach's Alpha score for the instruments and the overall mean scores for the demographic variables of the study. The Bivariate correlation test was used to find out probable correlations between four attributes of the instrument.

Moreover, the inductive coding approach and the conceptual content analysis were used to code and analyze the qualitative data. Besides results from the content analysis, some of the participants' most relevant and detailed answers will be quoted directly in the findings section below.

### Ethical approval

For the research design, data collection procedures, data analysis, and data report, all research regulations of University of Malaya (UM) were considered and applied. The study was approved by the ethics committee of the Department of Media and Communication Studies of University of Malaya (UM). A letter of informed consent was obtained from all subjects and/or their legal guardian(s) before the data collection procedure.

## Results

The results section of the current manuscript includes findings from the analysis of both the quantitative and qualitative datasets.

### Quantitative results

The quantitative findings reported in the current work are results from descriptive, scale, and correlation tests of SPSS applied to analyze the quantitative dataset. The descriptive test was applied to find basic statistical information, such as frequencies, percentages, and Mean and Std. Deviation scores, and the scale test was applied to check the instrument reliability. The Bivariate correlation test was applied to find the probable correlations between four attributes constructed from 15 structured questionnaire items. To have the Mean/ Std. Deviation scores of all participants for each item of the quantitative instrument, the descriptive test of SPSS applied. Table 3 below indicates Mean/ Std. Deviation scores of all participants for each item.

**Table 3:** The mean/SD scores of all participants for each item.

Item	No of options	No of participants	Mean score	SD
1. AI and AI-powered machines can be helpful in bridging communication gaps between people from different cultures	5	115	1.9	0.90
2. So far, AI has been successful in facilitating cross-cultural understanding and collaboration in mediated communication	5	115	2.3	0.87
3. AI can help overcome language barriers and promote more inclusive communication among diverse groups	5	115	2.0	0.80
4. There are some cultural biases and stereotypes in AI-powered language tools and communication platforms	5	115	2.5	0.88
5. AI has affected the localization and adaptation of content for different cultural audiences in digital communication	5	115	2.5	0.81
6. I encountered some challenges in using AI-powered translation tools when communicating with people from different cultural backgrounds	5	115	2.8	0.98
7. AI has the potential to promote cultural exchange and appreciation by enabling easier access to diverse perspectives and information	5	115	1.9	0.81
8. AI-powered machines and chatbots should be programmed to respect and adapt to cultural norms and communication styles while interacting with users from different cultures	5	115	1.7	0.87
9. There are some potential risks and concerns regarding AI's role in cross-cultural communication, such as perpetuating cultural stereotypes and misinterpretations	5	115	2.1	0.97
10. I have participated in some cross-cultural virtual collaborations that relied on AI for communication and coordination	5	115	3.4	1.1
11. I think AI can enhance intercultural learning experiences and foster empathy among individuals from different cultural backgrounds	5	115	2.2	0.98
12. AI-powered virtual reality (VR) and augmented reality (AR) technologies can impact cross-cultural communication and understanding	5	115	2.1	0.87
13. Somehow AI-generated content may unintentionally offend and misled individuals from specific cultural backgrounds	5	115	2.3	0.99
14. Ethical considerations should be taken into account when designing AI-powered communication tools that cater to diverse cultural contexts	5	115	1.5	0.90
15. Universities and educational institutions leverage AI to create more inclusive and culturally diverse online learning environments	5	115	1.9	0.88

Moreover, before the analysis and based on factor analysis, all 15 structured items of the survey instrument were grouped under four attributes through the use of the Transform test of the SPSS. To find out the probable correlations between the attributes, the Bivariate correlation test of SPSS applied. Based on the results, the correlation between AI attitudes and AI benefits was 0.558; between AI attitudes and AI challenges was 0.167; between AI attitudes and AI regulation was 0.321; between AI benefits and AI Challenges 0.275; and between AI benefits and AI regulation was 0.577, and between AI challenges and AI regulation was 0.366. Table 4 below illustrates correlations between four attributes of the study. The results from the correlation test included in the table below indicate the effectiveness of positive attitudes towards AI and AI-related technologies and also AI regulation on AI application benefits.

**Table 4:** Correlations between four attributes of the study.

Attribute	AI attitude	AI benefits	AI challenges
AI attitude			
AI benefits	0.558*		
AI challenges	0.167*	0.275*	
AI regulation	0.321*	0.577*	0.366*

*C.I. Level: 95.0.

Based on the results, there were strong positive correlations between AI attitudes and AI benefits and AI benefits and AI regulations. Also, there were remarkable positive correlations between AI attitude and AI regulation, and between AI regulation and AI challenges. It means that positive attitudes regarding the application of AI and also regulating the application of AI increase the level of effectiveness of AI applications in daily interactions among different people. Based on the results, attitudes regarding AI affect AI regulation and vice versa, and in some cases AI regulation increases challenges in the application of AI, as may ask users to follow some particular rules.

### Qualitative results

The survey questionnaire included 15 structured and six open-ended items. All participants were asked to share/write down their points of view regarding the issues stated in the qualitative items. Of all 115 participants, 105 (91.3%) answered the open-ended questions, and the findings reported in the qualitative section of the results of this study are based on the written answers of 105 participants for six open-ended items/questions. To analyze and report the results from the open-ended questions, all the respondents' written answers/points of view were read carefully and thoroughly. The inductive coding approach was applied to code and categorize the written answers, and the conceptual content analysis was used to analyze and report the results. Table 5 below indicates the principal results and concepts from the conceptual content analysis. Each concept was obtained based on the content analysis of the participants' detailed answers to the qualitative questions of the study.

**Table 5:** The principal results and concepts from the conceptual content analysis.

Element/attribute	Number of participants mentioned	Percentage
People broadly use AI technologies	96	91.4
ChatGPT is part of modern academic tools	83	79
AI transcend language barriers	57	54.2
AI connects different cultures	92	87.6
AI bridges communication gaps	67	63.8
AI helps people to gain knowledge fast	45	42.8
AI is an essential part of modern research	28	26.6
AI helps different people to interact	74	70.4
AI helps people to know different cultures	49	46.6
AI helps to transcend technical barriers	86	81.9
AI helps to improve language skills	37	35.2
AI helps to have access to enormous sources	53	50.4
Regulation of AI is essential	71	67.6

Moreover, all 105 respondents to the open-ended questions were numbered P1, P2, …, and P105 in the questionnaires, and the given number will be mentioned when quoting the responses of some of them below. Based on the answers of the vast majority (91.4%) of the respondents who responded to the open-ended question, people, including them, broadly use AI technologies, especially ChatGPT, in their daily lives. Most (87.7%) of the respondents stated that AI and AI-powered technologies connect different cultures and help people of different cultures to be connected. For example, P1, a 24-year-old PhD student from China, said, “ChatGPT is like a Wikipedia for me, and I use it for searching everything related to life and study. For the study, I used ChatGPT for searching literature, definitions, etc. For daily life, I use it for solving every question.” Also, P3, a female 26-year-old Malaysian PhD student, said, “I believe that people from different cultures do not face any problem using AI and AI technologies, and it definitely could help people from different cultures to understand each other more.”

Based on the answers of most respondents, AI and AI technologies, such as ChatGPT, bridge language barriers between people who speak different languages and enable them to interact with one another even without knowing the language of their communication partner. For instance, P4, a 46-year-old female PhD student from Pakistan, stated, “AI helps people to communicate with each other despite language barriers, which helps them to interact and be familiarized with people of different regions.” Additionally, P 12, a 28-year-old female PhD student from China, said, “AI and AI machines break the language obstacles, break the space barrier, and offer knowledge to people to understand cultures.” She also said, “I used the translating App when I traveled to other countries when people could not understand English language.” Moreover, P3, a female 26 years old Malaysian PhD student, said, “AI did help us to overcome language barriers; for example, before AI, we used body language to communicate with other people, but now, with the help of AI, people can easily communicate using AI-translate. However, P1, a 24 years old PhD student from China, stated, “When AI translates some Chinese words to English, it sometimes translates to wrong words, as 'Ying Xiang in Chinese means impart and it is a noun, but AI usually translates it as a verb and with –ing, and it changes the meaning.”

Moreover, most of the participants reported using AI and AI technologies, such as ChatGPT, to overcome language and technical barriers and conduct their academic studies efficiently. For example, P16, a 38-year-old male PhD student from Pakistan, said, “I mostly use ChatGPT for searching information regarding my study.” Also, P19, a 35-year-old female PhD student from Indonesia, stated that she uses AI for educational purposes, saying, “I use AI for education purposes.” P2, a 34-year-old female PhD student from China, said, “I used AI for technical support for the library's website and eWallet app and ChatGPT to improve my English.” Moreover, P9, a 40-year-old male Maters' student from Iran, pointed out, “I usually use AI for educational and research purposes.”

Based on the results from the qualitative data, the majority (67.6%) of the participants focus on AI regulation and the consideration of ethical concerns of AI application in different aspects of life, especially academic activities. For instance, P7, a 27-year-old male PhD student from China, said, “The ethical concern should be considered an important part of AI abilities.” P13, a 36-year-old male PhD student from Pakistan, said, “AI is very effective if used in certain ethical and cultural domains.” Moreover, P11, a 28-year-old female PhD student from China, also said, “Universities can leverage AI to design creative and interesting content.” Results from both the quantitative and qualitative datasets indicate the importance and effectiveness of AI and AI technologies on different aspects of human life, especially human communication and modern education.

## Discussion

Communication, especially intercultural communication, is among modern life's main characteristics and requirements in the current ever-growing multicultural environments and organizations. Nowadays, to overcome time, space, and language barriers, people use modern technologies, including artificial intelligence (AI) to interact with one another and gain information about one another. Thus, modern technologies, especially AI and AI-related systems and technologies, broadly influence human communication. Based on some researchers and scholars, AI and AI technologies are broadly used in human communication and human communication is among the fields that are deeply influenced by AI and AI technologies^3–5^. It means that there are deep connections between human communication and AI technologies.

Previously, language, time and space barriers mostly undermined the speed, duration, and quality of intercultural interactions among different people, but by the use of AI and AI technologies, people can overcome the mentioned barriers and interact with one another with the help of an interpreter^3–7^. To say it in other words, AI and AI-related technologies help people to interact with one another and get information about one another without knowing different languages or being in the same place or at the same time when interacting with one another. The mentioned privileges are mostly applicable in intercultural communication. However, the results from the application of the mentioned technologies in daily interactions among different people may not always be demanding and free of challenges. The concern could be more serious when people of different languages and cultures interact with one another without knowing the language of one another and interacting through the help of AI technologies.

It is essential to know that, among different aspects of human communication, intercultural communication is an interesting and, at the same time, challenging type of communication, and the rapid growth and massive application of AI and AI technologies could raise some questions regarding the probable effects of the mentioned modern technologies on daily interactions among different people. One of the main questions and concerns regarding the mentioned issue is whether AI technologies make communication among people of different cultures easier or more complicated. To answer the question, the current study assessed the impacts of AI and AI technologies on interactions among postgraduate students in a multicultural university environment, and the participation of all participants was voluntary.

A mixed-methods survey using a survey instrument was applied to conduct this study, which had 115 participants from nine different countries. The qualitative items were added at the end of the questionnaire, and the participants were asked to share their points of view on the mentioned items. Of all the 115 participants, 105 of them shared their detailed written answers to the qualitative questions. The descriptive, reliability, and Bivariate correlation tests of the 29th version of the IBM-SPSS software were used to analyze the quantitative data. The inductive coding and conceptual content analysis were used to code and analyze the qualitative data.

Based on the descriptive results, the vast majority (93%) of the participants had already experienced the use of AI and AI technologies, especially ChatGPT. Based on the qualitative data, the participants from different cultures and countries mostly used AI and AI technologies to get information regarding different cultures, interact with individuals from different cultures, and do their university-related assignments and duties quickly and easily. Based on the results, AI systems and technologies, especially ChatGPT, helped and enabled the participants to know different perspectives and norms of other cultures and interact with people of other cultures who speak different languages through AI-powered translation and interpretation Apps and facilities. The results indicate that AI and AI technologies, especially ChatGPT, helped the participants overcome language and technical barriers, access enormous information sources, and improve their language and technical skills. Based on the mentioned results, currently, AI and AI technologies are the essential parts and requirements of daily interactions among people in modern multicultural environments and help them overcome different language, cultural, and technical barriers.

Based on the qualitative results, the vast majority (87.7%) of 105 respondents to the open-ended questions believed that AI and AI technologies connect different cultures, reduce cultural barriers, and help people of different cultures interact and connect. The mentioned findings support the findings and assertions of some researchers and scholars^4^^6^^8^, on the effectiveness of AI and AI technologies in human communication.

Using the Transform test of the SPSS software and based on the factor analysis and aim and structure of the study, all 15 structured items of the survey instrument were grouped under four attributes: AI attitudes, AI benefits, AI challenges, and AI regulation. The Bivariate correlation test was applied to determine the probable correlation between the attributes mentioned. The results showed strong positive correlations between AI attitudes and AI benefits, with a correlation score of 0.558, and AI regulation and AI benefits, with a score of 0.577. It means that the main attributes that affect AI benefits and outcomes are users' attitudes towards AI and AI technologies, and the ways people use AI and AI technologies.

Moreover, based on the results from the conceptual content analysis of the qualitative data, nowadays, people broadly use AI technologies. AI technologies, especially ChatGPT, are essential to their personal and professional lives. Based on the results, AI and AI technologies connect different cultures, transcend language barriers, and help people to know and interact with people of different cultures. The results indicated that AI and AI technologies bridge communication gaps between people and help them gain and apply new knowledge in different aspects of their lives. The above-mentioned results are supportive of the findings and assertions of some researchers and scholars^3–7^, on the profound influence and effects of AI and AI-powered technologies on daily interactions among people from different cultures, especially in helping them to overcome language barriers. Furthermore, based on the results from the conceptual content analysis, the consideration of ethical issues and regulation of AI applications in different aspects of human life, especially human communication, should be among the priorities of both the producers and users of AI and AI technologies.

As the main contributions, findings from the current study confirmed the effectiveness of AI and AI-powered technologies on interactions among people from different cultures, connecting different cultures, and reducing language and culture barriers among people from different cultures. Also, the results indicate the effectiveness of positive attitudes towards AI and AI-related technologies and AI regulation on the increase of helpful outcomes of the application of AI and AI-related technologies in daily interactions among different people. The findings reported in the current study are new and could be helpful for communicators and researchers of the related areas.

## Conclusion

The current study assessed the impacts of artificial intelligence (AI) and AI technologies on intercultural communication among postgraduate students from different nationalities in a multicultural university environment. A mixed-methods survey, using a newly developed survey instrument, was applied to conduct this study. This study had 115 participants from postgraduate students of a Malaysian top public university, and the participants were from nine different countries. Of all 115 participants of the study, 105 of them shared their detailed points of view for the open-ended questions as well. The descriptive, reliability, and Bivariate correlation tests of SPSS software were used to analyze the quantitative data. The inductive coding and conceptual content analysis were used to code and analyze the qualitative data.

Based on descriptive results from the current study, the vast majority (93%) of the participants already experienced using AI and AI technologies, especially ChatGPT. The vast majority (87.7%) of 105 respondents to the open-ended questions believed that AI and AI technologies connect different cultures, reduce cultural barriers, and help people of different cultures interact and connect. Based on their qualitative answers, the participants from different cultures and countries mostly used AI and AI technologies to overcome language barriers, get information regarding different cultures, interact with individuals from different cultures, and do their university-related assignments and duties quickly and easily. Based on the results, AI systems and technologies, especially ChatGPT, helped and enabled the participants to know different perspectives and norms of other cultures and interact with people of other cultures who speak different languages through AI-powered translation and interpretation Apps and facilities.

Using the Transform test of the SPSS software and based on the factor analysis and aim and structure of the study, all 15 structured items of the survey instrument were grouped under four attributes: AI attitudes, AI benefits, AI challenges, and AI regulation. Based on the results from the correlation test, there were strong positive correlations between AI attitudes and AI benefits with a correlation score of 0.558, and AI regulation and AI benefits with a score of 0.577. It means that the main attributes that affect AI benefits and outcomes are users' attitudes towards AI and AI technologies and the ways that people use AI and AI technologies. Based on the qualitative results, considering ethical issues and regulating the use of AI and AI technologies could enhance the effectiveness of AI and AI technologies in daily communication among people from different cultures and countries in the ever-growing multicultural environments and organizations. Findings from the current study could add some helpful information to the literature and be helpful for communicators and researchers.

### Implications and limitations

Because of the need for more evidence on the outcomes of the massive application of AI and AI technologies on intercultural communication, the current study assessed the impacts of AI and AI technologies on daily interactions among postgraduate students from different cultures and countries. The results from this study include some new and helpful information regarding the impacts of AI and AI technologies on intercultural communication in the ever-growing multicultural environments and organizations. The results from the current study could be used by communicators, researchers, and policymakers to enhance the effectiveness of the use of AI and AI technologies in daily interaction with/ among different people. The instrument and structure of the current study could be used in similar studies in different multicultural environments and organizations.

The main limitation of the current study was the absence of pre-established and well-tested instruments to assess the impacts of AI and AI technologies on intercultural communication. Through a lengthy and time-consuming procedure, a new instrument has been developed by the corresponding author and used in the current study. Another primary limitation was the presence of a relatively small number of postgraduate students on campus, as after the COVID-19 pandemic, most of the postgraduate students stayed in their home countries and hometowns and did their studies online and visited the university based on their own or their supervisors' demand. Applying the same or similar study with more participants may bring more exciting results.

### Supplementary Information

Supplementary Information.

## Data Availability

All data generated or analyzed during this study are included in this published article and its supplementary information file.
