# Supplementary material for: Assessment of the impacts of artificial intelligence (AI) on intercultural communication among postgraduate students in a multicultural university environment
Source: Sci Rep. 2024 Jun 15;14:13849. doi: 10.1038/s41598-024-63276-5 (PMC11180179; doi:10.1038/s41598-024-63276-5)
Supplement: Supplementary file 1 — Supplementary Information. [file 41598_2024_63276_MOESM1_ESM.pdf]

Original Results from the SPSS Tests applied in the study, Survey Instrument, and the Scanned Copies of all Questionnaires Filled and Answered by the Participants

Reliability result of pilot study with 19 participants

Case Processing Summary

		N	%
Cases	Valid	19	100.0
	Excluded[a]	0	.0
	Total	19	100.0

a. Listwise deletion based on all variables in the procedure.

Reliability Statistics

Cronbach's Alpha	N of Items
.804	15

Reliability result of final survey with 115 participants

Case Processing Summary

		N	%
Cases	Valid	115	100.0
	Excluded[a]	0	.0
	Total	115	100.0

a. Listwise deletion based on all variables in the procedure.

Reliability Statistics

Cronbach's Alpha	N of Items
.769	15

Results from Descriptive Tests

Case Processing Summary

		N	%
Cases	Valid	115	100.0
	Excluded[a]	0	.0
	Total	115	100.0

a. Listwise deletion based on all variables in the procedure.

Reliability Statistics

Cronbach's Alpha	N of Items
.769	15

Frequencies

Statistics

		Gender of Participants	Age of Participants	Nationality of Participants	Level of Education	AI and ChatGPT Experience of PArticipants	Question 1	Question 2	Question 3	Question 4	Question 5	Que[illegible]
N	Valid	115	115	115	115	115	115	115	115	115	115	
	Missing	0	0	0	0	0	0	0	0	0	0	
Mean		1.60	1.92	2.05	1.43	1.08	1.97	2.39	2.02	2.50	2.51	
Median		2.00	2.00	2.00	1.00	1.00	2.00	2.00	2.00	3.00	2.00	
Std. Deviation		.492	.956	1.583	.498	.301	.903	.876	.805	.882	.810	
[illegible]		[illegible]	[illegible]	[illegible]	[illegible]	[illegible]	[illegible]	[illegible]	[illegible]	[illegible]	[illegible]	

[illegible]on 2	Question 3	Question 4	Question 5	Question 6	Question 7	Question 8	Question 9	Question 10	Question 11	Question 12	Question 13	Question 14	Question 15
115	115	115	115	115	115	115	115	115	115	115	115	115	115
0	0	0	0	0	0	0	0	0	0	0	0	0	0
2.39	2.02	2.50	2.51	2.83	1.97	1.72	2.19	3.40	2.26	2.17	2.35	1.58	1.96
2.00	2.00	3.00	2.00	3.00	2.00	2.00	2.00	4.00	2.00	2.00	2.00	1.00	2.00
.876	.805	.882	.810	.982	.816	.874	.972	1.168	.983	.878	.992	.908	.882

Item Statistics

	Mean	Std. Deviation	N
Question 1	1.97	.903	115
Question 2	2.39	.876	115
Question 3	2.02	.805	115
Question 4	2.50	.882	115
Question 5	2.51	.810	115
Question 6	2.83	.982	115
Question 7	1.97	.816	115

Question 8	1.72	.874	115
Question 9	2.19	.972	115
Question 10	3.40	1.168	115
Question 11	2.26	.983	115
Question 12	2.17	.878	115
Question 13	2.35	.992	115
Question 14	1.58	.908	115
Question 15	1.96	.882	115

Descriptive Statistics

	N	Mean	Std. Deviation
AI_Attitude	115	2.45	.592
AI_Benifits	115	2.27	.561
AI_Challenges	115	2.47	.621
AI_Regulation	115	1.75	.681
Valid N (listwise)	115		

Results of scale test for overall mean scores of demographic variables for all items

All Participants

Case Processing Summary

		N	%
Cases	Valid	115	100.0
	Excluded[a]	0	.0
	Total	115	100.0

a. Listwise deletion based on all variables in the procedure.

Scale Statistics

Mean	Variance	Std. Deviation	N of Items
33.82	44.923	6.702	15

Female participants

Case Processing Summary

		N	%
Cases	Valid	69	100.0
	Excluded[a]	0	.0
	Total	69	100.0

a. Listwise deletion based on all variables in the procedure.

Scale Statistics

Mean	Variance	Std. Deviation	N of Items
33.46	32.370	5.689	15

Male Participants

Case Processing Summary

		N	%
Cases	Valid	46	100.0
	Excluded[a]	0	.0
	Total	46	100.0

a. Listwise deletion based on all variables in the procedure.

Scale Statistics

Mean	Variance	Std. Deviation	N of Items
34.35	64.410	8.026	15

MA participants

Case Processing Summary

		N	%
Cases	Valid	65	100.0
	Excluded[a]	0	.0
	Total	65	100.0

a. Listwise deletion based on all variables in the procedure.

Scale Statistics

Mean	Variance	Std. Deviation	N of Items
33.89	48.910	6.994	15

PhD

Case Processing Summary

		N	%
Cases	Valid	51	100.0
	Excluded[a]	0	.0
	Total	51	100.0

a. Listwise deletion based on all variables in the procedure.

Scale Statistics

Mean	Variance	Std. Deviation	N of Items
33.57	40.970	6.401	15

Correlation test results

Correlations

Variable	Variable2	Correlation	Count	Statistic Lower C.I.	Upper C.I.	Notes
AI_Attitude	AI_Attitude	1.000	115	--	--	
	AI_Benifits	.558	115	.418	.673	
	AI_Challenges	.167	115	-.016	.340	
	AI_Regulation	.321	115	.146	.476	
AI_Benifits	AI_Attitude	.558	115	.418	.673	
	AI_Benifits	1.000	115	--	--	
	AI_Challenges	.275	115	.096	.436	
	AI_Regulation	.577	115	.441	.688	
AI_Challenges	AI_Attitude	.167	115	-.016	.340	
	AI_Benifits	.275	115	.096	.436	
	AI_Challenges	1.000	115	--	--	
	AI_Regulation	.366	115	.196	.515	
AI_Regulation	AI_Attitude	.321	115	.146	.476	
	AI_Benifits	.577	115	.441	.688	
	AI_Challenges	.366	115	.196	.515	
	AI_Regulation	1.000	115	--	--	

Missing value handling: PAIRWISE, EXCLUDE. C.I. Level: 95.0

UNIVERSITI MALAYA

Department of Media and Communication Studies

AI and Mediated Intercultural Communication Questionnaire

Dear Student/ Researcher,

Through this survey, I want to assess the probable effects of Artificial Intelligence (AI) on mediated communication among people from different cultures. This instrument which been developed by the help of the existing literature and ChatGPT, has 15 structured items with five (5) options per item and six (6) open-ended questions. Your participation in the survey is voluntary and the personal identifications of the participants will be treated as strictly confidential.

Thank you for your cooperation.

Dr. Abdul Qahar Sarwari,
Post-Doctoral Research Fellow
Department of Media and Communication Studies
University of Malaya

A. Demographic Information

1. Gender: ☐ Male ☐ Female

2. Age:

3. Nationality:

4. Level of education:

5. AI/ ChatGPT experience: ☐ Yes ☐ No

B. AI and ICC Scale

**Directions:** The current AI-ICC questionnaire includes 15 items/ statements with five (5) options per item, as: 1 – Strongly agree; 2 – Agree, 3 – Uncertain; 4 – Disagree, and 5 – Strongly disagree.	Strongly agree	Agree	Uncertain	Disagree	Strongly disagree
1. AI and AI-powered machines can be helpful in bridging communication gaps between people from different cultures.	1	2	3	4	5
2. So far, AI has been successful in facilitating cross-cultural understanding and collaboration in mediated communication.	1	2	3	4	5
3. AI can help overcome language barriers and promote more inclusive communication among diverse groups.	1	2	3	4	5
4. There are some cultural biases and stereotypes in AI-powered language tools and communication platforms.	1	2	3	4	5
5. AI has affected the localization and adaptation of content for different cultural audiences in digital communication.	1	2	3	4	5
6. I encountered some challenges in using AI-powered translation tools when communicating with people from different cultural backgrounds.	1	2	3	4	5
7. AI has the potential to promote cultural exchange and appreciation by enabling easier access to diverse perspectives and information.	1	2	3	4	5
8. AI-powered machines and chatbots should be programmed to respect and adapt to cultural norms and communication styles while interacting with users from different cultures.	1	2	3	4	5
9. There are some potential risks and concerns regarding AI's role in cross-cultural communication, such as perpetuating cultural stereotypes and misinterpretations.	1	2	3	4	5
10. I have participated in some cross-cultural virtual collaborations that relied on AI for communication and coordination.	1	2	3	4	5
11. I think AI can enhance intercultural learning experiences and foster empathy among individuals from different cultural backgrounds.	1	2	3	4	5
12. AI-powered virtual reality (VR) and augmented reality (AR) technologies can impact cross-cultural communication and understanding.	1	2	3	4	5
13. Somehow AI-generated content may unintentionally offend and misled individuals from specific cultural backgrounds.	1	2	3	4	5
14. Ethical considerations should be taken into account when designing AI-powered communication tools that cater to diverse cultural contexts.	1	2	3	4	5
15. Universities and educational institutions leverage AI to create more inclusive and culturally diverse online learning environments.	1	2	3	4	5

C. Open-ended Questions

In the case of your agreement on sharing your answer/ points of views for questions below, please write down your comment/ point of view for all questions or any question that you prefer:

1. Did you experience the use of AI and AI-powered chatbots, such as ChatGPT and for what purpose?
2. How do you perceive the role of AI and AI-powered machines in bridging communication gaps between people from different cultures?
3. Have you experienced and observed any instances where AI has been successful in facilitating cross-cultural understanding and collaboration in mediated communication?
4. In what ways do you think AI can help overcome language barriers and promote more inclusive communication among diverse groups?
5. Are there any cultural biases or stereotypes that you have noticed in AI-powered language tools or communication platforms?
6. How can universities and educational institutions leverage AI to create more inclusive and culturally diverse online learning environments?

Thank you for your time and participation

The 2nd part of the supplementary document below is the scanned copies of all questionnaires filled and answered by the participants

Participant No (01) & P(1)

UNIVERSITI MALAYA

Depart of Media and Communication Studies

AI and Mediated Intercultural Communication Questionnaire

Dear UM Student/ Researcher,

Through this survey, we want to assess the probable effects of Artificial Intelligence (AI) on mediated communication among people from different cultures. This instrument which been developed by the help of the existing literature and ChatGPT, has 15 structured items with five (5) options per item and six (6) open-ended questions. Your participation in the survey is voluntary and the personal identifications of the participants will be treated as strictly confidential.

Thank you for your cooperation.

The researchers

A. Demographic Information

1. Gender: ☐ Male ☑ Female

2. Age: 28

3. Nationality: Chinese

4. Level of education: phd

5. AI/ ChatGPT experience: ☑ Yes ☐ No

B. Intercultural Sensitivity Scale

**Directions:** The current AI-ICC questionnaire includes 15 items/ statements with five (5) options per item, as: 1 – Strongly agree; 2 – Agree, 3 – Uncertain; 2 – Disagree, and 5 – Strongly disagree.	Strongly agree	Agree	Uncertain	Disagree	Strongly disagree
1. AI and AI-powered machines can be helpful in bridging communication gaps between people from different cultures.	1	2 ✓	3	4	5
2. So far, AI has been successful in facilitating cross-cultural understanding and collaboration in mediated communication.	1	2 ✓	3	4	5
3. AI can help overcome language barriers and promote more inclusive communication among diverse groups.	1	2 ✓	3	4	5

CamScanner

PNo-(01) & P(2)

4. There some cultural biases and stereotypes in AI-powered language tools and communication platforms.	1	2	3 ✓	4	5
5. AI has affected the localization and adaptation of content for different cultural audiences in digital communication.	1	2	3 ✓	4	5
6. I encountered any challenges in using AI-powered translation tools when communicating with people from different cultural backgrounds.	1	2	3	4 ✓	5
7. AI has the potential to promote cultural exchange and appreciation by enabling easier access to diverse perspectives and information.	1	2 ✓	3	4	5
8. AI-powered machines and chatbots should be programmed to respect and adapt to cultural norms and communication styles while interacting with users from different cultures.	1 ✓	2	3	4	5
9. There are some potential risks and concerns regarding AI's role in cross-cultural communication, such as perpetuating cultural stereotypes and misinterpretations.	1	2	3 ✓	4	5
10. I have participated in some cross-cultural virtual collaborations that relied on AI for communication and coordination.	1	2 ✓	3	4	5
11. I think AI can enhance intercultural learning experiences and foster empathy among individuals from different cultural backgrounds.	1	2 ✓	3	4	5
12. AI-powered virtual reality (VR) and augmented reality (AR) technologies can impact cross-cultural communication and understanding.	1	2	3 ✓	4	5
13. Somehow AI-generated content may unintentionally offend and misled individuals from specific cultural backgrounds.	1	2	3 ✓	4	5
14. Ethical considerations should be taken into account when designing AI-powered communication tools that cater to diverse cultural contexts.	1	2 ✓	3	4	5
15. Universities and educational institutions leverage AI to create more inclusive and culturally diverse online learning environments.	1	2 ✓	3	4	5

In the case of your agreement on sharing your answer/ points of views for questions below, please write down your comment/ point of view for all questions or any question that you prefer:

1. Did you experience the use of AI and AI-powered chatbots, such as ChatGPT and for what purpose?

- I used the translating App when I traveled ~~in~~ other countries where peop[illegible] can not understand English
- chatGPT: get some idea in my research field.

2. How do you perceive the role of AI and AI-powered machines in bridging communication gaps between people from different cultures?

AI and AI powered machines ~~do~~ do bridge communication gaps between people from different cultures:

- break the language limit/obstacle
- break the space/location limit/obstacle
- offer knowledge to help people understand the different culture. world tog[illegible]

[illegible]

CamScanner

P01 & P3

3. Have you experienced and observed any instances where AI has been successful in facilitating cross-cultural understanding and collaboration in mediated communication?

YouTube offer the ~~instant~~ translating, and also google meeting, etc.

4. In what ways do you think AI can help overcome language barriers and promote more inclusive communication among diverse groups?

- AI is the tool to overcome the language barriers.
- promote / ~~or~~ innovate more AI Apps.

5. Are there any cultural biases or stereotypes that you have noticed in AI-powered language tools or communication platforms?

I am only ~~fa~~ familiar with ChatGPT, I think it's always offer inclusive and informative content, since it has huge database. didn't notice the ontheure biases / stereotypes.

6. How can universities and educational institutions leverage AI to create more inclusive and culturally diverse online learning environments?

- facilities is ~~the~~ basic
- enough teaching staff who have ~~sufficient~~ professional AI knowledge
- running more online learning activities

Thank you for your time and participation

CamScanner

Participant No (02) E, P(1)

UNIVERSITI
MALAYA

Depart of Media and Communication Studies

AI and Mediated Intercultural Communication Questionnaire

Dear UM Student/ Researcher,

Through this survey, we want to assess the probable effects of Artificial Intelligence (AI) on mediated communication among people from different cultures. This instrument which been developed by the help of the existing literature and ChatGPT, has 15 structured items with five (5) options per item and six (6) open-ended questions. Your participation in the survey is voluntary and the personal identifications of the participants will be treated as strictly confidential.

Thank you for your cooperation.

The researchers

A. Demographic Information

1. Gender: ☑ Male ☐ Female

2. Age: 36

3. Nationality: Pakistani

4. Level of education: Post Graduate PhD

5. AI/ ChatGPT experience: ☑ Yes ☐ No

B. Intercultural Sensitivity Scale

**Directions:** The current AI-ICC questionnaire includes 15 items/ statements with five (5) options per item, as: 1 – Strongly agree; 2 – Agree, 3 – Uncertain; 4 – Disagree, and 5 – Strongly disagree.	Strongly agree	Agree	Uncertain	Disagree	Strongly disagree
1. AI and AI-powered machines can be helpful in bridging communication gaps between people from different cultures.	1 ✓	2	3	4	5
2. So far, AI has been successful in facilitating cross-cultural understanding and collaboration in mediated communication.	1 ✓	2	3	4	5
3. AI can help overcome language barriers and promote more inclusive communication among diverse groups.	1 ✓	2	3	4	5

CamScanner

P-02 & P(2)

4. There some cultural biases and stereotypes in AI-powered language tools and communication platforms.	1	2	3 ✓	4	5
5. AI has affected the localization and adaptation of content for different cultural audiences in digital communication.	1	2	3 ✓	4	5
6. I encountered any challenges in using AI-powered translation tools when communicating with people from different cultural backgrounds.	1	2	3	4 ✓	5
7. AI has the potential to promote cultural exchange and appreciation by enabling easier access to diverse perspectives and information.	1	2 ✓	3	4	5
8. AI-powered machines and chatbots should be programmed to respect and adapt to cultural norms and communication styles while interacting with users from different cultures.	1 ✓	2	3	4	5
9. There are some potential risks and concerns regarding AI's role in cross-cultural communication, such as perpetuating cultural stereotypes and misinterpretations.	1	2	3 ✓	4	5
10. I have participated in some cross-cultural virtual collaborations that relied on AI for communication and coordination.	1	2	3	4 ✓	5
11. I think AI can enhance intercultural learning experiences and foster empathy among individuals from different cultural backgrounds.	1	2 ✓	3	4	5
12. AI-powered virtual reality (VR) and augmented reality (AR) technologies can impact cross-cultural communication and understanding.	1	2 ✓	3	4	5
13. Somehow AI-generated content may unintentionally offend and misled individuals from specific cultural backgrounds.	1	2	3	4 ✓	5
14. Ethical considerations should be taken into account when designing AI-powered communication tools that cater to diverse cultural contexts.	1 ✓	2	3	4	5
15. Universities and educational institutions leverage AI to create more inclusive and culturally diverse online learning environments.	1 ✓	~~2~~	3	4	5

In the case of your agreement on sharing your answer/ points of views for questions below, please write down your comment/ point of view for all questions or any question that you prefer:

1. Did you experience the use of AI and AI-powered chatbots, such as ChatGPT and for what purpose?

   one time, I used ChatGPT To get some information about some ~~retat~~ research related activities

2. How do you perceive the role of AI and AI-powered machines in bridging communication gaps between people from different cultures?

   It is very Effective if use in certain Ethical and cultural domain.

CS CamScanner

P(02) & P(3)

3. Have you experienced and observed any instances where AI has been successful in facilitating cross-cultural understanding and collaboration in mediated communication?

No - I deo not have any such Experience.

4. In what ways do you think AI can help overcome language barriers and promote more inclusive communication among diverse groups?

If AI can be developed in different Languages.

5. Are there any cultural biases or stereotypes that you have noticed in AI-powered language tools or communication platforms?

No - Idea.

6. How can universities and educational institutions leverage AI to create more inclusive and culturally diverse online learning environments?

Universities and Education institutions have Sophisticated Technologies as well as

Thank you for your time and participation

highly Equiped human Resources So it is Quite easy for them TO develop a Good Learning Environments where different cultures are included.

CamScanner

Participant No (03) & P(1)

UNIVERSITI MALAYA

Depart of Media and Communication Studies

AI and Mediated Intercultural Communication Questionnaire

Dear UM Student/ Researcher,

Through this survey, we want to assess the probable effects of Artificial Intelligence (AI) on mediated communication among people from different cultures. This instrument which been developed by the help of the existing literature and ChatGPT, has 15 structured items with five (5) options per item and six (6) open-ended questions. Your participation in the survey is voluntary and the personal identifications of the participants will be treated as strictly confidential.

Thank you for your cooperation.

The researchers

A. Demographic Information

1. Gender: ☑ Male ☐ Female

2. Age: 34

3. Nationality: Iran

4. Level of education: M.S (Master)

5. AI/ ChatGPT experience: ☑ Yes ☐ No

B. Intercultural Sensitivity Scale

**Directions:** The current AI-ICC questionnaire includes 15 items/ statements with five (5) options per item, as: 1 – Strongly agree; 2 – Agree, 3 – Uncertain; 4 – Disagree, and 5 – Strongly disagree.	Strongly agree	Agree	Uncertain	Disagree	[illegible]
1. AI and AI-powered machines can be helpful in bridging communication gaps between people from different cultures.	1	✓	3	4	
2. So far, AI has been successful in facilitating cross-cultural understanding and collaboration in mediated communication.	1	2	3 ✓	4	
3. AI can help overcome language barriers and promote more inclusive communication among diverse groups.	1	2 ✓	3	4	

CS CamScanner

No (03) E, P(2)

4. There some cultural biases and stereotypes in AI-powered language tools and communication platforms.	1	2 ✓	3	4	5
5. AI has affected the localization and adaptation of content for different cultural audiences in digital communication.	1	2 ✓	3	4	5
6. I encountered any challenges in using AI-powered translation tools when communicating with people from different cultural backgrounds.	1	2 ✓	3	4	5
7. AI has the potential to promote cultural exchange and appreciation by enabling easier access to diverse perspectives and information.	1	2 ✓	3	4	5
8. AI-powered machines and chatbots should be programmed to respect and adapt to cultural norms and communication styles while interacting with users from different cultures.	1	2 ✓	3	4	5
9. There are some potential risks and concerns regarding AI's role in cross-cultural communication, such as perpetuating cultural stereotypes and misinterpretations.	1	2 ✓	3	4	5
10. I have participated in some cross-cultural virtual collaborations that relied on AI for communication and coordination.	1	~~2~~	3	✓ 4	5
11. I think AI can enhance intercultural learning experiences and foster empathy among individuals from different cultural backgrounds.	1	2	3 ✓	4	5
12. AI-powered virtual reality (VR) and augmented reality (AR) technologies can impact cross-cultural communication and understanding.	1	2	3 ✓	4	5
13. Somehow AI-generated content may unintentionally offend and misled individuals from specific cultural backgrounds.	1	2	3 ✓	4	5
14. Ethical considerations should be taken into account when designing AI-powered communication tools that cater to diverse cultural contexts.	1	2 ✓	3	4	5
15. Universities and educational institutions leverage AI to create more inclusive and culturally diverse online learning environments.	1	2 ✓	3	4	5

In the case of your agreement on sharing your answer/ points of views for questions below, please write down your comment/ point of view for all questions or any question that you prefer:

1. Did you experience the use of AI and AI-powered chatbots, such as ChatGPT and for what purpose?

I use it for writing research articals

2. How do you perceive the role of AI and AI-powered machines in bridging communication gaps between people from different cultures?

The AI powered Machines communicate in a language that is beyond any race and culture. This is how homogenity b/w different cultures.

CS CamScanner

No (03) & (P3)

3. Have you experienced and observed any instances where AI has been successful in facilitating cross-cultural understanding and collaboration in mediated communication?

The knowledge + for instance, is in single language but but can be a single platform to mediate between different culture,

4. In what ways do you think AI can help overcome language barriers and promote more inclusive communication among diverse groups?

The AIs are available is almost every major language but they translate same knowledge, so the language barriers between cultures is removed

5. Are there any cultural biases or stereotypes that you have noticed in AI-powered language tools or communication platforms?

Mostly. The AI forums lik ChatGTP, avoid on answering any stereotyped or negative opinion about any group.

6. How can universities and educational institutions leverage AI to create more inclusive and culturally diverse online learning environments?

Not certain.

Thank you for your time and participation

CS CamScanner

Participant No 04 E Pⓘ

UNIVERSITI MALAYA

Depart of Media and Communication Studies

AI and Mediated Intercultural Communication Questionnaire

Dear UM Student/ Researcher,

Through this survey, we want to assess the probable effects of Artificial Intelligence (AI) on mediated communication among people from different cultures. This instrument which been developed by the help of the existing literature and ChatGPT, has 15 structured items with five (5) options per item and six (6) open-ended questions. Your participation in the survey is voluntary and the personal identifications of the participants will be treated as strictly confidential.

Thank you for your cooperation.

The researchers

A. Demographic Information

1. Gender: ☒ Male ☐ Female

2. Age: 41

3. Nationality: Jordan

4. Level of education: Master degree

5. AI/ ChatGPT experience: ☒ Yes ☐ No

B. Intercultural Sensitivity Scale

**Directions:** The current AI-ICC questionnaire includes 15 items/ statements with five (5) options per item, as: 1 – Strongly agree; 2 – Agree, 3 – Uncertain; 4 – Disagree, and 5 – Strongly disagree.	Strongly agree	Agree	Uncertain	Disagree	Strongly disagree
1. AI and AI-powered machines can be helpful in bridging communication gaps between people from different cultures.	1	2	3	4	⑤
2. So far, AI has been successful in facilitating cross-cultural understanding and collaboration in mediated communication.	1	2	③	4	5
3. AI can help overcome language barriers and promote more inclusive communication among diverse groups.	1	②	3	4	5

CamScanner

No (24) Ep PO
04

4. There some cultural biases and stereotypes in AI-powered language tools and communication platforms.	1	2	(3)	4	5
5. AI has affected the localization and adaptation of content for different cultural audiences in digital communication.	1	2	(3)	4	5
6. I encountered any challenges in using AI-powered translation tools when communicating with people from different cultural backgrounds.	1	2	(3)	4	5
7. AI has the potential to promote cultural exchange and appreciation by enabling easier access to diverse perspectives and information.	1	2	3	4	(5)
8. AI-powered machines and chatbots should be programmed to respect and adapt to cultural norms and communication styles while interacting with users from different cultures.	(1)	2	3	4	5
9. There are some potential risks and concerns regarding AI's role in cross-cultural communication, such as perpetuating cultural stereotypes and misinterpretations.	(1)	2	3	4	5
10. I have participated in some cross-cultural virtual collaborations that relied on AI for communication and coordination.	1	2	(3)	4	5
11. I think AI can enhance intercultural learning experiences and foster empathy among individuals from different cultural backgrounds.	1	2	3	4	(5)
12. AI-powered virtual reality (VR) and augmented reality (AR) technologies can impact cross-cultural communication and understanding.	(1)	2	3	4	5
13. Somehow AI-generated content may unintentionally offend and misled individuals from specific cultural backgrounds.	1	(2)	3	4	5
14. Ethical considerations should be taken into account when designing AI-powered communication tools that cater to diverse cultural contexts.	(1)	2	3	4	5
15. Universities and educational institutions leverage AI to create more inclusive and culturally diverse online learning environments.	1	2	3	(4)	5

In the case of your agreement on sharing your answer/ points of views for questions below, please write down your comment/ point of view for all questions or any question that you prefer:

1. Did you experience the use of AI and AI-powered chatbots, such as ChatGPT and for what purpose?

yes, I did. I use it to explain some of text. moreover I use it to traslate something I could not understand

2. How do you perceive the role of AI and AI-powered machines in bridging communication gaps between people from different cultures?

I think the face-to-face communication has vital role in bridging communication. ~~It~~ ~~[illegible]~~ it might use it to translate another language

CamScanner

No (20) Ec P(3)

3. Have you experienced and observed any instances where AI has been successful in facilitating cross-cultural understanding and collaboration in mediated communication?

no, I have not experienced that

4. In what ways do you think AI can help overcome language barriers and promote more inclusive communication among diverse groups?

I agree that AI would help to overcome language barriers.

5. Are there any cultural biases or stereotypes that you have noticed in AI-powered language tools or communication platforms?

6. How can universities and educational institutions leverage AI to create more inclusive and culturally diverse online learning environments?

the universities must support the programmer to do what they should do

Thank you for your time and participation

CamScanner

Participant No (05) & P(1)
(05)

UNIVERSITI MALAYA

Depart of Media and Communication Studies

AI and Mediated Intercultural Communication Questionnaire

Dear UM Student/ Researcher,

Through this survey, we want to assess the probable effects of Artificial Intelligence (AI) on mediated communication among people from different cultures. This instrument which been developed by the help of the existing literature and ChatGPT, has 15 structured items with five (5) options per item and six (6) open-ended questions. Your participation in the survey is voluntary and the personal identifications of the participants will be treated as strictly confidential.

Thank you for your cooperation.

The researchers

A. Demographic Information

1. Gender: ☑ Male ☐ Female

2. Age: 33

3. Nationality: MALAYSIAN

4. Level of education: MASTER

5. AI/ ChatGPT experience: ☑ Yes ☐ No

B. Intercultural Sensitivity Scale

Directions: The current AI-ICC questionnaire includes 15 items/ statements with five (5) options per item, as: 1 – Strongly agree; 2 – Agree, 3 – Uncertain; 4 – Disagree, and 5 – Strongly disagree.	Strongly agree	Agree	Uncertain	Disagree	Strongly disagree
1. AI and AI-powered machines can be helpful in bridging communication gaps between people from different cultures.	1	2 ✓	3	4	5
2. So far, AI has been successful in facilitating cross-cultural understanding and collaboration in mediated communication.	1	2	3 ✓	4	5
3. AI can help overcome language barriers and promote more inclusive communication among diverse groups.	1	2 ✓	3	4	5

CamScanner

No (08) & R2)

	1	2	3	4	5
4. There some cultural biases and stereotypes in AI-powered language tools and communication platforms.	1	2	3	4	5
5. AI has affected the localization and adaptation of content for different cultural audiences in digital communication.	1	2	3	4	5
6. I encountered any challenges in using AI-powered translation tools when communicating with people from different cultural backgrounds.	1	2	3	4	5
7. AI has the potential to promote cultural exchange and appreciation by enabling easier access to diverse perspectives and information.	1	2	3	4	5
8. AI-powered machines and chatbots should be programmed to respect and adapt to cultural norms and communication styles while interacting with users from different cultures.	1	2	3	4	5
9. There are some potential risks and concerns regarding AI's role in cross-cultural communication, such as perpetuating cultural stereotypes and misinterpretations.	1	2	3	4	5
10. I have participated in some cross-cultural virtual collaborations that relied on AI for communication and coordination.	1	2	3	4	5
11. I think AI can enhance intercultural learning experiences and foster empathy among individuals from different cultural backgrounds.	1	2	3	4	5
12. AI-powered virtual reality (VR) and augmented reality (AR) technologies can impact cross-cultural communication and understanding.	1	2	3	4	5
13. Somehow AI-generated content may unintentionally offend and misled individuals from specific cultural backgrounds.	1	2	3	4	5
14. Ethical considerations should be taken into account when designing AI-powered communication tools that cater to diverse cultural contexts.	1	2	3	4	5
15. Universities and educational institutions leverage AI to create more inclusive and culturally diverse online learning environments.	1	2	3	4	5

In the case of your agreement on sharing your answer/ points of views for questions below, please write down your comment/ point of view for all questions or any question that you prefer:

1. Did you experience the use of AI and AI-powered chatbots, such as ChatGPT and for what purpose?

Yes, For Academia, Research &, education.

2. How do you perceive the role of AI and AI-powered machines in bridging communication gaps between people from different cultures?

It enhance globalisation concepts,

CamScanner

No (05) & (3)
No (05)

3. Have you experienced and observed any instances where AI has been successful in facilitating cross-cultural understanding and collaboration in mediated communication?

Yes, in the field of robotics surgery including the expertise of pioneer surgeon, in related to knee surgery procedure.

4. In what ways do you think AI can help overcome language barriers and promote more inclusive communication among diverse groups?

AI is a tool, hence the language settings is dependent to the user of origin, hence the non-language barrier is adverse independent. <Selection criteria>.

5. Are there any cultural biases or stereotypes that you have noticed in AI-powered language tools or communication platforms?

Bias — information
— people / user of origin
— practical <tools / domain>

6. How can universities and educational institutions leverage AI to create more inclusive and culturally diverse online learning environments?

Standardized the concept, and be legitimate to user friendly.

Thank you for your time and participation

CS CamScanner

Participant No (~~48~~) & PO
(06)

UNIVERSITI
MALAYA

Depart of Media and Communication Studies

AI and Mediated Intercultural Communication Questionnaire

Dear UM Student/ Researcher,

Through this survey, we want to assess the probable effects of Artificial Intelligence (AI) on mediated communication among people from different cultures. This instrument which been developed by the help of the existing literature and ChatGPT, has 15 structured items with five (5) options per item and six (6) open-ended questions. Your participation in the survey is voluntary and the personal identifications of the participants will be treated as strictly confidential.

Thank you for your cooperation.

The researchers

A. Demographic Information

1. Gender: ☑ Male ☐ Female
2. Age: 38
3. Nationality: Pakistani
4. Level of education: PhD student
5. AI/ ChatGPT experience: ☑ Yes ☐ No

B. Intercultural Sensitivity Scale

Directions: The current AI-ICC questionnaire includes 15 items/ statements with five (5) options per item, as: 1 – Strongly agree; 2 – Agree, 3 – Uncertain; 4 – Disagree, and 5 – Strongly disagree.	Strongly agree	Agree	Uncertain	Disagree	Strongly disagree
1. AI and AI-powered machines can be helpful in bridging communication gaps between people from different cultures.	1	2	3	4 ✓	5
2. So far, AI has been successful in facilitating cross-cultural understanding and collaboration in mediated communication.	1	2	3 ✓	4	5
3. AI can help overcome language barriers and promote more inclusive communication among diverse groups.	1	2	3 ✓	4	5

CS CamScanner

No 48 & P2 06

4. There some cultural biases and stereotypes in AI-powered language tools and communication platforms.	1	2	3 ✓	4	5
5. AI has affected the localization and adaptation of content for different cultural audiences in digital communication.	1	2 ✓	3	4	5
6. I encountered any challenges in using AI-powered translation tools when communicating with people from different cultural backgrounds.	1	2	3	4	5 ✓
7. AI has the potential to promote cultural exchange and appreciation by enabling easier access to diverse perspectives and information.	1 ✓	2	3	4	5
8. AI-powered machines and chatbots should be programmed to respect and adapt to cultural norms and communication styles while interacting with users from different cultures.	1 ✓	2	3	4	5
9. There are some potential risks and concerns regarding AI's role in cross-cultural communication, such as perpetuating cultural stereotypes and misinterpretations.	1 ✓	2	3	4	5
10. I have participated in some cross-cultural virtual collaborations that relied on AI for communication and coordination.	1	2	3	4	5 ✓
11. I think AI can enhance intercultural learning experiences and foster empathy among individuals from different cultural backgrounds.	1	2 ✓	3	4	5
12. AI-powered virtual reality (VR) and augmented reality (AR) technologies can impact cross-cultural communication and understanding.	1	2	3 ✓	4	5
13. Somehow AI-generated content may unintentionally offend and misled individuals from specific cultural backgrounds.	1	2	3 ✓	4	5
14. Ethical considerations should be taken into account when designing AI-powered communication tools that cater to diverse cultural contexts.	1 ✓	2	3	4	5
15. Universities and educational institutions leverage AI to create more inclusive and culturally diverse online learning environments.	1 ✓	2	3	4	5

In the case of your agreement on sharing your answer/ points of views for questions below, please write down your comment/ point of view for all questions or any question that you prefer:

1. Did you experience the use of AI and AI-powered chatbots, such as ChatGPT and for what purpose?

   I mostly used ChatGPT for searching information regarding my study.

2. How do you perceive the role of AI and AI-powered machines in bridging communication gaps between people from different cultures?

   I am not sure.

CS CamScanner

No ~~48~~ & P3
(06)

3. Have you experienced and observed any instances where AI has been successful in facilitating cross-cultural understanding and collaboration in mediated communication?

No.

4. In what ways do you think AI can help overcome language barriers and promote more inclusive communication among diverse groups?

If the AI developers can also ~~to~~ add the context of ~~certain~~ in the languages. As any word/sentence can have different meanings.

5. Are there any cultural biases or stereotypes that you have noticed in AI-powered language tools or communication platforms?

Not yet.

6. How can universities and educational institutions leverage AI to create more inclusive and culturally diverse online learning environments?

I think it would be good to include/study the different languages/contexts.

Thank you for your time and participation

CS CamScanner

Participant No (37) & PO (07)

UNIVERSITI MALAYA

Depart of Media and Communication Studies

AI and Mediated Intercultural Communication Questionnaire

Dear UM Student/ Researcher,

Through this survey, we want to assess the probable effects of Artificial Intelligence (AI) on mediated communication among people from different cultures. This instrument which been developed by the help of the existing literature and ChatGPT, has 15 structured items with five (5) options per item and six (6) open-ended questions. Your participation in the survey is voluntary and the personal identifications of the participants will be treated as strictly confidential.

Thank you for your cooperation.

The researchers

A. Demographic Information

1. Gender: ☑ Male ☐ Female
2. Age: 25
3. Nationality: China
4. Level of education: Phd.
5. AI/ ChatGPT experience: ☑ Yes ☐ No

B. Intercultural Sensitivity Scale

Directions: The current AI-ICC questionnaire includes 15 items/ statements with five (5) options per item, as: 1 – Strongly agree; 2 – Agree, 3 – Uncertain; 4 – Disagree, and 5 – Strongly disagree.	Strongly agree	Agree	Uncertain	Disagree	Strongly disagree
1. AI and AI-powered machines can be helpful in bridging communication gaps between people from different cultures.	1 ✓	2	3	4	5
2. So far, AI has been successful in facilitating cross-cultural understanding and collaboration in mediated communication.	1	2	3 ✓	4	5
3. AI can help overcome language barriers and promote more inclusive communication among diverse groups.	1	2 ✓	3	4	5

CamScanner

No ~~01~~ 07 & P2

4. There some cultural biases and stereotypes in AI-powered language tools and communication platforms.	1 ✓	2	3	4	5
5. AI has affected the localization and adaptation of content for different cultural audiences in digital communication.	1	2 ✓	3	4	5
6. I encountered any challenges in using AI-powered translation tools when communicating with people from different cultural backgrounds.	1 ✓	2	3	4	5
7. AI has the potential to promote cultural exchange and appreciation by enabling easier access to diverse perspectives and information.	1 ✓	2	3	4	5
8. AI-powered machines and chatbots should be programmed to respect and adapt to cultural norms and communication styles while interacting with users from different cultures.	1	2 ✓	3	4	5
9. There are some potential risks and concerns regarding AI's role in cross-cultural communication, such as perpetuating cultural stereotypes and misinterpretations.	1	2 ✓	3	4	5
10. I have participated in some cross-cultural virtual collaborations that relied on AI for communication and coordination.	1	2 ✓	3	4	5
11. I think AI can enhance intercultural learning experiences and foster empathy among individuals from different cultural backgrounds.	1 ✓	2	3	4	5
12. AI-powered virtual reality (VR) and augmented reality (AR) technologies can impact cross-cultural communication and understanding.	1 ✓	2	3	4	5
13. Somehow AI-generated content may unintentionally offend and misled individuals from specific cultural backgrounds.	1 ✓	2	3	4	5
14. Ethical considerations should be taken into account when designing AI-powered communication tools that cater to diverse cultural contexts.	1	2 ✓	3	4	5
15. Universities and educational institutions leverage AI to create more inclusive and culturally diverse online learning environments.	1 ✓	2	3	4	5

In the case of your agreement on sharing your answer/ points of views for questions below, please write down your comment/ point of view for all questions or any question that you prefer:

1. Did you experience the use of AI and AI-powered chatbots, such as ChatGPT and for what purpose?

Yes, I have. use it to acquire knowledge.

2. How do you perceive the role of AI and AI-powered machines in bridging communication gaps between people from different cultures?

People from different countries can use it to translate and communicate easily.

CamScanner

No (87) & P (3)
(67)

3. Have you experienced and observed any instances where AI has been successful in facilitating cross-cultural understanding and collaboration in mediated communication?

Sorry, I am not experience in this.

4. In what ways do you think AI can help overcome language barriers and promote more inclusive communication among diverse groups?

AI can help me translate other language to communicate other people in some international meeting.

5. Are there any cultural biases or stereotypes that you have noticed in AI-powered language tools or communication platforms?

Sorry, I don't kown this.

6. How can universities and educational institutions leverage AI to create more inclusive and culturally diverse online learning environments?

University can create a knowledge platform to help student acquire knowledge.

Thank you for your time and participation

CS CamScanner

Partcipant No ~~8~~ & P① (08)

UNIVERSITI MALAYA

Depart of Media and Communication Studies

AI and Mediated Intercultural Communication Questionnaire

Dear UM Student/ Researcher,

Through this survey, we want to assess the probable effects of Artificial Intelligence (AI) on mediated communication among people from different cultures. This instrument which been developed by the help of the existing literature and ChatGPT, has 15 structured items with five (5) options per item and six (6) open-ended questions. Your participation in the survey is voluntary and the personal identifications of the participants will be treated as strictly confidential.

Thank you for your cooperation.

The researchers

A. Demographic Information

1. Gender: ☑ Male ☐ Female

2. Age: 30

3. Nationality: Malaysian

4. Level of education: Masters

5. AI/ ChatGPT experience: ☐ Yes ☑ No

B. Intercultural Sensitivity Scale

Directions: The current AI-ICC questionnaire includes 15 items/ statements with five (5) options per item, as: 1 – Strongly agree; 2 – Agree, 3 – Uncertain; 4 – Disagree, and 5 – Strongly disagree.	Strongly agree	Agree	Uncertain	Disagree	Strongly disagree
1. AI and AI-powered machines can be helpful in bridging communication gaps between people from different cultures.	1	(2)	3	4	5
2. So far, AI has been successful in facilitating cross-cultural understanding and collaboration in mediated communication.	1	2	(3)	4	5
3. AI can help overcome language barriers and promote more inclusive communication among diverse groups.	1	(2)	3	4	5

CamScanner

No [illegible] 9 P 2
08

4. There some cultural biases and stereotypes in AI-powered language tools and communication platforms.	1	(2)	3	4	5
5. AI has affected the localization and adaptation of content for different cultural audiences in digital communication.	1	2	(3)	4	5
6. I encountered any challenges in using AI-powered translation tools when communicating with people from different cultural backgrounds.	1	2	(3)	4	5
7. AI has the potential to promote cultural exchange and appreciation by enabling easier access to diverse perspectives and information.	1	(2)	3	4	5
8. AI-powered machines and chatbots should be programmed to respect and adapt to cultural norms and communication styles while interacting with users from different cultures.	(1)	2	3	4	5
9. There are some potential risks and concerns regarding AI's role in cross-cultural communication, such as perpetuating cultural stereotypes and misinterpretations.	1	(2)	3	4	5
10. I have participated in some cross-cultural virtual collaborations that relied on AI for communication and coordination.	1	2	3	(4)	5
11. I think AI can enhance intercultural learning experiences and foster empathy among individuals from different cultural backgrounds.	1	(2)	3	4	5
12. AI-powered virtual reality (VR) and augmented reality (AR) technologies can impact cross-cultural communication and understanding.	1	(2)	3	4	5
13. Somehow AI-generated content may unintentionally offend and misled individuals from specific cultural backgrounds.	1	(2)	3	4	5
14. Ethical considerations should be taken into account when designing AI-powered communication tools that cater to diverse cultural contexts.	(1)	2	3	4	5
15. Universities and educational institutions leverage AI to create more inclusive and culturally diverse online learning environments.	1	(2)	3	4	5

In the case of your agreement on sharing your answer/ points of views for questions below, please write down your comment/ point of view for all questions or any question that you prefer:

1. Did you experience the use of AI and AI-powered chatbots, such as ChatGPT and for what purpose?

   I have not used AI / chatbots before

2. How do you perceive the role of AI and AI-powered machines in bridging communication gaps between people from different cultures?

   Yes

CS CamScanner

No ~~[illegible]~~ (08) & P(3)

3. Have you experienced and observed any instances where AI has been successful in facilitating cross-cultural understanding and collaboration in mediated communication?

Not yet.

4. In what ways do you think AI can help overcome language barriers and promote more inclusive communication among diverse groups?

It has the potential to aid translations and cross-cultural communication

5. Are there any cultural biases or stereotypes that you have noticed in AI-powered language tools or communication platforms?

Grammar and specific language rules are difficult to get correct only by AI. I observed this when a teacher checked an exam answer for Japanese. done by AI.

6. How can universities and educational institutions leverage AI to create more inclusive and culturally diverse online learning environments?

~~A~~ Not sure yet.

Thank you for your time and participation

CamScanner

Participant No [illegible] & P(1)
(09)

UNIVERSITI MALAYA

Depart of Media and Communication Studies

AI and Mediated Intercultural Communication Questionnaire

Dear UM Student/ Researcher,

Through this survey, we want to assess the probable effects of Artificial Intelligence (AI) on mediated communication among people from different cultures. This instrument which been developed by the help of the existing literature and ChatGPT, has 15 structured items with five (5) options per item and six (6) open-ended questions. Your participation in the survey is voluntary and the personal identifications of the participants will be treated as strictly confidential.

Thank you for your cooperation.

The researchers

A. Demographic Information

1. Gender: ☐ Male ☑ Female

2. Age: 35

3. Nationality: Indonesia

4. Level of education: Ph.D.

5. AI/ ChatGPT experience: ☑ Yes ☐ No

B. Intercultural Sensitivity Scale

Directions: The current AI-ICC questionnaire includes 15 items/ statements with five (5) options per item, as: 1 – Strongly agree; 2 – Agree, 3 – Uncertain; 4 – Disagree, and 5 – Strongly disagree.	Strongly agree	Agree	Uncertain	Disagree	Strongly disagree
1. AI and AI-powered machines can be helpful in bridging communication gaps between people from different cultures.	1	(2)	3	4	5
2. So far, AI has been successful in facilitating cross-cultural understanding and collaboration in mediated communication.	1	2	(3)	4	5
3. AI can help overcome language barriers and promote more inclusive communication among diverse groups.	1	2	3	(4)	5

CamScanner

No [illegible] (09) & P(2)

4. There some cultural biases and stereotypes in AI-powered language tools and communication platforms.	1	2	(3)	4	5
5. AI has affected the localization and adaptation of content for different cultural audiences in digital communication.	1	(2)	3	4	5
6. I encountered any challenges in using AI-powered translation tools when communicating with people from different cultural backgrounds.	1	2	3	(4)	5
7. AI has the potential to promote cultural exchange and appreciation by enabling easier access to diverse perspectives and information.	1	(2)	3	4	5
8. AI-powered machines and chatbots should be programmed to respect and adapt to cultural norms and communication styles while interacting with users from different cultures.	1	(2)	3	4	5
9. There are some potential risks and concerns regarding AI's role in cross-cultural communication, such as perpetuating cultural stereotypes and misinterpretations.	1	2	(3)	4	5
10. I have participated in some cross-cultural virtual collaborations that relied on AI for communication and coordination.	1	2	3	(4)	5
11. I think AI can enhance intercultural learning experiences and foster empathy among individuals from different cultural backgrounds.	1	(2)	3	4	5
12. AI-powered virtual reality (VR) and augmented reality (AR) technologies can impact cross-cultural communication and understanding.	1	(2)	3	4	5
13. Somehow AI-generated content may unintentionally offend and misled individuals from specific cultural backgrounds.	1	2	(3)	4	5
14. Ethical considerations should be taken into account when designing AI-powered communication tools that cater to diverse cultural contexts.	1	(2)	3	4	5
15. Universities and educational institutions leverage AI to create more inclusive and culturally diverse online learning environments.	1	(2)	3	4	5

In the case of your agreement on sharing your answer/ points of views for questions below, please write down your comment/ point of view for all questions or any question that you prefer:

1. Did you experience the use of AI and AI-powered chatbots, such as ChatGPT and for what purpose?

   Yes, I used AI for educational purposes

2. How do you perceive the role of AI and AI-powered machines in bridging communication gaps between people from different cultures?

   Not sure about it

CS CamScanner

No (39) E, P(3)
(09)

3. Have you experienced and observed any instances where AI has been successful in facilitating cross-cultural understanding and collaboration in mediated communication?

No, I haven't

4. In what ways do you think AI can help overcome language barriers and promote more inclusive communication among diverse groups?

By providing translation a features that ~~can~~ could erase the language barrier.

5. Are there any cultural biases or stereotypes that you have noticed in AI-powered language tools or communication platforms?

Not sure

6. How can universities and educational institutions leverage AI to create more inclusive and culturally diverse online learning environments?

By increasing awarness of the students about AI without neglecting the ethics

Thank you for your time and participation

CS CamScanner

Participant No (~~12~~) & PO (1)
(10)

UNIVERSITI
MALAYA

Depart of Media and Communication Studies

AI and Mediated Intercultural Communication Questionnaire

Dear UM Student/ Researcher,

Through this survey, we want to assess the probable effects of Artificial Intelligence (AI) on mediated communication among people from different cultures. This instrument which been developed by the help of the existing literature and ChatGPT, has 15 structured items with five (5) options per item and six (6) open-ended questions. Your participation in the survey is voluntary and the personal identifications of the participants will be treated as strictly confidential.

Thank you for your cooperation.

The researchers

A. Demographic Information

1. Gender: ☐ Male ☑ Female

2. Age: 31

3. Nationality: Indonesia

4. Level of education: Master

5. AI/ ChatGPT experience: ☑ Yes ☐ No

B. Intercultural Sensitivity Scale

Directions: The current AI-ICC questionnaire includes 15 items/ statements with five (5) options per item, as: 1 – Strongly agree; 2 – Agree, 3 – Uncertain; 4 – Disagree, and 5 – Strongly disagree.	Strongly agree	Agree	Uncertain	Disagree	Strongly disagree
1. AI and AI-powered machines can be helpful in bridging communication gaps between people from different cultures.	(1)	2	3	4	5
2. So far, AI has been successful in facilitating cross-cultural understanding and collaboration in mediated communication.	1	(2)	3	4	5
3. AI can help overcome language barriers and promote more inclusive communication among diverse groups.	1	(2)	3	4	5

CamScanner

No (10) & P2

4. There some cultural biases and stereotypes in AI-powered language tools and communication platforms.	1	(2)	3	4	5
5. AI has affected the localization and adaptation of content for different cultural audiences in digital communication.	1	(2)	3	4	5
6. I encountered any challenges in using AI-powered translation tools when communicating with people from different cultural backgrounds.	1	2	3	(4)	5
7. AI has the potential to promote cultural exchange and appreciation by enabling easier access to diverse perspectives and information.	1	(2)	3	4	5
8. AI-powered machines and chatbots should be programmed to respect and adapt to cultural norms and communication styles while interacting with users from different cultures.	(1)	2	3	4	5
9. There are some potential risks and concerns regarding AI's role in cross-cultural communication, such as perpetuating cultural stereotypes and misinterpretations.	(1)	2	3	4	5
10. I have participated in some cross-cultural virtual collaborations that relied on AI for communication and coordination.	1	2	3	(4)	5
11. I think AI can enhance intercultural learning experiences and foster empathy among individuals from different cultural backgrounds.	1	(2)	3	4	5
12. AI-powered virtual reality (VR) and augmented reality (AR) technologies can impact cross-cultural communication and understanding.	(1)	2	3	4	5
13. Somehow AI-generated content may unintentionally offend and misled individuals from specific cultural backgrounds.	(1)	2	3	4	5
14. Ethical considerations should be taken into account when designing AI-powered communication tools that cater to diverse cultural contexts.	(1)	2	3	4	5
15. Universities and educational institutions leverage AI to create more inclusive and culturally diverse online learning environments.	(1)	2	3	4	5

In the case of your agreement on sharing your answer/ points of views for questions below, please write down your comment/ point of view for all questions or any question that you prefer:

1. Did you experience the use of AI and AI-powered chatbots, such as ChatGPT and for what purpose?
   Yes, to help me writing my ideas in more academic writing

2. How do you perceive the role of AI and AI-powered machines in bridging communication gaps between people from different cultures?
   It surely can help people to write and explain their ideas in more specific purpose of communication

CamScanner

No [illegible] (10) E P3

3. Have you experienced and observed any instances where AI has been successful in facilitating cross-cultural understanding and collaboration in mediated communication?

not yet

4. In what ways do you think AI can help overcome language barriers and promote more inclusive communication among diverse groups?

by helping people communicate for specific purpose, like AI ability to know what purpose of the paraphrase or translation

5. Are there any cultural biases or stereotypes that you have noticed in AI-powered language tools or communication platforms?

so far not encounter any

6. How can universities and educational institutions leverage AI to create more inclusive and culturally diverse online learning environments?

by using AI on their online interactive learning

Thank you for your time and participation

CamScanner

Participant No (11) & Page (1)

UNIVERSITI MALAYA

Depart of Media and Communication Studies

AI and Mediated Intercultural Communication Questionnaire

Dear UM Student/ Researcher,

Through this survey, we want to assess the probable effects of Artificial Intelligence (AI) on mediated communication among people from different cultures. This instrument which been developed by the help of the existing literature and ChatGPT, has 15 structured items with five (5) options per item and six (6) open-ended questions. Your participation in the survey is voluntary and the personal identifications of the participants will be treated as strictly confidential.

Thank you for your cooperation.

The researchers

A. Demographic Information

1. Gender: ☐ Male ☑ Female

2. Age: 24

3. Nationality: China

4. Level of education: PhD candidate

5. AI/ ChatGPT experience: ☑ Yes ☐ No

B. Intercultural Sensitivity Scale

**Directions:** The current AI-ICC questionnaire includes 15 items/ statements with five (5) options per item, as: 1 – Strongly agree; 2 – Agree, 3 – Uncertain; 4 – Disagree, and 5 – Strongly disagree.	Strongly agree	Agree	Uncertain	Disagree	Strongly disagree
1. AI and AI-powered machines can be helpful in bridging communication gaps between people from different cultures.	1	2 ✓	3	4	5
2. So far, AI has been successful in facilitating cross-cultural understanding and collaboration in mediated communication.	1	2 ✓	3	4	5
3. AI can help overcome language barriers and promote more inclusive communication among diverse groups.	1	2 ✓	3	4	5

CamScanner

Participant no. (1) & P2

4. There some cultural biases and stereotypes in AI-powered language tools and communication platforms.	1	2	3 ✓	4	5
5. AI has affected the localization and adaptation of content for different cultural audiences in digital communication.	1	2 ✓	3	4	5
6. I encountered any challenges in using AI-powered translation tools when communicating with people from different cultural backgrounds.	1	2	3	4 ✓	5
7. AI has the potential to promote cultural exchange and appreciation by enabling easier access to diverse perspectives and information.	1 ✓	2	3	4	5
8. AI-powered machines and chatbots should be programmed to respect and adapt to cultural norms and communication styles while interacting with users from different cultures.	1 ✓	2	3	4	5
9. There are some potential risks and concerns regarding AI's role in cross-cultural communication, such as perpetuating cultural stereotypes and misinterpretations.	1	2 ✓	3	4	5
10. I have participated in some cross-cultural virtual collaborations that relied on AI for communication and coordination.	1	2	3 ✓	4	5
11. I think AI can enhance intercultural learning experiences and foster empathy among individuals from different cultural backgrounds.	1 ✓	2	3	4	5
12. AI-powered virtual reality (VR) and augmented reality (AR) technologies can impact cross-cultural communication and understanding.	1	2 ✓	3	4	5
13. Somehow AI-generated content may unintentionally offend and misled individuals from specific cultural backgrounds.	1	2	3	4 ✓	5
14. Ethical considerations should be taken into account when designing AI-powered communication tools that cater to diverse cultural contexts.	1 ✓	2	3	4	5
15. Universities and educational institutions leverage AI to create more inclusive and culturally diverse online learning environments.	1 ✓	2	3	4	5

In the case of your agreement on sharing your answer/ points of views for questions below, please write down your comment/ point of view for all questions or any question that you prefer:

1. Did you experience the use of AI and AI-powered chatbots, such as ChatGPT and for what purpose?

Yes. chatGPT is like a Wikipedia for me now. no matter study and life. I use it for searching everything. For study, I use it for searching Literature. definition, etc. For d[illegible] life, I use it for figuring out every question.

2. How do you perceive the role of AI and AI-powered machines in bridging communication gaps between people from different cultures?

it's a key to open the door of me and foreigners. I can use AI to translate any language that I don't know. So that I can communicate with th[illegible] [illegible]wever, sometimes the translation can't directly transmit my meaning. even transl[illegible] a wrong way or inaccurate words. I will say I can't trust it 100%. but for daily communication. it's fine.

CamScanner

3. Have you experienced and observed any instances where AI has been successful in facilitating cross-cultural understanding and collaboration in mediated communication?

Yes. My bf is work with foreiners. he needs to talk with every races. so he usually use AI to translate and text with other ppl.

4. In what ways do you think AI can help overcome language barriers and promote more inclusive communication among diverse groups?

for daily conversation. it can transmit your idea to other ppl.

5. Are there any cultural biases or stereotypes that you have noticed in AI-powered language tools or communication platforms?

Yes. when AI translate some chinese words to English. it translated wrong words. such as "Ying xiang" in Chinese means impact, it's a noun. but English translation usually translate in verb or n-ing, it's actually changed the meaning.

6. How can universities and educational institutions leverage AI to create more inclusive and culturally diverse online learning environments?

Maybe understand different cultural background first.

make ~~sure~~

Thank you for your time and participation

CS CamScanner

Participant (12) & Page (1)

UNIVERSITI MALAYA

Depart of Media and Communication Studies

AI and Mediated Intercultural Communication Questionnaire

Dear UM Student/ Researcher,

Through this survey, we want to assess the probable effects of Artificial Intelligence (AI) on mediated communication among people from different cultures. This instrument which been developed by the help of the existing literature and ChatGPT, has 15 structured items with five (5) options per item and six (6) open-ended questions. Your participation in the survey is voluntary and the personal identifications of the participants will be treated as strictly confidential.

Thank you for your cooperation.

The researchers

A. Demographic Information

1. Gender: ☐ Male ☑ Female

2. Age: 34

3. Nationality: China

4. Level of education: PhD student

5. AI/ ChatGPT experience: ☑ Yes ☐ No

B. Intercultural Sensitivity Scale

Directions: The current AI-ICC questionnaire includes 15 items/ statements with five (5) options per item, as: 1 – Strongly agree; 2 – Agree, 3 – Uncertain; 4 – Disagree, and 5 – Strongly disagree.	Strongly agree	Agree	Uncertain	Disagree	Strongly disagree
1. AI and AI-powered machines can be helpful in bridging communication gaps between people from different cultures.	(1)	2	3	4	5
2. So far, AI has been successful in facilitating cross-cultural understanding and collaboration in mediated communication.	1	(2)	3	4	5
3. AI can help overcome language barriers and promote more inclusive communication among diverse groups.	1	(2)	3	4	5

CamScanner

P12 Pa & I Page 2

4. There some cultural biases and stereotypes in AI-powered language tools and communication platforms.	1	2	**3**	4	5
5. AI has affected the localization and adaptation of content for different cultural audiences in digital communication.	1	2	3	**4**	5
6. I encountered any challenges in using AI-powered translation tools when communicating with people from different cultural backgrounds.	1	2	3	**4**	5
7. AI has the potential to promote cultural exchange and appreciation by enabling easier access to diverse perspectives and information.	**1**	2	3	4	5
8. AI-powered machines and chatbots should be programmed to respect and adapt to cultural norms and communication styles while interacting with users from different cultures.	**1**	2	3	4	5
9. There are some potential risks and concerns regarding AI's role in cross-cultural communication, such as perpetuating cultural stereotypes and misinterpretations.	1 [illegible]	**2**	3	4	5
10. I have participated in some cross-cultural virtual collaborations that relied on AI for communication and coordination.	1	2	3	**4**	5
11. I think AI can enhance intercultural learning experiences and foster empathy among individuals from different cultural backgrounds.	1	**2**	3	4	5
12. AI-powered virtual reality (VR) and augmented reality (AR) technologies can impact cross-cultural communication and understanding.	1	**2**	3	4	5
13. Somehow AI-generated content may unintentionally offend and misled individuals from specific cultural backgrounds.	1	**2**	3	4	5
14. Ethical considerations should be taken into account when designing AI-powered communication tools that cater to diverse cultural contexts.	**1**	2	3	4	5
15. Universities and educational institutions leverage AI to create more inclusive and culturally diverse online learning environments.	**1**	2	3	4	5

In the case of your agreement on sharing your answer/ points of views for questions below, please write down your comment/ point of view for all questions or any question that you prefer:

1. Did you experience the use of AI and AI-powered chatbots, such as ChatGPT and for what purpose?

I ever used Chat GPT for helping me searching for literature in my research.

2. How do you perceive the role of AI and AI-powered machines in bridging communication gaps between people from different cultures?

AI-powered machines need to be designed more localized and involve ~~more~~ wide-range of up-to-date info (not only internet info but also authentic occurrence) in order to narrowing down the information gap and cultural gaps between cultures.

CamScanner

P12 — Page 3
P12

3. Have you experienced and observed any instances where AI has been successful in facilitating cross-cultural understanding and collaboration in mediated communication?

For many ~~interf~~ international conferences held in mainland China, human interpreters have been less employed since AI instant translation tools are so powerful and with high efficiency and accuracy. That's AI's function in breaking the communication barriers in such contexts.

4. In what ways do you think AI can help overcome language barriers and promote more inclusive communication among diverse groups?

Please see above.

5. Are there any cultural biases or stereotypes that you have noticed in AI-powered language tools or communication platforms?

Yes, I did noticed cultural stereotypes in AI-powered tools and I believe it is hard to alleviate or eliminate ~~of~~ cultural biases and stereotypes because each technology actually indicates the will of the state. from my personal understanding.

6. How can universities and educational institutions leverage AI to create more inclusive and culturally diverse online learning environments?

Maybe could employee AI in some side research area not in the key areas for the sake of ethical concern and security concern.

Thank you for your time and participation

CamScanner

Participant (13) & Page (1)
(13)

UNIVERSITI MALAYA

Depart of Media and Communication Studies

AI and Mediated Intercultural Communication Questionnaire

Dear UM Student/ Researcher,

Through this survey, we want to assess the probable effects of Artificial Intelligence (AI) on mediated communication among people from different cultures. This instrument which been developed by the help of the existing literature and ChatGPT, has 15 structured items with five (5) options per item and six (6) open-ended questions. Your participation in the survey is voluntary and the personal identifications of the participants will be treated as strictly confidential.

Thank you for your cooperation.

The researchers

A. Demographic Information

1. Gender: ☐ Male ☑ Female

2. Age: 26

3. Nationality: Malaysian

4. Level of education: PhD

5. AI/ ChatGPT experience: ☑ Yes ☐ No

B. Intercultural Sensitivity Scale

**Directions:** The current AI-ICC questionnaire includes 15 items/ statements with five (5) options per item, as: 1 – Strongly agree; 2 – Agree, 3 – Uncertain; 4 – Disagree, and 5 – Strongly disagree.	Strongly agree	Agree	Uncertain	Disagree	Strongly disagree
1. AI and AI-powered machines can be helpful in bridging communication gaps between people from different cultures.	(1)	2	3	4	5
2. So far, AI has been successful in facilitating cross-cultural understanding and collaboration in mediated communication.	(1)	2	3	4	5
3. AI can help overcome language barriers and promote more inclusive communication among diverse groups.	1 ✓	2	3	4	5

CS CamScanner

Participant No (13) & Page (2)

4. There some cultural biases and stereotypes in AI-powered language tools and communication platforms.	1	2	3	4	(5)
5. AI has affected the localization and adaptation of content for different cultural audiences in digital communication.	1	2	3	4	(5)
6. I encountered any challenges in using AI-powered translation tools when communicating with people from different cultural backgrounds.	1	2	(3)	4	5
7. AI has the potential to promote cultural exchange and appreciation by enabling easier access to diverse perspectives and information.	(1)	2	3	4	5
8. AI-powered machines and chatbots should be programmed to respect and adapt to cultural norms and communication styles while interacting with users from different cultures.	(1)	2	3	4	5
9. There are some potential risks and concerns regarding AI's role in cross-cultural communication, such as perpetuating cultural stereotypes and misinterpretations.	1	2	3	(4)	5
10. I have participated in some cross-cultural virtual collaborations that relied on AI for communication and coordination.	1	2	3	4	(5)
11. I think AI can enhance intercultural learning experiences and foster empathy among individuals from different cultural backgrounds.	(1)	2	3	4	5
12. AI-powered virtual reality (VR) and augmented reality (AR) technologies can impact cross-cultural communication and understanding.	(1)	2	3	4	5
13. Somehow AI-generated content may unintentionally offend and misled individuals from specific cultural backgrounds.	1	2	(3)	4	5
14. Ethical considerations should be taken into account when designing AI-powered communication tools that cater to diverse cultural contexts.	(1)	2	3	4	5
15. Universities and educational institutions leverage AI to create more inclusive and culturally diverse online learning environments.	(1)	2	3	4	5

In the case of your agreement on sharing your answer/ points of views for questions below, please write down your comment/ point of view for all questions or any question that you prefer:

1. Did you experience the use of AI and AI-powered chatbots, such as ChatGPT and for what purpose?

Yes. I used AI-powered chatbot such as AirAsia app, UM library website TNG eWallet app and ChatGPT. I used them for the need of technical support for the first 3 and for ChatGPT I used it for improve my English

2. How do you perceive the role of AI and AI-powered machines in bridging communication gaps between people from different cultures?

I believe people from different cultures do not face any problem using AI and AI-powered machines, and it definitely could help people from different cultures to understand each other more.

CS CamScanner

Partcipant (13) & Page (3)
(13)

3. Have you experienced and observed any instances where AI has been successful in facilitating cross-cultural understanding and collaboration in mediated communication?

Yes, I have been using AI auto-translate by picture when I ~~hav~~ was in a ~~cou~~ country that is not in my language.

4. In what ways do you think AI can help overcome language barriers and promote more inclusive communication among diverse groups?

AI did help overcome language barriers. For eg., we used body language to communicate when AI was not invented. But now with AI, people can easily communicate using AI-translate.

5. Are there any cultural biases or stereotypes that you have noticed in AI-powered language tools or communication platforms?

No.

6. How can universities and educational institutions leverage AI to create more inclusive and culturally diverse online learning environments?

I believe using AI can create more interactions between users with no language gaps through instant AI-translations. Users can easily make friends with each other.

Thank you for your time and participation

CS CamScanner

Partcipant No (14) & P(1)

UNIVERSITI MALAYA

Depart of Media and Communication Studies

AI and Mediated Intercultural Communication Questionnaire

Dear UM Student/ Researcher,

Through this survey, we want to assess the probable effects of Artificial Intelligence (AI) on mediated communication among people from different cultures. This instrument which been developed by the help of the existing literature and ChatGPT, has 15 structured items with five (5) options per item and six (6) open-ended questions. Your participation in the survey is voluntary and the personal identifications of the participants will be treated as strictly confidential.

Thank you for your cooperation.

The researchers

A. Demographic Information

1. Gender: ☐ Male ☑ Female

2. Age: 35

3. Nationality: China

4. Level of education: PhD

5. AI/ ChatGPT experience: ☑ Yes ☐ No

B. Intercultural Sensitivity Scale

Directions: The current AI-ICC questionnaire includes 15 items/ statements with five (5) options per item, as: 1 – Strongly agree; 2 – Agree, 3 – Uncertain; 4 – Disagree, and 5 – Strongly disagree.	Strongly agree	Agree	Uncertain	Disagree	Strongly disagree
1. AI and AI-powered machines can be helpful in bridging communication gaps between people from different cultures.	1 ✓	2	3	4	5
2. So far, AI has been successful in facilitating cross-cultural understanding and collaboration in mediated communication.	1 ✓	2	3	4	5
3. AI can help overcome language barriers and promote more inclusive communication among diverse groups.	1 ✓	2	3	4	5

CamScanner

No (14) 乙 P2

4. There some cultural biases and stereotypes in AI-powered language tools and communication platforms.	1	2 ✓	3	4	5
5. AI has affected the localization and adaptation of content for different cultural audiences in digital communication.	1	2 ✓	3	4	5
6. I encountered any challenges in using AI-powered translation tools when communicating with people from different cultural backgrounds.	1	2 ✓	3	4	5
7. AI has the potential to promote cultural exchange and appreciation by enabling easier access to diverse perspectives and information.	1 ✓	2	3	4	5
8. AI-powered machines and chatbots should be programmed to respect and adapt to cultural norms and communication styles while interacting with users from different cultures.	1 ✓	2	3	4	5
9. There are some potential risks and concerns regarding AI's role in cross-cultural communication, such as perpetuating cultural stereotypes and misinterpretations.	1	2 ✓	3	4	5
10. I have participated in some cross-cultural virtual collaborations that relied on AI for communication and coordination.	1	2	3 ✓	4	5
11. I think AI can enhance intercultural learning experiences and foster empathy among individuals from different cultural backgrounds.	1 ✓	2	3	4	5
12. AI-powered virtual reality (VR) and augmented reality (AR) technologies can impact cross-cultural communication and understanding.	1 ✓	2	3	4	5
13. Somehow AI-generated content may unintentionally offend and misled individuals from specific cultural backgrounds.	1	2 ✓	3	4	5
14. Ethical considerations should be taken into account when designing AI-powered communication tools that cater to diverse cultural contexts.	1 ✓	2	3	4	5
15. Universities and educational institutions leverage AI to create more inclusive and culturally diverse online learning environments.	1 ✓	2	3	4	5

In the case of your agreement on sharing your answer/ points of views for questions below, please write down your comment/ point of view for all questions or any question that you prefer:

1. Did you experience the use of AI and AI-powered chatbots, such as ChatGPT and for what purpose?

Yes.

For study, searching for literature and facilitate learning.

2. How do you perceive the role of AI and AI-powered machines in bridging communication gaps between people from different cultures?

Helpful.

CS CamScanner

No (14) & P3

3. Have you experienced and observed any instances where AI has been successful in facilitating cross-cultural understanding and collaboration in mediated communication?

Not yet.

4. In what ways do you think AI can help overcome language barriers and promote more inclusive communication among diverse groups?

Give the exact and real examples of language barriers between different cultures.

5. Are there any cultural biases or stereotypes that you have noticed in AI-powered language tools or communication platforms?

No.

6. How can universities and educational institutions leverage AI to create more inclusive and culturally diverse online learning environments?

Giving lectures on how to use AI to aid learning. Encourage group dissaussion and group project to find more efficient way to navigate AI in learning.

Thank you for your time and participation

CamScanner

Participant No (15) & P(1)

UNIVERSITI MALAYA

Depart of Media and Communication Studies

AI and Mediated Intercultural Communication Questionnaire

Dear UM Student/ Researcher,

Through this survey, we want to assess the probable effects of Artificial Intelligence (AI) on mediated communication among people from different cultures. This instrument which been developed by the help of the existing literature and ChatGPT, has 15 structured items with five (5) options per item and six (6) open-ended questions. Your participation in the survey is voluntary and the personal identifications of the participants will be treated as strictly confidential.

Thank you for your cooperation.

The researchers

A. Demographic Information

1. Gender: ☑ Male ☐ Female
2. Age: 33
3. Nationality: malaysia
4. Level of education: post grade degree
5. AI/ ChatGPT experience: ☑ Yes ☐ No

B. Intercultural Sensitivity Scale

**Directions:** The current AI-ICC questionnaire includes 15 items/ statements with five (5) options per item, as: 1 – Strongly agree; 2 – Agree, 3 – Uncertain; 4 – Disagree, and 5 – Strongly disagree.	Strongly agree	Agree	Uncertain	Disagree	Strongly disagree
1. AI and AI-powered machines can be helpful in bridging communication gaps between people from different cultures.	1	2	(3)	4	5
2. So far, AI has been successful in facilitating cross-cultural understanding and collaboration in mediated communication.	1	2	3	(4)	5
3. AI can help overcome language barriers and promote more inclusive communication among diverse groups.	1	(2)	3	4	5

CamScanner

No (15) & P(2)

4. There some cultural biases and stereotypes in AI-powered language tools and communication platforms.	1	2	3	(4)	5
5. AI has affected the localization and adaptation of content for different cultural audiences in digital communication.	1	2	(3)	4	5
6. I encountered any challenges in using AI-powered translation tools when communicating with people from different cultural backgrounds.	1	(2)	3	4	5
7. AI has the potential to promote cultural exchange and appreciation by enabling easier access to diverse perspectives and information.	1	(2)	3	4	5
8. AI-powered machines and chatbots should be programmed to respect and adapt to cultural norms and communication styles while interacting with users from different cultures.	1	2	3	(4)	5
9. There are some potential risks and concerns regarding AI's role in cross-cultural communication, such as perpetuating cultural stereotypes and misinterpretations.	1	2	3	(4)	5
10. I have participated in some cross-cultural virtual collaborations that relied on AI for communication and coordination.	1	2	3	4	(5)
11. I think AI can enhance intercultural learning experiences and foster empathy among individuals from different cultural backgrounds.	1	2	3	(4)	5
12. AI-powered virtual reality (VR) and augmented reality (AR) technologies can impact cross-cultural communication and understanding.	1	2	(3)	4	5
13. Somehow AI-generated content may unintentionally offend and misled individuals from specific cultural backgrounds.	1	2	(3)	4	5
14. Ethical considerations should be taken into account when designing AI-powered communication tools that cater to diverse cultural contexts.	1	2	(3)	4	5
15. Universities and educational institutions leverage AI to create more inclusive and culturally diverse online learning environments.	1	2	(3)	4	5

In the case of your agreement on sharing your answer/ points of views for questions below, please write down your comment/ point of view for all questions or any question that you prefer:

1. Did you experience the use of AI and AI-powered chatbots, such as ChatGPT and for what purpose?

Yes, for paraphrasing sentences, to get an idea of a matter.

2. How do you perceive the role of AI and AI-powered machines in bridging communication gaps between people from different cultures?

it doesn't matter, cultures and knowledge is different.
it's two different thing.

CamScanner

No (13) & P(3)

3. Have you experienced and observed any instances where AI has been successful in facilitating cross-cultural understanding and collaboration in mediated communication?

it help in translating from english to Bahasa Malaysia.
But not all are correct. AI is assisted intelligent not artificial. It is a Tool to help human.

4. In what ways do you think AI can help overcome language barriers and promote more inclusive communication among diverse groups?

no need, its okay as it is.

5. Are there any cultural biases or stereotypes that you have noticed in AI-powered language tools or communication platforms?

No.

6. How can universities and educational institutions leverage AI to create more inclusive and culturally diverse online learning environments?

it's already inclusive & culturally appropriate in my opinion.

Thank you for your time and participation

CS CamScanner

Participant No (16) & P(1)

UNIVERSITI MALAYA

Depart of Media and Communication Studies

AI and Mediated Intercultural Communication Questionnaire

Dear UM Student/ Researcher,

Through this survey, we want to assess the probable effects of Artificial Intelligence (AI) on mediated communication among people from different cultures. This instrument which been developed by the help of the existing literature and ChatGPT, has 15 structured items with five (5) options per item and six (6) open-ended questions. Your participation in the survey is voluntary and the personal identifications of the participants will be treated as strictly confidential.

Thank you for your cooperation.

The researchers

A. Demographic Information

1. Gender: ☐ Male ☑ Female
2. Age: 28
3. Nationality: China
4. Level of education: PhD
5. AI/ ChatGPT experience: ☑ Yes ☐ No

3. Intercultural Sensitivity Scale

Directions: The current AI-ICC questionnaire includes 15 items/ statements with five (5) options per item, as: 1 – Strongly agree; 2 – Agree, 3 – Uncertain; 4 – Disagree, and 5 – Strongly disagree.	Strongly agree	Agree	Uncertain	Disagree	Strongly disagree
1. AI and AI-powered machines can be helpful in bridging communication gaps between people from different cultures.	1	2 ✓	3	4	5
2. So far, AI has been successful in facilitating cross-cultural understanding and collaboration in mediated communication.	1	2	3 ✓	4	5
3. AI can help overcome language barriers and promote more inclusive communication among diverse groups.	1	2 ✓	3	4	5

CamScanner

No (16) & P (2)

4. There some cultural biases and stereotypes in AI-powered language tools and communication platforms.	1	2 ✓	3	4	5
5. AI has affected the localization and adaptation of content for different cultural audiences in digital communication.	1	2	3 ✓	4	5
6. I encountered any challenges in using AI-powered translation tools when communicating with people from different cultural backgrounds.	1	2 ✓	3	4	5
7. AI has the potential to promote cultural exchange and appreciation by enabling easier access to diverse perspectives and information.	1 ✓	2	3	4	5
8. AI-powered machines and chatbots should be programmed to respect and adapt to cultural norms and communication styles while interacting with users from different cultures.	1	2 ✓	3	4	5
9. There are some potential risks and concerns regarding AI's role in cross-cultural communication, such as perpetuating cultural stereotypes and misinterpretations.	1	2 ✓	3	4	5
10. I have participated in some cross-cultural virtual collaborations that relied on AI for communication and coordination.	1 ✓	2	3	4	5
11. I think AI can enhance intercultural learning experiences and foster empathy among individuals from different cultural backgrounds.	1 ✓	2	3	4	5
12. AI-powered virtual reality (VR) and augmented reality (AR) technologies can impact cross-cultural communication and understanding.	1 ✓	2	3	4	5
13. Somehow AI-generated content may unintentionally offend and misled individuals from specific cultural backgrounds.	1	2 ✓	3	4	5
14. Ethical considerations should be taken into account when designing AI-powered communication tools that cater to diverse cultural contexts.	1	2 ✓	3	4	5
15. Universities and educational institutions leverage AI to create more inclusive and culturally diverse online learning environments.	1 ✓	2	3	4	5

In the case of your agreement on sharing your answer/ points of views for questions below, please write down your comment/ point of view for all questions or any question that you prefer:

1. Did you experience the use of AI and AI-powered chatbots, such as ChatGPT and for what purpose?

   Let AI help generate views and perspectives.

2. How do you perceive the role of AI and AI-powered machines in bridging communication gaps between people from different cultures?

   I personally think that AI has playes a big role, and there will be more opportunities for it in the future.

CamScanner

No (16) E, P(3)

3. Have you experienced and observed any instances where AI has been successful in facilitating cross-cultural understanding and collaboration in mediated communication?

Not yet.

4. In what ways do you think AI can help overcome language barriers and promote more inclusive communication among diverse groups?

Language expression, Language habits, etc.

5. Are there any cultural biases or stereotypes that you have noticed in AI-powered language tools or communication platforms?

Yes. For example, the sentence structure of Chinese and English is different, and the meaning of expression is very different.

6. How can universities and educational institutions leverage AI to create more inclusive and culturally diverse online learning environments?

Develop an easy-to-use sofeware or app.

Thank you for your time and participation

CS CamScanner

Partcipant No (17) & P(1)

UNIVERSITI
MALAYA

Depart of Media and Communication Studies

AI and Mediated Intercultural Communication Questionnaire

Dear UM Student/ Researcher,

Through this survey, we want to assess the probable effects of Artificial Intelligence (AI) on mediated communication among people from different cultures. This instrument which been developed by the help of the existing literature and ChatGPT, has 15 structured items with five (5) options per item and six (6) open-ended questions. Your participation in the survey is voluntary and the personal identifications of the participants will be treated as strictly confidential.

Thank you for your cooperation.

The researchers

A. Demographic Information

1. Gender: ☐ Male ✓ Female

2. Age: 31

3. Nationality: China

4. Level of education: PhD. Student

5. AI/ ChatGPT experience: ✓ Yes ☐ No

B. Intercultural Sensitivity Scale

Directions: The current AI-ICC questionnaire includes 15 items/ statements with five (5) options per item, as: 1 – Strongly agree; 2 – Agree, 3 – Uncertain; 4 – Disagree, and 5 – Strongly disagree.	Strongly agree	Agree	Uncertain	Disagree	Strongly disagree
1. AI and AI-powered machines can be helpful in bridging communication gaps between people from different cultures.	1	2 ✓	3	4	5
2. So far, AI has been successful in facilitating cross-cultural understanding and collaboration in mediated communication.	1	2	3 ✓	4	5
3. AI can help overcome language barriers and promote more inclusive communication among diverse groups.	1	2 ✓	3	4	5

CamScanner

No (17) [illegible] P(2)

4. There some cultural biases and stereotypes in AI-powered language tools and communication platforms.	1 ✓	2	3	4	5
5. AI has affected the localization and adaptation of content for different cultural audiences in digital communication.	1	2 ✓	3	4	5
6. I encountered any challenges in using AI-powered translation tools when communicating with people from different cultural backgrounds.	1 ✓	2	3	4	5
7. AI has the potential to promote cultural exchange and appreciation by enabling easier access to diverse perspectives and information.	1	2 ✓	3	4	5
8. AI-powered machines and chatbots should be programmed to respect and adapt to cultural norms and communication styles while interacting with users from different cultures.	1 ✓	2	3	4	5
9. There are some potential risks and concerns regarding AI's role in cross-cultural communication, such as perpetuating cultural stereotypes and misinterpretations.	1 ✓	2	3	4	5
10. I have participated in some cross-cultural virtual collaborations that relied on AI for communication and coordination.	1	2 ✓	3	4	5
11. I think AI can enhance intercultural learning experiences and foster empathy among individuals from different cultural backgrounds.	1	2 ✓	3	4	5
12. AI-powered virtual reality (VR) and augmented reality (AR) technologies can impact cross-cultural communication and understanding.	1 ✓	2	3	4	5
13. Somehow AI-generated content may unintentionally offend and misled individuals from specific cultural backgrounds.	1	2	3 ✓	4	5
14. Ethical considerations should be taken into account when designing AI-powered communication tools that cater to diverse cultural contexts.	1 ✓	2	3	4	5
15. Universities and educational institutions leverage AI to create more inclusive and culturally diverse online learning environments.	1	2 ✓	3	4	5

In the case of your agreement on sharing your answer/ points of views for questions below, please write down your comment/ point of view for all questions or any question that you prefer:

1. Did you experience the use of AI and AI-powered chatbots, such as ChatGPT and for what purpose?

for academic writing

2. How do you perceive the role of AI and AI-powered machines in bridging communication gaps between people from different cultures?

it plays a positive role becoz
it isn't place or time constrained

CamScanner

No (17) & P (3)

3. Have you experienced and observed any instances where AI has been successful in facilitating cross-cultural understanding and collaboration in mediated communication?

Yes, in the lab or museums in foreign countries.

4. In what ways do you think AI can help overcome language barriers and promote more inclusive communication among diverse groups?

it provides authentic learning materials esp. for EFL or second language learners.

5. Are there any cultural biases or stereotypes that you have noticed in AI-powered language tools or communication platforms?

Yes, for cultural bias.

6. How can universities and educational institutions leverage AI to create more inclusive and culturally diverse online learning environments?

Take into account of more cultural factors and establish cross-border institutions.

Thank you for your time and participation

CS CamScanner

Participants No (18) & Page (1)

UNIVERSITI MALAYA

Depart of Media and Communication Studies

AI and Mediated Intercultural Communication Questionnaire

Dear UM Student/ Researcher,

Through this survey, we want to assess the probable effects of Artificial Intelligence (AI) on mediated communication among people from different cultures. This instrument which been developed by the help of the existing literature and ChatGPT, has 15 structured items with five (5) options per item and six (6) open-ended questions. Your participation in the survey is voluntary and the personal identifications of the participants will be treated as strictly confidential.

Thank you for your cooperation.

The researchers

A. Demographic Information

1. Gender: ✓ Male ☐ Female
2. Age: 28
3. Nationality: China
4. Level of education: PhD
5. AI/ ChatGPT experience: (✓) Yes ☐ No

B. Intercultural Sensitivity Scale

Directions: The current AI-ICC questionnaire includes 15 items/ statements with five (5) options per item, as: 1 – Strongly agree; 2 – Agree, 3 – Uncertain; 4 – Disagree, and 5 – Strongly disagree.	Strongly agree	Agree	Uncertain	Disagree	Strongly disagree
1. AI and AI-powered machines can be helpful in bridging communication gaps between people from different cultures.	1	2 ✓	3	4	5
2. So far, AI has been successful in facilitating cross-cultural understanding and collaboration in mediated communication.	1	2 ✓	3	4	5
3. AI can help overcome language barriers and promote more inclusive communication among diverse groups.	1 ✓	2	3	4	5

P

CamScanner

Participant No (18) & Page (2)

	1	2	3	4	5
4. There some cultural biases and stereotypes in AI-powered language tools and communication platforms.	1 ✓	2	3	4	5
5. AI has affected the localization and adaptation of content for different cultural audiences in digital communication.	1	2	3 ✓	4	5
6. I encountered any challenges in using AI-powered translation tools when communicating with people from different cultural backgrounds.	1	2	3	4 ✓	5
7. AI has the potential to promote cultural exchange and appreciation by enabling easier access to diverse perspectives and information.	1	2 ✓	3	4	5
8. AI-powered machines and chatbots should be programmed to respect and adapt to cultural norms and communication styles while interacting with users from different cultures.	1 ✓	2	3	4	5
9. There are some potential risks and concerns regarding AI's role in cross-cultural communication, such as perpetuating cultural stereotypes and misinterpretations.	1 ✓	2	3	4	5
10. I have participated in some cross-cultural virtual collaborations that relied on AI for communication and coordination.	1	2 ✓	3	4	5
11. I think AI can enhance intercultural learning experiences and foster empathy among individuals from different cultural backgrounds.	1	2 ✓	3	4	5
12. AI-powered virtual reality (VR) and augmented reality (AR) technologies can impact cross-cultural communication and understanding.	1	2	3 ✓	4	5
13. Somehow AI-generated content may unintentionally offend and misled individuals from specific cultural backgrounds.	1	2 ✓	3	4	5
14. Ethical considerations should be taken into account when designing AI-powered communication tools that cater to diverse cultural contexts.	1 ✓	2	3	4	5
15. Universities and educational institutions leverage AI to create more inclusive and culturally diverse online learning environments.	1	2	3 ✓	4	5

In the case of your agreement on sharing your answer/ points of views for questions below, please write down your comment/ point of view for all questions or any question that you prefer:

1. Did you experience the use of AI and AI-powered chatbots, such as ChatGPT and for what purpose?

~~[illegible]~~ often such as direction, research, & assignment making & Notes preparation

2. How do you perceive the role of AI and AI-powered machines in bridging communication gaps between people from different cultures?

I think it can improve the efficiency between different people when 2 they use kinds of language. On the other hand, AI skill should put the Ethical [illegible]

Participant No ⑧ E P(3)
(18)

3. Have you experienced and observed any instances where AI has been successful in facilitating cross-cultural understanding and collaboration in mediated communication?

Maybe the AI games and films can improve different kinds of culture to communite.

4. In what ways do you think AI can help overcome language barriers and promote more inclusive communication among diverse groups?

AI dictionary is a good software. It play an important role between different lungnage people.

5. Are there any cultural biases or stereotypes that you have noticed in AI-powered language tools or communication platforms?

Not yet. I haven't noticed something about this question.

6. How can universities and educational institutions leverage AI to create more inclusive and culturally diverse online learning environments?

Maybe we should creat a couse which can help students have a right attitude to use AI technology and use a application to check the assignement of students who have use AI to write thesis.

Thank you for your time and participation

CamScanner

Participant No (19) Er P(1)

UNIVERSITI MALAYA

Depart of Media and Communication Studies

AI and Mediated Intercultural Communication Questionnaire

Dear UM Student/ Researcher,

Through this survey, we want to assess the probable effects of Artificial Intelligence (AI) on mediated communication among people from different cultures. This instrument which been developed by the help of the existing literature and ChatGPT, has 15 structured items with five (5) options per item and six (6) open-ended questions. Your participation in the survey is voluntary and the personal identifications of the participants will be treated as strictly confidential.

Thank you for your cooperation.

The researchers

A. Demographic Information

1. Gender: ☐ Male ☑ Female

2. Age: 25

3. Nationality: CHINA

4. Level of education: P.HD

5. AI/ ChatGPT experience: ☑ Yes ☒ No

B. Intercultural Sensitivity Scale

**Directions:** The current AI-ICC questionnaire includes 15 items/ statements with five (5) options per item, as: 1 – Strongly agree; 2 – Agree, 3 – Uncertain; 4 – Disagree, and 5 – Strongly disagree.	Strongly agree	Agree	Uncertain	Disagree	Strongly disagree
1. AI and AI-powered machines can be helpful in bridging communication gaps between people from different cultures.	1	2 ✓	3	4	5
2. So far, AI has been successful in facilitating cross-cultural understanding and collaboration in mediated communication.	1	2	3 ✓	4	5
3. AI can help overcome language barriers and promote more inclusive communication among diverse groups.	1	2	3	4 ✓	5

No Q(19) & P(2)

4. There some cultural biases and stereotypes in AI-powered language tools and communication platforms.	1	2 ✓	3	4	5
5. AI has affected the localization and adaptation of content for different cultural audiences in digital communication.	1	2 ✓	3	4	5
6. I encountered any challenges in using AI-powered translation tools when communicating with people from different cultural backgrounds.	1	2 ✓	3	4	5
7. AI has the potential to promote cultural exchange and appreciation by enabling easier access to diverse perspectives and information.	1	2 ✓	3	4	5
8. AI-powered machines and chatbots should be programmed to respect and adapt to cultural norms and communication styles while interacting with users from different cultures.	1	2 ✓	3	4	5
9. There are some potential risks and concerns regarding AI's role in cross-cultural communication, such as perpetuating cultural stereotypes and misinterpretations.	1	2	3 ✓	4	5
10. I have participated in some cross-cultural virtual collaborations that relied on AI for communication and coordination.	1	2	3	4 ✓	5
11. I think AI can enhance intercultural learning experiences and foster empathy among individuals from different cultural backgrounds.	1	2	3 ✓	4	5
12. AI-powered virtual reality (VR) and augmented reality (AR) technologies can impact cross-cultural communication and understanding.	1	2 ✓	3	4	5
13. Somehow AI-generated content may unintentionally offend and misled individuals from specific cultural backgrounds.	1	2 ✓	3	4	5
14. Ethical considerations should be taken into account when designing AI-powered communication tools that cater to diverse cultural contexts.	1 ✓	2	3	4	5
15. Universities and educational institutions leverage AI to create more inclusive and culturally diverse online learning environments.	1	2	3 ✓	4	5

In the case of your agreement on sharing your answer/ points of views for questions below, please write down your comment/ point of view for all questions or any question that you prefer:

1. Did you experience the use of AI and AI-powered chatbots, such as ChatGPT and for what purpose?

   No. It's not yet ~~to~~ in use.

2. How do you perceive the role of AI and AI-powered machines in bridging communication gaps between people from different cultures?

   AI does not fully ~~[illegible]~~ fulfill the function of cultural communication, but it can 2 appropriately meet basic needs

CS CamScanner

No (19) & P(3)

3. Have you experienced and observed any instances where AI has been successful in facilitating cross-cultural understanding and collaboration in mediated communication?

Not very familiar.

4. In what ways do you think AI can help overcome language barriers and promote more inclusive communication among diverse groups?

Cultural differences are always presented through language, this barrier, I think, it's difficult to overcome through AI, only help us to understand this gap.

5. Are there any cultural biases or stereotypes that you have noticed in AI-powered language tools or communication platforms?

I'm not very familiar with AI, but I think the cultural contained traditions and customs. In many country, there are many completely different dialects, and the communication between these dialects is difficult. But the characteristic of AI is that it can constantly learn. So can it be trained to overcome this problem?

6. How can universities and educational institutions leverage AI to create more inclusive and culturally diverse online learning environments?

Training AI.

Thank you for your time and participation

CamScanner

Participant No [illegible] & Page ①
(20)

UNIVERSITI MALAYA

Depart of Media and Communication Studies

AI and Mediated Intercultural Communication Questionnaire

Dear UM Student/ Researcher,

Through this survey, we want to assess the probable effects of Artificial Intelligence (AI) on mediated communication among people from different cultures. This instrument which been developed by the help of the existing literature and ChatGPT, has 15 structured items with five (5) options per item and six (6) open-ended questions. Your participation in the survey is voluntary and the personal identifications of the participants will be treated as strictly confidential.

Thank you for your cooperation.

The researchers

A. Demographic Information

1. Gender: ☐ Male ☑ Female

2. Age: 46

3. Nationality: Pakistani

4. Level of education: P.hD student.

5. AI/ ChatGPT experience: ☑ Yes ☐ No

B. Intercultural Sensitivity Scale

Directions: The current AI-ICC questionnaire includes 15 items/ statements with five (5) options per item, as: 1 – Strongly agree; 2 – Agree, 3 – Uncertain; 4 – Disagree, and 5 – Strongly disagree.	Strongly agree	Agree	Uncertain	Disagree	Strongly disagree
1. AI and AI-powered machines can be helpful in bridging communication gaps between people from different cultures.	1	✓ 2	3	4	5
2. So far, AI has been successful in facilitating cross-cultural understanding and collaboration in mediated communication.	1	✓ 2	3	4	5
3. AI can help overcome language barriers and promote more inclusive communication among diverse groups.	1	✓ 2	3	4	5

CamScanner

Participant No [illegible] & Page ①
(20)

UNIVERSITI MALAYA

Depart of Media and Communication Studies

AI and Mediated Intercultural Communication Questionnaire

Dear UM Student/ Researcher,

Through this survey, we want to assess the probable effects of Artificial Intelligence (AI) on mediated communication among people from different cultures. This instrument which been developed by the help of the existing literature and ChatGPT, has 15 structured items with five (5) options per item and six (6) open-ended questions. Your participation in the survey is voluntary and the personal identifications of the participants will be treated as strictly confidential.

Thank you for your cooperation.

The researchers

A. Demographic Information

1. Gender: ☐ Male ☑ Female

2. Age: 46

3. Nationality: Pakistani

4. Level of education: P.hD student.

5. AI/ ChatGPT experience: ☑ Yes ☐ No

B. Intercultural Sensitivity Scale

**Directions:** The current AI-ICC questionnaire includes 15 items/ statements with five (5) options per item, as: 1 – Strongly agree; 2 – Agree, 3 – Uncertain; 4 – Disagree, and 5 – Strongly disagree.	Strongly agree	Agree	Uncertain	Disagree	Strongly disagree
1. AI and AI-powered machines can be helpful in bridging communication gaps between people from different cultures.	1	✓2	3	4	5
2. So far, AI has been successful in facilitating cross-cultural understanding and collaboration in mediated communication.	1	✓2	3	4	5
3. AI can help overcome language barriers and promote more inclusive communication among diverse groups.	1	✓2	3	4	5

CamScanner

Participant No (20) & Page (2)

4. There some cultural biases and stereotypes in AI-powered language tools and communication platforms.	1	2	3	4 ✓	5
5. AI has affected the localization and adaptation of content for different cultural audiences in digital communication.	1	2 ✓	3	4	5
6. I encountered any challenges in using AI-powered translation tools when communicating with people from different cultural backgrounds.	1	2	3	4 ✓	5
7. AI has the potential to promote cultural exchange and appreciation by enabling easier access to diverse perspectives and information.	1 ✓	2	3	4	5
8. AI-powered machines and chatbots should be programmed to respect and adapt to cultural norms and communication styles while interacting with users from different cultures.	1 ✓	2	3	4	5
9. There are some potential risks and concerns regarding AI's role in cross-cultural communication, such as perpetuating cultural stereotypes and misinterpretations.	1	2	3	4 ✓	5
10. I have participated in some cross-cultural virtual collaborations that relied on AI for communication and coordination.	1	2	3	4	5 ✓
11. I think AI can enhance intercultural learning experiences and foster empathy among individuals from different cultural backgrounds.	1 ✓	2	3	4	5
12. AI-powered virtual reality (VR) and augmented reality (AR) technologies can impact cross-cultural communication and understanding.	1	2 ✓	3	4	5
13. Somehow AI-generated content may unintentionally offend and misled individuals from specific cultural backgrounds.	1	2	3	4 ✓	5
14. Ethical considerations should be taken into account when designing AI-powered communication tools that cater to diverse cultural contexts.	1 ✓	2	3	4	5
15. Universities and educational institutions leverage AI to create more inclusive and culturally diverse online learning environments.	1	2	3 ✓	4	5

In the case of your agreement on sharing your answer/ points of views for questions below, please write down your comment/ point of view for all questions or any question that you prefer:

1. Did you experience the use of AI and AI-powered chatbots, such as ChatGPT and for what purpose?

Yes I use AI for transcription of my reseach data and it is beneficial and user friendly.

2. How do you perceive the role of AI and AI-powered machines in bridging communication gaps between people from different cultures?

It helps people to communicate with each other despite language barries which help them to interact and familiarize with the people of different regions.

CamScanner

Participant No [illegible] (20) & Page (3)

3. Have you experienced and observed any instances where AI has been successful in facilitating cross-cultural understanding and collaboration in mediated communication?

Yes I observed and experienced it and the results were quite satisfactory.

4. In what ways do you think AI can help overcome language barriers and promote more inclusive communication among diverse groups?

It provides translation facility, coding different languages, exploring cultural similarities etc. Overall it reduces human effort and facilitate people to interact with each other in a better way.

5. Are there any cultural biases or stereotypes that you have noticed in AI-powered language tools or communication platforms?

No, as far as my experience I have not observed any biases while using AI so far.

6. How can universities and educational institutions leverage AI to create more inclusive and culturally diverse online learning environments?

It is useful tool for interacting and understanding various cultures but for content generation and research there should be some limitations on its use.

Thank you for your time and participation

CamScanner

Participant No (21) & Page (1)

UNIVERSITI MALAYA

Depart of Media and Communication Studies

AI and Mediated Intercultural Communication Questionnaire

Dear UM Student/ Researcher,

Through this survey, we want to assess the probable effects of Artificial Intelligence (AI) on mediated communication among people from different cultures. This instrument which been developed by the help of the existing literature and ChatGPT, has 15 structured items with five (5) options per item and six (6) open-ended questions. Your participation in the survey is voluntary and the personal identifications of the participants will be treated as strictly confidential.

Thank you for your cooperation.

The researchers

A. Demographic Information

1. Gender: ☑ Male ☐ Female

2. Age: 40

3. Nationality: pakistani

4. Level of education: PhD student

5. AI/ ChatGPT experience: ☑ Yes ☐ No

B. Intercultural Sensitivity Scale

Directions: The current AI-ICC questionnaire includes 15 items/ statements with five (5) options per item, as: 1 – Strongly agree; 2 – Agree, 3 – Uncertain; 4 – Disagree, and 5 – Strongly disagree.	Strongly agree	Agree	Uncertain	Disagree	Strongly disagree
1. AI and AI-powered machines can be helpful in bridging communication gaps between people from different cultures.	1	2 ✓	3	4	5
2. So far, AI has been successful in facilitating cross-cultural understanding and collaboration in mediated communication.	1	2	3 ✓	4	5
3. AI can help overcome language barriers and promote more inclusive communication among diverse groups.	1	2 ✓	3	4	5

CamScanner

No (21) & P(2)

4. There some cultural biases and stereotypes in AI-powered language tools and communication platforms.	1 ✓	2	3	4	5
5. AI has affected the localization and adaptation of content for different cultural audiences in digital communication.	1	2	3 ✓	4	5
6. I encountered any challenges in using AI-powered translation tools when communicating with people from different cultural backgrounds.	1	2	3 ✓	4	5
7. AI has the potential to promote cultural exchange and appreciation by enabling easier access to diverse perspectives and information.	1	2 ✓	3	4	5
8. AI-powered machines and chatbots should be programmed to respect and adapt to cultural norms and communication styles while interacting with users from different cultures.	1 ✓	2	3	4	5
9. There are some potential risks and concerns regarding AI's role in cross-cultural communication, such as perpetuating cultural stereotypes and misinterpretations.	1 ✓	2	3	4	5
10. I have participated in some cross-cultural virtual collaborations that relied on AI for communication and coordination.	1	2	3 ✓	4	5
11. I think AI can enhance intercultural learning experiences and foster empathy among individuals from different cultural backgrounds.	1	2 ✓	3	4	5
12. AI-powered virtual reality (VR) and augmented reality (AR) technologies can impact cross-cultural communication and understanding.	1	2	3 ✓	4	5
13. Somehow AI-generated content may unintentionally offend and misled individuals from specific cultural backgrounds.	1 ✓	2	3	4	5
14. Ethical considerations should be taken into account when designing AI-powered communication tools that cater to diverse cultural contexts.	1 ✓	2	3	4	5
15. Universities and educational institutions leverage AI to create more inclusive and culturally diverse online learning environments.	1	2	3 ✓	4	5

In the case of your agreement on sharing your answer/ points of views for questions below, please write down your comment/ point of view for all questions or any question that you prefer:

1. Did you experience the use of AI and AI-powered chatbots, such as ChatGPT and for what purpose?

I use AI apps for general information and especially Research purposes

2. How do you perceive the role of AI and AI-powered machines in bridging communication gaps between people from different cultures?

AI as a personal assistant can help over come many barriers. However, it needs a regulatory policy to over come bias and privacy issues.

CS CamScanner

No (21) E, P(3)

3. Have you experienced and observed any instances where AI has been successful in facilitating cross-cultural understanding and collaboration in mediated communication?

No

4. In what ways do you think AI can help overcome language barriers and promote more inclusive communication among diverse groups?

Its translation features can bridge language barriers.

5. Are there any cultural biases or stereotypes that you have noticed in AI-powered language tools or communication platforms?

Yes, cultural, religious biases exists in AI apps and tools.

6. How can universities and educational institutions leverage AI to create more inclusive and culturally diverse online learning environments?

Through open debates and feedback.

Thank you for your time and participation

CamScanner

Participant No (22) & P(1)

UNIVERSITI MALAYA

Depart of Media and Communication Studies

AI and Mediated Intercultural Communication Questionnaire

Dear UM Student/ Researcher,

Through this survey, we want to assess the probable effects of Artificial Intelligence (AI) on mediated communication among people from different cultures. This instrument which been developed by the help of the existing literature and ChatGPT, has 15 structured items with five (5) options per item and six (6) open-ended questions. Your participation in the survey is voluntary and the personal identifications of the participants will be treated as strictly confidential.

Thank you for your cooperation.

The researchers

A. Demographic Information

1. Gender: ☐ Male ☒ Female

2. Age: 26

3. Nationality: Malaysia

4. Level of education: post-Bachelor Degree

5. AI/ ChatGPT experience: ☒ Yes ☒ No

B. Intercultural Sensitivity Scale

Directions: The current AI-ICC questionnaire includes 15 items/ statements with five (5) options per item, as: 1 – Strongly agree; 2 – Agree, 3 – Uncertain; 2 – Disagree, and 5 – Strongly disagree.	Strongly agree	Agree	Uncertain	Disagree	Strongly disagree
1. AI and AI-powered machines can be helpful in bridging communication gaps between people from different cultures.	1	(2)	3	4	5
2. So far, AI has been successful in facilitating cross-cultural understanding and collaboration in mediated communication.	1	2	(3)	4	5
3. AI can help overcome language barriers and promote more inclusive communication among diverse groups.	1	(2)	3	4	5

CamScanner

No 22 E P2

4. There some cultural biases and stereotypes in AI-powered language tools and communication platforms.	(1)	2	3	4	5
5. AI has affected the localization and adaptation of content for different cultural audiences in digital communication.	1	2	3	(4)	5
6. I encountered any challenges in using AI-powered translation tools when communicating with people from different cultural backgrounds.	1	2	(3)	4	5
7. AI has the potential to promote cultural exchange and appreciation by enabling easier access to diverse perspectives and information.	1	(2)	3	4	5
8. AI-powered machines and chatbots should be programmed to respect and adapt to cultural norms and communication styles while interacting with users from different cultures.	(1)	2	3	4	5
9. There are some potential risks and concerns regarding AI's role in cross-cultural communication, such as perpetuating cultural stereotypes and misinterpretations.	1	2	(3)	4	5
10. I have participated in some cross-cultural virtual collaborations that relied on AI for communication and coordination.	1	2	3	(4)	5
11. I think AI can enhance intercultural learning experiences and foster empathy among individuals from different cultural backgrounds.	(1)	2	3	4	5
12. AI-powered virtual reality (VR) and augmented reality (AR) technologies can impact cross-cultural communication and understanding.	(1)	2	3	4	5
13. Somehow AI-generated content may unintentionally offend and misled individuals from specific cultural backgrounds.	1	(2)	3	4	5
14. Ethical considerations should be taken into account when designing AI-powered communication tools that cater to diverse cultural contexts.	1	(2)	3	4	5
15. Universities and educational institutions leverage AI to create more inclusive and culturally diverse online learning environments.	(1)	2	3	4	5

In the case of your agreement on sharing your answer/ points of views for questions below, please write down your comment/ point of view for all questions or any question that you prefer:

1. Did you experience the use of AI and AI-powered chatbots, such as ChatGPT and for what purpose?

   For research purpose, just to obtain more info in shorter time

2. How do you perceive the role of AI and AI-powered machines in bridging communication gaps between people from different cultures?

   AI may be more neutral in its roles but may be biased ~~[illegible]~~ to the majority view.

CS CamScanner

No (22) & P(3)

3. Have you experienced and observed any instances where AI has been successful in facilitating cross-cultural understanding and collaboration in mediated communication?

Not really. I don't use it that frequently for this purpose

4. In what ways do you think AI can help overcome language barriers and promote more inclusive communication among diverse groups?

Perhaps AI can obtain info from different platforms, languages and sources and translate them in a common language that the users can understand

5. Are there any cultural biases or stereotypes that you have noticed in AI-powered language tools or communication platforms?

When the publicly available information are mostly published by a certain group of people which are the majority, AI tools may tend to be biased to those info.

6. How can universities and educational institutions leverage AI to create more inclusive and culturally diverse online learning environments?

Build a centralised system for students from different faculties to learn and interact. Would be best if it can be extended to other universities or countries for more knowledge exchange opportunities.

Thank you for your time and participation

CamScanner

Participant No (23) & PO

UNIVERSITI MALAYA

Depart of Media and Communication Studies

AI and Mediated Intercultural Communication Questionnaire

Dear UM Student/ Researcher,

Through this survey, we want to assess the probable effects of Artificial Intelligence (AI) on mediated communication among people from different cultures. This instrument which been developed by the help of the existing literature and ChatGPT, has 15 structured items with five (5) options per item and six (6) open-ended questions. Your participation in the survey is voluntary and the personal identifications of the participants will be treated as strictly confidential.

Thank you for your cooperation.

The researchers

A. Demographic Information

1. Gender: ☐ Male ☑ Female

2. Age: 40 Years Old.

3. Nationality: Malaysia

4. Level of education: PhD

5. AI/ ChatGPT experience: ☑ Yes ☐ No

B. Intercultural Sensitivity Scale

Directions: The current AI-ICC questionnaire includes 15 items/ statements with five (5) options per item, as: 1 – Strongly agree; 2 – Agree, 3 – Uncertain; 4 – Disagree, and 5 – Strongly disagree.	Strongly agree	Agree	Uncertain	Disagree	Strongly disagree
1. AI and AI-powered machines can be helpful in bridging communication gaps between people from different cultures.	1	2	(3)	4	5
2. So far, AI has been successful in facilitating cross-cultural understanding and collaboration in mediated communication.	1	(2)	3	4	5
3. AI can help overcome language barriers and promote more inclusive communication among diverse groups.	1	(2)	3	4	5

CamScanner

No (23) E1 P(2)

4. There some cultural biases and stereotypes in AI-powered language tools and communication platforms.	1	2	**(3)**	4	5
5. AI has affected the localization and adaptation of content for different cultural audiences in digital communication.	1	**(2)**	3	4	5
6. I encountered any challenges in using AI-powered translation tools when communicating with people from different cultural backgrounds.	1	**(2)**	3	4	5
7. AI has the potential to promote cultural exchange and appreciation by enabling easier access to diverse perspectives and information.	1	**(2)**	3	4	5
8. AI-powered machines and chatbots should be programmed to respect and adapt to cultural norms and communication styles while interacting with users from different cultures.	1	**(2)**	3	4	5
9. There are some potential risks and concerns regarding AI's role in cross-cultural communication, such as perpetuating cultural stereotypes and misinterpretations.	**(1)**	2	3	4	5
10. I have participated in some cross-cultural virtual collaborations that relied on AI for communication and coordination.	1	2	**(3)**	4	5
11. I think AI can enhance intercultural learning experiences and foster empathy among individuals from different cultural backgrounds.	1	**(2)**	3	4	5
12. AI-powered virtual reality (VR) and augmented reality (AR) technologies can impact cross-cultural communication and understanding.	**(1)**	2	3	4	5
13. Somehow AI-generated content may unintentionally offend and misled individuals from specific cultural backgrounds.	1	**(2)**	3	4	5
14. Ethical considerations should be taken into account when designing AI-powered communication tools that cater to diverse cultural contexts.	1	**(2)**	3	4	5
15. Universities and educational institutions leverage AI to create more inclusive and culturally diverse online learning environments.	1	**(2)**	3	4	5

In the case of your agreement on sharing your answer/ points of views for questions below, please write down your comment/ point of view for all questions or any question that you prefer:

1. Did you experience the use of AI and AI-powered chatbots, such as ChatGPT and for what purpose?

- Seeking for answer on literature writing in study purpos
- Create presentation slide.
- Seeking answer from Customer service chatbots in websit

2. How do you perceive the role of AI and AI-powered machines in bridging communication gaps between people from different cultures?

Not really promote great impact for different cultures, unless for traveller or tourist perhaps.

CS CamScanner

No (23) & P(3)

3. Have you experienced and observed any instances where AI has been successful in facilitating cross-cultural understanding and collaboration in mediated communication?

Maybe, but not so obvious in real life experience.

4. In what ways do you think AI can help overcome language barriers and promote more inclusive communication among diverse groups?

create application or techonology enhanced integrated with AI to help human communication/connection.

5. Are there any cultural biases or stereotypes that you have noticed in AI-powered language tools or communication platforms?

Not sure.

6. How can universities and educational institutions leverage AI to create more inclusive and culturally diverse online learning environments?

Building more institution application or system integrate with AI.

Thank you for your time and participation

CamScanner

Participant No (24) & P(1)

UNIVERSITI
MALAYA

Depart of Media and Communication Studies

AI and Mediated Intercultural Communication Questionnaire

Dear UM Student/ Researcher,

Through this survey, we want to assess the probable effects of Artificial Intelligence (AI) on mediated communication among people from different cultures. This instrument which been developed by the help of the existing literature and ChatGPT, has 15 structured items with five (5) options per item and six (6) open-ended questions. Your participation in the survey is voluntary and the personal identifications of the participants will be treated as strictly confidential.

Thank you for your cooperation.

The researchers

A. Demographic Information

1. Gender: ☐ Male ☑ Female

2. Age: 35

3. Nationality: Malaysian.

4. Level of education: post Secondary grade degree

5. AI/ ChatGPT experience: ☑ Yes ☐ No

B. Intercultural Sensitivity Scale

Directions: The current AI-ICC questionnaire includes 15 items/ statements with five (5) options per item, as: 1 – Strongly agree; 2 – Agree, 3 – Uncertain; 4 – Disagree, and 5 – Strongly disagree.	Strongly agree	Agree	Uncertain	Disagree	Strongly disagree
1. AI and AI-powered machines can be helpful in bridging communication gaps between people from different cultures.	1	(2)	3	4	5
2. So far, AI has been successful in facilitating cross-cultural understanding and collaboration in mediated communication.	1	(2)	3	4	5
3. AI can help overcome language barriers and promote more inclusive communication among diverse groups.	(1)	2	3	4	5

CamScanner

Participant No (24) [illegible]

4. There some cultural biases and stereotypes in AI-powered language tools and communication platforms.	1	2	3	4	5
5. AI has affected the localization and adaptation of content for different cultural audiences in digital communication.	1	2	3	4	5
6. I encountered any challenges in using AI-powered translation tools when communicating with people from different cultural backgrounds.	1	2	3	4	5
7. AI has the potential to promote cultural exchange and appreciation by enabling easier access to diverse perspectives and information.	1	2	3	4	5
8. AI-powered machines and chatbots should be programmed to respect and adapt to cultural norms and communication styles while interacting with users from different cultures.	1	2	3	4	5
9. There are some potential risks and concerns regarding AI's role in cross-cultural communication, such as perpetuating cultural stereotypes and misinterpretations.	1	2	3	4	5
10. I have participated in some cross-cultural virtual collaborations that relied on AI for communication and coordination.	1	2	3	4	5
11. I think AI can enhance intercultural learning experiences and foster empathy among individuals from different cultural backgrounds.	1	2	3	4	5
12. AI-powered virtual reality (VR) and augmented reality (AR) technologies can impact cross-cultural communication and understanding.	1	2	3	4	5
13. Somehow AI-generated content may unintentionally offend and misled individuals from specific cultural backgrounds.	1	2	3	4	5
14. Ethical considerations should be taken into account when designing AI-powered communication tools that cater to diverse cultural contexts.	1	2	3	4	5
15. Universities and educational institutions leverage AI to create more inclusive and culturally diverse online learning environments.	1	2	3	4	5

In the case of your agreement on sharing your answer/ points of views for questions below, please write down your comment/ point of view for all questions or any question that you prefer:

1. Did you experience the use of AI and AI-powered chatbots, such as ChatGPT and for what purpose?

getting certain knowledge & also improving language construction

2. How do you perceive the role of AI and AI-powered machines in bridging communication gaps between people from different cultures?

It can overcome communication barriers especially in medical field, whereby the doctors & patients can get more clear & precise. understanding ~~among~~ between each other.

No (24) & P(3)

3. Have you experienced and observed any instances where AI has been successful in facilitating cross-cultural understanding and collaboration in mediated communication?

Have used google translator & Temi, which found useful.

4. In what ways do you think AI can help overcome language barriers and promote more inclusive communication among diverse groups?

improve understanding, information exchange & instructions.

5. Are there any cultural biases or stereotypes that you have noticed in AI-powered language tools or communication platforms?

the detection of the spoken language is poor for non-caucasian.

6. How can universities and educational institutions leverage AI to create more inclusive and culturally diverse online learning environments?

Not sure.

Thank you for your time and participation

CamScanner

Participant No (25) E PO

UNIVERSITI MALAYA

Depart of Media and Communication Studies

AI and Mediated Intercultural Communication Questionnaire

Dear UM Student/ Researcher,

Through this survey, we want to assess the probable effects of Artificial Intelligence (AI) on mediated communication among people from different cultures. This instrument which been developed by the help of the existing literature and ChatGPT, has 15 structured items with five (5) options per item and six (6) open-ended questions. Your participation in the survey is voluntary and the personal identifications of the participants will be treated as strictly confidential.

Thank you for your cooperation.

The researchers

A. Demographic Information

1. Gender: ☑ Male ☐ Female
2. Age: 34
3. Nationality: Pakistan
4. Level of education: Ph.D
5. AI/ ChatGPT experience: ☑ Yes ☐ No

B. Intercultural Sensitivity Scale

Directions: The current AI-ICC questionnaire includes 15 items/ statements with five (5) options per item, as: 1 – Strongly agree; 2 – Agree, 3 – Uncertain; 4 – Disagree, and 5 – Strongly disagree.	Strongly agree	Agree	Uncertain	Disagree	Strongly disagree
1. AI and AI-powered machines can be helpful in bridging communication gaps between people from different cultures.	1 ✓	2	3	4	5
2. So far, AI has been successful in facilitating cross-cultural understanding and collaboration in mediated communication.	1 ✓	2	3	4	5
3. AI can help overcome language barriers and promote more inclusive communication among diverse groups.	1	2 ✓	3	4	5

CamScanner

No (25) E (R)

4. There some cultural biases and stereotypes in AI-powered language tools and communication platforms.	1	2	3 ✓	4	5
5. AI has affected the localization and adaptation of content for different cultural audiences in digital communication.	1	2 ✓	3	4	5
6. I encountered any challenges in using AI-powered translation tools when communicating with people from different cultural backgrounds.	1	2 ✓	3	4	5
7. AI has the potential to promote cultural exchange and appreciation by enabling easier access to diverse perspectives and information.	1	2 ✓	3	4	5
8. AI-powered machines and chatbots should be programmed to respect and adapt to cultural norms and communication styles while interacting with users from different cultures.	1	2	3 ✓	4	5
9. There are some potential risks and concerns regarding AI's role in cross-cultural communication, such as perpetuating cultural stereotypes and misinterpretations.	1	2	3 ✓	4	5
10. I have participated in some cross-cultural virtual collaborations that relied on AI for communication and coordination.	1	2 ✓	3	4	5
11. I think AI can enhance intercultural learning experiences and foster empathy among individuals from different cultural backgrounds.	1	2	3	4 ✓	5
12. AI-powered virtual reality (VR) and augmented reality (AR) technologies can impact cross-cultural communication and understanding.	1	2 ✓	3	4	5
13. Somehow AI-generated content may unintentionally offend and misled individuals from specific cultural backgrounds.	1	2 ✓	3	4	5
14. Ethical considerations should be taken into account when designing AI-powered communication tools that cater to diverse cultural contexts.	1 ✓	2	3	4	5
15. Universities and educational institutions leverage AI to create more inclusive and culturally diverse online learning environments.	1 ✓	2	3	4	5

In the case of your agreement on sharing your answer/ points of views for questions below, please write down your comment/ point of view for all questions or any question that you prefer:

1. Did you experience the use of AI and AI-powered chatbots, such as ChatGPT and for what purpose?

yes, I have experience for education purpose, for assignment making, Research.

2. How do you perceive the role of AI and AI-powered machines in bridging communication gaps between people from different cultures?

yes, AI playing a marvelous role in the bridging this gap. It is significa[illegible]

for [illegible]

No P(25) & P(3)

3. Have you experienced and observed any instances where AI has been successful in facilitating cross-cultural understanding and collaboration in mediated communication?

Yes, in every aspect of life, I noticed.

4. In what ways do you think AI can help overcome language barriers and promote more inclusive communication among diverse groups?

yes, due to different AI language translation & understanding & Data processing ability make 2 ways -

5. Are there any cultural biases or stereotypes that you have noticed in AI-powered language tools or communication platforms?

No, I never seen such stereotype in the use of AI.

6. How can universities and educational institutions leverage AI to create more inclusive and culturally diverse online learning environments?

of universities & institution, started program coaching, training, workshop, then, it would be better.

Thank you for your time and participation

CS CamScanner

Participant No (26) & P (1)

UNIVERSITI MALAYA

Depart of Media and Communication Studies

AI and Mediated Intercultural Communication Questionnaire

Dear UM Student/ Researcher,

Through this survey, we want to assess the probable effects of Artificial Intelligence (AI) on mediated communication among people from different cultures. This instrument which been developed by the help of the existing literature and ChatGPT, has 15 structured items with five (5) options per item and six (6) open-ended questions. Your participation in the survey is voluntary and the personal identifications of the participants will be treated as strictly confidential.

Thank you for your cooperation.

The researchers

A. Demographic Information

1. Gender: ☑ Male ☐ Female

2. Age: 35

3. Nationality: Indonesia (Indonesie)

4. Level of education: Master

5. AI/ ChatGPT experience: ☑ Yes ☐ No

B. Intercultural Sensitivity Scale

Directions: The current AI-ICC questionnaire includes 15 items/ statements with five (5) options per item, as: 1 – Strongly agree; 2 – Agree, 3 – Uncertain; 4 – Disagree, and 5 – Strongly disagree.	Strongly agree	Agree	Uncertain	Disagree	Strongly disagree
1. AI and AI-powered machines can be helpful in bridging communication gaps between people from different cultures.	1	2	(3)	4	5
2. So far, AI has been successful in facilitating cross-cultural understanding and collaboration in mediated communication.	1	2	(3)	4	5
3. AI can help overcome language barriers and promote more inclusive communication among diverse groups.	1	(2)	3	4	5

CamScanner

No (26) & P (2)

4. There some cultural biases and stereotypes in AI-powered language tools and communication platforms.	1	**2**	3	4	5
5. AI has affected the localization and adaptation of content for different cultural audiences in digital communication.	1	**2**	3	4	5
6. I encountered any challenges in using AI-powered translation tools when communicating with people from different cultural backgrounds.	1	2	**3**	4	5
7. AI has the potential to promote cultural exchange and appreciation by enabling easier access to diverse perspectives and information.	1	**2**	3	4	5
8. AI-powered machines and chatbots should be programmed to respect and adapt to cultural norms and communication styles while interacting with users from different cultures.	1	2	**3**	4	5
9. There are some potential risks and concerns regarding AI's role in cross-cultural communication, such as perpetuating cultural stereotypes and misinterpretations.	**1**	2	3	4	5
10. I have participated in some cross-cultural virtual collaborations that relied on AI for communication and coordination.	1	2	**3**	4	5
11. I think AI can enhance intercultural learning experiences and foster empathy among individuals from different cultural backgrounds.	1	2	**3**	4	5
12. AI-powered virtual reality (VR) and augmented reality (AR) technologies can impact cross-cultural communication and understanding.	1	**2**	3	4	5
13. Somehow AI-generated content may unintentionally offend and misled individuals from specific cultural backgrounds.	1	**2**	3	4	5
14. Ethical considerations should be taken into account when designing AI-powered communication tools that cater to diverse cultural contexts.	1	2	3	**4**	5
15. Universities and educational institutions leverage AI to create more inclusive and culturally diverse online learning environments.	1	**2**	3	4	5

In the case of your agreement on sharing your answer/ points of views for questions below, please write down your comment/ point of view for all questions or any question that you prefer:

1. Did you experience the use of AI and AI-powered chatbots, such as ChatGPT and for what purpose?

   yes, for Educational, Research purpose & assignment making.

2. How do you perceive the role of AI and AI-powered machines in bridging communication gaps between people from different cultures?

   yes, It play a important role to fill this Gap.

CamScanner

No (26) E P(3)

3. Have you experienced and observed any instances where AI has been successful in facilitating cross-cultural understanding and collaboration in mediated communication?

I have no experience about it

4. In what ways do you think AI can help overcome language barriers and promote more inclusive communication among diverse groups?

I think that AI can help overcome language and promote more but no to any cultural biases

5. Are there any cultural biases or stereotypes that you have noticed in AI-powered language tools or communication platforms?

AI Cannot to the Cultural Biases

6. How can universities and educational institutions leverage AI to create more inclusive and culturally diverse online learning environments?

May be in the future, this knowledge can be done, but I don't know to be for sure

Thank you for your time and participation

CamScanner

Partecepant No (27) E1 P(1)

UNIVERSITI MALAYA

Depart of Media and Communication Studies

AI and Mediated Intercultural Communication Questionnaire

Dear UM Student/ Researcher,

Through this survey, we want to assess the probable effects of Artificial Intelligence (AI) on mediated communication among people from different cultures. This instrument which been developed by the help of the existing literature and ChatGPT, has 15 structured items with five (5) options per item and six (6) open-ended questions. Your participation in the survey is voluntary and the personal identifications of the participants will be treated as strictly confidential.

Thank you for your cooperation.

The researchers

A. Demographic Information

1. Gender: ☐ Male ☑ Female

2. Age: [illegible]

3. Nationality: Malaysian

4. Level of education: ~~[illegible]~~ post-grade

5. AI/ ChatGPT experience: ☐ Yes ☑ No

B. Intercultural Sensitivity Scale

Directions: The current AI-ICC questionnaire includes 15 items/ statements with five (5) options per item, as: 1 – Strongly agree; 2 – Agree, 3 – Uncertain; 4 – Disagree, and 5 – Strongly disagree.	Strongly agree	Agree	Uncertain	Disagree	Strongly disagree
1. AI and AI-powered machines can be helpful in bridging communication gaps between people from different cultures.	(1)	2	3	4	5
2. So far, AI has been successful in facilitating cross-cultural understanding and collaboration in mediated communication.	(1)	2	3	4	5
3. AI can help overcome language barriers and promote more inclusive communication among diverse groups.	(1)	2	3	4	5

CamScanner

N/o (27) E1 P(2)

4. There some cultural biases and stereotypes in AI-powered language tools and communication platforms.	1	2	(3)	4	5
5. AI has affected the localization and adaptation of content for different cultural audiences in digital communication.	1	(2)	3	4	5
6. I encountered any challenges in using AI-powered translation tools when communicating with people from different cultural backgrounds.	1	2	(3)	4	5
7. AI has the potential to promote cultural exchange and appreciation by enabling easier access to diverse perspectives and information.	(1)	2	3	4	5
8. AI-powered machines and chatbots should be programmed to respect and adapt to cultural norms and communication styles while interacting with users from different cultures.	(1)	2	3	4	5
9. There are some potential risks and concerns regarding AI's role in cross-cultural communication, such as perpetuating cultural stereotypes and misinterpretations.	1	(2)	3	4	5
10. I have participated in some cross-cultural virtual collaborations that relied on AI for communication and coordination.	1	2	3	4	(5)
11. I think AI can enhance intercultural learning experiences and foster empathy among individuals from different cultural backgrounds.	1	(2)	3	4	5
12. AI-powered virtual reality (VR) and augmented reality (AR) technologies can impact cross-cultural communication and understanding.	1	(2)	3	4	5
13. Somehow AI-generated content may unintentionally offend and misled individuals from specific cultural backgrounds.	(1)	2	3	4	5
14. Ethical considerations should be taken into account when designing AI-powered communication tools that cater to diverse cultural contexts.	(1)	2	3	4	5
15. Universities and educational institutions leverage AI to create more inclusive and culturally diverse online learning environments.	(1)	2	3	4	5

In the case of your agreement on sharing your answer/ points of views for questions below, please write down your comment/ point of view for all questions or any question that you prefer:

1. Did you experience the use of AI and AI-powered chatbots, such as ChatGPT and for what purpose?

I know of its existence, but I've never had the chance to use it.

2. How do you perceive the role of AI and AI-powered machines in bridging communication gaps between people from different cultures?

~~AI can t~~ I believe AI can be used to bridge gaps between cultures, but as it is a machine, it can be programmed to be insensitive and culturally blind towards issues in certain communities.

CS CamScanner

No (27) G P(3)

3. Have you experienced and observed any instances where AI has been successful in facilitating cross-cultural understanding and collaboration in mediated communication?

No, but I can see how it can do it.

4. In what ways do you think AI can help overcome language barriers and promote more inclusive communication among diverse groups?

It's a man-made machine. It can be done.

5. Are there any cultural biases or stereotypes that you have noticed in AI-powered language tools or communication platforms?

Not personally, ~~but it~~.

6. How can universities and educational institutions leverage AI to create more inclusive and culturally diverse online learning environments?

idk sorry i'm not creative or inovational like that.

Thank you for your time and participation

CS CamScanner

Participant No (28) & P(1)

UNIVERSITI MALAYA

Depart of Media and Communication Studies

AI and Mediated Intercultural Communication Questionnaire

Dear UM Student/ Researcher,

Through this survey, we want to assess the probable effects of Artificial Intelligence (AI) on mediated communication among people from different cultures. This instrument which been developed by the help of the existing literature and ChatGPT, has 15 structured items with five (5) options per item and six (6) open-ended questions. Your participation in the survey is voluntary and the personal identifications of the participants will be treated as strictly confidential.

Thank you for your cooperation.

The researchers

A. Demographic Information

1. Gender: ☐ Male ✓Female
2. Age: 29
3. Nationality: CHINESE
4. Level of education: PhD
5. AI/ ChatGPT experience: ✓Yes ☐ No

B. Intercultural Sensitivity Scale

**Directions:** The current AI-ICC questionnaire includes 15 items/ statements with five (5) options per item, as: 1 – Strongly agree; 2 – Agree, 3 – Uncertain; 4 – Disagree, and 5 – Strongly disagree.	Strongly agree	Agree	Uncertain	Disagree	Strongly disagree
1. AI and AI-powered machines can be helpful in bridging communication gaps between people from different cultures.	1	2 ✓	3	4	5
2. So far, AI has been successful in facilitating cross-cultural understanding and collaboration in mediated communication.	1	2	3 ✓	4	5
3. AI can help overcome language barriers and promote more inclusive communication among diverse groups.	1	2 ✓	3	4	5

CamScanner

No (28) & P(2)

4. There some cultural biases and stereotypes in AI-powered language tools and communication platforms.	1	2 ✓	3	4	5
5. AI has affected the localization and adaptation of content for different cultural audiences in digital communication.	1	2	3 ✓	4	5
6. I encountered any challenges in using AI-powered translation tools when communicating with people from different cultural backgrounds.	1	2 ✓	3	4	5
7. AI has the potential to promote cultural exchange and appreciation by enabling easier access to diverse perspectives and information.	1	2 ✓	3	4	5
8. AI-powered machines and chatbots should be programmed to respect and adapt to cultural norms and communication styles while interacting with users from different cultures.	1 ✓	2	3	4	5
9. There are some potential risks and concerns regarding AI's role in cross-cultural communication, such as perpetuating cultural stereotypes and misinterpretations.	1	2	3 ✓	4	5
10. I have participated in some cross-cultural virtual collaborations that relied on AI for communication and coordination.	1	2 ✓	3	4	5
11. I think AI can enhance intercultural learning experiences and foster empathy among individuals from different cultural backgrounds.	1	2 ✓	3	4	5
12. AI-powered virtual reality (VR) and augmented reality (AR) technologies can impact cross-cultural communication and understanding.	1	2 ✓	3	4	5
13. Somehow AI-generated content may unintentionally offend and misled individuals from specific cultural backgrounds.	1	2 ✓	3	4	5
14. Ethical considerations should be taken into account when designing AI-powered communication tools that cater to diverse cultural contexts.	1 ✓	2	3	4	5
15. Universities and educational institutions leverage AI to create more inclusive and culturally diverse online learning environments.	1	2	3 ✓	4	5

In the case of your agreement on sharing your answer/ points of views for questions below, please write down your comment/ point of view for all questions or any question that you prefer:

1. Did you experience the use of AI and AI-powered chatbots, such as ChatGPT and for what purpose?

Yes, for class to understand lecture's words.

2. How do you perceive the role of AI and AI-powered machines in bridging communication gaps between people from different cultures?

AI can as a tool to promote or help [illegible] interaction among people from

different cultural context, but it cannot substitute human beings

CamScanner

No (28) & P(3)

3. Have you experienced and observed any instances where AI has been successful in facilitating cross-cultural understanding and collaboration in mediated communication?

Not yet.

4. In what ways do you think AI can help overcome language barriers and promote more inclusive communication among diverse groups?

Translator

5. Are there any cultural biases or stereotypes that you have noticed in AI-powered language tools or communication platforms?

Yes. some specific proverbs or idioms AI cannot identified

6. How can universities and educational institutions leverage AI to create more inclusive and culturally diverse online learning environments?

May be I insert AI language tools into classroom App.

Thank you for your time and participation

CamScanner

Participant No (29) & P(1)

UNIVERSITI MALAYA

Depart of Media and Communication Studies

AI and Mediated Intercultural Communication Questionnaire

Dear UM Student/ Researcher,

Through this survey, we want to assess the probable effects of Artificial Intelligence (AI) on mediated communication among people from different cultures. This instrument which been developed by the help of the existing literature and ChatGPT, has 15 structured items with five (5) options per item and six (6) open-ended questions. Your participation in the survey is voluntary and the personal identifications of the participants will be treated as strictly confidential.

Thank you for your cooperation.

The researchers

A. Demographic Information

1. Gender: ☐ Male ☑ Female

2. Age: 28

3. Nationality: malaysia

4. Level of education: Postdtution (Post Graduation)

5. AI/ ChatGPT experience: ☑ Yes ☐ No

B. Intercultural Sensitivity Scale

**Directions:** The current AI-ICC questionnaire includes 15 items/ statements with five (5) options per item, as: 1 – Strongly agree; 2 – Agree, 3 – Uncertain; 4 – Disagree, and 5 – Strongly disagree.	Strongly agree	Agree	Uncertain	Disagree	Strongly disagree
1. AI and AI-powered machines can be helpful in bridging communication gaps between people from different cultures.	1	(2)	3	4	5
2. So far, AI has been successful in facilitating cross-cultural understanding and collaboration in mediated communication.	1	(2)	3	4	5
3. AI can help overcome language barriers and promote more inclusive communication among diverse groups.	1	(2)	3	4	5

CS CamScanner

No (29) E₁ P(2)

4. There some cultural biases and stereotypes in AI-powered language tools and communication platforms.	1	2	(3)	4	5
5. AI has affected the localization and adaptation of content for different cultural audiences in digital communication.	1	2	(3)	4	5
6. I encountered any challenges in using AI-powered translation tools when communicating with people from different cultural backgrounds.	1	2	3	(4)	5
7. AI has the potential to promote cultural exchange and appreciation by enabling easier access to diverse perspectives and information.	1	(2)	3	4	5
8. AI-powered machines and chatbots should be programmed to respect and adapt to cultural norms and communication styles while interacting with users from different cultures.	1	(2)	3	4	5
9. There are some potential risks and concerns regarding AI's role in cross-cultural communication, such as perpetuating cultural stereotypes and misinterpretations.	1	2	(3)	4	5
10. I have participated in some cross-cultural virtual collaborations that relied on AI for communication and coordination.	1	2	3	(4)	5
11. I think AI can enhance intercultural learning experiences and foster empathy among individuals from different cultural backgrounds.	1	(2)	3	4	5
12. AI-powered virtual reality (VR) and augmented reality (AR) technologies can impact cross-cultural communication and understanding.	1	2	(3)	4	5
13. Somehow AI-generated content may unintentionally offend and misled individuals from specific cultural backgrounds.	1	2	3	(4)	5
14. Ethical considerations should be taken into account when designing AI-powered communication tools that cater to diverse cultural contexts.	1	(2)	3	4	5
15. Universities and educational institutions leverage AI to create more inclusive and culturally diverse online learning environments.	1	(2)	3	4	5

In the case of your agreement on sharing your answer/ points of views for questions below, please write down your comment/ point of view for all questions or any question that you prefer:

1. Did you experience the use of AI and AI-powered chatbots, such as ChatGPT and for what purpose?

yes, for searching information for my studies

2. How do you perceive the role of AI and AI-powered machines in bridging communication gaps between people from different cultures?

The AI have lots of info and data that allow ~~other~~ people from different cultures learn about each other.

CamScanner

No (29) S P (3)

3. Have you experienced and observed any instances where AI has been successful in facilitating cross-cultural understanding and collaboration in mediated communication?

Yes i have

4. In what ways do you think AI can help overcome language barriers and promote more inclusive communication among diverse groups?

by translating for different languages

5. Are there any cultural biases or stereotypes that you have noticed in AI-powered language tools or communication platforms?

no

6. How can universities and educational institutions leverage AI to create more inclusive and culturally diverse online learning environments?

accommodating more computer or loptop with new update for students to use

Thank you for your time and participation

CamScanner

Participant No (30) & P(1)

UNIVERSITI MALAYA

Depart of Media and Communication Studies

AI and Mediated Intercultural Communication Questionnaire

Dear UM Student/ Researcher,

Through this survey, we want to assess the probable effects of Artificial Intelligence (AI) on mediated communication among people from different cultures. This instrument which been developed by the help of the existing literature and ChatGPT, has 15 structured items with five (5) options per item and six (6) open-ended questions. Your participation in the survey is voluntary and the personal identifications of the participants will be treated as strictly confidential.

Thank you for your cooperation.

The researchers

A. Demographic Information

1. Gender: ☑ Male ☐ Female
2. Age: 25
3. Nationality: CHINA
4. Level of education: MASTER
5. AI/ ChatGPT experience: ☑ Yes ☐ No

B. Intercultural Sensitivity Scale

**Directions:** The current AI-ICC questionnaire includes 15 items/ statements with five (5) options per item, as: 1 – Strongly agree; 2 – Agree, 3 – Uncertain; 4 – Disagree, and 5 – Strongly disagree.	Strongly agree	Agree	Uncertain	Disagree	Strongly disagree
1. AI and AI-powered machines can be helpful in bridging communication gaps between people from different cultures.	1	2 ✓	3	4	5
2. So far, AI has been successful in facilitating cross-cultural understanding and collaboration in mediated communication.	1	2	3	4 ✓	5
3. AI can help overcome language barriers and promote more inclusive communication among diverse groups.	1	2 ✓	3	4	5

CamScanner

No (30) & P (2)

4. There some cultural biases and stereotypes in AI-powered language tools and communication platforms.	1 ✓	2	3	4	5
5. AI has affected the localization and adaptation of content for different cultural audiences in digital communication.	1	2	3 ✓	4	5
6. I encountered any challenges in using AI-powered translation tools when communicating with people from different cultural backgrounds.	1	2 ✓	3	4	5
7. AI has the potential to promote cultural exchange and appreciation by enabling easier access to diverse perspectives and information.	1	2	3 ✓	4	5
8. AI-powered machines and chatbots should be programmed to respect and adapt to cultural norms and communication styles while interacting with users from different cultures.	1	2	3 ✓	4	5
9. There are some potential risks and concerns regarding AI's role in cross-cultural communication, such as perpetuating cultural stereotypes and misinterpretations.	1	2 ✓	3	4	5
10. I have participated in some cross-cultural virtual collaborations that relied on AI for communication and coordination.	1	2	3	4 ✓	5
11. I think AI can enhance intercultural learning experiences and foster empathy among individuals from different cultural backgrounds.	1	2	3 ✓	4	5
12. AI-powered virtual reality (VR) and augmented reality (AR) technologies can impact cross-cultural communication and understanding.	1	2	3 ✓	4	5
13. Somehow AI-generated content may unintentionally offend and misled individuals from specific cultural backgrounds.	1	2 ✓	3	4	5
14. Ethical considerations should be taken into account when designing AI-powered communication tools that cater to diverse cultural contexts.	1	2 ✓	3	4	5
15. Universities and educational institutions leverage AI to create more inclusive and culturally diverse online learning environments.	1	2 ✓	3	4	5

In the case of your agreement on sharing your answer/ points of views for questions below, please write down your comment/ point of view for all questions or any question that you prefer:

1. Did you experience the use of AI and AI-powered chatbots, such as ChatGPT and for what purpose?

   Yes. for ~~x~~ information search

2. How do you perceive the role of AI and AI-powered machines in bridging communication gaps between people from different cultures?

   I don't think AI can bridge our cultural gap due to stratospheric phenomenon.

No (30) [illegible] P(3)

3. Have you experienced and observed any instances where AI has been successful in facilitating cross-cultural understanding and collaboration in mediated communication?

No.

4. In what ways do you think AI can help overcome language barriers and promote more inclusive communication among diverse groups?

AI may provide some objective contents for diverse groups

5. Are there any cultural biases or stereotypes that you have noticed in AI-powered language tools or communication platforms?

Yes. Religon, ~~Rati~~ Race and politics are three main ~~senti~~ sensitive topics that different countries may have different bias, which result in different setup of AI machine ~~[illegible]~~ in different regions.

6. How can universities and educational institutions leverage AI to create more inclusive and culturally diverse online learning environments?

Thank you for your time and participation

CS CamScanner

Participant No (4) & P (1)
(31)

UNIVERSITI MALAYA

Depart of Media and Communication Studies

AI and Mediated Intercultural Communication Questionnaire

Dear UM Student/ Researcher,

Through this survey, we want to assess the probable effects of Artificial Intelligence (AI) on mediated communication among people from different cultures. This instrument which been developed by the help of the existing literature and ChatGPT, has 15 structured items with five (5) options per item and six (6) open-ended questions. Your participation in the survey is voluntary and the personal identifications of the participants will be treated as strictly confidential.

Thank you for your cooperation.

The researchers

A. Demographic Information

1. Gender: ☐ Male ☑ Female

2. Age: 29

3. Nationality: MALAY

4. Level of education: ~~BACHELOR'S DEGREE~~ Post-MA

5. AI/ ChatGPT experience: ☑ Yes ☐ No

B. Intercultural Sensitivity Scale

Directions: The current AI-ICC questionnaire includes 15 items/ statements with five (5) options per item, as: 1 – Strongly agree; 2 – Agree, 3 – Uncertain; 2 – Disagree, and 5 – Strongly disagree.	Strongly agree	Agree	Uncertain	Disagree	Strongly disagree
1. AI and AI-powered machines can be helpful in bridging communication gaps between people from different cultures.	(1)	2	3	4	5
2. So far, AI has been successful in facilitating cross-cultural understanding and collaboration in mediated communication.	(1)	2	3	4	5
3. AI can help overcome language barriers and promote more inclusive communication among diverse groups.	(1)	2	3	4	5

CS CamScanner

Participant No. [illegible] & P2

31

4. There some cultural biases and stereotypes in AI-powered language tools and communication platforms.	1	2	(3)	4	5
5. AI has affected the localization and adaptation of content for different cultural audiences in digital communication.	1	2	(3)	4	5
6. I encountered any challenges in using AI-powered translation tools when communicating with people from different cultural backgrounds.	1	2	3	4	(5)
7. AI has the potential to promote cultural exchange and appreciation by enabling easier access to diverse perspectives and information.	(1)	2	3	4	5
8. AI-powered machines and chatbots should be programmed to respect and adapt to cultural norms and communication styles while interacting with users from different cultures.	(1)	2	3	4	5
9. There are some potential risks and concerns regarding AI's role in cross-cultural communication, such as perpetuating cultural stereotypes and misinterpretations.	(1)	2	3	4	5
10. I have participated in some cross-cultural virtual collaborations that relied on AI for communication and coordination.	1	2	3	4	(5)
11. I think AI can enhance intercultural learning experiences and foster empathy among individuals from different cultural backgrounds.	(1)	2	3	4	5
12. AI-powered virtual reality (VR) and augmented reality (AR) technologies can impact cross-cultural communication and understanding.	1	2	(3)	4	5
13. Somehow AI-generated content may unintentionally offend and misled individuals from specific cultural backgrounds.	~~(1)~~	2	3	4	(5)
14. Ethical considerations should be taken into account when designing AI-powered communication tools that cater to diverse cultural contexts.	(1)	2	3	4	5
15. Universities and educational institutions leverage AI to create more inclusive and culturally diverse online learning environments.	(1)	2	3	4	5

In the case of your agreement on sharing your answer/ points of views for questions below, please write down your comment/ point of view for all questions or any question that you prefer:

1. Did you experience the use of AI and AI-powered chatbots, such as ChatGPT and for what purpose?

Yes. ~~For~~ To search for extra knowledge out from academic syllabus & gaining extra information from the topic related.

2. How do you perceive the role of AI and AI-powered machines in bridging communication gaps between people from different cultures?

The role of AI in bridging the communication gaps ~~or~~ is by providing a clear information regarding cultural knowledge especially if the information is related 2 with sensitive topics/issue.

Participant No (29) & (13)
(31)

3. Have you experienced and observed any instances where AI has been successful in facilitating cross-cultural understanding and collaboration in mediated communication?

NO.

4. In what ways do you think AI can help overcome language barriers and promote more inclusive communication among diverse groups?

AI can interpret the information into more simpler & understandable knowledge to make it easier for people from diverse groups to understand.

5. Are there any cultural biases or stereotypes that you have noticed in AI-powered language tools or communication platforms?

No.

6. How can universities and educational institutions leverage AI to create more inclusive and culturally diverse online learning environments?

Facilitate mutual understanding.

Thank you for your time and participation

CamScanner

Partcipant No (32) Ec P(1)

UNIVERSITI
MALAYA

Depart of Media and Communication Studies

AI and Mediated Intercultural Communication Questionnaire

Dear UM Student/ Researcher,

Through this survey, we want to assess the probable effects of Artificial Intelligence (AI) on mediated communication among people from different cultures. This instrument which been developed by the help of the existing literature and ChatGPT, has 15 structured items with five (5) options per item and six (6) open-ended questions. Your participation in the survey is voluntary and the personal identifications of the participants will be treated as strictly confidential.

Thank you for your cooperation.

The researchers

A. Demographic Information

1. Gender: ☐ Male ✓ Female

2. Age: 28

3. Nationality: MALAYSIAN

4. Level of education: POSTGDATION (Post graduation Degree)

5. AI/ ChatGPT experience: ✓ Yes ☐ No

B. Intercultural Sensitivity Scale

**Directions:** The current AI-ICC questionnaire includes 15 items/ statements with five (5) options per item, as: 1 – Strongly agree; 2 – Agree, 3 – Uncertain; 4 – Disagree, and 5 – Strongly disagree.	Strongly agree	Agree	Uncertain	Disagree	Strongly disagree
1. AI and AI-powered machines can be helpful in bridging communication gaps between people from different cultures.	(1)	2	3	4	5
2. So far, AI has been successful in facilitating cross-cultural understanding and collaboration in mediated communication.	(1)	2	3	4	5
3. AI can help overcome language barriers and promote more inclusive communication among diverse groups.	(1)	2	3	4	5

CS CamScanner

No-(32) & P(2)

4. There some cultural biases and stereotypes in AI-powered language tools and communication platforms.	1	(2)	3	4	5
5. AI has affected the localization and adaptation of content for different cultural audiences in digital communication.	1	(2)	3	4	5
6. I encountered any challenges in using AI-powered translation tools when communicating with people from different cultural backgrounds.	1	(2)	3	4	5
7. AI has the potential to promote cultural exchange and appreciation by enabling easier access to diverse perspectives and information.	1	(2)	3	4	5
8. AI-powered machines and chatbots should be programmed to respect and adapt to cultural norms and communication styles while interacting with users from different cultures.	(1)	2	3	4	5
9. There are some potential risks and concerns regarding AI's role in cross-cultural communication, such as perpetuating cultural stereotypes and misinterpretations.	(1)	2	3	4	5
10. I have participated in some cross-cultural virtual collaborations that relied on AI for communication and coordination.	1	2	3	(4)	5
11. I think AI can enhance intercultural learning experiences and foster empathy among individuals from different cultural backgrounds.	1	(2)	3	4	5
12. AI-powered virtual reality (VR) and augmented reality (AR) technologies can impact cross-cultural communication and understanding.	1	(2)	3	4	5
13. Somehow AI-generated content may unintentionally offend and misled individuals from specific cultural backgrounds.	1	(2)	3	4	5
14. Ethical considerations should be taken into account when designing AI-powered communication tools that cater to diverse cultural contexts.	(1)	2	3	4	5
15. Universities and educational institutions leverage AI to create more inclusive and culturally diverse online learning environments.	(1)	2	3	4	5

In the case of your agreement on sharing your answer/ points of views for questions below, please write down your comment/ point of view for all questions or any question that you prefer:

1. Did you experience the use of AI and AI-powered chatbots, such as ChatGPT and for what purpose?

   I have never experienced in using AI and AI-powered chatbots.

2. How do you perceive the role of AI and AI-powered machines in bridging communication gaps between people from different cultures?

   AI and AI-powered machines help with having easier to talk with people from different countries with different language.

CS CamScanner

No 62 & P3

3. Have you experienced and observed any instances where AI has been successful in facilitating cross-cultural understanding and collaboration in mediated communication?

No, I had never experience it.

4. In what ways do you think AI can help overcome language barriers and promote more inclusive communication among diverse groups?

AI can ~~undes~~ understand multiple languages at the same time.

5. Are there any cultural biases or stereotypes that you have noticed in AI-powered language tools or communication platforms?

No, ~~at this time~~ as far as I know

6. How can universities and educational institutions leverage AI to create more inclusive and culturally diverse online learning environments?

universities and educational institutions use more AI-based ~~in~~ apps in their daily activities

Thank you for your time and participation

CamScanner

Participant No (33) & P(1)

UNIVERSITI MALAYA

Depart of Media and Communication Studies

AI and Mediated Intercultural Communication Questionnaire

Dear UM Student/ Researcher,

Through this survey, we want to assess the probable effects of Artificial Intelligence (AI) on mediated communication among people from different cultures. This instrument which been developed by the help of the existing literature and ChatGPT, has 15 structured items with five (5) options per item and six (6) open-ended questions. Your participation in the survey is voluntary and the personal identifications of the participants will be treated as strictly confidential.

Thank you for your cooperation.

The researchers

A. Demographic Information

1. Gender: ☐ Male ☒ Female

2. Age: 28

3. Nationality: MALAYSIAN

4. Level of education: ~~[illegible]~~ post grade degree IN SOCIAL SCIENCE

5. AI/ ChatGPT experience: ☒ Yes ☐ No

B. Intercultural Sensitivity Scale

**Directions:** The current AI-ICC questionnaire includes 15 items/ statements with five (5) options per item, as: 1 – Strongly agree; 2 – Agree, 3 – Uncertain; 4 – Disagree, and 5 – Strongly disagree.	Strongly agree	Agree	Uncertain	Disagree	Strongly disagree
1. AI and AI-powered machines can be helpful in bridging communication gaps between people from different cultures.	1	(2)	3	4	5
2. So far, AI has been successful in facilitating cross-cultural understanding and collaboration in mediated communication.	1	2	3	(4)	5
3. AI can help overcome language barriers and promote more inclusive communication among diverse groups.	1	(2)	3	4	5

CS CamScanner

No (33) E, P(2)

4. There some cultural biases and stereotypes in AI-powered language tools and communication platforms.	1	2	(3)	4	5
5. AI has affected the localization and adaptation of content for different cultural audiences in digital communication.	1	2	(3)	4	5
6. I encountered any challenges in using AI-powered translation tools when communicating with people from different cultural backgrounds.	1	2	3	(4)	5
7. AI has the potential to promote cultural exchange and appreciation by enabling easier access to diverse perspectives and information.	1	(2)	3	4	5
8. AI-powered machines and chatbots should be programmed to respect and adapt to cultural norms and communication styles while interacting with users from different cultures.	(1)	2	3	4	5
9. There are some potential risks and concerns regarding AI's role in cross-cultural communication, such as perpetuating cultural stereotypes and misinterpretations.	(1)	2	3	4	5
10. I have participated in some cross-cultural virtual collaborations that relied on AI for communication and coordination.	1	2	3	(4)	5
11. I think AI can enhance intercultural learning experiences and foster empathy among individuals from different cultural backgrounds.	1	(2)	3	4	5
12. AI-powered virtual reality (VR) and augmented reality (AR) technologies can impact cross-cultural communication and understanding.	(1)	2	3	4	5
13. Somehow AI-generated content may unintentionally offend and misled individuals from specific cultural backgrounds.	(1)	2	3	4	5
14. Ethical considerations should be taken into account when designing AI-powered communication tools that cater to diverse cultural contexts.	(1)	2	3	4	5
15. Universities and educational institutions leverage AI to create more inclusive and culturally diverse online learning environments.	(1)	2	3	4	5

In the case of your agreement on sharing your answer/ points of views for questions below, please write down your comment/ point of view for all questions or any question that you prefer:

1. Did you experience the use of AI and AI-powered chatbots, such as ChatGPT and for what purpose?

   I have experience using a AI & AI-powered chatbots. For research purposes and also in my study.

2. How do you perceive the role of AI and AI-powered machines in bridging communication gaps between people from different cultures?

   Some information can ~~bet~~ be taken out of context or mistaken as the AI get information from the internet primarily.

CS CamScanner

No (33) E P(3)

3. Have you experienced and observed any instances where AI has been successful in facilitating cross-cultural understanding and collaboration in mediated communication?

I have not.

4. In what ways do you think AI can help overcome language barriers and promote more inclusive communication among diverse groups?

AI can directly translates ~~on~~ languages with access to the Internet. ~~Ho~~ However, inclusiveness may be hard to reach as miscommunication occur.

5. Are there any cultural biases or stereotypes that you have noticed in AI-powered language tools or communication platforms?

As the AI based on the Internet, AI-powered language tools ~~can~~ may have a more western outlook on occasion as the media is largely controlled by them.

6. How can universities and educational institutions leverage AI to create more inclusive and culturally diverse online learning environments?

Enabling students to utilize AI for their work to get a more comphrehensive and wider look for a task.

Thank you for your time and participation

CamScanner

Participant No (34) E P(1)

UNIVERSITI
MALAYA

Depart of Media and Communication Studies

AI and Mediated Intercultural Communication Questionnaire

Dear UM Student/ Researcher,

Through this survey, we want to assess the probable effects of Artificial Intelligence (AI) on mediated communication among people from different cultures. This instrument which been developed by the help of the existing literature and ChatGPT, has 15 structured items with five (5) options per item and six (6) open-ended questions. Your participation in the survey is voluntary and the personal identifications of the participants will be treated as strictly confidential.

Thank you for your cooperation.

The researchers

A. Demographic Information

1. Gender: ✓ Male ☐ Female

2. Age: 28

3. Nationality: MALAYSIAN

4. Level of education: Postgraduate

5. AI/ ChatGPT experience: ✓ Yes ✓ No

B. Intercultural Sensitivity Scale

Directions: The current AI-ICC questionnaire includes 15 items/ statements with five (5) options per item, as: 1 – Strongly agree; 2 – Agree, 3 – Uncertain; 4 – Disagree, and 5 – Strongly disagree.	Strongly agree	Agree	Uncertain	Disagree	Strongly disagree
1. AI and AI-powered machines can be helpful in bridging communication gaps between people from different cultures.	✓	2	3	4	5
2. So far, AI has been successful in facilitating cross-cultural understanding and collaboration in mediated communication.	1	✓	3	4	5
3. AI can help overcome language barriers and promote more inclusive communication among diverse groups.	✓	2	3	4	5

CamScanner

No (34) E P(2)

4. There some cultural biases and stereotypes in AI-powered language tools and communication platforms.	1	2 ✓	3	4	5
5. AI has affected the localization and adaptation of content for different cultural audiences in digital communication.	1	2 ✓	3	4	5
6. I encountered any challenges in using AI-powered translation tools when communicating with people from different cultural backgrounds.	1	2 ✓	3	4	5
7. AI has the potential to promote cultural exchange and appreciation by enabling easier access to diverse perspectives and information.	1	2 ✓	3	4	5
8. AI-powered machines and chatbots should be programmed to respect and adapt to cultural norms and communication styles while interacting with users from different cultures.	1	2 ✓	3	4	5
9. There are some potential risks and concerns regarding AI's role in cross-cultural communication, such as perpetuating cultural stereotypes and misinterpretations.	1	2	3 ✓	4	5
10. I have participated in some cross-cultural virtual collaborations that relied on AI for communication and coordination.	1 ✓	2	3	4	5
11. I think AI can enhance intercultural learning experiences and foster empathy among individuals from different cultural backgrounds.	1	2 ✓	3	4	5
12. AI-powered virtual reality (VR) and augmented reality (AR) technologies can impact cross-cultural communication and understanding.	1	2 ✓	3	4	5
13. Somehow AI-generated content may unintentionally offend and misled individuals from specific cultural backgrounds.	1	2 ✓	3	4	5
14. Ethical considerations should be taken into account when designing AI-powered communication tools that cater to diverse cultural contexts.	1	2 ✓	3	4	5
15. Universities and educational institutions leverage AI to create more inclusive and culturally diverse online learning environments.	1 ✓	2	3	4	5

In the case of your agreement on sharing your answer/ points of views for questions below, please write down your comment/ point of view for all questions or any question that you prefer:

1. Did you experience the use of AI and AI-powered chatbots, such as ChatGPT and for what purpose?
   No, I haven't use any AI and AI-powered chatbots before.

2. How do you perceive the role of AI and AI-powered machines in bridging communication gaps between people from different cultures?
   Easier

CamScanner

No-(34) & P(3)

3. Have you experienced and observed any instances where AI has been successful in facilitating cross-cultural understanding and collaboration in mediated communication?

No.

4. In what ways do you think AI can help overcome language barriers and promote more inclusive communication among diverse groups?

~~This~~ These days, there is an apps that can translate a language t another language.

5. Are there any cultural biases or stereotypes that you have noticed in AI-powered language tools or communication platforms?

No.

6. How can universities and educational institutions leverage AI to create more inclusive and culturally diverse online learning environments?

Online quiz or online classes or using more AI based apps.

Thank you for your time and participation

CamScanner

/

UNIVERSITI MALAYA

Depart of Media and Communication Studies

AI and Mediated Intercultural Communication Questionnaire

Dear UM Student/ Researcher,

Through this survey, we want to assess the probable effects of Artificial Intelligence (AI) on mediated communication among people from different cultures. This instrument which been developed by the help of the existing literature and ChatGPT, has 15 structured items with five (5) options per item and six (6) open-ended questions. Your participation in the survey is voluntary and the personal identifications of the participants will be treated as strictly confidential.

Thank you for your cooperation.

The researchers

A. Demographic Information

1. Gender: ☐ Male ☑ Female

2. Age: 35

3. Nationality: Chinese

4. Level of education: P.hD.

5. AI/ ChatGPT experience: ☑ Yes ☐ No

B. Intercultural Sensitivity Scale

Directions: The current AI-ICC questionnaire includes 15 items/ statements with five (5) options per item, as: 1 – Strongly agree; 2 – Agree, 3 – Uncertain; 4 – Disagree, and 5 – Strongly disagree.	Strongly agree	Agree	Uncertain	Disagree	Strongly disagree
1. AI and AI-powered machines can be helpful in bridging communication gaps between people from different cultures.	(1)	2	3	4	5
2. So far, AI has been successful in facilitating cross-cultural understanding and collaboration in mediated communication.	1	(2)	3	4	5
3. AI can help overcome language barriers and promote more inclusive communication among diverse groups.	(1)	2	3	4	5

Participant No (35) E Page (1)

CamScanner

4. There some cultural biases and stereotypes in AI-powered language tools and communication platforms.	1	2	3	(4)	5
5. AI has affected the localization and adaptation of content for different cultural audiences in digital communication.	1	2	3	(4)	5
6. I encountered any challenges in using AI-powered translation tools when communicating with people from different cultural backgrounds.	1	2	3	(4)	5
7. AI has the potential to promote cultural exchange and appreciation by enabling easier access to diverse perspectives and information.	1	(2)	3	4	5
8. AI-powered machines and chatbots should be programmed to respect and adapt to cultural norms and communication styles while interacting with users from different cultures.	1	(2)	3	4	5
9. There are some potential risks and concerns regarding AI's role in cross-cultural communication, such as perpetuating cultural stereotypes and misinterpretations.	1	(2)	3	4	5
10. I have participated in some cross-cultural virtual collaborations that relied on AI for communication and coordination.	1	2	(3)	4	5
11. I think AI can enhance intercultural learning experiences and foster empathy among individuals from different cultural backgrounds.	1	(2)	3	4	5
12. AI-powered virtual reality (VR) and augmented reality (AR) technologies can impact cross-cultural communication and understanding.	1	(2)	3	4	5
13. Somehow AI-generated content may unintentionally offend and misled individuals from specific cultural backgrounds.	1	2	(3)	4	5
14. Ethical considerations should be taken into account when designing AI-powered communication tools that cater to diverse cultural contexts.	(1)	2	3	4	5
15. Universities and educational institutions leverage AI to create more inclusive and culturally diverse online learning environments.	1	(2)	3	4	5

In the case of your agreement on sharing your answer/ points of views for questions below, please write down your comment/ point of view for all questions or any question that you prefer:

1. Did you experience the use of AI and AI-powered chatbots, such as ChatGPT and for what purpose?

Yes. I use Chat-GPT(4) for English writing promotion and Coding Language learning, such as R, Python, which is so helpful to guide me solve challenging problems.

2. How do you perceive the role of AI and AI-powered machines in bridging communication gaps between people from different cultures?

In my mind. AI machines play a significant role in translating different languages, removing the misunderstanding of some challenging and ~~hard~~ complexible sentences.

Participant No(5) E, Page(2)

CamScanner

3. Have you experienced and observed any instances where AI has been successful in facilitating cross-cultural understanding and collaboration in mediated communication?

No.

4. In what ways do you think AI can help overcome language barriers and promote more inclusive communication among diverse groups?

For some professional academic ~~English~~ learning, AI can help overcome language barriers by providing very detailed and easy-understanding explanation.

5. Are there any cultural biases or stereotypes that you have noticed in AI-powered language tools or communication platforms?

No.

6. How can universities and educational institutions leverage AI to create more inclusive and culturally diverse online learning environments?

1. First, they can promote and provide more educational courses on how to use AI properly.

Thank you for your time and participation

2. Second, they can hold some activities to encourage students to ~~use AI properly~~ get touch with AI. and have deep concept on using AI in proper and correct way.

Participant No (35) & P(3)

CamScanner

Partecipant No (36) & P(1)

UNIVERSITI MALAYA

Depart of Media and Communication Studies

AI and Mediated Intercultural Communication Questionnaire

Dear UM Student/ Researcher,

Through this survey, we want to assess the probable effects of Artificial Intelligence (AI) on mediated communication among people from different cultures. This instrument which been developed by the help of the existing literature and ChatGPT, has 15 structured items with five (5) options per item and six (6) open-ended questions. Your participation in the survey is voluntary and the personal identifications of the participants will be treated as strictly confidential.

Thank you for your cooperation.

The researchers

A. Demographic Information

1. Gender: ☑ Male ☐ Female

2. Age: 22

3. Nationality: MALAYSIAN

4. Level of education: ~~BACHELOR~~ Post-Graduate degree

5. AI/ ChatGPT experience: ☑ Yes ☐ No

B. Intercultural Sensitivity Scale

**Directions:** The current AI-ICC questionnaire includes 15 items/ statements with five (5) options per item, as: 1 – Strongly agree; 2 – Agree, 3 – Uncertain; 4 – Disagree, and 5 – Strongly disagree.	Strongly agree	Agree	Uncertain	Disagree	Strongly disagree
1. AI and AI-powered machines can be helpful in bridging communication gaps between people from different cultures.	1	2	3	4 ✓	5
2. So far, AI has been successful in facilitating cross-cultural understanding and collaboration in mediated communication.	1	2	3	4 ✓	5
3. AI can help overcome language barriers and promote more inclusive communication among diverse groups.	1	2	3	4 ✓	5

CamScanner

No (36) & P(2)

4. There some cultural biases and stereotypes in AI-powered language tools and communication platforms.	1	2	3	4 ✓	5
5. AI has affected the localization and adaptation of content for different cultural audiences in digital communication.	1	2	3	4	5 ✓
6. I encountered any challenges in using AI-powered translation tools when communicating with people from different cultural backgrounds.	1	2	3 ✓	4	5
7. AI has the potential to promote cultural exchange and appreciation by enabling easier access to diverse perspectives and information.	1	2 ✓	3	4	5
8. AI-powered machines and chatbots should be programmed to respect and adapt to cultural norms and communication styles while interacting with users from different cultures.	1	2	3	4	5 ✓
9. There are some potential risks and concerns regarding AI's role in cross-cultural communication, such as perpetuating cultural stereotypes and misinterpretations.	1	2	3	4	5 ✓
10. I have participated in some cross-cultural virtual collaborations that relied on AI for communication and coordination.	1 ✓	2	3	4	5
11. I think AI can enhance intercultural learning experiences and foster empathy among individuals from different cultural backgrounds.	1	2	3 ✓	4	5
12. AI-powered virtual reality (VR) and augmented reality (AR) technologies can impact cross-cultural communication and understanding.	1	2	3	4	5 ✓
13. Somehow AI-generated content may unintentionally offend and misled individuals from specific cultural backgrounds.	1	2	3	4	5 ✓
14. Ethical considerations should be taken into account when designing AI-powered communication tools that cater to diverse cultural contexts.	1	2	3	4	5 ✓
15. Universities and educational institutions leverage AI to create more inclusive and culturally diverse online learning environments.	1	2	3	4	5 ✓

In the case of your agreement on sharing your answer/ points of views for questions below, please write down your comment/ point of view for all questions or any question that you prefer:

1. Did you experience the use of AI and AI-powered chatbots, such as ChatGPT and for what purpose?

yes, writing letter and assignment purpose but not solely copy and paste

2. How do you perceive the role of AI and AI-powered machines in bridging communication gaps between people from different cultures?

I do not think different cultures will be a gap given that AI has 2 comprehensive knowledge

CS CamScanner

No (36) & P(3)

3. Have you experienced and observed any instances where AI has been successful in facilitating cross-cultural understanding and collaboration in mediated communication?

4. In what ways do you think AI can help overcome language barriers and promote more inclusive communication among diverse groups?

5. Are there any cultural biases or stereotypes that you have noticed in AI-powered language tools or communication platforms?

6. How can universities and educational institutions leverage AI to create more inclusive and culturally diverse online learning environments?

Thank you for your time and participation

CamScanner

Participant No (37) Ec P(1)

UNIVERSITI MALAYA

Depart of Media and Communication Studies

AI and Mediated Intercultural Communication Questionnaire

Dear UM Student/ Researcher,

Through this survey, we want to assess the probable effects of Artificial Intelligence (AI) on mediated communication among people from different cultures. This instrument which been developed by the help of the existing literature and ChatGPT, has 15 structured items with five (5) options per item and six (6) open-ended questions. Your participation in the survey is voluntary and the personal identifications of the participants will be treated as strictly confidential.

Thank you for your cooperation.

The researchers

A. Demographic Information

1. Gender: ☐ Male ☑ Female

2. Age: 27

3. Nationality: Malaysian

4. Level of education: ~~Post Bachelor degree~~ (Post Graduate)

5. AI/ ChatGPT experience: ☑ Yes ☐ No

B. Intercultural Sensitivity Scale

Directions: The current AI-ICC questionnaire includes 15 items/ statements with five (5) options per item, as: 1 – Strongly agree; 2 – Agree, 3 – Uncertain; 4 – Disagree, and 5 – Strongly disagree.	Strongly agree	Agree	Uncertain	Disagree	Strongly disagree
1. AI and AI-powered machines can be helpful in bridging communication gaps between people from different cultures.	1 ✓	2	3	4	5
2. So far, AI has been successful in facilitating cross-cultural understanding and collaboration in mediated communication.	1	2 ✓	3	4	5
3. AI can help overcome language barriers and promote more inclusive communication among diverse groups.	1 ✓	2	3	4	5

CamScanner

No (37) & P(2)

4. There some cultural biases and stereotypes in AI-powered language tools and communication platforms.	1	2	3 ✓	4	5
5. AI has affected the localization and adaptation of content for different cultural audiences in digital communication.	1	2 ✓	3	4	5
6. I encountered any challenges in using AI-powered translation tools when communicating with people from different cultural backgrounds.	1	2 ✓	3	4	5
7. AI has the potential to promote cultural exchange and appreciation by enabling easier access to diverse perspectives and information.	1	2 ✓	3	4	5
8. AI-powered machines and chatbots should be programmed to respect and adapt to cultural norms and communication styles while interacting with users from different cultures.	1 ✓	2	3	4	5
9. There are some potential risks and concerns regarding AI's role in cross-cultural communication, such as perpetuating cultural stereotypes and misinterpretations.	1	2	3 ✓	4	5
10. I have participated in some cross-cultural virtual collaborations that relied on AI for communication and coordination.	1	2	3	4 ✓	5
11. I think AI can enhance intercultural learning experiences and foster empathy among individuals from different cultural backgrounds.	1	2 ✓	3	4	5
12. AI-powered virtual reality (VR) and augmented reality (AR) technologies can impact cross-cultural communication and understanding.	1	2 ✓	3	4	5
13. Somehow AI-generated content may unintentionally offend and misled individuals from specific cultural backgrounds.	1 ✓	2	3	4	5
14. Ethical considerations should be taken into account when designing AI-powered communication tools that cater to diverse cultural contexts.	1 ✓	2	3	4	5
15. Universities and educational institutions leverage AI to create more inclusive and culturally diverse online learning environments.	1	2 ✓	3	4	5

In the case of your agreement on sharing your answer/ points of views for questions below, please write down your comment/ point of view for all questions or any question that you prefer:

1. Did you experience the use of AI and AI-powered chatbots, such as ChatGPT and for what purpose?

   Yes. To simplify ~~[illegible]~~ research articles.

2. How do you perceive the role of AI and AI-powered machines in bridging communication gaps between people from different cultures?

   Very helpful.

CamScanner

No (37) E P(3)

3. Have you experienced and observed any instances where AI has been successful in facilitating cross-cultural understanding and collaboration in mediated communication?

Yes. People with different backgrounds can experience each other's cultures through the result generated by AI

4. In what ways do you think AI can help overcome language barriers and promote more inclusive communication among diverse groups?

AI should be more well-promoted to reach every level of society.

5. Are there any cultural biases or stereotypes that you have noticed in AI-powered language tools or communication platforms?

AI being used ~~by~~ for stupid contents in media socials.

6. How can universities and educational institutions leverage AI to create more inclusive and culturally diverse online learning environments?

- AI should be introduced to students in a fun way
- Put the device at centres where students can gain access easily.

Thank you for your time and participation

CS CamScanner

Partcipant No (38) & P(1)

UNIVERSITI MALAYA

Depart of Media and Communication Studies

AI and Mediated Intercultural Communication Questionnaire

Dear UM Student/ Researcher,

Through this survey, we want to assess the probable effects of Artificial Intelligence (AI) on mediated communication among people from different cultures. This instrument which been developed by the help of the existing literature and ChatGPT, has 15 structured items with five (5) options per item and six (6) open-ended questions. Your participation in the survey is voluntary and the personal identifications of the participants will be treated as strictly confidential.

Thank you for your cooperation.

The researchers

A. Demographic Information

1. Gender: ☐ Male ☑ Female

2. Age: 26

3. Nationality: Chinese

4. Level of education: ~~master~~ postgraduate student

5. AI/ ChatGPT experience: ☑ Yes ☐ No

B. Intercultural Sensitivity Scale

**Directions:** The current AI-ICC questionnaire includes 15 items/ statements with five (5) options per item, as: 1 – Strongly agree; 2 – Agree, 3 – Uncertain; 4 – Disagree, and 5 – Strongly disagree.	Strongly agree	Agree	Uncertain	Disagree	Strongly disagree
**1. AI and AI-powered machines can be helpful in bridging communication gaps between people from different cultures.**	1	2 ✓	3	4	5
**2. So far, AI has been successful in facilitating cross-cultural understanding and collaboration in mediated communication.**	1	2 ✓	3	4	5
**3. AI can help overcome language barriers and promote more inclusive communication among diverse groups.**	1	2 ✓	3	4	5

CamScanner

Partecipant No 38 E PG2

4. There some cultural biases and stereotypes in AI-powered language tools and communication platforms.	1	2 ✓	3	4	5
5. AI has affected the localization and adaptation of content for different cultural audiences in digital communication.	1	2	3 ✓	4	5
6. I encountered any challenges in using AI-powered translation tools when communicating with people from different cultural backgrounds.	1	2	3	4 ✓	5
7. AI has the potential to promote cultural exchange and appreciation by enabling easier access to diverse perspectives and information.	1	2	3 ✓	4	5
8. AI-powered machines and chatbots should be programmed to respect and adapt to cultural norms and communication styles while interacting with users from different cultures.	1	2 ✓	3	4	5
9. There are some potential risks and concerns regarding AI's role in cross-cultural communication, such as perpetuating cultural stereotypes and misinterpretations.	1 ✓	2	3	4	5
10. I have participated in some cross-cultural virtual collaborations that relied on AI for communication and coordination.	1	2	3	4 ✓	5
11. I think AI can enhance intercultural learning experiences and foster empathy among individuals from different cultural backgrounds.	1	2	3	4 ✓	5
12. AI-powered virtual reality (VR) and augmented reality (AR) technologies can impact cross-cultural communication and understanding.	1	2	3 ✓	4	5
13. Somehow AI-generated content may unintentionally offend and misled individuals from specific cultural backgrounds.	1	2 ✓	3	4	5
14. Ethical considerations should be taken into account when designing AI-powered communication tools that cater to diverse cultural contexts.	1 ✓	2	3	4	5
15. Universities and educational institutions leverage AI to create more inclusive and culturally diverse online learning environments.	1	2 ✓	3	4	5

In the case of your agreement on sharing your answer/ points of views for questions below, please write down your comment/ point of view for all questions or any question that you prefer:

1. Did you experience the use of AI and AI-powered chatbots, such as ChatGPT and for what purpose?

   Yes. using ChatGPT for searching some question about dissertation, or using it like a wiki

2. How do you perceive the role of AI and AI-powered machines in bridging communication gaps between people from different cultures?

   I think the pros ~~w~~ outweigh the disadvantages. It's more easier ~~than~~ to communication withe different peoples.

CS CamScanner

No (38) & P(3)

3. Have you experienced and observed any instances where AI has been successful in facilitating cross-cultural understanding and collaboration in mediated communication?

No

4. In what ways do you think AI can help overcome language barriers and promote more inclusive communication among diverse groups?

Can translate the language to other person.

5. Are there any cultural biases or stereotypes that you have noticed in AI-powered language tools or communication platforms?

~~I think it's can't foll~~

I don't think it can express information very accurately.

6. How can universities and educational institutions leverage AI to create more inclusive and culturally diverse online learning environments?

Could accept to use it.

Thank you for your time and participation

CS CamScanner

Participant No [illegible] & [illegible]
(39)

UNIVERSITI
MALAYA

Depart of Media and Communication Studies

AI and Mediated Intercultural Communication Questionnaire

Dear UM Student/ Researcher,

Through this survey, we want to assess the probable effects of Artificial Intelligence (AI) on mediated communication among people from different cultures. This instrument which been developed by the help of the existing literature and ChatGPT, has 15 structured items with five (5) options per item and six (6) open-ended questions. Your participation in the survey is voluntary and the personal identifications of the participants will be treated as strictly confidential.

Thank you for your cooperation.

The researchers

A. Demographic Information

1. Gender: ☑ Male ☐ Female
2. Age: 40
3. Nationality: IRAN
4. Level of education: Master
5. AI/ ChatGPT experience: ☑ Yes ☐ No

B. Intercultural Sensitivity Scale

**Directions:** The current AI-ICC questionnaire includes 15 items/ statements with five (5) options per item, as: 1 – Strongly agree; 2 – Agree, 3 – Uncertain; 2 – Disagree, and 5 – Strongly disagree.	Strongly agree	Agree	Uncertain	Disagree	Strongly disagree
1. AI and AI-powered machines can be helpful in bridging communication gaps between people from different cultures.	1	2 ✓	3	4	5
2. So far, AI has been successful in facilitating cross-cultural understanding and collaboration in mediated communication.	1	2 ✓	3	4	5
3. AI can help overcome language barriers and promote more inclusive communication among diverse groups.	1	2 ✓	3	4	5

CamScanner

(39)

Participant No [illegible]

4. There some cultural biases and stereotypes in AI-powered language tools and communication platforms.	1	2 ✓	3	4	5
5. AI has affected the localization and adaptation of content for different cultural audiences in digital communication.	1	2 ✓	3	4	5
6. I encountered any challenges in using AI-powered translation tools when communicating with people from different cultural backgrounds.	1 ✓	2	3	4	5
7. AI has the potential to promote cultural exchange and appreciation by enabling easier access to diverse perspectives and information.	1 ✓	2	3	4	5
8. AI-powered machines and chatbots should be programmed to respect and adapt to cultural norms and communication styles while interacting with users from different cultures.	1 ✓	2	3	4	5
9. There are some potential risks and concerns regarding AI's role in cross-cultural communication, such as perpetuating cultural stereotypes and misinterpretations.	1 ✓	2	3	4	5
10. I have participated in some cross-cultural virtual collaborations that relied on AI for communication and coordination.	1 ✓	2	3	4	5
11. I think AI can enhance intercultural learning experiences and foster empathy among individuals from different cultural backgrounds.	1 ✓	2	3	4	5
12. AI-powered virtual reality (VR) and augmented reality (AR) technologies can impact cross-cultural communication and understanding.	1 ✓	2	3	4	5
13. Somehow AI-generated content may unintentionally offend and misled individuals from specific cultural backgrounds.	1 ✓	2	3	4	5
14. Ethical considerations should be taken into account when designing AI-powered communication tools that cater to diverse cultural contexts.	1 ✓	2	3	4	5
15. Universities and educational institutions leverage AI to create more inclusive and culturally diverse online learning environments.	1 ✓	2	3	4	5

In the case of your agreement on sharing your answer/ points of views for questions below, please write down your comment/ point of view for all questions or any question that you prefer:

1. Did you experience the use of AI and AI-powered chatbots, such as ChatGPT and for what purpose?

yes, Education purpose, Research purpose

2. How do you perceive the role of AI and AI-powered machines in bridging communication gaps between people from different cultures?

It provide answer for any language. this is sth that a lecturer or teacher can not do.

CS CamScanner

Participant No. [illegible] & P(3)
(38)

3. Have you experienced and observed any instances where AI has been successful in facilitating cross-cultural understanding and collaboration in mediated communication?

No,

4. In what ways do you think AI can help overcome language barriers and promote more inclusive communication among diverse groups? In any area. but it is important to provide more safisticated tools, that are user friendly.

5. Are there any cultural biases or stereotypes that you have noticed in AI-powered language tools or communication platforms?

No, It is a Technological tool like smart phone & labtop and it is welcomed by any culture.

6. How can universities and educational institutions leverage AI to create more inclusive and culturally diverse online learning environments?

Use AI to translate content like website, slides, book, ... etc.

Thank you for your time and participation

Translate online lecturer with subtitle.

CS CamScanner

Participant No 9 & P1
(10)

UNIVERSITI
MALAYA

Depart of Media and Communication Studies

AI and Mediated Intercultural Communication Questionnaire

Dear UM Student/ Researcher,

Through this survey, we want to assess the probable effects of Artificial Intelligence (AI) on mediated communication among people from different cultures. This instrument which been developed by the help of the existing literature and ChatGPT, has 15 structured items with five (5) options per item and six (6) open-ended questions. Your participation in the survey is voluntary and the personal identifications of the participants will be treated as strictly confidential.

Thank you for your cooperation.

The researchers

A. Demographic Information

1. Gender: ☑ Male ☐ Female

2. Age: 29

3. Nationality: China

4. Level of education: PhD student

5. AI/ ChatGPT experience: ☑ Yes ☐ No

B. Intercultural Sensitivity Scale

**Directions:** The current AI-ICC questionnaire includes 15 items/ statements with five (5) options per item, as: 1 – Strongly agree; 2 – Agree, 3 – Uncertain; 2 – Disagree, and 5 – Strongly disagree.	Strongly agree	Agree	Uncertain	Disagree	Strongly disagree
1. AI and AI-powered machines can be helpful in bridging communication gaps between people from different cultures.	1 ✓	2	3	4	5
2. So far, AI has been successful in facilitating cross-cultural understanding and collaboration in mediated communication.	1	2	3 ✓	4	5
3. AI can help overcome language barriers and promote more inclusive communication among diverse groups.	1	2 ✓	3	4	5

CamScanner

Participant No [illegible] & P(2)
(40)

4. There some cultural biases and stereotypes in AI-powered language tools and communication platforms.	1	2	3	4 ✓	5
5. AI has affected the localization and adaptation of content for different cultural audiences in digital communication.	1	2 ✓	3	4	5
6. I encountered any challenges in using AI-powered translation tools when communicating with people from different cultural backgrounds.	1	2	3	4 ✓	5
7. AI has the potential to promote cultural exchange and appreciation by enabling easier access to diverse perspectives and information.	1	2 ✓	3	4	5
8. AI-powered machines and chatbots should be programmed to respect and adapt to cultural norms and communication styles while interacting with users from different cultures.	1 ✓	2	3	4	5
9. There are some potential risks and concerns regarding AI's role in cross-cultural communication, such as perpetuating cultural stereotypes and misinterpretations.	1	2	3 ✓	4	5
10. I have participated in some cross-cultural virtual collaborations that relied on AI for communication and coordination.	1	2	3	4 ✓	5
11. I think AI can enhance intercultural learning experiences and foster empathy among individuals from different cultural backgrounds.	1 ✓	2	3	4	5
12. AI-powered virtual reality (VR) and augmented reality (AR) technologies can impact cross-cultural communication and understanding.	1 ✓	2	3	4	5
13. Somehow AI-generated content may unintentionally offend and misled individuals from specific cultural backgrounds.	1	2 ✓	3	4	5
14. Ethical considerations should be taken into account when designing AI-powered communication tools that cater to diverse cultural contexts.	1 ✓	2	3	4	5
15. Universities and educational institutions leverage AI to create more inclusive and culturally diverse online learning environments.	1 ✓	2	3	4	5

In the case of your agreement on sharing your answer/ points of views for questions below, please write down your comment/ point of view for all questions or any question that you prefer:

1. Did you experience the use of AI and AI-powered chatbots, such as ChatGPT and for what purpose?

   Yes. For information seeking and sometimes there is no one can chat with me. I will use ChatGPT to killing time and being chilled. And ask for companion.

2. How do you perceive the role of AI and AI-powered machines in bridging communication gaps between people from different cultures?

   Firstly. It is the way to know more and better about other culture from AI. Such as some features and taboos. Secondly. I can also ask AI about what I need to pay attention based on my culture.

CS CamScanner

Participant No [illegible] E P(3) (40)

3. Have you experienced and observed any instances where AI has been successful in facilitating cross-cultural understanding and collaboration in mediated communication?

I'm a Chinese with no religon. So before I came to Malaysia, I use Internet and AI to tell me what I need to do and should not to do. Especially communicationg with local people.

4. In what ways do you think AI can help overcome language barriers and promote more inclusive communication among diverse groups?

AI can give me specific advice and explanation what I can not understand from other culture.

5. Are there any cultural biases or stereotypes that you have noticed in AI-powered language tools or communication platforms?

Not yet. In my view. AI with diverse and ~~common~~ normal value better than I am.

6. How can universities and educational institutions leverage AI to create more inclusive and culturally diverse online learning environments?

In technique way, improve AI's logical problems. And also moral consideration. making sure the culture module is based on all of different ~~people~~ culture.

Thank you for your time and participation

CamScanner

Partecipant No (41) & P(1)

UNIVERSITI MALAYA

Depart of Media and Communication Studies

AI and Mediated Intercultural Communication Questionnaire

Dear UM Student/ Researcher,

Through this survey, we want to assess the probable effects of Artificial Intelligence (AI) on mediated communication among people from different cultures. This instrument which been developed by the help of the existing literature and ChatGPT, has 15 structured items with five (5) options per item and six (6) open-ended questions. Your participation in the survey is voluntary and the personal identifications of the participants will be treated as strictly confidential.

Thank you for your cooperation.

The researchers

A. Demographic Information

1. Gender: ☑ Male ☐ Female

2. Age: 24

3. Nationality: MALAYSIA

4. Level of education: ~~Bachelor~~ Post (Post graduate degree)

5. AI/ ChatGPT experience: ☑ Yes ☐ No

B. Intercultural Sensitivity Scale

Directions: The current AI-ICC questionnaire includes 15 items/ statements with five (5) options per item, as: 1 – Strongly agree; 2 – Agree, 3 – Uncertain; 4 – Disagree, and 5 – Strongly disagree.	Strongly agree	Agree	Uncertain	Disagree	Strongly disagree
1. AI and AI-powered machines can be helpful in bridging communication gaps between people from different cultures.	1	(2)	3	4	5
2. So far, AI has been successful in facilitating cross-cultural understanding and collaboration in mediated communication.	1	(2)	3	4	5
3. AI can help overcome language barriers and promote more inclusive communication among diverse groups.	1	(2)	3	4	5

CamScanner

No (41) & P(2)

4. There some cultural biases and stereotypes in AI-powered language tools and communication platforms.	1	2	(3)	4	5
5. AI has affected the localization and adaptation of content for different cultural audiences in digital communication.	1	2	(3)	4	5
6. I encountered any challenges in using AI-powered translation tools when communicating with people from different cultural backgrounds.	1	2	(3)	4	5
7. AI has the potential to promote cultural exchange and appreciation by enabling easier access to diverse perspectives and information.	1	(2)	3	4	5
8. AI-powered machines and chatbots should be programmed to respect and adapt to cultural norms and communication styles while interacting with users from different cultures.	1	(2)	3	4	5
9. There are some potential risks and concerns regarding AI's role in cross-cultural communication, such as perpetuating cultural stereotypes and misinterpretations.	1	(2)	3	4	5
10. I have participated in some cross-cultural virtual collaborations that relied on AI for communication and coordination.	1	2	3	(4)	5
11. I think AI can enhance intercultural learning experiences and foster empathy among individuals from different cultural backgrounds.	1	(2)	3	4	5
12. AI-powered virtual reality (VR) and augmented reality (AR) technologies can impact cross-cultural communication and understanding.	1	(2)	3	4	5
13. Somehow AI-generated content may unintentionally offend and misled individuals from specific cultural backgrounds.	1	(2)	3	4	5
14. Ethical considerations should be taken into account when designing AI-powered communication tools that cater to diverse cultural contexts.	(1)	2	3	4	5
15. Universities and educational institutions leverage AI to create more inclusive and culturally diverse online learning environments.	1	2	(3)	4	5

In the case of your agreement on sharing your answer/ points of views for questions below, please write down your comment/ point of view for all questions or any question that you prefer:

1. Did you experience the use of AI and AI-powered chatbots, such as ChatGPT and for what purpose?

   Yes. For ~~a~~ general search, academic research and translation purposes

2. How do you perceive the role of AI and AI-powered machines in bridging communication gaps between people from different cultures?

   provide ~~fast~~ better and accurate translation.

No (41) Ec P(3)

3. Have you experienced and observed any instances where AI has been successful in facilitating cross-cultural understanding and collaboration in mediated communication?

Not yet. I don't use AI to communicate ~~[illegible]~~ so far.

4. In what ways do you think AI can help overcome language barriers and promote more inclusive communication among diverse groups?

provide better translation. provide Info on other culture with better context so that people do not misunderstood other culture.

5. Are there any cultural biases or stereotypes that you have noticed in AI-powered language tools or communication platforms?

I haven't noticed since I didn't used for that ~~p~~ purpose yet.

6. How can universities and educational institutions leverage AI to create more inclusive and culturally diverse online learning environments?

Probably uni used ~~[illegible]~~ to get information on other cultures. Uni also can used AI to provide learning in other language.

Thank you for your time and participation

CamScanner

Participant No 42 & P①

UNIVERSITI MALAYA

Depart of Media and Communication Studies

AI and Mediated Intercultural Communication Questionnaire

Dear UM Student/ Researcher,

Through this survey, we want to assess the probable effects of Artificial Intelligence (AI) on mediated communication among people from different cultures. This instrument which been developed by the help of the existing literature and ChatGPT, has 15 structured items with five (5) options per item and six (6) open-ended questions. Your participation in the survey is voluntary and the personal identifications of the participants will be treated as strictly confidential.

Thank you for your cooperation.

The researchers

A. Demographic Information

1. Gender: ☐ Male ✓ Female
2. Age: 28
3. Nationality: China
4. Level of education: PhD
5. AI/ ChatGPT experience: ✓ Yes ☐ No

B. Intercultural Sensitivity Scale

**Directions:** The current AI-ICC questionnaire includes 15 items/ statements with five (5) options per item, as: 1 – Strongly agree; 2 – Agree, 3 – Uncertain; 2 – Disagree, and 5 – Strongly disagree.	Strongly agree	Agree	Uncertain	Disagree	Strongly disagree
1. AI and AI-powered machines can be helpful in bridging communication gaps between people from different cultures.	1	2 ✓	3	4	5
2. So far, AI has been successful in facilitating cross-cultural understanding and collaboration in mediated communication.	1	2 ✓	3	4	5
3. AI can help overcome language barriers and promote more inclusive communication among diverse groups.	1	2 ✓	3	4	5

CS CamScanner

Participant No. [illegible] & P(2)

(42)

4. There some cultural biases and stereotypes in AI-powered language tools and communication platforms.	1	2	3 ✓	4	5
5. AI has affected the localization and adaptation of content for different cultural audiences in digital communication.	1	2	3 ✓	4	5
6. I encountered any challenges in using AI-powered translation tools when communicating with people from different cultural backgrounds.	1	2	3 ✓	4	5
7. AI has the potential to promote cultural exchange and appreciation by enabling easier access to diverse perspectives and information.	1	2 ✓	3	4	5
8. AI-powered machines and chatbots should be programmed to respect and adapt to cultural norms and communication styles while interacting with users from different cultures.	1	2 ✓	3	4	5
9. There are some potential risks and concerns regarding AI's role in cross-cultural communication, such as perpetuating cultural stereotypes and misinterpretations.	1	2 ✓	3	4	5
10. I have participated in some cross-cultural virtual collaborations that relied on AI for communication and coordination.	1	2 ✓	3	4	5
11. I think AI can enhance intercultural learning experiences and foster empathy among individuals from different cultural backgrounds.	1	2 ✓	3	4	5
12. AI-powered virtual reality (VR) and augmented reality (AR) technologies can impact cross-cultural communication and understanding.	1	2 ✓	3	4	5
13. Somehow AI-generated content may unintentionally offend and misled individuals from specific cultural backgrounds.	1	2	3 ✓	4	5
14. Ethical considerations should be taken into account when designing AI-powered communication tools that cater to diverse cultural contexts.	1	2 ✓	3	4	5
15. Universities and educational institutions leverage AI to create more inclusive and culturally diverse online learning environments.	1	2 ✓	3	4	5

In the case of your agreement on sharing your answer/ points of views for questions below, please write down your comment/ point of view for all questions or any question that you prefer:

1. Did you experience the use of AI and AI-powered chatbots, such as ChatGPT and for what purpose?

   Yes. I use it for information seeking and solve problems.

2. How do you perceive the role of AI and AI-powered machines in bridging communication gaps between people from different cultures?

   People can obtain more information about different cultures via AI, which leads to more communication.

CS CamScanner

P10 & P13
P12

3. Have you experienced and observed any instances where AI has been successful in facilitating cross-cultural understanding and collaboration in mediated communication?

AI can be seen to be used in online live-streaming to introduce the culture of a country.

4. In what ways do you think AI can help overcome language barriers and promote more inclusive communication among diverse groups?

AI can help people to translate different language, which ~~proved~~ provides them with a more convinient way to communicate.

5. Are there any cultural biases or stereotypes that you have noticed in AI-powered language tools or communication platforms?

No.

6. How can universities and educational institutions leverage AI to create more inclusive and culturally diverse online learning environments?

They can leverage AI to design creative and interesting content. By using the translation function of AI, ~~peop~~ students can communicate with each other without language barrier.

Thank you for your time and participation

CamScanner

Participant No (43) E, PO

UNIVERSITI MALAYA

Depart of Media and Communication Studies

AI and Mediated Intercultural Communication Questionnaire

Dear UM Student/ Researcher,

Through this survey, we want to assess the probable effects of Artificial Intelligence (AI) on mediated communication among people from different cultures. This instrument which been developed by the help of the existing literature and ChatGPT, has 15 structured items with five (5) options per item and six (6) open-ended questions. Your participation in the survey is voluntary and the personal identifications of the participants will be treated as strictly confidential.

Thank you for your cooperation.

The researchers

A. Demographic Information

1. Gender: ☐ Male ☑ Female
2. Age: 47
3. Nationality: Malaysian
4. Level of education: Postdoc/faculty MA (Master)
5. AI/ ChatGPT experience: ☑ Yes ☐ No

B. Intercultural Sensitivity Scale

**Directions:** The current AI-ICC questionnaire includes 15 items/ statements with five (5) options per item, as: 1 – Strongly agree; 2 – Agree, 3 – Uncertain; 4 – Disagree, and 5 – Strongly disagree.	Strongly agree	Agree	Uncertain	Disagree	Strongly disagree
1. AI and AI-powered machines can be helpful in bridging communication gaps between people from different cultures.	1	2 ✓	3	4	5
2. So far, AI has been successful in facilitating cross-cultural understanding and collaboration in mediated communication.	1	2	3 ✓	4	5
3. AI can help overcome language barriers and promote more inclusive communication among diverse groups.	1	2 ✓	3	4	5

CS CamScanner

P2 No (43) & P(2)

	1	2	3	4	5
4. There some cultural biases and stereotypes in AI-powered language tools and communication platforms.	1 ✓	2	3	4	5
5. AI has affected the localization and adaptation of content for different cultural audiences in digital communication.	1	2	3 ✓	4	5
6. I encountered any challenges in using AI-powered translation tools when communicating with people from different cultural backgrounds.	1 ✓	2	3	4	5
7. AI has the potential to promote cultural exchange and appreciation by enabling easier access to diverse perspectives and information.	1 ✓	2	3	4	5
8. AI-powered machines and chatbots should be programmed to respect and adapt to cultural norms and communication styles while interacting with users from different cultures.	1 ✓	2	3	4	5
9. There are some potential risks and concerns regarding AI's role in cross-cultural communication, such as perpetuating cultural stereotypes and misinterpretations.	1 ✓	2	3	4	5
10. I have participated in some cross-cultural virtual collaborations that relied on AI for communication and coordination.	1	2 ✓	3	4	5
11. I think AI can enhance intercultural learning experiences and foster empathy among individuals from different cultural backgrounds.	1 ✓	2	3	4	5
12. AI-powered virtual reality (VR) and augmented reality (AR) technologies can impact cross-cultural communication and understanding.	1 ✓	2	3	4	5
13. Somehow AI-generated content may unintentionally offend and misled individuals from specific cultural backgrounds.	1 ✓	2	3	4	5
14. Ethical considerations should be taken into account when designing AI-powered communication tools that cater to diverse cultural contexts.	1 ✓	2	3	4	5
15. Universities and educational institutions leverage AI to create more inclusive and culturally diverse online learning environments.	1 ✓	2	3	4	5

In the case of your agreement on sharing your answer/ points of views for questions below, please write down your comment/ point of view for all questions or any question that you prefer:

1. Did you experience the use of AI and AI-powered chatbots, such as ChatGPT and for what purpose?

Discussion with Chinese national students especially for group work.

2. How do you perceive the role of AI and AI-powered machines in bridging communication gaps between people from different cultures?

Important however, it needs to built by people who are feminist (meaning inclusive & safe) unlike current ~~pol~~ platforms such as Facebook Twitter which have allowed for 'hate' to ex[illegible] cause of non-feminist alograrhythms (sp?)

CamScanner

No (43) E P(3)

3. Have you experienced and observed any instances where AI has been successful in facilitating cross-cultural understanding and collaboration in mediated communication?

Yes in group work with coursemates who are not apt in English

4. In what ways do you think AI can help overcome language barriers and promote more inclusive communication among diverse groups?

By working well enough to take into account ~~[illegible]~~ nuances & being designed on feminist ideals.

5. Are there any cultural biases or stereotypes that you have noticed in AI-powered language tools or communication platforms?

Yes but can't think of specifics right now but definitely encountered in Facebook translations from Malay to English

6. How can universities and educational institutions leverage AI to create more inclusive and culturally diverse online learning environments?

Spending money on staffing & technological tools it needs. I also think this will be very good for the university in managing globally student population

Thank you for your time and participation

CamScanner

Participant No (44) & PO

UNIVERSITI MALAYA

Depart of Media and Communication Studies

AI and Mediated Intercultural Communication Questionnaire

Dear UM Student/ Researcher,

Through this survey, we want to assess the probable effects of Artificial Intelligence (AI) on mediated communication among people from different cultures. This instrument which been developed by the help of the existing literature and ChatGPT, has 15 structured items with five (5) options per item and six (6) open-ended questions. Your participation in the survey is voluntary and the personal identifications of the participants will be treated as strictly confidential.

Thank you for your cooperation.

The researchers

A. Demographic Information

1. Gender: ☐ Male ☑ Female

2. Age: 27

3. Nationality: CHINA

4. Level of education: MASTER

5. AI/ ChatGPT experience: ☑ Yes ☐ No

B. Intercultural Sensitivity Scale

Directions: The current AI-ICC questionnaire includes 15 items/ statements with five (5) options per item, as: 1 – Strongly agree; 2 – Agree, 3 – Uncertain; 4 – Disagree, and 5 – Strongly disagree.	Strongly agree	Agree	Uncertain	Disagree	Strongly disagree
1. AI and AI-powered machines can be helpful in bridging communication gaps between people from different cultures.	1	2	3 ✓	4	5
2. So far, AI has been successful in facilitating cross-cultural understanding and collaboration in mediated communication.	1	2	3 ✓	4	5
3. AI can help overcome language barriers and promote more inclusive communication among diverse groups.	1	2 ✓	3	4	5

CamScanner

No 44 & 12

4. There some cultural biases and stereotypes in AI-powered language tools and communication platforms.	1 ✓	2	3	4	5
5. AI has affected the localization and adaptation of content for different cultural audiences in digital communication.	1	2	3 ✓	4	5
6. I encountered any challenges in using AI-powered translation tools when communicating with people from different cultural backgrounds.	1	2	3	4 ✓	5
7. AI has the potential to promote cultural exchange and appreciation by enabling easier access to diverse perspectives and information.	1 ✓	2	3	4	5
8. AI-powered machines and chatbots should be programmed to respect and adapt to cultural norms and communication styles while interacting with users from different cultures.	1 ✓	2	3	4	5
9. There are some potential risks and concerns regarding AI's role in cross-cultural communication, such as perpetuating cultural stereotypes and misinterpretations.	1 ✓	2	3	4	5
10. I have participated in some cross-cultural virtual collaborations that relied on AI for communication and coordination.	1	2	3	4 ✓	5
11. I think AI can enhance intercultural learning experiences and foster empathy among individuals from different cultural backgrounds.	1 ✓	2	3	4	5
12. AI-powered virtual reality (VR) and augmented reality (AR) technologies can impact cross-cultural communication and understanding.	1 ✓	2	3	4	5
13. Somehow AI-generated content may unintentionally offend and misled individuals from specific cultural backgrounds.	1	2 ✓	3	4	5
14. Ethical considerations should be taken into account when designing AI-powered communication tools that cater to diverse cultural contexts.	1 ✓	2	3	4	5
15. Universities and educational institutions leverage AI to create more inclusive and culturally diverse online learning environments.	1	2 ✓	3	4	5

In the case of your agreement on sharing your answer/ points of views for questions below, please write down your comment/ point of view for all questions or any question that you prefer:

1. Did you experience the use of AI and AI-powered chatbots, such as ChatGPT and for what purpose?

   Yes, Search Information or check Information.

2. How do you perceive the role of AI and AI-powered machines in bridging communication gaps between people from different cultures?

   Heplful.

CamScanner

No (44) & P (3)

3. Have you experienced and observed any instances where AI has been successful in facilitating cross-cultural understanding and collaboration in mediated communication?

Not Yet

4. In what ways do you think AI can help overcome language barriers and promote more inclusive communication among diverse groups?

when searching different language website.

5. Are there any cultural biases or stereotypes that you have noticed in AI-powered language tools or communication platforms?

Don't know

6. How can universities and educational institutions leverage AI to create more inclusive and culturally diverse online learning environments?

Collect information from students and teachers.

Thank you for your time and participation

CamScanner

Participant No (45) E P0

UNIVERSITI MALAYA

Depart of Media and Communication Studies

AI and Mediated Intercultural Communication Questionnaire

Dear UM Student/ Researcher,

Through this survey, we want to assess the probable effects of Artificial Intelligence (AI) on mediated communication among people from different cultures. This instrument which been developed by the help of the existing literature and ChatGPT, has 15 structured items with five (5) options per item and six (6) open-ended questions. Your participation in the survey is voluntary and the personal identifications of the participants will be treated as strictly confidential.

Thank you for your cooperation.

The researchers

A. Demographic Information

1. Gender: ☐ Male ☑ Female

2. Age: 24

3. Nationality: China

4. Level of education: Master

5. AI/ ChatGPT experience: ☑ Yes ☐ No

B. Intercultural Sensitivity Scale

Directions: The current AI-ICC questionnaire includes 15 items/ statements with five (5) options per item, as: 1 – Strongly agree; 2 – Agree, 3 – Uncertain; 4 – Disagree, and 5 – Strongly disagree.	Strongly agree	Agree	Uncertain	Disagree	Strongly disagree
1. AI and AI-powered machines can be helpful in bridging communication gaps between people from different cultures.	1	2 ✓	3	4	5
2. So far, AI has been successful in facilitating cross-cultural understanding and collaboration in mediated communication.	1	2 ✓	3	4	5
3. AI can help overcome language barriers and promote more inclusive communication among diverse groups.	1	2 ✓	3	4	5

CamScanner

No (45) E P(2)

4. There some cultural biases and stereotypes in AI-powered language tools and communication platforms.	1	2 ✓	3	4	5
5. AI has affected the localization and adaptation of content for different cultural audiences in digital communication.	1	2 ✓	3	4	5
6. I encountered any challenges in using AI-powered translation tools when communicating with people from different cultural backgrounds.	1	2 ✓	3	4	5
7. AI has the potential to promote cultural exchange and appreciation by enabling easier access to diverse perspectives and information.	1	2 ✓	3	4	5
8. AI-powered machines and chatbots should be programmed to respect and adapt to cultural norms and communication styles while interacting with users from different cultures.	1	2 ✓	3	4	5
9. There are some potential risks and concerns regarding AI's role in cross-cultural communication, such as perpetuating cultural stereotypes and misinterpretations.	1	2	3 ✓	4	5
10. I have participated in some cross-cultural virtual collaborations that relied on AI for communication and coordination.	1	2	3	4 ✓	5
11. I think AI can enhance intercultural learning experiences and foster empathy among individuals from different cultural backgrounds.	1	2	3 ✓	4	5
12. AI-powered virtual reality (VR) and augmented reality (AR) technologies can impact cross-cultural communication and understanding.	1	2	3 ✓	4	5
13. Somehow AI-generated content may unintentionally offend and misled individuals from specific cultural backgrounds.	1	2 ✓	3	4	5
14. Ethical considerations should be taken into account when designing AI-powered communication tools that cater to diverse cultural contexts.	1	2 ✓	3	4	5
15. Universities and educational institutions leverage AI to create more inclusive and culturally diverse online learning environments.	1	2 ✓	3	4	5

In the case of your agreement on sharing your answer/ points of views for questions below, please write down your comment/ point of view for all questions or any question that you prefer:

1. Did you experience the use of AI and AI-powered chatbots, such as ChatGPT and for what purpose?

Yes

2. How do you perceive the role of AI and AI-powered machines in bridging communication gaps between people from different cultures?

Language translation, Cultural Difference, Information Collection

CamScanner

No (45) E P3

3. Have you experienced and observed any instances where AI has been successful in facilitating cross-cultural understanding and collaboration in mediated communication?

Language translation apps,

4. In what ways do you think AI can help overcome language barriers and promote more inclusive communication among diverse groups?

Real time translation, virtual chatbots

5. Are there any cultural biases or stereotypes that you have noticed in AI-powered language tools or communication platforms?

Gender bias, Cultural

6. How can universities and educational institutions leverage AI to create more inclusive and culturally diverse online learning environments?

Personalized learning paths, language support.

Thank you for your time and participation

CS CamScanner

Participant No (46) & PO

UNIVERSITI MALAYA

Depart of Media and Communication Studies

AI and Mediated Intercultural Communication Questionnaire

Dear UM Student/ Researcher,

Through this survey, we want to assess the probable effects of Artificial Intelligence (AI) on mediated communication among people from different cultures. This instrument which been developed by the help of the existing literature and ChatGPT, has 15 structured items with five (5) options per item and six (6) open-ended questions. Your participation in the survey is voluntary and the personal identifications of the participants will be treated as strictly confidential.

Thank you for your cooperation.

The researchers

A. Demographic Information

1. Gender: ☑ Male ☐ Female

2. Age: 25

3. Nationality: Chinese

4. Level of education: PHD

5. AI/ ChatGPT experience: ☑ Yes ☐ No

B. Intercultural Sensitivity Scale

**Directions:** The current AI-ICC questionnaire includes 15 items/ statements with five (5) options per item, as: 1 – Strongly agree; 2 – Agree, 3 – Uncertain; 4 – Disagree, and 5 – Strongly disagree.	Strongly agree	Agree	Uncertain	Disagree	Strongly disagree
1. AI and AI-powered machines can be helpful in bridging communication gaps between people from different cultures.	1 ✓	2	3	4	5
2. So far, AI has been successful in facilitating cross-cultural understanding and collaboration in mediated communication.	1	2	3 ✓	4	5
3. AI can help overcome language barriers and promote more inclusive communication among diverse groups.	1 ✓	2	3	4	5

CamScanner

No (46) E, P2

4. There some cultural biases and stereotypes in AI-powered language tools and communication platforms.	1	2	3	4	5
5. AI has affected the localization and adaptation of content for different cultural audiences in digital communication.	1	2	3	4	5
6. I encountered any challenges in using AI-powered translation tools when communicating with people from different cultural backgrounds.	1	2	3	4	5
7. AI has the potential to promote cultural exchange and appreciation by enabling easier access to diverse perspectives and information.	1	2	3	4	5
8. AI-powered machines and chatbots should be programmed to respect and adapt to cultural norms and communication styles while interacting with users from different cultures.	1	2	3	4	5
9. There are some potential risks and concerns regarding AI's role in cross-cultural communication, such as perpetuating cultural stereotypes and misinterpretations.	1	2	3	4	5
10. I have participated in some cross-cultural virtual collaborations that relied on AI for communication and coordination.	1	2	3	4	5
11. I think AI can enhance intercultural learning experiences and foster empathy among individuals from different cultural backgrounds.	1	2	3	4	5
12. AI-powered virtual reality (VR) and augmented reality (AR) technologies can impact cross-cultural communication and understanding.	1	2	3	4	5
13. Somehow AI-generated content may unintentionally offend and misled individuals from specific cultural backgrounds.	1	2	3	4	5
14. Ethical considerations should be taken into account when designing AI-powered communication tools that cater to diverse cultural contexts.	1	2	3	4	5
15. Universities and educational institutions leverage AI to create more inclusive and culturally diverse online learning environments.	1	2	3	4	5

In the case of your agreement on sharing your answer/ points of views for questions below, please write down your comment/ point of view for all questions or any question that you prefer:

1. Did you experience the use of AI and AI-powered chatbots, such as ChatGPT and for what purpose?

   get knowledge / get guide. for working plan

2. How do you perceive the role of AI and AI-powered machines in bridging communication gaps between people from different cultures?

   Can communicate easier with translation.

CS CamScanner

No (46) & P(3)

3. Have you experienced and observed any instances where AI has been successful in facilitating cross-cultural understanding and collaboration in mediated communication?

Not really.

4. In what ways do you think AI can help overcome language barriers and promote more inclusive communication among diverse groups?

translation quickly and more accurant to make people understand each other

5. Are there any cultural biases or stereotypes that you have noticed in AI-powered language tools or communication platforms?

translation may not in current context or. follow the new meaning in that culture,

6. How can universities and educational institutions leverage AI to create more inclusive and culturally diverse online learning environments?

I think AI could ① create diverse cultural moudles to students with different backgrounds ② provide differant cultural resources. such as history, culture meanings to study.

Thank you for your time and participation

CamScanner

Partcipant No (47) & P(1)

UNIVERSITI MALAYA

Depart of Media and Communication Studies

AI and Mediated Intercultural Communication Questionnaire

Dear UM Student/ Researcher,

Through this survey, we want to assess the probable effects of Artificial Intelligence (AI) on mediated communication among people from different cultures. This instrument which been developed by the help of the existing literature and ChatGPT, has 15 structured items with five (5) options per item and six (6) open-ended questions. Your participation in the survey is voluntary and the personal identifications of the participants will be treated as strictly confidential.

Thank you for your cooperation.

The researchers

A. Demographic Information

1. Gender: ☐ Male ☑ Female

2. Age: 28

3. Nationality: Chinese

4. Level of education: PHD

5. AI/ ChatGPT experience: ☑ Yes ☐ No

B. Intercultural Sensitivity Scale

**Directions:** The current AI-ICC questionnaire includes 15 items/ statements with five (5) options per item, as: 1 – Strongly agree; 2 – Agree, 3 – Uncertain; 4 – Disagree, and 5 – Strongly disagree.	Strongly agree	Agree	Uncertain	Disagree	Strongly disagree
1. AI and AI-powered machines can be helpful in bridging communication gaps between people from different cultures.	1	2	3 ✓	4	5
2. So far, AI has been successful in facilitating cross-cultural understanding and collaboration in mediated communication.	1	2	3 ✓	4	5
3. AI can help overcome language barriers and promote more inclusive communication among diverse groups.	1	2	3 ✓	4	5

CamScanner

No (47) & P2

4. There some cultural biases and stereotypes in AI-powered language tools and communication platforms.	1	2 ✓	3	4	5
5. AI has affected the localization and adaptation of content for different cultural audiences in digital communication.	1	2 ✓	3	4	5
6. I encountered any challenges in using AI-powered translation tools when communicating with people from different cultural backgrounds.	1	2	3 ✓	4	5
7. AI has the potential to promote cultural exchange and appreciation by enabling easier access to diverse perspectives and information.	1	2 ✓	3	4	5
8. AI-powered machines and chatbots should be programmed to respect and adapt to cultural norms and communication styles while interacting with users from different cultures.	1 ✓	2	3	4	5
9. There are some potential risks and concerns regarding AI's role in cross-cultural communication, such as perpetuating cultural stereotypes and misinterpretations.	1	2 ✓	3	4	5
10. I have participated in some cross-cultural virtual collaborations that relied on AI for communication and coordination.	1	2	3	4 ✓	5
11. I think AI can enhance intercultural learning experiences and foster empathy among individuals from different cultural backgrounds.	1	2	3 ✓	4	5
12. AI-powered virtual reality (VR) and augmented reality (AR) technologies can impact cross-cultural communication and understanding.	1	2	3 ✓	4	5
13. Somehow AI-generated content may unintentionally offend and misled individuals from specific cultural backgrounds.	1 ✓	2	3	4	5
14. Ethical considerations should be taken into account when designing AI-powered communication tools that cater to diverse cultural contexts.	1 ✓	2	3	4	5
15. Universities and educational institutions leverage AI to create more inclusive and culturally diverse online learning environments.	1	2	3 ✓	4	5

In the case of your agreement on sharing your answer/ points of views for questions below, please write down your comment/ point of view for all questions or any question that you prefer:

1. Did you experience the use of AI and AI-powered chatbots, such as ChatGPT and for what purpose?

For the purpose of ~~finding~~ ~~the~~ ~~a~~ narrowing my knowledge gap

2. How do you perceive the role of AI and AI-powered machines in bridging communication gaps between people from different cultures?

I ~~don't~~ ~~can't~~ can not agree with AI and AI-powered machines play important 2 role in narrowing the gaps between people from diferents cultures.

CS CamScanner

No (47) & P(3)

3. Have you experienced and observed any instances where AI has been successful in facilitating cross-cultural understanding and collaboration in mediated communication?

NO

4. In what ways do you think AI can help overcome language barriers and promote more inclusive communication among diverse groups?

~~I~~ ~~In ways that critically~~

~~I~~ Sorry. I am ~~m~~ not sure.

5. Are there any cultural biases or stereotypes that you have noticed in AI-powered language tools or communication platforms?

NO

6. How can universities and educational institutions leverage AI to create more inclusive and culturally diverse online learning environments?

I have no idea

Thank you for your time and participation

CS CamScanner

Partcipant No (48) & P0

UNIVERSITI MALAYA

Depart of Media and Communication Studies

AI and Mediated Intercultural Communication Questionnaire

Dear UM Student/ Researcher,

Through this survey, we want to assess the probable effects of Artificial Intelligence (AI) on mediated communication among people from different cultures. This instrument which been developed by the help of the existing literature and ChatGPT, has 15 structured items with five (5) options per item and six (6) open-ended questions. Your participation in the survey is voluntary and the personal identifications of the participants will be treated as strictly confidential.

Thank you for your cooperation.

The researchers

A. Demographic Information

1. Gender: ☐ Male ☑ Female

2. Age: 23 years old

3. Nationality: Malaysian

4. Level of education: ~~Tertiary~~ Post Graduation

5. AI/ ChatGPT experience: ☑ Yes ☐ No

B. Intercultural Sensitivity Scale

**Directions:** The current AI-ICC questionnaire includes 15 items/ statements with five (5) options per item, as: 1 – Strongly agree; 2 – Agree, 3 – Uncertain; 4 – Disagree, and 5 – Strongly disagree.	Strongly agree	Agree	Uncertain	Disagree	Strongly disagree
1. AI and AI-powered machines can be helpful in bridging communication gaps between people from different cultures.	1	(2)	3	4	5
2. So far, AI has been successful in facilitating cross-cultural understanding and collaboration in mediated communication.	1	(2)	3	4	5
3. AI can help overcome language barriers and promote more inclusive communication among diverse groups.	1	(2)	3	4	5

CamScanner

No (48) & P(3)

4. There some cultural biases and stereotypes in AI-powered language tools and communication platforms.	1	2	3	4	5
5. AI has affected the localization and adaptation of content for different cultural audiences in digital communication.	1	2	3	4	5
6. I encountered any challenges in using AI-powered translation tools when communicating with people from different cultural backgrounds.	1	2	3	4	5
7. AI has the potential to promote cultural exchange and appreciation by enabling easier access to diverse perspectives and information.	1	2	3	4	5
8. AI-powered machines and chatbots should be programmed to respect and adapt to cultural norms and communication styles while interacting with users from different cultures.	1	2	3	4	5
9. There are some potential risks and concerns regarding AI's role in cross-cultural communication, such as perpetuating cultural stereotypes and misinterpretations.	1	2	3	4	5
10. I have participated in some cross-cultural virtual collaborations that relied on AI for communication and coordination.	1	2	3	4	5
11. I think AI can enhance intercultural learning experiences and foster empathy among individuals from different cultural backgrounds.	1	2	3	4	5
12. AI-powered virtual reality (VR) and augmented reality (AR) technologies can impact cross-cultural communication and understanding.	1	2	3	4	5
13. Somehow AI-generated content may unintentionally offend and misled individuals from specific cultural backgrounds.	1	2	3	4	5
14. Ethical considerations should be taken into account when designing AI-powered communication tools that cater to diverse cultural contexts.	1	2	3	4	5
15. Universities and educational institutions leverage AI to create more inclusive and culturally diverse online learning environments.	1	2	3	4	5

In the case of your agreement on sharing your answer/ points of views for questions below, please write down your comment/ point of view for all questions or any question that you prefer:

1. Did you experience the use of AI and AI-powered chatbots, such as ChatGPT and for what purpose?

   Yes, for obtaining information for learning.

2. How do you perceive the role of AI and AI-powered machines in bridging communication gaps between people from different cultures?

   Sorry, not sure.

CamScanner

No (48) & P(3)

3. Have you experienced and observed any instances where AI has been successful in facilitating cross-cultural understanding and collaboration in mediated communication?

No

4. In what ways do you think AI can help overcome language barriers and promote more inclusive communication among diverse groups?

It can directly translates the information in different languages that allow other people to understand.

5. Are there any cultural biases or stereotypes that you have noticed in AI-powered language tools or communication platforms?

So far, no.

6. How can universities and educational institutions leverage AI to create more inclusive and culturally diverse online learning environments?

Not sure.

Thank you for your time and participation

CamScanner

Participant No (49) E, PO

UNIVERSITI MALAYA

Depart of Media and Communication Studies

AI and Mediated Intercultural Communication Questionnaire

Dear UM Student/ Researcher,

Through this survey, we want to assess the probable effects of Artificial Intelligence (AI) on mediated communication among people from different cultures. This instrument which been developed by the help of the existing literature and ChatGPT, has 15 structured items with five (5) options per item and six (6) open-ended questions. Your participation in the survey is voluntary and the personal identifications of the participants will be treated as strictly confidential.

Thank you for your cooperation.

The researchers

A. Demographic Information

1. Gender: ☑ Male ☐ Female

2. Age: 26

3. Nationality: Malaysian

4. Level of education: Postgrade-M

5. AI/ ChatGPT experience: ☑ Yes ☐ No

B. Intercultural Sensitivity Scale

Directions: The current AI-ICC questionnaire includes 15 items/ statements with five (5) options per item, as: 1 – Strongly agree; 2 – Agree, 3 – Uncertain; 4 – Disagree, and 5 – Strongly disagree.	Strongly agree	Agree	Uncertain	Disagree	Strongly disagree
1. AI and AI-powered machines can be helpful in bridging communication gaps between people from different cultures.	✓	2	3	4	5
2. So far, AI has been successful in facilitating cross-cultural understanding and collaboration in mediated communication.	1 ✓	2	3	4	5
3. AI can help overcome language barriers and promote more inclusive communication among diverse groups.	1 ✓	2	3	4	5

CS CamScanner

No – (49) E, P(2)

4. There some cultural biases and stereotypes in AI-powered language tools and communication platforms.	1 ✓	2	3	4	5
5. AI has affected the localization and adaptation of content for different cultural audiences in digital communication.	1 ✓	2	3	4	5
6. I encountered any challenges in using AI-powered translation tools when communicating with people from different cultural backgrounds.	1 ✓	2	3	4	5
7. AI has the potential to promote cultural exchange and appreciation by enabling easier access to diverse perspectives and information.	1 ✓	2	3	4	5
8. AI-powered machines and chatbots should be programmed to respect and adapt to cultural norms and communication styles while interacting with users from different cultures.	1 ✓	2	3	4	5
9. There are some potential risks and concerns regarding AI's role in cross-cultural communication, such as perpetuating cultural stereotypes and misinterpretations.	1 ✓	2	3	4	5
10. I have participated in some cross-cultural virtual collaborations that relied on AI for communication and coordination.	1	2	3	4	5 ✓
11. I think AI can enhance intercultural learning experiences and foster empathy among individuals from different cultural backgrounds.	1	2	3	4	5 ✓
12. AI-powered virtual reality (VR) and augmented reality (AR) technologies can impact cross-cultural communication and understanding.	1	2	3	4	5 ✓
13. Somehow AI-generated content may unintentionally offend and misled individuals from specific cultural backgrounds.	1	2	3 ✓	4	5
14. Ethical considerations should be taken into account when designing AI-powered communication tools that cater to diverse cultural contexts.	1 ✓	2	3	4	5
15. Universities and educational institutions leverage AI to create more inclusive and culturally diverse online learning environments.	1 ✓	2	3	4	5

In the case of your agreement on sharing your answer/ points of views for questions below, please write down your comment/ point of view for all questions or any question that you prefer:

1. Did you experience the use of AI and AI-powered chatbots, such as ChatGPT and for what purpose?

   Yes. Coding and understanding unknown terms

2. How do you perceive the role of AI and AI-powered machines in bridging communication gaps between people from different cultures?

   Optimistic about the ability to bridge different 2 cultures

CS CamScanner

No (49) E, P (3)

3. Have you experienced and observed any instances where AI has been successful in facilitating cross-cultural understanding and collaboration in mediated communication?

Not yet

4. In what ways do you think AI can help overcome language barriers and promote more inclusive communication among diverse groups?

Apps with automatic translation

5. Are there any cultural biases or stereotypes that you have noticed in AI-powered language tools or communication platforms?

Not experience any.

6. How can universities and educational institutions leverage AI to create more inclusive and culturally diverse online learning environments?

Utilize more generative AI tools

Thank you for your time and participation

CamScanner

Participant (50) & P(1)

UNIVERSITI MALAYA

Depart of Media and Communication Studies

AI and Mediated Intercultural Communication Questionnaire

Dear UM Student/ Researcher,

Through this survey, we want to assess the probable effects of Artificial Intelligence (AI) on mediated communication among people from different cultures. This instrument which been developed by the help of the existing literature and ChatGPT, has 15 structured items with five (5) options per item and six (6) open-ended questions. Your participation in the survey is voluntary and the personal identifications of the participants will be treated as strictly confidential.

Thank you for your cooperation.

The researchers

A. Demographic Information

1. Gender: ☑ Male ☐ Female

2. Age: 24

3. Nationality: MALAYSIAN

4. Level of education: BACHELOR'S DEGREE

5. AI/ ChatGPT experience: ☑ Yes ☐ No

B. Intercultural Sensitivity Scale

**Directions:** The current AI-ICC questionnaire includes 15 items/ statements with five (5) options per item, as: 1 – Strongly agree; 2 – Agree, 3 – Uncertain; 4 – Disagree, and 5 – Strongly disagree.	Strongly agree	Agree	Uncertain	Disagree	Strongly disagree
1. AI and AI-powered machines can be helpful in bridging communication gaps between people from different cultures.	1	(2)	3	4	5
2. So far, AI has been successful in facilitating cross-cultural understanding and collaboration in mediated communication.	1	2	(3)	4	5
3. AI can help overcome language barriers and promote more inclusive communication among diverse groups.	1	(2)	3	4	5

CamScanner

No (50) & PQ

4. There some cultural biases and stereotypes in AI-powered language tools and communication platforms.	1	2	3	4	5
5. AI has affected the localization and adaptation of content for different cultural audiences in digital communication.	1	2	3	4	5
6. I encountered any challenges in using AI-powered translation tools when communicating with people from different cultural backgrounds.	1	2	3	4	5
7. AI has the potential to promote cultural exchange and appreciation by enabling easier access to diverse perspectives and information.	1	2	3	4	5
8. AI-powered machines and chatbots should be programmed to respect and adapt to cultural norms and communication styles while interacting with users from different cultures.	1	2	3	4	5
9. There are some potential risks and concerns regarding AI's role in cross-cultural communication, such as perpetuating cultural stereotypes and misinterpretations.	1	2	3	4	5
10. I have participated in some cross-cultural virtual collaborations that relied on AI for communication and coordination.	1	2	3	4	5
11. I think AI can enhance intercultural learning experiences and foster empathy among individuals from different cultural backgrounds.	1	2	3	4	5
12. AI-powered virtual reality (VR) and augmented reality (AR) technologies can impact cross-cultural communication and understanding.	1	2	3	4	5
13. Somehow AI-generated content may unintentionally offend and misled individuals from specific cultural backgrounds.	1	2	3	4	5
14. Ethical considerations should be taken into account when designing AI-powered communication tools that cater to diverse cultural contexts.	1	2	3	4	5
15. Universities and educational institutions leverage AI to create more inclusive and culturally diverse online learning environments.	1	2	3	4	5

In the case of your agreement on sharing your answer/ points of views for questions below, please write down your comment/ point of view for all questions or any question that you prefer:

1. Did you experience the use of AI and AI-powered chatbots, such as ChatGPT and for what purpose?

   Yes. Purpose included generating ideas for certain function/purposes

2. How do you perceive the role of AI and AI-powered machines in bridging communication gaps between people from different cultures?

   No experience with one

CamScanner

No (50) & P3

3. Have you experienced and observed any instances where AI has been successful in facilitating cross-cultural understanding and collaboration in mediated communication?

No

4. In what ways do you think AI can help overcome language barriers and promote more inclusive communication among diverse groups?

optimizing Text to speech and speech to text

5. Are there any cultural biases or stereotypes that you have noticed in AI-powered language tools or communication platforms?

No

6. How can universities and educational institutions leverage AI to create more inclusive and culturally diverse online learning environments?

Auto captions on live classes

Thank you for your time and participation

CS CamScanner

Participant (51) & P(1)

UNIVERSITI MALAYA

Depart of Media and Communication Studies

AI and Mediated Intercultural Communication Questionnaire

Dear UM Student/ Researcher,

Through this survey, we want to assess the probable effects of Artificial Intelligence (AI) on mediated communication among people from different cultures. This instrument which been developed by the help of the existing literature and ChatGPT, has 15 structured items with five (5) options per item and six (6) open-ended questions. Your participation in the survey is voluntary and the personal identifications of the participants will be treated as strictly confidential.

Thank you for your cooperation.

The researchers

A. Demographic Information

1. Gender: ☐ Male ☑ Female

2. Age: 25

3. Nationality: china

4. Level of education: master

5. AI/ ChatGPT experience: ☑ Yes ☐ No

B. Intercultural Sensitivity Scale

Directions: The current AI-ICC questionnaire includes 15 items/ statements with five (5) options per item, as: 1 – Strongly agree; 2 – Agree, 3 – Uncertain; 2 – Disagree, and 5 – Strongly disagree.	Strongly agree	Agree	Uncertain	Disagree	Strongly disagree
1. AI and AI-powered machines can be helpful in bridging communication gaps between people from different cultures.	1	2 ✓	3	4	5
2. So far, AI has been successful in facilitating cross-cultural understanding and collaboration in mediated communication.	1	2 ✓	3	4	5
3. AI can help overcome language barriers and promote more inclusive communication among diverse groups.	1	2 ✓	3	4	5

CamScanner

No (51) E P(2)

	1	2	3	4	5
4. There some cultural biases and stereotypes in AI-powered language tools and communication platforms.	1	2 ✓	3	4	5
5. AI has affected the localization and adaptation of content for different cultural audiences in digital communication.	1	2 ✓	3	4	5
6. I encountered any challenges in using AI-powered translation tools when communicating with people from different cultural backgrounds.	1	2	3 ✓	4	5
7. AI has the potential to promote cultural exchange and appreciation by enabling easier access to diverse perspectives and information.	1	2	3 ✓	4	5
8. AI-powered machines and chatbots should be programmed to respect and adapt to cultural norms and communication styles while interacting with users from different cultures.	1	2 ✓	3	4	5
9. There are some potential risks and concerns regarding AI's role in cross-cultural communication, such as perpetuating cultural stereotypes and misinterpretations.	1	2 ✓	3	4	5
10. I have participated in some cross-cultural virtual collaborations that relied on AI for communication and coordination.	1	2 ✓	3	4	5
11. I think AI can enhance intercultural learning experiences and foster empathy among individuals from different cultural backgrounds.	1	2	3 ✓	4	5
12. AI-powered virtual reality (VR) and augmented reality (AR) technologies can impact cross-cultural communication and understanding.	1	2	3 ✓	4	5
13. Somehow AI-generated content may unintentionally offend and misled individuals from specific cultural backgrounds.	1	2 ✓	3	4	5
14. Ethical considerations should be taken into account when designing AI-powered communication tools that cater to diverse cultural contexts.	1	2	3 ✓	4	5
15. Universities and educational institutions leverage AI to create more inclusive and culturally diverse online learning environments.	1	2	3 ✓	4	5

In the case of your agreement on sharing your answer/ points of views for questions below, please write down your comment/ point of view for all questions or any question that you prefer:

1. Did you experience the use of AI and AI-powered chatbots, such as ChatGPT and for what purpose?

   yes, find some informacion

2. How do you perceive the role of AI and AI-powered machines in bridging communication gaps between people from different cultures?

   fast, and conveniente

CamScanner

No (51) & P(3)

3. Have you experienced and observed any instances where AI has been successful in facilitating cross-cultural understanding and collaboration in mediated communication?

NO

4. In what ways do you think AI can help overcome language barriers and promote more inclusive communication among diverse groups?

it can present a question in multiple languages.

5. Are there any cultural biases or stereotypes that you have noticed in AI-powered language tools or communication platforms?

NO

6. How can universities and educational institutions leverage AI to create more inclusive and culturally diverse online learning environments?

use it extensively

Thank you for your time and participation

CS CamScanner

Partcipant (52) & P(1)

UNIVERSITI MALAYA

Depart of Media and Communication Studies

AI and Mediated Intercultural Communication Questionnaire

Dear UM Student/ Researcher,

Through this survey, we want to assess the probable effects of Artificial Intelligence (AI) on mediated communication among people from different cultures. This instrument which been developed by the help of the existing literature and ChatGPT, has 15 structured items with five (5) options per item and six (6) open-ended questions. Your participation in the survey is voluntary and the personal identifications of the participants will be treated as strictly confidential.

Thank you for your cooperation.

The researchers

A. Demographic Information

1. Gender: ☑ Male ☐ Female

2. Age: 26

3. Nationality: Malaysian

4. Level of education: Post-grade ~~undergrad~~

5. AI/ ChatGPT experience: ☑ Yes ☐ No

B. Intercultural Sensitivity Scale

Directions: The current AI-ICC questionnaire includes 15 items/ statements with five (5) options per item, as: 1 – Strongly agree; 2 – Agree, 3 – Uncertain; 2 – Disagree, and 5 – Strongly disagree.	Strongly agree	Agree	Uncertain	Disagree	Strongly disagree
1. AI and AI-powered machines can be helpful in bridging communication gaps between people from different cultures.	(1)	2	3	4	5
2. So far, AI has been successful in facilitating cross-cultural understanding and collaboration in mediated communication.	(1)	2	3	4	5
3. AI can help overcome language barriers and promote more inclusive communication among diverse groups.	(1)	2	3	4	5

CamScanner

No 52 & R2

4. There some cultural biases and stereotypes in AI-powered language tools and communication platforms.	1	2	**3**	4	5
5. AI has affected the localization and adaptation of content for different cultural audiences in digital communication.	**1**	2	3	4	5
6. I encountered any challenges in using AI-powered translation tools when communicating with people from different cultural backgrounds.	1	2	**3**	4	5
7. AI has the potential to promote cultural exchange and appreciation by enabling easier access to diverse perspectives and information.	**1**	2	3	4	5
8. AI-powered machines and chatbots should be programmed to respect and adapt to cultural norms and communication styles while interacting with users from different cultures.	**1**	2	3	4	5
9. There are some potential risks and concerns regarding AI's role in cross-cultural communication, such as perpetuating cultural stereotypes and misinterpretations.	**1**	2	3	4	5
10. I have participated in some cross-cultural virtual collaborations that relied on AI for communication and coordination.	1	2	3	4	**5**
11. I think AI can enhance intercultural learning experiences and foster empathy among individuals from different cultural backgrounds.	1	**2**	3	4	5
12. AI-powered virtual reality (VR) and augmented reality (AR) technologies can impact cross-cultural communication and understanding.	1	**2**	3	4	5
13. Somehow AI-generated content may unintentionally offend and misled individuals from specific cultural backgrounds.	1	**2**	3	4	5
14. Ethical considerations should be taken into account when designing AI-powered communication tools that cater to diverse cultural contexts.	1	2	**3**	4	5
15. Universities and educational institutions leverage AI to create more inclusive and culturally diverse online learning environments.	1	2	**3**	4	5

In the case of your agreement on sharing your answer/ points of views for questions below, please write down your comment/ point of view for all questions or any question that you prefer:

1. Did you experience the use of AI and AI-powered chatbots, such as ChatGPT and for what purpose?

   Yes, for finding medical info.

2. How do you perceive the role of AI and AI-powered machines in bridging communication gaps between people from different cultures?

   I'm sure it will help to bridge the gap between cultures in terms of language barriors.

CS CamScanner

No (59) & P(3)

3. Have you experienced and observed any instances where AI has been successful in facilitating cross-cultural understanding and collaboration in mediated communication?

NO.

4. In what ways do you think AI can help overcome language barriers and promote more inclusive communication among diverse groups? By helping translate sentences and social cues so that we can better understand people from other cultures.

5. Are there any cultural biases or stereotypes that you have noticed in AI-powered language tools or communication platforms?

No.

6. How can universities and educational institutions leverage AI to create more inclusive and culturally diverse online learning environments? By integrating AI into all classes and institutions

Thank you for your time and participation

CS CamScanner

Participant No (53) & P(1)

UNIVERSITI MALAYA

Depart of Media and Communication Studies

AI and Mediated Intercultural Communication Questionnaire

Dear UM Student/ Researcher,

Through this survey, we want to assess the probable effects of Artificial Intelligence (AI) on mediated communication among people from different cultures. This instrument which been developed by the help of the existing literature and ChatGPT, has 15 structured items with five (5) options per item and six (6) open-ended questions. Your participation in the survey is voluntary and the personal identifications of the participants will be treated as strictly confidential.

Thank you for your cooperation.

The researchers

A. Demographic Information

1. Gender: ☐ Male ✓ Female

2. Age: 25

3. Nationality: China

4. Level of education: Postgraduate-M

5. AI/ ChatGPT experience: ✓ Yes ☐ No

B. Intercultural Sensitivity Scale

**Directions:** The current AI-ICC questionnaire includes 15 items/ statements with five (5) options per item, as: 1 – Strongly agree; 2 – Agree, 3 – Uncertain; 2 – Disagree, and 5 – Strongly disagree.	Strongly agree	Agree	Uncertain	Disagree	Strongly disagree
1. AI and AI-powered machines can be helpful in bridging communication gaps between people from different cultures.	1	2	3 ✓	4	5
2. So far, AI has been successful in facilitating cross-cultural understanding and collaboration in mediated communication.	1	2	3	4 ✓	5
3. AI can help overcome language barriers and promote more inclusive communication among diverse groups.	1	2	3 ✓	4	5

CamScanner

No (53) & P(2)

4. There some cultural biases and stereotypes in AI-powered language tools and communication platforms.	1	2	3 ✓	4	5
5. AI has affected the localization and adaptation of content for different cultural audiences in digital communication.	1	2	3	4 ✓	5
6. I encountered any challenges in using AI-powered translation tools when communicating with people from different cultural backgrounds.	1	2	3	4 ✓	5
7. AI has the potential to promote cultural exchange and appreciation by enabling easier access to diverse perspectives and information.	1	2 ✓	3	4	5
8. AI-powered machines and chatbots should be programmed to respect and adapt to cultural norms and communication styles while interacting with users from different cultures.	1 ✓	2	3	4	5
9. There are some potential risks and concerns regarding AI's role in cross-cultural communication, such as perpetuating cultural stereotypes and misinterpretations.	1	2	3 ✓	4	5
10. I have participated in some cross-cultural virtual collaborations that relied on AI for communication and coordination.	1	2	3	4 ✓	5
11. I think AI can enhance intercultural learning experiences and foster empathy among individuals from different cultural backgrounds.	1	2	3 ✓	4	5
12. AI-powered virtual reality (VR) and augmented reality (AR) technologies can impact cross-cultural communication and understanding.	1	2	3	4 ✓	5
13. Somehow AI-generated content may unintentionally offend and misled individuals from specific cultural backgrounds.	1	2	3 ✓	4	5
14. Ethical considerations should be taken into account when designing AI-powered communication tools that cater to diverse cultural contexts.	1	2 ✓	3	4	5
15. Universities and educational institutions leverage AI to create more inclusive and culturally diverse online learning environments.	1	2	3 ✓	4	5

In the case of your agreement on sharing your answer/ points of views for questions below, please write down your comment/ point of view for all questions or any question that you prefer:

1. Did you experience the use of AI and AI-powered chatbots, such as ChatGPT and for what purpose?

yeah, ChatGPT, for academic

2. How do you perceive the role of AI and AI-powered machines in bridging communication gaps between people from different cultures?

It's a great way if it can achieve the role in bridging Communication gap

CamScanner

NO (53) & P(3)

3. Have you experienced and observed any instances where AI has been successful in facilitating cross-cultural understanding and collaboration in mediated communication?

no experience about that.

4. In what ways do you think AI can help overcome language barriers and promote more inclusive communication among diverse groups?

It should be designedto respect diffierent culture norms.

5. Are there any cultural biases or stereotypes that you have noticed in AI-powered language tools or communication platforms?

No, ~~have~~ I rarely use AI.

6. How can universities and educational institutions leverage AI to create more inclusive and culturally diverse online learning environments?

No idea

Thank you for your time and participation

CamScanner

Partcipant No (54) & P(1)

UNIVERSITI MALAYA

Depart of Media and Communication Studies

AI and Mediated Intercultural Communication Questionnaire

Dear UM Student/ Researcher,

Through this survey, we want to assess the probable effects of Artificial Intelligence (AI) on mediated communication among people from different cultures. This instrument which been developed by the help of the existing literature and ChatGPT, has 15 structured items with five (5) options per item and six (6) open-ended questions. Your participation in the survey is voluntary and the personal identifications of the participants will be treated as strictly confidential.

Thank you for your cooperation.

The researchers

A. Demographic Information

1. Gender: ☐ Male ☑ Female

2. Age: 28.

3. Nationality: ~~thies~~ chinese.

4. Level of education: Master

5. AI/ ChatGPT experience: ☑ Yes ☐ No

B. Intercultural Sensitivity Scale

**Directions:** The current AI-ICC questionnaire includes 15 items/ statements with five (5) options per item, as: 1 – Strongly agree; 2 – Agree, 3 – Uncertain; 2 – Disagree, and 5 – Strongly disagree.	Strongly agree	Agree	Uncertain	Disagree	Strongly disagree
1. AI and AI-powered machines can be helpful in bridging communication gaps between people from different cultures.	1	✓2	3	4	5
2. So far, AI has been successful in facilitating cross-cultural understanding and collaboration in mediated communication.	1	✓2	3	4	5
3. AI can help overcome language barriers and promote more inclusive communication among diverse groups.	✓1	2	3	4	5

CamScanner

No (54) [illegible]

4. There some cultural biases and stereotypes in AI-powered language tools and communication platforms.	1	2 ✓	3	4	5
5. AI has affected the localization and adaptation of content for different cultural audiences in digital communication.	1	2 ✓	3	4	5
6. I encountered any challenges in using AI-powered translation tools when communicating with people from different cultural backgrounds.	1 ✓	2	3	4	5
7. AI has the potential to promote cultural exchange and appreciation by enabling easier access to diverse perspectives and information.	1 ✓	2	3	4	5
8. AI-powered machines and chatbots should be programmed to respect and adapt to cultural norms and communication styles while interacting with users from different cultures.	1	2 ✓	3	4	5
9. There are some potential risks and concerns regarding AI's role in cross-cultural communication, such as perpetuating cultural stereotypes and misinterpretations.	1	2	3 ✓	4	5
10. I have participated in some cross-cultural virtual collaborations that relied on AI for communication and coordination.	1	2	3 ✓	4	5
11. I think AI can enhance intercultural learning experiences and foster empathy among individuals from different cultural backgrounds.	1	2 ✓	3	4	5
12. AI-powered virtual reality (VR) and augmented reality (AR) technologies can impact cross-cultural communication and understanding.	1 ✓	2	3	4	5
13. Somehow AI-generated content may unintentionally offend and misled individuals from specific cultural backgrounds.	1	2 ✓	3	4	5
14. Ethical considerations should be taken into account when designing AI-powered communication tools that cater to diverse cultural contexts.	1 ✓	2	3	4	5
15. Universities and educational institutions leverage AI to create more inclusive and culturally diverse online learning environments.	1 ✓	2	3	4	5

In the case of your agreement on sharing your answer/ points of views for questions below, please write down your comment/ point of view for all questions or any question that you prefer:

1. Did you experience the use of AI and AI-powered chatbots, such as ChatGPT and for what purpose?

   Yes. use AI for assignments, (ChatGPT) but just for give me a logic content.

2. How do you perceive the role of AI and AI-powered machines in bridging communication gaps between people from different cultures?

   ~~Tras Transla~~ Translation.

CamScanner

No (54) E PB

3. Have you experienced and observed any instances where AI has been successful in facilitating cross-cultural understanding and collaboration in mediated communication?

No.

4. In what ways do you think AI can help overcome language barriers and promote more inclusive communication among diverse groups?

X

5. Are there any cultural biases or stereotypes that you have noticed in AI-powered language tools or communication platforms?

X.

6. How can universities and educational institutions leverage AI to create more inclusive and culturally diverse online learning environments?

Creating a new platform.

Thank you for your time and participation

CS CamScanner

Partcipant No (55) E P (1)

UNIVERSITI MALAYA

Depart of Media and Communication Studies

AI and Mediated Intercultural Communication Questionnaire

Dear UM Student/ Researcher,

Through this survey, we want to assess the probable effects of Artificial Intelligence (AI) on mediated communication among people from different cultures. This instrument which been developed by the help of the existing literature and ChatGPT, has 15 structured items with five (5) options per item and six (6) open-ended questions. Your participation in the survey is voluntary and the personal identifications of the participants will be treated as strictly confidential.

Thank you for your cooperation.

The researchers

A. Demographic Information

1. Gender: ☑ Male ☐ Female

2. Age: 27

3. Nationality: China.

4. Level of education: PHD student

5. AI/ ChatGPT experience: ☑ Yes ☐ No

B. Intercultural Sensitivity Scale

**Directions:** The current AI-ICC questionnaire includes 15 items/ statements with five (5) options per item, as: 1 – Strongly agree; 2 – Agree, 3 – Uncertain; 2 – Disagree, and 5 – Strongly disagree.	Strongly agree	Agree	Uncertain	Disagree	Strongly disagree
1. AI and AI-powered machines can be helpful in bridging communication gaps between people from different cultures.	1	2 ✓	3	4	5
2. So far, AI has been successful in facilitating cross-cultural understanding and collaboration in mediated communication.	1	2	3 ✓	4	5
3. AI can help overcome language barriers and promote more inclusive communication among diverse groups.	1	2 ✓	3	4	5

No (55) & P2

4. There some cultural biases and stereotypes in AI-powered language tools and communication platforms.	1	2	3 ✓	4	5
5. AI has affected the localization and adaptation of content for different cultural audiences in digital communication.	1	2 ✓	3	4	5
6. I encountered any challenges in using AI-powered translation tools when communicating with people from different cultural backgrounds.	1	2	3 ✓	4	5
7. AI has the potential to promote cultural exchange and appreciation by enabling easier access to diverse perspectives and information.	1 ✓	2	3	4	5
8. AI-powered machines and chatbots should be programmed to respect and adapt to cultural norms and communication styles while interacting with users from different cultures.	1	2 ✓	3	4	5
9. There are some potential risks and concerns regarding AI's role in cross-cultural communication, such as perpetuating cultural stereotypes and misinterpretations.	1	2 ✓	3	4	5
10. I have participated in some cross-cultural virtual collaborations that relied on AI for communication and coordination.	1	2	3	4 ✓	5
11. I think AI can enhance intercultural learning experiences and foster empathy among individuals from different cultural backgrounds.	1	2 ✓	3	4	5
12. AI-powered virtual reality (VR) and augmented reality (AR) technologies can impact cross-cultural communication and understanding.	1	2 ✓	3	4	5
13. Somehow AI-generated content may unintentionally offend and misled individuals from specific cultural backgrounds.	1	2 ✓	3	4	5
14. Ethical considerations should be taken into account when designing AI-powered communication tools that cater to diverse cultural contexts.	1	2	3 ✓	4	5
15. Universities and educational institutions leverage AI to create more inclusive and culturally diverse online learning environments.	1	2 ✓	3	4	5

In the case of your agreement on sharing your answer/ points of views for questions below, please write down your comment/ point of view for all questions or any question that you prefer:

1. Did you experience the use of AI and AI-powered chatbots, such as ChatGPT and for what purpose?

   Chat GPT Get some information

2. How do you perceive the role of AI and AI-powered machines in bridging communication gaps between people from different cultures?

   AI maybe can help people get some information about other cultures or other ~~ethic~~ races

CS CamScanner

No 55 5/6 P3

3. Have you experienced and observed any instances where AI has been successful in facilitating cross-cultural understanding and collaboration in mediated communication?

Sorry. I dont know

4. In what ways do you think AI can help overcome language barriers and promote more inclusive communication among diverse groups?

Communicate with different people who from different races and backg

5. Are there any cultural biases or stereotypes that you have noticed in AI-powered language tools or communication platforms?

6. How can universities and educational institutions leverage AI to create more inclusive and culturally diverse online learning environments?

universities may need create a platforms or

Thank you for your time and participation

Somethings like that

CS CamScanner

Participant (56) & P(1)

UNIVERSITI MALAYA

Depart of Media and Communication Studies

AI and Mediated Intercultural Communication Questionnaire

Dear UM Student/ Researcher,

Through this survey, we want to assess the probable effects of Artificial Intelligence (AI) on mediated communication among people from different cultures. This instrument which been developed by the help of the existing literature and ChatGPT, has 15 structured items with five (5) options per item and six (6) open-ended questions. Your participation in the survey is voluntary and the personal identifications of the participants will be treated as strictly confidential.

Thank you for your cooperation.

The researchers

A. Demographic Information

1. Gender: ☐ Male ☑ Female

2. Age: 47

3. Nationality: Malaysian

4. Level of education: PhD

5. AI/ ChatGPT experience: ☑ Yes ☐ No

B. Intercultural Sensitivity Scale

**Directions:** The current AI-ICC questionnaire includes 15 items/ statements with five (5) options per item, as: 1 – Strongly agree; 2 – Agree, 3 – Uncertain; 2 – Disagree, and 5 – Strongly disagree.	Strongly agree	Agree	Uncertain	Disagree	Strongly disagree
1. AI and AI-powered machines can be helpful in bridging communication gaps between people from different cultures.	1	(2)	3	4	5
2. So far, AI has been successful in facilitating cross-cultural understanding and collaboration in mediated communication.	1	(2)	3	4	5
3. AI can help overcome language barriers and promote more inclusive communication among diverse groups.	1	2	(3)	4	5

CamScanner

No (56) & PC

4. There some cultural biases and stereotypes in AI-powered language tools and communication platforms.	1	2	(3)	4	5
5. AI has affected the localization and adaptation of content for different cultural audiences in digital communication.	1	2	(3)	4	5
6. I encountered any challenges in using AI-powered translation tools when communicating with people from different cultural backgrounds.	1	2	(3)	4	5
7. AI has the potential to promote cultural exchange and appreciation by enabling easier access to diverse perspectives and information.	1	(2)	3	4	5
8. AI-powered machines and chatbots should be programmed to respect and adapt to cultural norms and communication styles while interacting with users from different cultures.	(1)	2	3	4	5
9. There are some potential risks and concerns regarding AI's role in cross-cultural communication, such as perpetuating cultural stereotypes and misinterpretations.	1	(2)	3	4	5
10. I have participated in some cross-cultural virtual collaborations that relied on AI for communication and coordination.	1	2	3	(4)	5
11. I think AI can enhance intercultural learning experiences and foster empathy among individuals from different cultural backgrounds.	(1)	2	3	4	5
12. AI-powered virtual reality (VR) and augmented reality (AR) technologies can impact cross-cultural communication and understanding.	1	(2)	3	4	5
13. Somehow AI-generated content may unintentionally offend and misled individuals from specific cultural backgrounds.	1	(2)	3	4	5
14. Ethical considerations should be taken into account when designing AI-powered communication tools that cater to diverse cultural contexts.	(1)	2	3	4	5
15. Universities and educational institutions leverage AI to create more inclusive and culturally diverse online learning environments.	1	(2)	3	4	5

In the case of your agreement on sharing your answer/ points of views for questions below, please write down your comment/ point of view for all questions or any question that you prefer:

1. Did you experience the use of AI and AI-powered chatbots, such as ChatGPT and for what purpose?

I have tried to use it but only for personal use to see how well the AI works.

2. How do you perceive the role of AI and AI-powered machines in bridging communication gaps between people from different cultures?

Im not sure how well AI can bridging comm gaps between people from different cultures.

CS CamScanner

No (56) E, P(3)

3. Have you experienced and observed any instances where AI has been successful in facilitating cross-cultural understanding and collaboration in mediated communication?

No

4. In what ways do you think AI can help overcome language barriers and promote more inclusive communication among diverse groups?

Not sure.

5. Are there any cultural biases or stereotypes that you have noticed in AI-powered language tools or communication platforms?

Not sure.

6. How can universities and educational institutions leverage AI to create more inclusive and culturally diverse online learning environments?

University & educational institutions need to do a research in addition to understand the role & the ability of AI to change the learning environment in positive ways.

Thank you for your time and participation

CS CamScanner

Partcipant No (57) & P(1)

UNIVERSITI
MALAYA

Depart of Media and Communication Studies

AI and Mediated Intercultural Communication Questionnaire

Dear UM Student/ Researcher,

Through this survey, we want to assess the probable effects of Artificial Intelligence (AI) on mediated communication among people from different cultures. This instrument which been developed by the help of the existing literature and ChatGPT, has 15 structured items with five (5) options per item and six (6) open-ended questions. Your participation in the survey is voluntary and the personal identifications of the participants will be treated as strictly confidential.

Thank you for your cooperation.

The researchers

A. Demographic Information

1. Gender: ☑ Male ☐ Female

2. Age: 25

3. Nationality: Malaysian

4. Level of education: MD

5. AI/ ChatGPT experience: ☑ Yes ☐ No

B. Intercultural Sensitivity Scale

**Directions:** The current AI-ICC questionnaire includes 15 items/ statements with five (5) options per item, as: 1 – Strongly agree; 2 – Agree, 3 – Uncertain; 4 – Disagree, and 5 – Strongly disagree.	Strongly agree	Agree	Uncertain	Disagree	Strongly disagree
1. AI and AI-powered machines can be helpful in bridging communication gaps between people from different cultures.	1 ✓	2	3	4	5
2. So far, AI has been successful in facilitating cross-cultural understanding and collaboration in mediated communication.	1 ✓	2	3	4	5
3. AI can help overcome language barriers and promote more inclusive communication among diverse groups.	1 ✓	2	3	4	5

CamScanner

No (57) E, PO

4. There some cultural biases and stereotypes in AI-powered language tools and communication platforms.	1 ✓	2	3	4	5
5. AI has affected the localization and adaptation of content for different cultural audiences in digital communication.	1 ✓	2	3	4	5
6. I encountered any challenges in using AI-powered translation tools when communicating with people from different cultural backgrounds.	1	2	3 ✓	4	5
7. AI has the potential to promote cultural exchange and appreciation by enabling easier access to diverse perspectives and information.	1 ✓	2	3	4	5
8. AI-powered machines and chatbots should be programmed to respect and adapt to cultural norms and communication styles while interacting with users from different cultures.	1 ✓	2	3	4	5
9. There are some potential risks and concerns regarding AI's role in cross-cultural communication, such as perpetuating cultural stereotypes and misinterpretations.	1	2	3 ✓	4	5
10. I have participated in some cross-cultural virtual collaborations that relied on AI for communication and coordination.	1	2	3	4	5 ✓
11. I think AI can enhance intercultural learning experiences and foster empathy among individuals from different cultural backgrounds.	1	2 ✓	3	4	5
12. AI-powered virtual reality (VR) and augmented reality (AR) technologies can impact cross-cultural communication and understanding.	1	2	3	4 ✓	5
13. Somehow AI-generated content may unintentionally offend and misled individuals from specific cultural backgrounds.	1	2	3	4 ✓	5
14. Ethical considerations should be taken into account when designing AI-powered communication tools that cater to diverse cultural contexts.	1 ✓	2	3	4	5
15. Universities and educational institutions leverage AI to create more inclusive and culturally diverse online learning environments.	1 ✓	2	3	4	5

In the case of your agreement on sharing your answer/ points of views for questions below, please write down your comment/ point of view for all questions or any question that you prefer:

1. Did you experience the use of AI and AI-powered chatbots, such as ChatGPT and for what purpose?

   Yes, article writing

2. How do you perceive the role of AI and AI-powered machines in bridging communication gaps between people from different cultures?

   No experience

CamScanner

No (57) E1 P3

3. Have you experienced and observed any instances where AI has been successful in facilitating cross-cultural understanding and collaboration in mediated communication?

No

4. In what ways do you think AI can help overcome language barriers and promote more inclusive communication among diverse groups?

AI couldn't have emotion

5. Are there any cultural biases or stereotypes that you have noticed in AI-powered language tools or communication platforms?

Maybe

6. How can universities and educational institutions leverage AI to create more inclusive and culturally diverse online learning environments?

Depends on how the user prompt his/her question.

Thank you for your time and participation

CamScanner

Participant no (58) & P(1)

UNIVERSITI
MALAYA

Depart of Media and Communication Studies

AI and Mediated Intercultural Communication Questionnaire

Dear UM Student/ Researcher,

Through this survey, we want to assess the probable effects of Artificial Intelligence (AI) on mediated communication among people from different cultures. This instrument which been developed by the help of the existing literature and ChatGPT, has 15 structured items with five (5) options per item and six (6) open-ended questions. Your participation in the survey is voluntary and the personal identifications of the participants will be treated as strictly confidential.

Thank you for your cooperation.

The researchers

A. Demographic Information

1. Gender: ☑ Male ☐ Female

2. Age: 28

3. Nationality: CHINESE

4. Level of education: 研一 student. MA

5. AI/ ChatGPT experience: ☑ Yes ☐ No

B. Intercultural Sensitivity Scale

Directions: The current AI-ICC questionnaire includes 15 items/ statements with five (5) options per item, as: 1 – Strongly agree; 2 – Agree, 3 – Uncertain; 4 – Disagree, and 5 – Strongly disagree.	Strongly agree	Agree	Uncertain	Disagree	Strongly disagree
1. AI and AI-powered machines can be helpful in bridging communication gaps between people from different cultures.	✓	2	3	4	5
2. So far, AI has been successful in facilitating cross-cultural understanding and collaboration in mediated communication.	✓	2	3	4	5
3. AI can help overcome language barriers and promote more inclusive communication among diverse groups.	✓	2	3	4	5

CS CamScanner

No (58) [illegible]

4. There some cultural biases and stereotypes in AI-powered language tools and communication platforms.	1 ✓	2	3	4	5
5. AI has affected the localization and adaptation of content for different cultural audiences in digital communication.	1 ✓	2	3	4	5
6. I encountered any challenges in using AI-powered translation tools when communicating with people from different cultural backgrounds.	1	2	3 ✓	4	5
7. AI has the potential to promote cultural exchange and appreciation by enabling easier access to diverse perspectives and information.	1 ✓	2	3	4	5
8. AI-powered machines and chatbots should be programmed to respect and adapt to cultural norms and communication styles while interacting with users from different cultures.	1 ✓	2	3	4	5
9. There are some potential risks and concerns regarding AI's role in cross-cultural communication, such as perpetuating cultural stereotypes and misinterpretations.	1	2	3	4	5 ✓
10. I have participated in some cross-cultural virtual collaborations that relied on AI for communication and coordination.	1	2	3	4	5 ✓
11. I think AI can enhance intercultural learning experiences and foster empathy among individuals from different cultural backgrounds.	1	2	3	4 ✓	5
12. AI-powered virtual reality (VR) and augmented reality (AR) technologies can impact cross-cultural communication and understanding.	1	2	3 ✓	4	5
13. Somehow AI-generated content may unintentionally offend and misled individuals from specific cultural backgrounds.	1 ✓	2	3	4	5
14. Ethical considerations should be taken into account when designing AI-powered communication tools that cater to diverse cultural contexts.	1 ✓	2	3	4	5
15. Universities and educational institutions leverage AI to create more inclusive and culturally diverse online learning environments.	1 ✓	2	3	4	5

In the case of your agreement on sharing your answer/ points of views for questions below, please write down your comment/ point of view for all questions or any question that you prefer:

1. Did you experience the use of AI and AI-powered chatbots, such as ChatGPT and for what purpose?

   Yes, literature reviews

2. How do you perceive the role of AI and AI-powered machines in bridging communication gaps between people from different cultures?

   Good

CS CamScanner

No (58) 乙 P(3)

3. Have you experienced and observed any instances where AI has been successful in facilitating cross-cultural understanding and collaboration in mediated communication?

Yes.

4. In what ways do you think AI can help overcome language barriers and promote more inclusive communication among diverse groups?

Somehow can provide us choices.

But. hard to [illegible] bring our emotion involved.

5. Are there any cultural biases or stereotypes that you have noticed in AI-powered language tools or communication platforms?

No,. any thing facilitate our - job / live

Should be ~~[illegible]~~ encouraged.

6. How can universities and educational institutions leverage AI to create more inclusive and culturally diverse online learning environments?

Depend on how the ~~leadership~~ leader use it.

Thank you for your time and participation

CS CamScanner

Participant No (59) Er PD

UNIVERSITI MALAYA

Depart of Media and Communication Studies

AI and Mediated Intercultural Communication Questionnaire

Dear UM Student/ Researcher,

Through this survey, we want to assess the probable effects of Artificial Intelligence (AI) on mediated communication among people from different cultures. This instrument which been developed by the help of the existing literature and ChatGPT, has 15 structured items with five (5) options per item and six (6) open-ended questions. Your participation in the survey is voluntary and the personal identifications of the participants will be treated as strictly confidential.

Thank you for your cooperation.

The researchers

A. Demographic Information

1. Gender: ☐ Male ✓ Female

2. Age: 30

3. Nationality: China

4. Level of education: PHD

5. AI/ ChatGPT experience: ✓ Yes ☐ No

B. Intercultural Sensitivity Scale

Directions: The current AI-ICC questionnaire includes 15 items/ statements with five (5) options per item, as: 1 – Strongly agree; 2 – Agree, 3 – Uncertain; 4 – Disagree, and 5 – Strongly disagree.	Strongly agree	Agree	Uncertain	Disagree	Strongly disagree
1. AI and AI-powered machines can be helpful in bridging communication gaps between people from different cultures.	✓	2	3	4	5
2. So far, AI has been successful in facilitating cross-cultural understanding and collaboration in mediated communication.	1 ✓	2	3	4	5
3. AI can help overcome language barriers and promote more inclusive communication among diverse groups.	1 ✓	2	3	4	5

CamScanner

No (59) Ec Pe

	1	2	3	4	5
4. There some cultural biases and stereotypes in AI-powered language tools and communication platforms.	1 ✓	2	3	4	5
5. AI has affected the localization and adaptation of content for different cultural audiences in digital communication.	1	2 ✓	3	4	5
6. I encountered any challenges in using AI-powered translation tools when communicating with people from different cultural backgrounds.	1	2 ✓	3	4	5
7. AI has the potential to promote cultural exchange and appreciation by enabling easier access to diverse perspectives and information.	1 ✓	2	3	4	5
8. AI-powered machines and chatbots should be programmed to respect and adapt to cultural norms and communication styles while interacting with users from different cultures.	1 ✓	2	3	4	5
9. There are some potential risks and concerns regarding AI's role in cross-cultural communication, such as perpetuating cultural stereotypes and misinterpretations.	1 ✓	2	3	4	5
10. I have participated in some cross-cultural virtual collaborations that relied on AI for communication and coordination.	1	2	3	4	5 ✓
11. I think AI can enhance intercultural learning experiences and foster empathy among individuals from different cultural backgrounds.	1 ✓	2	3	4	5
12. AI-powered virtual reality (VR) and augmented reality (AR) technologies can impact cross-cultural communication and understanding.	1 ✓	2	3	4	5
13. Somehow AI-generated content may unintentionally offend and misled individuals from specific cultural backgrounds.	1 ✓	2	3	4	5
14. Ethical considerations should be taken into account when designing AI-powered communication tools that cater to diverse cultural contexts.	1 ✓	2	3	4	5
15. Universities and educational institutions leverage AI to create more inclusive and culturally diverse online learning environments.	1 ✓	2	3	4	5

In the case of your agreement on sharing your answer/ points of views for questions below, please write down your comment/ point of view for all questions or any question that you prefer:

1. Did you experience the use of AI and AI-powered chatbots, such as ChatGPT and for what purpose?

   For more convenient use of some functions.

2. How do you perceive the role of AI and AI-powered machines in bridging communication gaps between people from different cultures?

   It is necessary to strengthen communication and understanding

CS CamScanner

No (59) E P(3)

3. Have you experienced and observed any instances where AI has been successful in facilitating cross-cultural understanding and collaboration in mediated communication?

No. I havn't.

4. In what ways do you think AI can help overcome language barriers and promote more inclusive communication among diverse groups?

Maybe technology develops, and then AI can have the same logic.

5. Are there any cultural biases or stereotypes that you have noticed in AI-powered language tools or communication platforms?

~~No.~~ I pay no attention to that.

6. How can universities and educational institutions leverage AI to create more inclusive and culturally diverse online learning environments?

I don't know.

Thank you for your time and participation

CS CamScanner

Participant No (60) & P(1)

UNIVERSITI MALAYA

Depart of Media and Communication Studies

AI and Mediated Intercultural Communication Questionnaire

Dear UM Student/ Researcher,

Through this survey, we want to assess the probable effects of Artificial Intelligence (AI) on mediated communication among people from different cultures. This instrument which been developed by the help of the existing literature and ChatGPT, has 15 structured items with five (5) options per item and six (6) open-ended questions. Your participation in the survey is voluntary and the personal identifications of the participants will be treated as strictly confidential.

Thank you for your cooperation.

The researchers

A. Demographic Information

1. Gender: ☑ Male ☐ Female

2. Age: 27

3. Nationality: China

4. Level of education: master

5. AI/ ChatGPT experience: ☑ Yes ☐ No

B. Intercultural Sensitivity Scale

Directions: The current AI-ICC questionnaire includes 15 items/ statements with five (5) options per item, as: 1 – Strongly agree; 2 – Agree, 3 – Uncertain; 2 4 – Disagree, and 5 – Strongly disagree.	Strongly agree	Agree	Uncertain	Disagree	Strongly disagree
1. AI and AI-powered machines can be helpful in bridging communication gaps between people from different cultures.	1 ✓	2	3	4	5
2. So far, AI has been successful in facilitating cross-cultural understanding and collaboration in mediated communication.	1	2 ✓	3	4	5
3. AI can help overcome language barriers and promote more inclusive communication among diverse groups.	1	2 ✓	3	4	5

CamScanner

No (60) E P(2)

4. There some cultural biases and stereotypes in AI-powered language tools and communication platforms.	1	2	3 ✓	4	5
5. AI has affected the localization and adaptation of content for different cultural audiences in digital communication.	1	2 ✓	3	4	5
6. I encountered any challenges in using AI-powered translation tools when communicating with people from different cultural backgrounds.	1	2 ✓	3	4	5
7. AI has the potential to promote cultural exchange and appreciation by enabling easier access to diverse perspectives and information.	1 ✓	2	3	4	5
8. AI-powered machines and chatbots should be programmed to respect and adapt to cultural norms and communication styles while interacting with users from different cultures.	1 ✓	2	3	4	5
9. There are some potential risks and concerns regarding AI's role in cross-cultural communication, such as perpetuating cultural stereotypes and misinterpretations.	1	2 ✓	3	4	5
10. I have participated in some cross-cultural virtual collaborations that relied on AI for communication and coordination.	1	2 ✓	3	4	5
11. I think AI can enhance intercultural learning experiences and foster empathy among individuals from different cultural backgrounds.	1 ✓	2	3	4	5
12. AI-powered virtual reality (VR) and augmented reality (AR) technologies can impact cross-cultural communication and understanding.	1	2 ✓	3	4	5
13. Somehow AI-generated content may unintentionally offend and misled individuals from specific cultural backgrounds.	1	2	3 ✓	4	5
14. Ethical considerations should be taken into account when designing AI-powered communication tools that cater to diverse cultural contexts.	1 ✓	2	3	4	5
15. Universities and educational institutions leverage AI to create more inclusive and culturally diverse online learning environments.	1	2 ✓	3	4	5

In the case of your agreement on sharing your answer/ points of views for questions below, please write down your comment/ point of view for all questions or any question that you prefer:

1. Did you experience the use of AI and AI-powered chatbots, such as ChatGPT and for what purpose?

   Yes, I did. I used ChatGPT to translate papers into Chinese, write a short essay and a sentence with modifiers.

2. How do you perceive the role of AI and AI-powered machines in bridging communication gaps between people from different cultures?

   A good tool.

CamScanner

No (60) E, P(3)

3. Have you experienced and observed any instances where AI has been successful in facilitating cross-cultural understanding and collaboration in mediated communication?

No. attention.

4. In what ways do you think AI can help overcome language barriers and promote more inclusive communication among diverse groups?

No attention.

5. Are there any cultural biases or stereotypes that you have noticed in AI-powered language tools or communication platforms?

No attention.

6. How can universities and educational institutions leverage AI to create more inclusive and culturally diverse online learning environments?

No attention

Thank you for your time and participation

CamScanner

Participant No (61) & P(1)

UNIVERSITI MALAYA

Depart of Media and Communication Studies

AI and Mediated Intercultural Communication Questionnaire

Dear UM Student/ Researcher,

Through this survey, we want to assess the probable effects of Artificial Intelligence (AI) on mediated communication among people from different cultures. This instrument which been developed by the help of the existing literature and ChatGPT, has 15 structured items with five (5) options per item and six (6) open-ended questions. Your participation in the survey is voluntary and the personal identifications of the participants will be treated as strictly confidential.

Thank you for your cooperation.

The researchers

A. Demographic Information

1. Gender: ☐ Male ✓ Female

2. Age: 31

3. Nationality: China

4. Level of education: Phd

5. AI/ ChatGPT experience: ✓Yes ☐ No

B. Intercultural Sensitivity Scale

**Directions:** The current AI-ICC questionnaire includes 15 items/ statements with five (5) options per item, as: 1 – Strongly agree; 2 – Agree, 3 – Uncertain; 4 – Disagree, and 5 – Strongly disagree.	Strongly agree	Agree	Uncertain	Disagree	Strongly disagree
1. AI and AI-powered machines can be helpful in bridging communication gaps between people from different cultures.	1	2	3 ✓	4	5
2. So far, AI has been successful in facilitating cross-cultural understanding and collaboration in mediated communication.	1	2	3 ✓	4	5
3. AI can help overcome language barriers and promote more inclusive communication among diverse groups.	1	2 ✓	3	4	5

CamScanner

No (61) & P(2)

4. There some cultural biases and stereotypes in AI-powered language tools and communication platforms.	1	2 ✓	3	4	5
5. AI has affected the localization and adaptation of content for different cultural audiences in digital communication.	1	2 ✓	3	4	5
6. I encountered any challenges in using AI-powered translation tools when communicating with people from different cultural backgrounds.	1 ✓	2	3	4	5
7. AI has the potential to promote cultural exchange and appreciation by enabling easier access to diverse perspectives and information.	1 ✓	2	3	4	5
8. AI-powered machines and chatbots should be programmed to respect and adapt to cultural norms and communication styles while interacting with users from different cultures.	1 ✓	2	3	4	5
9. There are some potential risks and concerns regarding AI's role in cross-cultural communication, such as perpetuating cultural stereotypes and misinterpretations.	1 ✓	2	3	4	5
10. I have participated in some cross-cultural virtual collaborations that relied on AI for communication and coordination.	1	2	3	4 ✓	5
11. I think AI can enhance intercultural learning experiences and foster empathy among individuals from different cultural backgrounds.	1	2 ✓	3	4	5
12. AI-powered virtual reality (VR) and augmented reality (AR) technologies can impact cross-cultural communication and understanding.	1	2 ✓	3	4	5
13. Somehow AI-generated content may unintentionally offend and misled individuals from specific cultural backgrounds.	1 ✓	2	3	4	5
14. Ethical considerations should be taken into account when designing AI-powered communication tools that cater to diverse cultural contexts.	1 ✓	2	3	4	5
15. Universities and educational institutions leverage AI to create more inclusive and culturally diverse online learning environments.	1	2 ✓	3	4	5

In the case of your agreement on sharing your answer/ points of views for questions below, please write down your comment/ point of view for all questions or any question that you prefer:

1. Did you experience the use of AI and AI-powered chatbots, such as ChatGPT and for what purpose?

   In order to write my thesis and drawing.

2. How do you perceive the role of AI and AI-powered machines in bridging communication gaps between people from different cultures?

   I have no idea.

CS CamScanner

No (61) & P (3)

3. Have you experienced and observed any instances where AI has been successful in facilitating cross-cultural understanding and collaboration in mediated communication?

No

4. In what ways do you think AI can help overcome language barriers and promote more inclusive communication among diverse groups?

I don't know.

5. Are there any cultural biases or stereotypes that you have noticed in AI-powered language tools or communication platforms?

did not notice.

6. How can universities and educational institutions leverage AI to create more inclusive and culturally diverse online learning environments?

learn / Enter different cultures.

Thank you for your time and participation

CS CamScanner

Participant No 62 & P11

UNIVERSITI MALAYA

Depart of Media and Communication Studies

AI and Mediated Intercultural Communication Questionnaire

Dear UM Student/ Researcher,

Through this survey, we want to assess the probable effects of Artificial Intelligence (AI) on mediated communication among people from different cultures. This instrument which been developed by the help of the existing literature and ChatGPT, has 15 structured items with five (5) options per item and six (6) open-ended questions. Your participation in the survey is voluntary and the personal identifications of the participants will be treated as strictly confidential.

Thank you for your cooperation.

The researchers

A. Demographic Information

1. Gender: ☒ Male ☐ Female

2. Age: 28

3. Nationality: Malay

4. Level of education: ~~Foundation~~ of Social Science post-degree grad

5. AI/ ChatGPT experience: ☒ Yes ☐ No

B. Intercultural Sensitivity Scale

Directions: The current AI-ICC questionnaire includes 15 items/ statements with five (5) options per item, as: 1 – Strongly agree; 2 – Agree, 3 – Uncertain; 4 – Disagree, and 5 – Strongly disagree.	Strongly agree	Agree	Uncertain	Disagree	Strongly disagree
1. AI and AI-powered machines can be helpful in bridging communication gaps between people from different cultures.	(1)	2	3	4	5
2. So far, AI has been successful in facilitating cross-cultural understanding and collaboration in mediated communication.	(1)	2	3	4	5
3. AI can help overcome language barriers and promote more inclusive communication among diverse groups.	(1)	2	3	4	5

CamScanner

No (62) E (2)

4. There some cultural biases and stereotypes in AI-powered language tools and communication platforms.	1	(2)	3	4	5
5. AI has affected the localization and adaptation of content for different cultural audiences in digital communication.	1	(2)	3	4	5
6. I encountered any challenges in using AI-powered translation tools when communicating with people from different cultural backgrounds.	1	2	(3)	4	5
7. AI has the potential to promote cultural exchange and appreciation by enabling easier access to diverse perspectives and information.	(1)	2	3	4	5
8. AI-powered machines and chatbots should be programmed to respect and adapt to cultural norms and communication styles while interacting with users from different cultures.	(1)	2	3	4	5
9. There are some potential risks and concerns regarding AI's role in cross-cultural communication, such as perpetuating cultural stereotypes and misinterpretations.	1	2	(3)	4	5
10. I have participated in some cross-cultural virtual collaborations that relied on AI for communication and coordination.	(1)	2	3	4	5
11. I think AI can enhance intercultural learning experiences and foster empathy among individuals from different cultural backgrounds.	(1)	2	3	4	5
12. AI-powered virtual reality (VR) and augmented reality (AR) technologies can impact cross-cultural communication and understanding.	(1)	2	3	4	5
13. Somehow AI-generated content may unintentionally offend and misled individuals from specific cultural backgrounds.	1	(2)	3	4	5
14. Ethical considerations should be taken into account when designing AI-powered communication tools that cater to diverse cultural contexts.	1	2	(3)	4	5
15. Universities and educational institutions leverage AI to create more inclusive and culturally diverse online learning environments.	(1)	2	3	4	5

In the case of your agreement on sharing your answer/ points of views for questions below, please write down your comment/ point of view for all questions or any question that you prefer:

1. Did you experience the use of AI and AI-powered chatbots, such as ChatGPT and for what purpose?

   Yes i am, the purpose of it is to do more research about certain topic that i learn.

2. How do you perceive the role of AI and AI-powered machines in bridging communication gaps between people from different cultures?

   Communication application such as google meet, zoom, discord and else.

No (62) E PB

3. Have you experienced and observed any instances where AI has been successful in facilitating cross-cultural understanding and collaboration in mediated communication?

Not yet

4. In what ways do you think AI can help overcome language barriers and promote more inclusive communication among diverse groups?

quick access to translate acces

5. Are there any cultural biases or stereotypes that you have noticed in AI-powered language tools or communication platforms?

No

6. How can universities and educational institutions leverage AI to create more inclusive and culturally diverse online learning environments?

Make their own education websith

Thank you for your time and participation

CamScanner

Participant No 63 & No P0

UNIVERSITI MALAYA

Depart of Media and Communication Studies

AI and Mediated Intercultural Communication Questionnaire

Dear UM Student/ Researcher,

Through this survey, we want to assess the probable effects of Artificial Intelligence (AI) on mediated communication among people from different cultures. This instrument which been developed by the help of the existing literature and ChatGPT, has 15 structured items with five (5) options per item and six (6) open-ended questions. Your participation in the survey is voluntary and the personal identifications of the participants will be treated as strictly confidential.

Thank you for your cooperation.

The researchers

A. Demographic Information

1. Gender: ☑ Male ☐ Female

2. Age: 34

3. Nationality: Malaysian.

4. Level of education: ~~Ba~~ Master Degree (MASTER)

5. AI/ ChatGPT experience: ☑ Yes ☐ No

B. Intercultural Sensitivity Scale

Directions: The current AI-ICC questionnaire includes 15 items/ statements with five (5) options per item, as: 1 – Strongly agree; 2 – Agree, 3 – Uncertain; 4 – Disagree, and 5 – Strongly disagree.	Strongly agree	Agree	Uncertain	Disagree	Strongly disagree
1. AI and AI-powered machines can be helpful in bridging communication gaps between people from different cultures.	1	(2)	3	4	5
2. So far, AI has been successful in facilitating cross-cultural understanding and collaboration in mediated communication.	1	2	(3)	4	5
3. AI can help overcome language barriers and promote more inclusive communication among diverse groups.	1	(2)	3	4	5

CamScanner

No (63) & P(2)

4. There some cultural biases and stereotypes in AI-powered language tools and communication platforms.	1	2	3	4	5
5. AI has affected the localization and adaptation of content for different cultural audiences in digital communication.	1	2	3	4	5
6. I encountered any challenges in using AI-powered translation tools when communicating with people from different cultural backgrounds.	1	2	3	4	5
7. AI has the potential to promote cultural exchange and appreciation by enabling easier access to diverse perspectives and information.	1	2	3	4	5
8. AI-powered machines and chatbots should be programmed to respect and adapt to cultural norms and communication styles while interacting with users from different cultures.	1	2	3	4	5
9. There are some potential risks and concerns regarding AI's role in cross-cultural communication, such as perpetuating cultural stereotypes and misinterpretations.	1	2	3	4	5
10. I have participated in some cross-cultural virtual collaborations that relied on AI for communication and coordination.	1	2	3	4	5
11. I think AI can enhance intercultural learning experiences and foster empathy among individuals from different cultural backgrounds.	1	2	3	4	5
12. AI-powered virtual reality (VR) and augmented reality (AR) technologies can impact cross-cultural communication and understanding.	1	2	3	4	5
13. Somehow AI-generated content may unintentionally offend and misled individuals from specific cultural backgrounds.	1	2	3	4	5
14. Ethical considerations should be taken into account when designing AI-powered communication tools that cater to diverse cultural contexts.	1	2	3	4	5
15. Universities and educational institutions leverage AI to create more inclusive and culturally diverse online learning environments.	1	2	3	4	5

In the case of your agreement on sharing your answer/ points of views for questions below, please write down your comment/ point of view for all questions or any question that you prefer:

1. Did you experience the use of AI and AI-powered chatbots, such as ChatGPT and for what purpose?

Yes, to get some ideas / outline for assignments.

2. How do you perceive the role of AI and AI-powered machines in bridging communication gaps between people from different cultures?

Keep up to date on technology available at current market.

No (63) E P(2)

4. There some cultural biases and stereotypes in AI-powered language tools and communication platforms.	1	**2**	3	4	5
5. AI has affected the localization and adaptation of content for different cultural audiences in digital communication.	1	**2**	3	4	5
6. I encountered any challenges in using AI-powered translation tools when communicating with people from different cultural backgrounds.	1	2	3	**4**	5
7. AI has the potential to promote cultural exchange and appreciation by enabling easier access to diverse perspectives and information.	1	**2**	3	4	5
8. AI-powered machines and chatbots should be programmed to respect and adapt to cultural norms and communication styles while interacting with users from different cultures.	1	**2**	3	4	5
9. There are some potential risks and concerns regarding AI's role in cross-cultural communication, such as perpetuating cultural stereotypes and misinterpretations.	1	**2**	3	4	5
10. I have participated in some cross-cultural virtual collaborations that relied on AI for communication and coordination.	1	2	3	4	**5**
11. I think AI can enhance intercultural learning experiences and foster empathy among individuals from different cultural backgrounds.	1	**2**	3	4	5
12. AI-powered virtual reality (VR) and augmented reality (AR) technologies can impact cross-cultural communication and understanding.	1	**2**	3	4	5
13. Somehow AI-generated content may unintentionally offend and misled individuals from specific cultural backgrounds.	1	2	3	**4**	5
14. Ethical considerations should be taken into account when designing AI-powered communication tools that cater to diverse cultural contexts.	1	2	3	4	**5**
15. Universities and educational institutions leverage AI to create more inclusive and culturally diverse online learning environments.	1	**2**	3	4	5

In the case of your agreement on sharing your answer/ points of views for questions below, please write down your comment/ point of view for all questions or any question that you prefer:

1. Did you experience the use of AI and AI-powered chatbots, such as ChatGPT and for what purpose?

Yes, to get some ideas / outline for assignments.

2. How do you perceive the role of AI and AI-powered machines in bridging communication gaps between people from different cultures?

Keep up to date on technology available at current market.

Partcipant (64) & P(1)

UNIVERSITI MALAYA

Depart of Media and Communication Studies

AI and Mediated Intercultural Communication Questionnaire

Dear UM Student/ Researcher,

Through this survey, we want to assess the probable effects of Artificial Intelligence (AI) on mediated communication among people from different cultures. This instrument which been developed by the help of the existing literature and ChatGPT, has 15 structured items with five (5) options per item and six (6) open-ended questions. Your participation in the survey is voluntary and the personal identifications of the participants will be treated as strictly confidential.

Thank you for your cooperation.

The researchers

A. Demographic Information

1. Gender: ☐ Male ☑ Female

2. Age: 35

3. Nationality: Indonesia

4. Level of education: Ph.D.

5. AI/ ChatGPT experience: ☑ Yes ☐ No

B. Intercultural Sensitivity Scale

**Directions:** The current AI-ICC questionnaire includes 15 items/ statements with five (5) options per item, as: 1 – Strongly agree; 2 – Agree, 3 – Uncertain; 4 – Disagree, and 5 – Strongly disagree.	Strongly agree	Agree	Uncertain	Disagree	Strongly disagree
1. AI and AI-powered machines can be helpful in bridging communication gaps between people from different cultures.	1	(2)	3	4	5
2. So far, AI has been successful in facilitating cross-cultural understanding and collaboration in mediated communication.	1	(2)	3	4	5
3. AI can help overcome language barriers and promote more inclusive communication among diverse groups.	1	2	(3)	4	5

CamScanner

Partecipant No (64) & P(2)

4. There some cultural biases and stereotypes in AI-powered language tools and communication platforms.	1	2	(3)	4	5
5. AI has affected the localization and adaptation of content for different cultural audiences in digital communication.	1	(2)	3	4	5
6. I encountered any challenges in using AI-powered translation tools when communicating with people from different cultural backgrounds.	1	2	3	(4)	5
7. AI has the potential to promote cultural exchange and appreciation by enabling easier access to diverse perspectives and information.	1	2	(3)	4	5
8. AI-powered machines and chatbots should be programmed to respect and adapt to cultural norms and communication styles while interacting with users from different cultures.	1	(2)	3	4	5
9. There are some potential risks and concerns regarding AI's role in cross-cultural communication, such as perpetuating cultural stereotypes and misinterpretations.	1	(2)	3	4	5
10. I have participated in some cross-cultural virtual collaborations that relied on AI for communication and coordination.	1	2	3	(4)	5
11. I think AI can enhance intercultural learning experiences and foster empathy among individuals from different cultural backgrounds.	1	2	(3)	4	5
12. AI-powered virtual reality (VR) and augmented reality (AR) technologies can impact cross-cultural communication and understanding.	1	2	(3)	4	5
13. Somehow AI-generated content may unintentionally offend and misled individuals from specific cultural backgrounds.	1	2	(3)	4	5
14. Ethical considerations should be taken into account when designing AI-powered communication tools that cater to diverse cultural contexts.	1	(2)	3	4	5
15. Universities and educational institutions leverage AI to create more inclusive and culturally diverse online learning environments.	1	(2)	3	4	5

In the case of your agreement on sharing your answer/ points of views for questions below, please write down your comment/ point of view for all questions or any question that you prefer:

1. Did you experience the use of AI and AI-powered chatbots, such as ChatGPT and for what purpose?

   I used the AI for educational purposes

2. How do you perceive the role of AI and AI-powered machines in bridging communication gaps between people from different cultures?

   Not sure about this, because of my limited experience using AI

CS CamScanner

Participant No (65) & P0

UNIVERSITI MALAYA

Depart of Media and Communication Studies

AI and Mediated Intercultural Communication Questionnaire

Dear UM Student/ Researcher,

Through this survey, we want to assess the probable effects of Artificial Intelligence (AI) on mediated communication among people from different cultures. This instrument which been developed by the help of the existing literature and ChatGPT, has 15 structured items with five (5) options per item and six (6) open-ended questions. Your participation in the survey is voluntary and the personal identifications of the participants will be treated as strictly confidential.

Thank you for your cooperation.

The researchers

A. Demographic Information

1. Gender: ☐ Male ☑ Female

2. Age: 22

3. Nationality: Malaysian

4. Level of education: ~~tertiary education~~ post Grade degree.

5. AI/ ChatGPT experience: ☑ Yes ☐ No

B. Intercultural Sensitivity Scale

**Directions:** The current AI-ICC questionnaire includes 15 items/ statements with five (5) options per item, as: 1 – Strongly agree; 2 – Agree, 3 – Uncertain; 4 – Disagree, and 5 – Strongly disagree.	Strongly agree	Agree	Uncertain	Disagree	Strongly disagree
1. AI and AI-powered machines can be helpful in bridging communication gaps between people from different cultures.	1	(2)	3	4	5
2. So far, AI has been successful in facilitating cross-cultural understanding and collaboration in mediated communication.	1	(2)	3	4	5
3. AI can help overcome language barriers and promote more inclusive communication among diverse groups.	1	(2)	3	4	5

CamScanner

No (65) E P(2)

4. There some cultural biases and stereotypes in AI-powered language tools and communication platforms.	1	(2)	3	4	5
5. AI has affected the localization and adaptation of content for different cultural audiences in digital communication.	1	2	(3)	4	5
6. I encountered any challenges in using AI-powered translation tools when communicating with people from different cultural backgrounds.	1	2	(3)	4	5
7. AI has the potential to promote cultural exchange and appreciation by enabling easier access to diverse perspectives and information.	1	(2)	3	4	5
8. AI-powered machines and chatbots should be programmed to respect and adapt to cultural norms and communication styles while interacting with users from different cultures.	(1)	2	3	4	5
9. There are some potential risks and concerns regarding AI's role in cross-cultural communication, such as perpetuating cultural stereotypes and misinterpretations.	(1)	2	3	4	5
10. I have participated in some cross-cultural virtual collaborations that relied on AI for communication and coordination.	1	2	3	(4)	5
11. I think AI can enhance intercultural learning experiences and foster empathy among individuals from different cultural backgrounds.	1	(2)	3	4	5
12. AI-powered virtual reality (VR) and augmented reality (AR) technologies can impact cross-cultural communication and understanding.	1	(2)	3	4	5
13. Somehow AI-generated content may unintentionally offend and misled individuals from specific cultural backgrounds.	1	(2)	3	4	5
14. Ethical considerations should be taken into account when designing AI-powered communication tools that cater to diverse cultural contexts.	1	(2)	3	4	5
15. Universities and educational institutions leverage AI to create more inclusive and culturally diverse online learning environments.	(1)	2	3	4	5

In the case of your agreement on sharing your answer/ points of views for questions below, please write down your comment/ point of view for all questions or any question that you prefer:

1. Did you experience the use of AI and AI-powered chatbots, such as ChatGPT and for what purpose?

   Yes, for education purpose

2. How do you perceive the role of AI and AI-powered machines in bridging communication gaps between people from different cultures?

   They have a more neutral standing when providing information.

CamScanner

NO (65) E P3

3. Have you experienced and observed any instances where AI has been successful in facilitating cross-cultural understanding and collaboration in mediated communication?

No.

4. In what ways do you think AI can help overcome language barriers and promote more inclusive communication among diverse groups?

AI provides more opportunities & resources to enable communication between people from different background

5. Are there any cultural biases or stereotypes that you have noticed in AI-powered language tools or communication platforms?

most info provided are more western - oriented unless specify

6. How can universities and educational institutions leverage AI to create more inclusive and culturally diverse online learning environments?

would be nice to have AI classes introduced as university elective courses

Thank you for your time and participation

CamScanner

Participant (66) E P(1)

UNIVERSITI MALAYA

Depart of Media and Communication Studies

AI and Mediated Intercultural Communication Questionnaire

Dear UM Student/ Researcher,

Through this survey, we want to assess the probable effects of Artificial Intelligence (AI) on mediated communication among people from different cultures. This instrument which been developed by the help of the existing literature and ChatGPT, has 15 structured items with five (5) options per item and six (6) open-ended questions. Your participation in the survey is voluntary and the personal identifications of the participants will be treated as strictly confidential.

Thank you for your cooperation.

The researchers

A. Demographic Information

1. Gender: ☑ Male ☐ Female

2. Age: 22

3. Nationality: MALAYSIAN

4. Level of education: ~~BACHELOR / DEGREE~~ post-graduation

5. AI/ ChatGPT experience: ☑ Yes ☐ No

B. Intercultural Sensitivity Scale

**Directions:** The current AI-ICC questionnaire includes 15 items/ statements with five (5) options per item, as: 1 – Strongly agree; 2 – Agree, 3 – Uncertain; 4 – Disagree, and 5 – Strongly disagree.	Strongly agree	Agree	Uncertain	Disagree	Strongly disagree
1. AI and AI-powered machines can be helpful in bridging communication gaps between people from different cultures.	1	2 ✓	3	4	5
2. So far, AI has been successful in facilitating cross-cultural understanding and collaboration in mediated communication.	1	2	3 ✓	4	5
3. AI can help overcome language barriers and promote more inclusive communication among diverse groups.	1	~~2~~	3 ✓	4	5

CamScanner

No (66) E P(2)

4. There some cultural biases and stereotypes in AI-powered language tools and communication platforms.	1	2	3 ✓	4	5
5. AI has affected the localization and adaptation of content for different cultural audiences in digital communication.	1	2	3 ✓	4	5
6. I encountered any challenges in using AI-powered translation tools when communicating with people from different cultural backgrounds.	1	2	3	4 ✓	5
7. AI has the potential to promote cultural exchange and appreciation by enabling easier access to diverse perspectives and information.	1	2 ✓	3	4	5
8. AI-powered machines and chatbots should be programmed to respect and adapt to cultural norms and communication styles while interacting with users from different cultures.	1	2 ✓	3	4	5
9. There are some potential risks and concerns regarding AI's role in cross-cultural communication, such as perpetuating cultural stereotypes and misinterpretations.	1 ✓	2	3	4	5
10. I have participated in some cross-cultural virtual collaborations that relied on AI for communication and coordination.	1	2	3	4 ✓	5
11. I think AI can enhance intercultural learning experiences and foster empathy among individuals from different cultural backgrounds.	1	2 ✓	3	4	5
12. AI-powered virtual reality (VR) and augmented reality (AR) technologies can impact cross-cultural communication and understanding.	1	2	3 ✓	4	5
13. Somehow AI-generated content may unintentionally offend and misled individuals from specific cultural backgrounds.	1	2 ✓	3	4	5
14. Ethical considerations should be taken into account when designing AI-powered communication tools that cater to diverse cultural contexts.	1	2 ✓	3	4	5
15. Universities and educational institutions leverage AI to create more inclusive and culturally diverse online learning environments.	1	2 ✓	3	4	5

In the case of your agreement on sharing your answer/ points of views for questions below, please write down your comment/ point of view for all questions or any question that you prefer:

1. Did you experience the use of AI and AI-powered chatbots, such as ChatGPT and for what purpose?

Yes, for university assignments

2. How do you perceive the role of AI and AI-powered machines in bridging communication gaps between people from different cultures?

When inputting prompts that are offensive & misleading. AI-powered machines can step in & correct such misunderstanding.

CamScanner

No 67 E P3

3. Have you experienced and observed any instances where AI has been successful in facilitating cross-cultural understanding and collaboration in mediated communication?

No

4. In what ways do you think AI can help overcome language barriers and promote more inclusive communication among diverse groups?

AI can provide translation functions with better accuracies.

5. Are there any cultural biases or stereotypes that you have noticed in AI-powered language tools or communication platforms?

No, but I have seen reports of AI doing so.

6. How can universities and educational institutions leverage AI to create more inclusive and culturally diverse online learning environments?

AI can supplement the shortcomings of teachers if their knowledge on other cultures are lacking.

Thank you for your time and participation

CS CamScanner

Partcipant No (67) E, PO

UNIVERSITI
MALAYA

Depart of Media and Communication Studies

AI and Mediated Intercultural Communication Questionnaire

Dear UM Student/ Researcher,

Through this survey, we want to assess the probable effects of Artificial Intelligence (AI) on mediated communication among people from different cultures. This instrument which been developed by the help of the existing literature and ChatGPT, has 15 structured items with five (5) options per item and six (6) open-ended questions. Your participation in the survey is voluntary and the personal identifications of the participants will be treated as strictly confidential.

Thank you for your cooperation.

The researchers

A. Demographic Information

1. Gender: ☐ Male ☑ Female

2. Age: 24

3. Nationality: Malaysian

4. Level of education: post-Grade MA

5. AI/ ChatGPT experience: ☐ Yes ☑ No

B. Intercultural Sensitivity Scale

Directions: The current AI-ICC questionnaire includes 15 items/ statements with five (5) options per item, as: 1 – Strongly agree; 2 – Agree, 3 – Uncertain; 2 – Disagree, and 5 – Strongly disagree.	Strongly agree	Agree	Uncertain	Disagree	Strongly disagree
1. AI and AI-powered machines can be helpful in bridging communication gaps between people from different cultures.	1	(2)	3	4	5
2. So far, AI has been successful in facilitating cross-cultural understanding and collaboration in mediated communication.	1	2	(3)	4	5
3. AI can help overcome language barriers and promote more inclusive communication among diverse groups.	1	(2)	3	4	5

CamScanner

No 67 E P2

4. There some cultural biases and stereotypes in AI-powered language tools and communication platforms.	1	**2**	3	4	5
5. AI has affected the localization and adaptation of content for different cultural audiences in digital communication.	1	**2**	3	4	5
6. I encountered any challenges in using AI-powered translation tools when communicating with people from different cultural backgrounds.	1	2	**3**	4	5
7. AI has the potential to promote cultural exchange and appreciation by enabling easier access to diverse perspectives and information.	1	**2**	3	4	5
8. AI-powered machines and chatbots should be programmed to respect and adapt to cultural norms and communication styles while interacting with users from different cultures.	1	**2**	3	4	5
9. There are some potential risks and concerns regarding AI's role in cross-cultural communication, such as perpetuating cultural stereotypes and misinterpretations.	1	2	**3**	4	5
10. I have participated in some cross-cultural virtual collaborations that relied on AI for communication and coordination.	1	2	3	4	**5**
11. I think AI can enhance intercultural learning experiences and foster empathy among individuals from different cultural backgrounds.	1	**2**	3	4	5
12. AI-powered virtual reality (VR) and augmented reality (AR) technologies can impact cross-cultural communication and understanding.	1	**2**	3	4	5
13. Somehow AI-generated content may unintentionally offend and misled individuals from specific cultural backgrounds.	1	**2**	3	4	5
14. Ethical considerations should be taken into account when designing AI-powered communication tools that cater to diverse cultural contexts.	**1**	2	3	4	5
15. Universities and educational institutions leverage AI to create more inclusive and culturally diverse online learning environments.	1	2	**3**	4	5

In the case of your agreement on sharing your answer/ points of views for questions below, please write down your comment/ point of view for all questions or any question that you prefer:

1. Did you experience the use of AI and AI-powered chatbots, such as ChatGPT and for what purpose?

   I had never experience using any.

2. How do you perceive the role of AI and AI-powered machines in bridging communication gaps between people from different cultures?

   I think it is a good effort but I think it is not well accepted in certain cultures and beliefs.

CamScanner

No (67) & P(3)

3. Have you experienced and observed any instances where AI has been successful in facilitating cross-cultural understanding and collaboration in mediated communication?

No.

4. In what ways do you think AI can help overcome language barriers and promote more inclusive communication among diverse groups?

Sure way like how internet overcome it

5. Are there any cultural biases or stereotypes that you have noticed in AI-powered language tools or communication platforms?

No because no experience.

6. How can universities and educational institutions leverage AI to create more inclusive and culturally diverse online learning environments?

By providing multi-language AI.

Thank you for your time and participation

CS CamScanner

Participant No (68) E P10

UNIVERSITI MALAYA

Depart of Media and Communication Studies

AI and Mediated Intercultural Communication Questionnaire

Dear UM Student/ Researcher,

Through this survey, we want to assess the probable effects of Artificial Intelligence (AI) on mediated communication among people from different cultures. This instrument which been developed by the help of the existing literature and ChatGPT, has 15 structured items with five (5) options per item and six (6) open-ended questions. Your participation in the survey is voluntary and the personal identifications of the participants will be treated as strictly confidential.

Thank you for your cooperation.

The researchers

A. Demographic Information

1. Gender: ☐ Male ☑ Female

2. Age: 29

3. Nationality: chinese

4. Level of education: master

5. AI/ ChatGPT experience: ☑ Yes ☐ No

B. Intercultural Sensitivity Scale

**Directions:** The current AI-ICC questionnaire includes 15 items/ statements with five (5) options per item, as: 1 – Strongly agree; 2 – Agree, 3 – Uncertain; 4 – Disagree, and 5 – Strongly disagree.	Strongly agree	Agree	Uncertain	Disagree	Strongly disagree
1. AI and AI-powered machines can be helpful in bridging communication gaps between people from different cultures.	1	2 ✓	3	4	5
2. So far, AI has been successful in facilitating cross-cultural understanding and collaboration in mediated communication.	1	2 ✓	3	4	5
3. AI can help overcome language barriers and promote more inclusive communication among diverse groups.	1	2 ✓	3	4	5

CamScanner

No (68) E PC

4. There some cultural biases and stereotypes in AI-powered language tools and communication platforms.	1	2	3 ✓	4	5
5. AI has affected the localization and adaptation of content for different cultural audiences in digital communication.	1	2 ✓	3	4	5
6. I encountered any challenges in using AI-powered translation tools when communicating with people from different cultural backgrounds.	1	2	3	4 ✓	5
7. AI has the potential to promote cultural exchange and appreciation by enabling easier access to diverse perspectives and information.	1	2 ✓	3	4	5
8. AI-powered machines and chatbots should be programmed to respect and adapt to cultural norms and communication styles while interacting with users from different cultures.	1	2 ✓	3	4	5
9. There are some potential risks and concerns regarding AI's role in cross-cultural communication, such as perpetuating cultural stereotypes and misinterpretations.	1	2	3 ✓	4	5
10. I have participated in some cross-cultural virtual collaborations that relied on AI for communication and coordination.	1	2 ✓	3	4	5
11. I think AI can enhance intercultural learning experiences and foster empathy among individuals from different cultural backgrounds.	1	2 ✓	3	4	5
12. AI-powered virtual reality (VR) and augmented reality (AR) technologies can impact cross-cultural communication and understanding.	1	2 ✓	3	4	5
13. Somehow AI-generated content may unintentionally offend and misled individuals from specific cultural backgrounds.	1	2 ✓	3	4	5
14. Ethical considerations should be taken into account when designing AI-powered communication tools that cater to diverse cultural contexts.	1	2 ✓	3	4	5
15. Universities and educational institutions leverage AI to create more inclusive and culturally diverse online learning environments.	1	2 ✓	3	4	5

In the case of your agreement on sharing your answer/ points of views for questions below, please write down your comment/ point of view for all questions or any question that you prefer:

1. Did you experience the use of AI and AI-powered chatbots, such as ChatGPT and for what purpose?

   Yes. to learn more about English words and to help understand materials

2. How do you perceive the role of AI and AI-powered machines in bridging communication gaps between people from different cultures?

   It's useful to some extent.

CS CamScanner

No (68) E1 P(3)

3. Have you experienced and observed any instances where AI has been successful in facilitating cross-cultural understanding and collaboration in mediated communication?

To ~~ask~~ questions about culture in Malaysia

4. In what ways do you think AI can help overcome language barriers and promote more inclusive communication among diverse groups?

when we learn a language encountering questions, asking AI tool maybe a effective way.

5. Are there any cultural biases or stereotypes that you have noticed in AI-powered language tools or communication platforms?

Yes.

6. How can universities and educational institutions leverage AI to create more inclusive and culturally diverse online learning environments?

I have no idea.

Thank you for your time and participation

CS CamScanner

Participants No (69) & P(1)

UNIVERSITI MALAYA

Depart of Media and Communication Studies

AI and Mediated Intercultural Communication Questionnaire

Dear UM Student/ Researcher,

Through this survey, we want to assess the probable effects of Artificial Intelligence (AI) on mediated communication among people from different cultures. This instrument which been developed by the help of the existing literature and ChatGPT, has 15 structured items with five (5) options per item and six (6) open-ended questions. Your participation in the survey is voluntary and the personal identifications of the participants will be treated as strictly confidential.

Thank you for your cooperation.

The researchers

A. Demographic Information

1. Gender: ☐ Male ☑ Female

2. Age: 22

3. Nationality: malaysian

4. Level of education: post- bachelor degree

5. AI/ ChatGPT experience: ☑ Yes ☐ No

B. Intercultural Sensitivity Scale

**Directions:** The current AI-ICC questionnaire includes 15 items/ statements with five (5) options per item, as: 1 – Strongly agree; 2 – Agree, 3 – Uncertain; 4 – Disagree, and 5 – Strongly disagree.	Strongly agree	Agree	Uncertain	Disagree	Strongly disagree
1. AI and AI-powered machines can be helpful in bridging communication gaps between people from different cultures.	1	(2)	3	4	5
2. So far, AI has been successful in facilitating cross-cultural understanding and collaboration in mediated communication.	1	(2)	3	4	5
3. AI can help overcome language barriers and promote more inclusive communication among diverse groups.	(1)	2	3	4	5

CamScanner

No (69) S, P(2)

4. There some cultural biases and stereotypes in AI-powered language tools and communication platforms.	1	2	(3)	4	5
5. AI has affected the localization and adaptation of content for different cultural audiences in digital communication.	1	(2)	3	4	5
6. I encountered any challenges in using AI-powered translation tools when communicating with people from different cultural backgrounds.	1	(2)	3	4	5
7. AI has the potential to promote cultural exchange and appreciation by enabling easier access to diverse perspectives and information.	(1)	2	3	4	5
8. AI-powered machines and chatbots should be programmed to respect and adapt to cultural norms and communication styles while interacting with users from different cultures.	(1)	2	3	4	5
9. There are some potential risks and concerns regarding AI's role in cross-cultural communication, such as perpetuating cultural stereotypes and misinterpretations.	(1)	2	3	4	5
10. I have participated in some cross-cultural virtual collaborations that relied on AI for communication and coordination.	1	2	3	(4)	5
11. I think AI can enhance intercultural learning experiences and foster empathy among individuals from different cultural backgrounds.	1	(2)	3	4	5
12. AI-powered virtual reality (VR) and augmented reality (AR) technologies can impact cross-cultural communication and understanding.	1	2	(3)	4	5
13. Somehow AI-generated content may unintentionally offend and misled individuals from specific cultural backgrounds.	(1)	2	3	4	5
14. Ethical considerations should be taken into account when designing AI-powered communication tools that cater to diverse cultural contexts.	(1)	2	3	4	5
15. Universities and educational institutions leverage AI to create more inclusive and culturally diverse online learning environments.	(1)	2	3	4	5

In the case of your agreement on sharing your answer/ points of views for questions below, please write down your comment/ point of view for all questions or any question that you prefer:

1. Did you experience the use of AI and AI-powered chatbots, such as ChatGPT and for what purpose?

   Yes, for assignment purpose and any kind of letter to create sentence

2. How do you perceive the role of AI and AI-powered machines in bridging communication gaps between people from different cultures?

CamScanner

No (69) & P(3)

3. Have you experienced and observed any instances where AI has been successful in facilitating cross-cultural understanding and collaboration in mediated communication?

4. In what ways do you think AI can help overcome language barriers and promote more inclusive communication among diverse groups?

understand better about the language, easy to access and have higher chances to get correct answer

5. Are there any cultural biases or stereotypes that you have noticed in AI-powered language tools or communication platforms?

NO

6. How can universities and educational institutions leverage AI to create more inclusive and culturally diverse online learning environments?

the acceptance
- increase the use of AI

Thank you for your time and participation

CS CamScanner

Participant No (70) E P(1)

UNIVERSITI MALAYA

Depart of Media and Communication Studies

AI and Mediated Intercultural Communication Questionnaire

Dear UM Student/ Researcher,

Through this survey, we want to assess the probable effects of Artificial Intelligence (AI) on mediated communication among people from different cultures. This instrument which been developed by the help of the existing literature and ChatGPT, has 15 structured items with five (5) options per item and six (6) open-ended questions. Your participation in the survey is voluntary and the personal identifications of the participants will be treated as strictly confidential.

Thank you for your cooperation.

The researchers

A. Demographic Information

1. Gender: ☑ Male ☐ Female

2. Age: 24

3. Nationality: China

4. Level of education: Master

5. AI/ ChatGPT experience: ☑ Yes ☐ No

B. Intercultural Sensitivity Scale

Directions: The current AI-ICC questionnaire includes 15 items/ statements with five (5) options per item, as: 1 – Strongly agree; 2 – Agree, 3 – Uncertain; 4 – Disagree, and 5 – Strongly disagree.	Strongly agree	Agree	Uncertain	Disagree	Strongly disagree
1. AI and AI-powered machines can be helpful in bridging communication gaps between people from different cultures.	1 ✓	2	3	4	5
2. So far, AI has been successful in facilitating cross-cultural understanding and collaboration in mediated communication.	1 ✓	2	3	4	5
3. AI can help overcome language barriers and promote more inclusive communication among diverse groups.	1	2 ✓	3	4	5

CamScanner

No (70) E, P2

4. There some cultural biases and stereotypes in AI-powered language tools and communication platforms.	1	2 ✓	3	4	5
5. AI has affected the localization and adaptation of content for different cultural audiences in digital communication.	1 ✓	2	3	4	5
6. I encountered any challenges in using AI-powered translation tools when communicating with people from different cultural backgrounds.	1	2	3	4 ✓	5
7. AI has the potential to promote cultural exchange and appreciation by enabling easier access to diverse perspectives and information.	1 ✓	2	3	4	5
8. AI-powered machines and chatbots should be programmed to respect and adapt to cultural norms and communication styles while interacting with users from different cultures.	1	2 ✓	3	4	5
9. There are some potential risks and concerns regarding AI's role in cross-cultural communication, such as perpetuating cultural stereotypes and misinterpretations.	1	2 ✓	3	4	5
10. I have participated in some cross-cultural virtual collaborations that relied on AI for communication and coordination.	1	2	3	4	5 ✓
11. I think AI can enhance intercultural learning experiences and foster empathy among individuals from different cultural backgrounds.	1	2 ✓	3	4	5
12. AI-powered virtual reality (VR) and augmented reality (AR) technologies can impact cross-cultural communication and understanding.	1 ✓	2	3	4	5
13. Somehow AI-generated content may unintentionally offend and misled individuals from specific cultural backgrounds.	1	2	3	4 ✓	5
14. Ethical considerations should be taken into account when designing AI-powered communication tools that cater to diverse cultural contexts.	1 ✓	2	3	4	5
15. Universities and educational institutions leverage AI to create more inclusive and culturally diverse online learning environments.	1	2 ✓	3	4	5

In the case of your agreement on sharing your answer/ points of views for questions below, please write down your comment/ point of view for all questions or any question that you prefer:

1. Did you experience the use of AI and AI-powered chatbots, such as ChatGPT and for what purpose?

   Access to learning materials

2. How do you perceive the role of AI and AI-powered machines in bridging communication gaps between people from different cultures?

   Not experience it yet.

CamScanner

No (70) & P(3)

3. Have you experienced and observed any instances where AI has been successful in facilitating cross-cultural understanding and collaboration in mediated communication?

When I want to search some information of Malaysia It really helpful.

4. In what ways do you think AI can help overcome language barriers and promote more inclusive communication among diverse groups?

Plug AI into our brain.

5. Are there any cultural biases or stereotypes that you have noticed in AI-powered language tools or communication platforms?

Not yet.

6. How can universities and educational institutions leverage AI to create more inclusive and culturally diverse online learning environments?

No idea. AI still need a big progress to realize it

Thank you for your time and participation

CamScanner

Participant No (11) Ec PO

UNIVERSITI MALAYA

Depart of Media and Communication Studies

AI and Mediated Intercultural Communication Questionnaire

Dear UM Student/ Researcher,

Through this survey, we want to assess the probable effects of Artificial Intelligence (AI) on mediated communication among people from different cultures. This instrument which been developed by the help of the existing literature and ChatGPT, has 15 structured items with five (5) options per item and six (6) open-ended questions. Your participation in the survey is voluntary and the personal identifications of the participants will be treated as strictly confidential.

Thank you for your cooperation.

The researchers

A. Demographic Information

1. Gender: ☑ Male ☐ Female

2. Age: 28

3. Nationality: Malaysia

4. Level of education: ~~Foundation~~ Post grade degree

5. AI/ ChatGPT experience: ☑ Yes ☑ No

B. Intercultural Sensitivity Scale

Directions: The current AI-ICC questionnaire includes 15 items/ statements with five (5) options per item, as: 1 – Strongly agree; 2 – Agree, 3 – Uncertain; 4 – Disagree, and 5 – Strongly disagree.	Strongly agree	Agree	Uncertain	Disagree	Strongly disagree
1. AI and AI-powered machines can be helpful in bridging communication gaps between people from different cultures.	1	(2)	3	4	5
2. So far, AI has been successful in facilitating cross-cultural understanding and collaboration in mediated communication.	1	2	(3)	4	5
3. AI can help overcome language barriers and promote more inclusive communication among diverse groups.	1	2	(3)	4	5

CamScanner

No (71) & P2

4. There some cultural biases and stereotypes in AI-powered language tools and communication platforms.	1	2	(3)	4	5
5. AI has affected the localization and adaptation of content for different cultural audiences in digital communication.	1	2	(3)	4	5
6. I encountered any challenges in using AI-powered translation tools when communicating with people from different cultural backgrounds.	1	2	3	(4)	5
7. AI has the potential to promote cultural exchange and appreciation by enabling easier access to diverse perspectives and information.	1	(2)	3	4	5
8. AI-powered machines and chatbots should be programmed to respect and adapt to cultural norms and communication styles while interacting with users from different cultures.	1	2	(3)	4	5
9. There are some potential risks and concerns regarding AI's role in cross-cultural communication, such as perpetuating cultural stereotypes and misinterpretations.	1	(2)	3	4	5
10. I have participated in some cross-cultural virtual collaborations that relied on AI for communication and coordination.	1	2	3	(4)	5
11. I think AI can enhance intercultural learning experiences and foster empathy among individuals from different cultural backgrounds.	1	(2)	3	4	5
12. AI-powered virtual reality (VR) and augmented reality (AR) technologies can impact cross-cultural communication and understanding.	1	(2)	3	4	5
13. Somehow AI-generated content may unintentionally offend and misled individuals from specific cultural backgrounds.	1	2	3	(4)	5
14. Ethical considerations should be taken into account when designing AI-powered communication tools that cater to diverse cultural contexts.	(1)	2	3	4	5
15. Universities and educational institutions leverage AI to create more inclusive and culturally diverse online learning environments.	1	(2)	3	4	5

In the case of your agreement on sharing your answer/ points of views for questions below, please write down your comment/ point of view for all questions or any question that you prefer:

1. Did you experience the use of AI and AI-powered chatbots, such as ChatGPT and for what purpose?

   I haven't experience with it at all.

2. How do you perceive the role of AI and AI-powered machines in bridging communication gaps between people from different cultures?

   So far, I don't see it ~~useful as~~ play a big role in term of ~~exploring culture~~ bridging communication from different cultures, because, I don't think AI, can't be ~~reas~~ such realistic ~~rather than~~ compared to other trusted sources, e.g, people, books, socmed influencer

CamScanner

No (71) & P(3)

3. Have you experienced and observed any instances where AI has been successful in facilitating cross-cultural understanding and collaboration in mediated communication?

No

4. In what ways do you think AI can help overcome language barriers and promote more inclusive communication among diverse groups?

Give exposure to more details, ~~under~~ native, realistic, simple, understandable,

5. Are there any cultural biases or stereotypes that you have noticed in AI-powered language tools or communication platforms?

No

6. How can universities and educational institutions leverage AI to create more inclusive and culturally diverse online learning environments?

~~Improving own our own AI powered~~

Accessible & comprehensive to variety of language speaker, level of education and age.

Thank you for your time and participation

CamScanner

Participants No (12) & P(1)

UNIVERSITI MALAYA

Depart of Media and Communication Studies

AI and Mediated Intercultural Communication Questionnaire

Dear UM Student/ Researcher,

Through this survey, we want to assess the probable effects of Artificial Intelligence (AI) on mediated communication among people from different cultures. This instrument which been developed by the help of the existing literature and ChatGPT, has 15 structured items with five (5) options per item and six (6) open-ended questions. Your participation in the survey is voluntary and the personal identifications of the participants will be treated as strictly confidential.

Thank you for your cooperation.

The researchers

A. Demographic Information

1. Gender: ☑ Male ☐ Female

2. Age: 25

3. Nationality: British (UK)

4. Level of education: MA

5. AI/ ChatGPT experience: ☑ Yes ☐ No

B. Intercultural Sensitivity Scale

Directions: The current AI-ICC questionnaire includes 15 items/ statements with five (5) options per item, as: 1 – Strongly agree; 2 – Agree, 3 – Uncertain; 4 – Disagree, and 5 – Strongly disagree.	Strongly agree	Agree	Uncertain	Disagree	Strongly disagree
1. AI and AI-powered machines can be helpful in bridging communication gaps between people from different cultures.	1	2	(3)	4	5
2. So far, AI has been successful in facilitating cross-cultural understanding and collaboration in mediated communication.	1	2	(3)	4	5
3. AI can help overcome language barriers and promote more inclusive communication among diverse groups.	1	(2)	3	4	5

CamScanner

Participant No (12) [illegible]

4. There [are] some cultural biases and stereotypes in AI-powered language tools and communication platforms.	(1)	2	3	4	5
5. AI has affected the localization and adaptation of content for different cultural audiences in digital communication.	1	(2)	3	4	5
6. I encountered any challenges in using AI-powered translation tools when communicating with people from different cultural backgrounds. NA	1	2	3	4	5
7. AI has the potential to promote cultural exchange and appreciation by enabling easier access to diverse perspectives and information.	1	(2)	3	4	5
8. AI-powered machines and chatbots should be programmed to respect and adapt to cultural norms and communication styles while interacting with users from different cultures.	(1)	2	3	4	5
9. There are some potential risks and concerns regarding AI's role in cross-cultural communication, such as perpetuating cultural stereotypes and misinterpretations.	(1)	2	3	4	5
10. I have participated in some cross-cultural virtual collaborations that relied on AI for communication and coordination.	1	2	3	4	(5)
11. I think AI can enhance intercultural learning experiences and foster empathy among individuals from different cultural backgrounds.	1	2	(3)	4	5
12. AI-powered virtual reality (VR) and augmented reality (AR) technologies can impact cross-cultural communication and understanding.	1	2	3	4	(5)
13. Somehow AI-generated content may unintentionally offend and misled individuals from specific cultural backgrounds.	1	(2)	3	4	5
14. Ethical considerations should be taken into account when designing AI-powered communication tools that cater to diverse cultural contexts.	(1)	2	3	4	5
15. Universities and educational institutions leverage AI to create more inclusive and culturally diverse online learning environments.	1	2	(3)	4	5

In the case of your agreement on sharing your answer/ points of views for questions below, please write down your comment/ point of view for all questions or any question that you prefer:

1. Did you experience the use of AI and AI-powered chatbots, such as ChatGPT and for what purpose?

   Translation, editing, conversation and to test its potential

2. How do you perceive the role of AI and AI-powered machines in bridging communication gaps between people from different cultures?

   As a language model it relies primarily on a corpus of English language written texts produced in the West. Thus, ~~the~~ it's outputs reflect that corpus and its biases. Other languages, and other forms of

CamScanner

Participant no (12) & [illegible]

3. Have you experienced and observed any instances where AI has been successful in facilitating cross-cultural understanding and collaboration in mediated communication?

I have not tried

4. In what ways do you think AI can help overcome language barriers and promote more inclusive communication among diverse groups?

As a translation tool primarily

5. Are there any cultural biases or stereotypes that you have noticed in AI-powered language tools or communication platforms?

I think it is more implicit than explicit

6. How can universities and educational institutions leverage AI to create more inclusive and culturally diverse online learning environments?

I'm not sure how helpful they can be. Other methods may be more productive.

Thank you for your time and participation

Perhaps as a language learning aid (not an end-all be-all solution)

CamScanner

Participant No (73) & PO(1)

UNIVERSITI MALAYA

Depart of Media and Communication Studies

AI and Mediated Intercultural Communication Questionnaire

Dear UM Student/ Researcher,

Through this survey, we want to assess the probable effects of Artificial Intelligence (AI) on mediated communication among people from different cultures. This instrument which been developed by the help of the existing literature and ChatGPT, has 15 structured items with five (5) options per item and six (6) open-ended questions. Your participation in the survey is voluntary and the personal identifications of the participants will be treated as strictly confidential.

Thank you for your cooperation.

The researchers

A. Demographic Information

1. Gender: ☑ Male ☐ Female
2. Age: 34
3. Nationality: MALAYSIA
4. Level of education: PHD
5. AI/ ChatGPT experience: ☑ Yes ☐ No

B. Intercultural Sensitivity Scale

**Directions:** The current AI-ICC questionnaire includes 15 items/ statements with five (5) options per item, as: 1 – Strongly agree; 2 – Agree, 3 – Uncertain; 2 – Disagree, and 5 – Strongly disagree.	Strongly agree	Agree	Uncertain	Disagree	Strongly disagree
1. AI and AI-powered machines can be helpful in bridging communication gaps between people from different cultures.	(1)	2	3	4	5
2. So far, AI has been successful in facilitating cross-cultural understanding and collaboration in mediated communication.	1	(2)	3	4	5
3. AI can help overcome language barriers and promote more inclusive communication among diverse groups.	1	(2)	3	4	5

CamScanner

No (73) S1 P2

4. There some cultural biases and stereotypes in AI-powered language tools and communication platforms.	1	2	3	(4)	5
5. AI has affected the localization and adaptation of content for different cultural audiences in digital communication.	1	2	(3)	4	5
6. I encountered any challenges in using AI-powered translation tools when communicating with people from different cultural backgrounds.	1	(2)	3	4	5
7. AI has the potential to promote cultural exchange and appreciation by enabling easier access to diverse perspectives and information.	1	(2)	3	4	5
8. AI-powered machines and chatbots should be programmed to respect and adapt to cultural norms and communication styles while interacting with users from different cultures.	1	(2)	3	4	5
9. There are some potential risks and concerns regarding AI's role in cross-cultural communication, such as perpetuating cultural stereotypes and misinterpretations.	1	2	(3)	4	5
10. I have participated in some cross-cultural virtual collaborations that relied on AI for communication and coordination.	1	(2)	3	4	5
11. I think AI can enhance intercultural learning experiences and foster empathy among individuals from different cultural backgrounds.	1	(2)	3	4	5
12. AI-powered virtual reality (VR) and augmented reality (AR) technologies can impact cross-cultural communication and understanding.	1	2	(3)	4	5
13. Somehow AI-generated content may unintentionally offend and misled individuals from specific cultural backgrounds.	1	2	(3)	4	5
14. Ethical considerations should be taken into account when designing AI-powered communication tools that cater to diverse cultural contexts.	(1)	2	3	4	5
15. Universities and educational institutions leverage AI to create more inclusive and culturally diverse online learning environments.	(1)	2	3	4	5

In the case of your agreement on sharing your answer/ points of views for questions below, please write down your comment/ point of view for all questions or any question that you prefer:

1. Did you experience the use of AI and AI-powered chatbots, such as ChatGPT and for what purpose?

   I use ~~expert~~ for searching information and checking my grammar

2. How do you perceive the role of AI and AI-powered machines in bridging communication gaps between people from different cultures?

   AI helping us to by providing information

CS CamScanner

No (73) & P(3)

3. Have you experienced and observed any instances where AI has been successful in facilitating cross-cultural understanding and collaboration in mediated communication?

Not sure

4. In what ways do you think AI can help overcome language barriers and promote more inclusive communication among diverse groups?

By providing information to us about diverse groups

5. Are there any cultural biases or stereotypes that you have noticed in AI-powered language tools or communication platforms?

Not really

6. How can universities and educational institutions leverage AI to create more inclusive and culturally diverse online learning environments?

Thank you for your time and participation

CS CamScanner

Partcipant No (74) E, P(1)

UNIVERSITI MALAYA

Depart of Media and Communication Studies

AI and Mediated Intercultural Communication Questionnaire

Dear UM Student/ Researcher,

Through this survey, we want to assess the probable effects of Artificial Intelligence (AI) on mediated communication among people from different cultures. This instrument which been developed by the help of the existing literature and ChatGPT, has 15 structured items with five (5) options per item and six (6) open-ended questions. Your participation in the survey is voluntary and the personal identifications of the participants will be treated as strictly confidential.

Thank you for your cooperation.

The researchers

A. Demographic Information

1. Gender: ✓ Male ☐ Female

2. Age: 42

3. Nationality: Malaysian

4. Level of education: PhD

5. AI/ ChatGPT experience: ✓ Yes ☐ No

B. Intercultural Sensitivity Scale

**Directions:** The current AI-ICC questionnaire includes 15 items/ statements with five (5) options per item, as: 1 – Strongly agree; 2 – Agree, 3 – Uncertain; 2 – Disagree, and 5 – Strongly disagree.	Strongly agree	Agree	Uncertain	Disagree	Strongly disagree
1. AI and AI-powered machines can be helpful in bridging communication gaps between people from different cultures.	1	(2)	3	4	5
2. So far, AI has been successful in facilitating cross-cultural understanding and collaboration in mediated communication.	1	2	(3)	4	5
3. AI can help overcome language barriers and promote more inclusive communication among diverse groups.	1	(2)	3	4	5

CamScanner

No (74) E, P2

4. There some cultural biases and stereotypes in AI-powered language tools and communication platforms.	1	(2)	3	4	5
5. AI has affected the localization and adaptation of content for different cultural audiences in digital communication.	1	(2)	3	4	5
6. I encountered any challenges in using AI-powered translation tools when communicating with people from different cultural backgrounds.	1	(2)	3	4	5
7. AI has the potential to promote cultural exchange and appreciation by enabling easier access to diverse perspectives and information.	1	(2)	3	4	5
8. AI-powered machines and chatbots should be programmed to respect and adapt to cultural norms and communication styles while interacting with users from different cultures.	1	(2)	3	4	5
9. There are some potential risks and concerns regarding AI's role in cross-cultural communication, such as perpetuating cultural stereotypes and misinterpretations.	1	(2)	3	4	5
10. I have participated in some cross-cultural virtual collaborations that relied on AI for communication and coordination.	1	2	3	(4)	5
11. I think AI can enhance intercultural learning experiences and foster empathy among individuals from different cultural backgrounds.	1	(2)	3	4	5
12. AI-powered virtual reality (VR) and augmented reality (AR) technologies can impact cross-cultural communication and understanding.	1	(2)	3	4	5
13. Somehow AI-generated content may unintentionally offend and misled individuals from specific cultural backgrounds.	1	(2)	3	4	5
14. Ethical considerations should be taken into account when designing AI-powered communication tools that cater to diverse cultural contexts.	1	(2)	3	4	5
15. Universities and educational institutions leverage AI to create more inclusive and culturally diverse online learning environments.	1	(2)	3	4	5

In the case of your agreement on sharing your answer/ points of views for questions below, please write down your comment/ point of view for all questions or any question that you prefer:

1. Did you experience the use of AI and AI-powered chatbots, such as ChatGPT and for what purpose?

   ~~Educa~~ education

2. How do you perceive the role of AI and AI-powered machines in bridging communication gaps between people from different cultures?

   Translation help to minimize communication gaps & language barriers.

CS CamScanner

No (74) E1 P3

3. Have you experienced and observed any instances where AI has been successful in facilitating cross-cultural understanding and collaboration in mediated communication?

During class presentation, International students used translation apps to understand question during QnA.

4. In what ways do you think AI can help overcome language barriers and promote more inclusive communication among diverse groups?

Translation will give massive contribution.

5. Are there any cultural biases or stereotypes that you have noticed in AI-powered language tools or communication platforms?

No.

6. How can universities and educational institutions leverage AI to create more inclusive and culturally diverse online learning environments?

To be included in teaching aids.

Thank you for your time and participation

CS CamScanner

Participant No (75) E P(1)

UNIVERSITI MALAYA

Depart of Media and Communication Studies

AI and Mediated Intercultural Communication Questionnaire

Dear UM Student/ Researcher,

Through this survey, we want to assess the probable effects of Artificial Intelligence (AI) on mediated communication among people from different cultures. This instrument which been developed by the help of the existing literature and ChatGPT, has 15 structured items with five (5) options per item and six (6) open-ended questions. Your participation in the survey is voluntary and the personal identifications of the participants will be treated as strictly confidential.

Thank you for your cooperation.

The researchers

A. Demographic Information

1. Gender: ☐ Male ☑ Female

2. Age: 27

3. Nationality: malaysian

4. Level of education: ~~post~~ ~~under~~ postgraduate

5. AI/ ChatGPT experience: ☑ Yes ☐ No

B. Intercultural Sensitivity Scale

Directions: The current AI-ICC questionnaire includes 15 items/ statements with five (5) options per item, as: 1 – Strongly agree; 2 – Agree, 3 – Uncertain; 2 – Disagree, and 5 – Strongly disagree.	Strongly agree	Agree	Uncertain	Disagree	Strongly disagree
1. AI and AI-powered machines can be helpful in bridging communication gaps between people from different cultures.	1	(2)	3	4	5
2. So far, AI has been successful in facilitating cross-cultural understanding and collaboration in mediated communication.	1	(2)	3	4	5
3. AI can help overcome language barriers and promote more inclusive communication among diverse groups.	(1)	2	3	4	5

CamScanner

No (75) [illegible]

4. There some cultural biases and stereotypes in AI-powered language tools and communication platforms.	1	(2)	3	4	5
5. AI has affected the localization and adaptation of content for different cultural audiences in digital communication.	1	(2)	3	4	5
6. I encountered any challenges in using AI-powered translation tools when communicating with people from different cultural backgrounds.	1	2	3	(4)	5
7. AI has the potential to promote cultural exchange and appreciation by enabling easier access to diverse perspectives and information.	1	(2)	3	4	5
8. AI-powered machines and chatbots should be programmed to respect and adapt to cultural norms and communication styles while interacting with users from different cultures.	(1)	2	3	4	5
9. There are some potential risks and concerns regarding AI's role in cross-cultural communication, such as perpetuating cultural stereotypes and misinterpretations.	(1)	2	3	4	5
10. I have participated in some cross-cultural virtual collaborations that relied on AI for communication and coordination.	1	2	3	4	(5)
11. I think AI can enhance intercultural learning experiences and foster empathy among individuals from different cultural backgrounds.	1	2	(3)	4	5
12. AI-powered virtual reality (VR) and augmented reality (AR) technologies can impact cross-cultural communication and understanding.	1	2	(3)	4	5
13. Somehow AI-generated content may unintentionally offend and misled individuals from specific cultural backgrounds.	1	(2)	3	4	5
14. Ethical considerations should be taken into account when designing AI-powered communication tools that cater to diverse cultural contexts.	(1)	2	3	4	5
15. Universities and educational institutions leverage AI to create more inclusive and culturally diverse online learning environments.	1	2	(3)	4	5

In the case of your agreement on sharing your answer/ points of views for questions below, please write down your comment/ point of view for all questions or any question that you prefer:

1. Did you experience the use of AI and AI-powered chatbots, such as ChatGPT and for what purpose?

   Yes. To help understand learning outcomes, assist in assignments, and to paraphrase words.

2. How do you perceive the role of AI and AI-powered machines in bridging communication gaps between people from different cultures?

   It's good, we should make use of it.

CS CamScanner

No (75) E P(3)

3. Have you experienced and observed any instances where AI has been successful in facilitating cross-cultural understanding and collaboration in mediated communication?

I have not

4. In what ways do you think AI can help overcome language barriers and promote more inclusive communication among diverse groups?

As translation

5. Are there any cultural biases or stereotypes that you have noticed in AI-powered language tools or communication platforms?

Nope

6. How can universities and educational institutions leverage AI to create more inclusive and culturally diverse online learning environments?

Not sure.

Thank you for your time and participation

CS CamScanner

Partigpant No (76) E PO

UNIVERSITI MALAYA

Depart of Media and Communication Studies

AI and Mediated Intercultural Communication Questionnaire

Dear UM Student/ Researcher,

Through this survey, we want to assess the probable effects of Artificial Intelligence (AI) on mediated communication among people from different cultures. This instrument which been developed by the help of the existing literature and ChatGPT, has 15 structured items with five (5) options per item and six (6) open-ended questions. Your participation in the survey is voluntary and the personal identifications of the participants will be treated as strictly confidential.

Thank you for your cooperation.

The researchers

A. Demographic Information

1. Gender: ☐ Male ✓☐ Female

2. Age: 26

3. Nationality: Pakistani

4. Level of education: Master

5. AI/ ChatGPT experience: ✓☐ Yes ☐ No

B. Intercultural Sensitivity Scale

Directions: The current AI-ICC questionnaire includes 15 items/ statements with five (5) options per item, as: 1 – Strongly agree; 2 – Agree, 3 – Uncertain; 4 – Disagree, and 5 – Strongly disagree.	Strongly agree	Agree	Uncertain	Disagree	Strongly disagree
1. AI and AI-powered machines can be helpful in bridging communication gaps between people from different cultures.	1	2	3	4	5 ✓
2. So far, AI has been successful in facilitating cross-cultural understanding and collaboration in mediated communication.	1	2	3	4 ✓	5
3. AI can help overcome language barriers and promote more inclusive communication among diverse groups.	1	2	3 ✓	4	5

CS CamScanner

NO (76) & Po

4. There some cultural biases and stereotypes in AI-powered language tools and communication platforms.	1	2	3	4	5
5. AI has affected the localization and adaptation of content for different cultural audiences in digital communication.	1	2	3	4	5
6. I encountered any challenges in using AI-powered translation tools when communicating with people from different cultural backgrounds.	1	2	3	4	5
7. AI has the potential to promote cultural exchange and appreciation by enabling easier access to diverse perspectives and information.	1	2	3	4	5
8. AI-powered machines and chatbots should be programmed to respect and adapt to cultural norms and communication styles while interacting with users from different cultures.	1	2	3	4	5
9. There are some potential risks and concerns regarding AI's role in cross-cultural communication, such as perpetuating cultural stereotypes and misinterpretations.	1	2	3	4	5
10. I have participated in some cross-cultural virtual collaborations that relied on AI for communication and coordination.	1	2	3	4	5
11. I think AI can enhance intercultural learning experiences and foster empathy among individuals from different cultural backgrounds.	1	2	3	4	5
12. AI-powered virtual reality (VR) and augmented reality (AR) technologies can impact cross-cultural communication and understanding.	1	2	3	4	5
13. Somehow AI-generated content may unintentionally offend and misled individuals from specific cultural backgrounds.	1	2	3	4	5
14. Ethical considerations should be taken into account when designing AI-powered communication tools that cater to diverse cultural contexts.	1	2	3	4	5
15. Universities and educational institutions leverage AI to create more inclusive and culturally diverse online learning environments.	1	2	3	4	5

In the case of your agreement on sharing your answer/ points of views for questions below, please write down your comment/ point of view for all questions or any question that you prefer:

1. Did you experience the use of AI and AI-powered chatbots, such as ChatGPT and for what purpose?

Rarely, for rephrasing.

2. How do you perceive the role of AI and AI-powered machines in bridging communication gaps between people from different cultures?

I do not see it playing any role.

CS CamScanner

No (76) E P3

3. Have you experienced and observed any instances where AI has been successful in facilitating cross-cultural understanding and collaboration in mediated communication?

NO

4. In what ways do you think AI can help overcome language barriers and promote more inclusive communication among diverse groups?

I do not see AI in a positive light.

5. Are there any cultural biases or stereotypes that you have noticed in AI-powered language tools or communication platforms?

NO

6. How can universities and educational institutions leverage AI to create more inclusive and culturally diverse online learning environments?

universities should not use it.

Thank you for your time and participation

CS CamScanner

Participant No (77) Ec PO

UNIVERSITI MALAYA

Depart of Media and Communication Studies

AI and Mediated Intercultural Communication Questionnaire

Dear UM Student/ Researcher,

Through this survey, we want to assess the probable effects of Artificial Intelligence (AI) on mediated communication among people from different cultures. This instrument which been developed by the help of the existing literature and ChatGPT, has 15 structured items with five (5) options per item and six (6) open-ended questions. Your participation in the survey is voluntary and the personal identifications of the participants will be treated as strictly confidential.

Thank you for your cooperation.

The researchers

A. Demographic Information

1. Gender: ☐ Male ☑ Female

2. Age: 27

3. Nationality: Malaysian

4. Level of education: Post Graduate Degree

5. AI/ ChatGPT experience: ☑ Yes ☐ No

B. Intercultural Sensitivity Scale

Directions: The current AI-ICC questionnaire includes 15 items/ statements with five (5) options per item, as: 1 – Strongly agree; 2 – Agree, 3 – Uncertain; 4 – Disagree, and 5 – Strongly disagree.	Strongly agree	Agree	Uncertain	Disagree	Strongly disagree
1. AI and AI-powered machines can be helpful in bridging communication gaps between people from different cultures.	1	2 ✗	3	4	5
2. So far, AI has been successful in facilitating cross-cultural understanding and collaboration in mediated communication.	1	2 ✗	3	4	5
3. AI can help overcome language barriers and promote more inclusive communication among diverse groups.	1	2 ✗	3	4	5

CamScanner

No (77) G P(2)

4. There some cultural biases and stereotypes in AI-powered language tools and communication platforms.	1	2	✓3	4	5
5. AI has affected the localization and adaptation of content for different cultural audiences in digital communication.	1	✓2	3	4	5
6. I encountered any challenges in using AI-powered translation tools when communicating with people from different cultural backgrounds.	1	2	✓3	4	5
7. AI has the potential to promote cultural exchange and appreciation by enabling easier access to diverse perspectives and information.	1	2	✓3	4	5
8. AI-powered machines and chatbots should be programmed to respect and adapt to cultural norms and communication styles while interacting with users from different cultures.	1	2	✓3	4	5
9. There are some potential risks and concerns regarding AI's role in cross-cultural communication, such as perpetuating cultural stereotypes and misinterpretations.	1	✓2	3	4	5
10. I have participated in some cross-cultural virtual collaborations that relied on AI for communication and coordination.	1	✓2	3	4	5
11. I think AI can enhance intercultural learning experiences and foster empathy among individuals from different cultural backgrounds.	1	✓2	3	4	5
12. AI-powered virtual reality (VR) and augmented reality (AR) technologies can impact cross-cultural communication and understanding.	1	✓2	3	4	5
13. Somehow AI-generated content may unintentionally offend and misled individuals from specific cultural backgrounds.	1	✓2	3	4	5
14. Ethical considerations should be taken into account when designing AI-powered communication tools that cater to diverse cultural contexts.	✓1	2	3	4	5
15. Universities and educational institutions leverage AI to create more inclusive and culturally diverse online learning environments.	✓1	2	3	4	5

In the case of your agreement on sharing your answer/ points of views for questions below, please write down your comment/ point of view for all questions or any question that you prefer:

1. Did you experience the use of AI and AI-powered chatbots, such as ChatGPT and for what purpose?

   Yes

   ~~for~~ when not understand something

2. How do you perceive the role of AI and AI-powered machines in bridging communication gaps between people from different cultures?

   help people to understand other cultures, hence better communicate coz reduce chances of offending someone ~~to~~ from different culture background.

CS CamScanner

No (17) & P(3)

3. Have you experienced and observed any instances where AI has been successful in facilitating cross-cultural understanding and collaboration in mediated communication?

sorry no ~~[illegible]~~

4. In what ways do you think AI can help overcome language barriers and promote more inclusive communication among diverse groups?

when someone not understand can ask AI to explain or translate.

5. Are there any cultural biases or stereotypes that you have noticed in AI-powered language tools or communication platforms?

No. but I think more or less will have this kind of situation.

6. How can universities and educational institutions leverage AI to create more inclusive and culturally diverse online learning environments?

allow student to use it instead of outright banning this tool. can implement tools like Turnitin to avoid plagiarism or ~~to~~ copy directly from AI ~~[illegible]~~. Hence, ~~[illegible]~~ at the same time, student can learn ~~[illegible]~~ efficiently ~~but~~ and teacher can also avoid them from cheating

Thank you for your time and participation

CamScanner

Partcipant No (78) E P(1)

UNIVERSITI MALAYA

Depart of Media and Communication Studies

AI and Mediated Intercultural Communication Questionnaire

Dear UM Student/ Researcher,

Through this survey, we want to assess the probable effects of Artificial Intelligence (AI) on mediated communication among people from different cultures. This instrument which been developed by the help of the existing literature and ChatGPT, has 15 structured items with five (5) options per item and six (6) open-ended questions. Your participation in the survey is voluntary and the personal identifications of the participants will be treated as strictly confidential.

Thank you for your cooperation.

The researchers

A. Demographic Information

1. Gender: ☐ Male ☑ Female

2. Age: 25

3. Nationality: China

4. Level of education: Master

5. AI/ ChatGPT experience: ☑ Yes ☐ No

B. Intercultural Sensitivity Scale

Directions: The current AI-ICC questionnaire includes 15 items/ statements with five (5) options per item, as: 1 – Strongly agree; 2 – Agree, 3 – Uncertain; 4 – Disagree, and 5 – Strongly disagree.	Strongly agree	Agree	Uncertain	Disagree	Strongly disagree
1. AI and AI-powered machines can be helpful in bridging communication gaps between people from different cultures.	1	(2)	3	4	5
2. So far, AI has been successful in facilitating cross-cultural understanding and collaboration in mediated communication.	1	(2)	3	4	5
3. AI can help overcome language barriers and promote more inclusive communication among diverse groups.	1	(2)	3	4	5

No 78 Er P2

4. There some cultural biases and stereotypes in AI-powered language tools and communication platforms.	1	2	3	4	5
5. AI has affected the localization and adaptation of content for different cultural audiences in digital communication.	1	2	3	4	5
6. I encountered any challenges in using AI-powered translation tools when communicating with people from different cultural backgrounds.	1	2	3	4	5
7. AI has the potential to promote cultural exchange and appreciation by enabling easier access to diverse perspectives and information.	1	2	3	4	5
8. AI-powered machines and chatbots should be programmed to respect and adapt to cultural norms and communication styles while interacting with users from different cultures.	1	2	3	4	5
9. There are some potential risks and concerns regarding AI's role in cross-cultural communication, such as perpetuating cultural stereotypes and misinterpretations.	1	2	3	4	5
10. I have participated in some cross-cultural virtual collaborations that relied on AI for communication and coordination.	1	2	3	4	5
11. I think AI can enhance intercultural learning experiences and foster empathy among individuals from different cultural backgrounds.	1	2	3	4	5
12. AI-powered virtual reality (VR) and augmented reality (AR) technologies can impact cross-cultural communication and understanding.	1	2	3	4	5
13. Somehow AI-generated content may unintentionally offend and misled individuals from specific cultural backgrounds.	1	2	3	4	5
14. Ethical considerations should be taken into account when designing AI-powered communication tools that cater to diverse cultural contexts.	1	2	3	4	5
15. Universities and educational institutions leverage AI to create more inclusive and culturally diverse online learning environments.	1	2	3	4	5

In the case of your agreement on sharing your answer/ points of views for questions below, please write down your comment/ point of view for all questions or any question that you prefer:

1. Did you experience the use of AI and AI-powered chatbots, such as ChatGPT and for what purpose?

Yes. use it for assignments.

2. How do you perceive the role of AI and AI-powered machines in bridging communication gaps between people from different cultures?

Helpful ~~expects~~ especially when dealing with paper work. the translation have to be accurate to know other member's idea. This is a big part when

[illegible]

No (78) & P(3)

3. Have you experienced and observed any instances where AI has been successful in facilitating cross-cultural understanding and collaboration in mediated communication?

Yes. sometime I chat with my friend from America. He just using the word or sentence that I don't understand. And I'll ask cha[illegible] for help.

4. In what ways do you think AI can help overcome language barriers and promote more inclusive communication among diverse groups?

I think it's hopeful and I'd love to accept it.

5. Are there any cultural biases or stereotypes that you have noticed in AI-powered language tools or communication platforms?

Not yet.

6. How can universities and educational institutions leverage AI to create more inclusive and culturally diverse online learning environments?

Not sure. ~~[illegible]~~ ~~I prefer~~ Maybe instant ~~subtitle~~ translation and some culture background tips with it?

Thank you for your time and participation

CamScanner

Partcipant No (73) [illegible] P(1)

UNIVERSITI
MALAYA

Depart of Media and Communication Studies

AI and Mediated Intercultural Communication Questionnaire

Dear UM Student/ Researcher,

Through this survey, we want to assess the probable effects of Artificial Intelligence (AI) on mediated communication among people from different cultures. This instrument which been developed by the help of the existing literature and ChatGPT, has 15 structured items with five (5) options per item and six (6) open-ended questions. Your participation in the survey is voluntary and the personal identifications of the participants will be treated as strictly confidential.

Thank you for your cooperation.

The researchers

A. Demographic Information

1. Gender: ☐ Male ✓ Female

2. Age: 30

3. Nationality: China

4. Level of education: PHD

5. AI/ ChatGPT experience: ✓ Yes ☐ No

B. Intercultural Sensitivity Scale

**Directions:** The current AI-ICC questionnaire includes 15 items/ statements with five (5) options per item, as: 1 – Strongly agree; 2 – Agree, 3 – Uncertain; 4 – Disagree, and 5 – Strongly disagree.	Strongly agree	Agree	Uncertain	Disagree	Strongly disagree
1. AI and AI-powered machines can be helpful in bridging communication gaps between people from different cultures.	1	2 ✓	3	4	5
2. So far, AI has been successful in facilitating cross-cultural understanding and collaboration in mediated communication.	1	2	3 ✓	4	5
3. AI can help overcome language barriers and promote more inclusive communication among diverse groups.	1	2	3 ✓	4	5

CamScanner

No (79) Ec P2

4. There some cultural biases and stereotypes in AI-powered language tools and communication platforms.	1	2 ✓	3	4	5
5. AI has affected the localization and adaptation of content for different cultural audiences in digital communication.	1	2 ✓	3	4	5
6. I encountered any challenges in using AI-powered translation tools when communicating with people from different cultural backgrounds.	1	2 ✓	3	4	5
7. AI has the potential to promote cultural exchange and appreciation by enabling easier access to diverse perspectives and information.	1	2 ✓	3	4	5
8. AI-powered machines and chatbots should be programmed to respect and adapt to cultural norms and communication styles while interacting with users from different cultures.	1 ✓	2	3	4	5
9. There are some potential risks and concerns regarding AI's role in cross-cultural communication, such as perpetuating cultural stereotypes and misinterpretations.	1 ✓	2	3	4	5
10. I have participated in some cross-cultural virtual collaborations that relied on AI for communication and coordination.	1	2	3	4 ✓	5
11. I think AI can enhance intercultural learning experiences and foster empathy among individuals from different cultural backgrounds.	1	2	3 ✓	4	5
12. AI-powered virtual reality (VR) and augmented reality (AR) technologies can impact cross-cultural communication and understanding.	1	2	3 ✓	4	5
13. Somehow AI-generated content may unintentionally offend and misled individuals from specific cultural backgrounds.	1	2	3 ✓	4	5
14. Ethical considerations should be taken into account when designing AI-powered communication tools that cater to diverse cultural contexts.	1 ✓	2	3	4	5
15. Universities and educational institutions leverage AI to create more inclusive and culturally diverse online learning environments.	1	2	3 ✓	4	5

In the case of your agreement on sharing your answer/ points of views for questions below, please write down your comment/ point of view for all questions or any question that you prefer:

1. Did you experience the use of AI and AI-powered chatbots, such as ChatGPT and for what purpose?

   I was used Chatgpt for translating the language I am not familiar.

2. How do you perceive the role of AI and AI-powered machines in bridging communication gaps between people from different cultures?

   Firstly, it provids tools for different culture people to ~~[illegible]~~ ~~got through each other~~, start communication without ~~ignoring~~ language limitation.

   Secondly, based on AI database, it can quickly provide some basic information to learn the other culture which

CS CamScanner

No (73) [illegible]

3. Have you experienced and observed any instances where AI has been successful in facilitating cross-cultural understanding and collaboration in mediated communication?

No. don't have related experience

4. In what ways do you think AI can help overcome language barriers and promote more inclusive communication among diverse groups?

when we use correct and some degree formal sentences

5. Are there any cultural biases or stereotypes that you have noticed in AI-powered language tools or communication platforms?

Sorry I don't.

6. How can universities and educational institutions leverage AI to create more inclusive and culturally diverse online learning environments?

Firstly, maybe enhance AI database or let AI learn more culture customs.
Second, maybe ~~provid~~ provide AI learning workshop to tea[illegible] student how to correctly and efficiently use AI.

Thank you for your time and participation

CS CamScanner

Participant No (80) [illegible]

UNIVERSITI MALAYA

Depart of Media and Communication Studies

AI and Mediated Intercultural Communication Questionnaire

Dear UM Student/ Researcher,

Through this survey, we want to assess the probable effects of Artificial Intelligence (AI) on mediated communication among people from different cultures. This instrument which been developed by the help of the existing literature and ChatGPT, has 15 structured items with five (5) options per item and six (6) open-ended questions. Your participation in the survey is voluntary and the personal identifications of the participants will be treated as strictly confidential.

Thank you for your cooperation.

The researchers

A. Demographic Information

1. Gender: ☐ Male ☐ Female ✓

2. Age: 25

3. Nationality: China

4. Level of education: Postgraduate-MA

5. AI/ ChatGPT experience: ☐ Yes ✓ ☐ No

B. Intercultural Sensitivity Scale

Directions: The current AI-ICC questionnaire includes 15 items/ statements with five (5) options per item, as: 1 – Strongly agree; 2 – Agree, 3 – Uncertain; 4 – Disagree, and 5 – Strongly disagree.	Strongly agree	Agree	Uncertain	Disagree	Strongly disagree
1. AI and AI-powered machines can be helpful in bridging communication gaps between people from different cultures.	1 ✓	2	3	4	5
2. So far, AI has been successful in facilitating cross-cultural understanding and collaboration in mediated communication.	1	2	3	4 ✓	5
3. AI can help overcome language barriers and promote more inclusive communication among diverse groups.	1 ✓	2	3	4	5

CamScanner

No (80) & P(2)

4. There some cultural biases and stereotypes in AI-powered language tools and communication platforms.	1	2 ✓	3	4	5
5. AI has affected the localization and adaptation of content for different cultural audiences in digital communication.	1	2	3	4 ✓	5
6. I encountered any challenges in using AI-powered translation tools when communicating with people from different cultural backgrounds.	1	2 ✓	3	4	5
7. AI has the potential to promote cultural exchange and appreciation by enabling easier access to diverse perspectives and information.	1	2 ✓	3	4	5
8. AI-powered machines and chatbots should be programmed to respect and adapt to cultural norms and communication styles while interacting with users from different cultures.	1	2 ✓	3	4	5
9. There are some potential risks and concerns regarding AI's role in cross-cultural communication, such as perpetuating cultural stereotypes and misinterpretations.	1	2 ✓	3	4	5
10. I have participated in some cross-cultural virtual collaborations that relied on AI for communication and coordination.	1	2	3	4 ✓	5
11. I think AI can enhance intercultural learning experiences and foster empathy among individuals from different cultural backgrounds.	1	2	3	4 ✓	5
12. AI-powered virtual reality (VR) and augmented reality (AR) technologies can impact cross-cultural communication and understanding.	1	2 ✓	3	4	5
13. Somehow AI-generated content may unintentionally offend and misled individuals from specific cultural backgrounds.	1	2 ✓	3	4	5
14. Ethical considerations should be taken into account when designing AI-powered communication tools that cater to diverse cultural contexts.	1	2 ✓	3	4	5
15. Universities and educational institutions leverage AI to create more inclusive and culturally diverse online learning environments.	1	2 ✓	3	4	5

In the case of your agreement on sharing your answer/ points of views for questions below, please write down your comment/ point of view for all questions or any question that you prefer:

1. Did you experience the use of AI and AI-powered chatbots, such as ChatGPT and for what purpose?

   For the work and assignment.

2. How do you perceive the role of AI and AI-powered machines in bridging communication gaps between people from different cultures?

   It's useful but not very useful.

CamScanner

No (80) & (P3)

3. Have you experienced and observed any instances where AI has been successful in facilitating cross-cultural understanding and collaboration in mediated communication?

Not yet

4. In what ways do you think AI can help overcome language barriers and promote more inclusive communication among diverse groups?

5. Are there any cultural biases or stereotypes that you have noticed in AI-powered language tools or communication platforms?

6. How can universities and educational institutions leverage AI to create more inclusive and culturally diverse online learning environments?

Thank you for your time and participation

CamScanner

Participant No (81) C P(1)

UNIVERSITI MALAYA

Depart of Media and Communication Studies

AI and Mediated Intercultural Communication Questionnaire

Dear UM Student/ Researcher,

Through this survey, we want to assess the probable effects of Artificial Intelligence (AI) on mediated communication among people from different cultures. This instrument which been developed by the help of the existing literature and ChatGPT, has 15 structured items with five (5) options per item and six (6) open-ended questions. Your participation in the survey is voluntary and the personal identifications of the participants will be treated as strictly confidential.

Thank you for your cooperation.

The researchers

A. Demographic Information

1. Gender: ☐ Male ✓ Female

2. Age: 40

3. Nationality: CHINA

4. Level of education: PhD.

5. AI/ ChatGPT experience: ✓ Yes ☐ No

B. Intercultural Sensitivity Scale

**Directions:** The current AI-ICC questionnaire includes 15 items/ statements with five (5) options per item, as: 1 – Strongly agree; 2 – Agree, 3 – Uncertain; 4 – Disagree, and 5 – Strongly disagree.	Strongly agree	Agree	Uncertain	Disagree	Strongly disagree
1. AI and AI-powered machines can be helpful in bridging communication gaps between people from different cultures.	1 ✓	2	3	4	5
2. So far, AI has been successful in facilitating cross-cultural understanding and collaboration in mediated communication.	1	2 ✓	3	4	5
3. AI can help overcome language barriers and promote more inclusive communication among diverse groups.	1 ✓	2	3	4	5

CamScanner

No (81) & 82

4. There some cultural biases and stereotypes in AI-powered language tools and communication platforms.	1	2	3 ✓	4	5
5. AI has affected the localization and adaptation of content for different cultural audiences in digital communication.	1	2	3 ✓	4	5
6. I encountered any challenges in using AI-powered translation tools when communicating with people from different cultural backgrounds.	1 ✓	2	3	4	5
7. AI has the potential to promote cultural exchange and appreciation by enabling easier access to diverse perspectives and information.	1	2 ✓	3	4	5
8. AI-powered machines and chatbots should be programmed to respect and adapt to cultural norms and communication styles while interacting with users from different cultures.	1	2	3 ✓	4	5
9. There are some potential risks and concerns regarding AI's role in cross-cultural communication, such as perpetuating cultural stereotypes and misinterpretations.	1	2	3 ✓	4	5
10. I have participated in some cross-cultural virtual collaborations that relied on AI for communication and coordination.	1	2	3 ✓	4	5
11. I think AI can enhance intercultural learning experiences and foster empathy among individuals from different cultural backgrounds.	1	2	3 ✓	4	5
12. AI-powered virtual reality (VR) and augmented reality (AR) technologies can impact cross-cultural communication and understanding.	1	2 ✓	3	4	5
13. Somehow AI-generated content may unintentionally offend and misled individuals from specific cultural backgrounds.	1	2	3	4 ✓	5
14. Ethical considerations should be taken into account when designing AI-powered communication tools that cater to diverse cultural contexts.	1	2	3 ✓	4	5
15. Universities and educational institutions leverage AI to create more inclusive and culturally diverse online learning environments.	1	2	3 ✓	4	5

In the case of your agreement on sharing your answer/ points of views for questions below, please write down your comment/ point of view for all questions or any question that you prefer:

1. Did you experience the use of AI and AI-powered chatbots, such as ChatGPT and for what purpose?

   Language translation

   literature conclusion

2. How do you perceive the role of AI and AI-powered machines in bridging communication gaps between people from different cultures?

CamScanner

No (81) E, P3

3. Have you experienced and observed any instances where AI has been successful in facilitating cross-cultural understanding and collaboration in mediated communication?

No

4. In what ways do you think AI can help overcome language barriers and promote more inclusive communication among diverse groups?

Social media chat way.

5. Are there any cultural biases or stereotypes that you have noticed in AI-powered language tools or communication platforms?

No.

6. How can universities and educational institutions leverage AI to create more inclusive and culturally diverse online learning environments?

don't know -

Thank you for your time and participation

CS CamScanner

Participant No (82) ♀ P①

UNIVERSITI
MALAYA

Depart of Media and Communication Studies

AI and Mediated Intercultural Communication Questionnaire

Dear UM Student/ Researcher,

Through this survey, we want to assess the probable effects of Artificial Intelligence (AI) on mediated communication among people from different cultures. This instrument which been developed by the help of the existing literature and ChatGPT, has 15 structured items with five (5) options per item and six (6) open-ended questions. Your participation in the survey is voluntary and the personal identifications of the participants will be treated as strictly confidential.

Thank you for your cooperation.

The researchers

A. Demographic Information

1. Gender: ☐ Male ☑ Female

2. Age: 27

3. Nationality: China

4. Level of education: Master

5. AI/ ChatGPT experience: ☑ Yes ☐ No

B. Intercultural Sensitivity Scale

Directions: The current AI-ICC questionnaire includes 15 items/ statements with five (5) options per item, as: 1 – Strongly agree; 2 – Agree, 3 – Uncertain; 4 – Disagree, and 5 – Strongly disagree.	Strongly agree	Agree	Uncertain	Disagree	Strongly disagree
1. AI and AI-powered machines can be helpful in bridging communication gaps between people from different cultures.	1 ✓	2	3	4	5
2. So far, AI has been successful in facilitating cross-cultural understanding and collaboration in mediated communication.	1 ✓	2	3	4	5
3. AI can help overcome language barriers and promote more inclusive communication among diverse groups.	1 ✓	2	3	4	5

CamScanner

No (82) [illegible] P2

4. There some cultural biases and stereotypes in AI-powered language tools and communication platforms.	1	2 ✓	3	4	5
5. AI has affected the localization and adaptation of content for different cultural audiences in digital communication.	1	2	3 ✓	4	5
6. I encountered any challenges in using AI-powered translation tools when communicating with people from different cultural backgrounds.	1	2	3	4 ✓	5
7. AI has the potential to promote cultural exchange and appreciation by enabling easier access to diverse perspectives and information.	1	2	3 ✓	4	5
8. AI-powered machines and chatbots should be programmed to respect and adapt to cultural norms and communication styles while interacting with users from different cultures.	1 ✓	2	3	4	5
9. There are some potential risks and concerns regarding AI's role in cross-cultural communication, such as perpetuating cultural stereotypes and misinterpretations.	1	2	3 ✓	4	5
10. I have participated in some cross-cultural virtual collaborations that relied on AI for communication and coordination.	1	2 ✓	3	4	5
11. I think AI can enhance intercultural learning experiences and foster empathy among individuals from different cultural backgrounds.	1	2	3 ✓	4	5
12. AI-powered virtual reality (VR) and augmented reality (AR) technologies can impact cross-cultural communication and understanding.	1	2 ✓	3	4	5
13. Somehow AI-generated content may unintentionally offend and misled individuals from specific cultural backgrounds.	1	2	3 ✓	4	5
14. Ethical considerations should be taken into account when designing AI-powered communication tools that cater to diverse cultural contexts.	1 ✓	2	3	4	5
15. Universities and educational institutions leverage AI to create more inclusive and culturally diverse online learning environments.	1	2 ✓	3	4	5

In the case of your agreement on sharing your answer/ points of views for questions below, please write down your comment/ point of view for all questions or any question that you prefer:

1. Did you experience the use of AI and AI-powered chatbots, such as ChatGPT and for what purpose?

   Yes,

   Purpose: seek information, ask question, get assisteance with write tasks

2. How do you perceive the role of AI and AI-powered machines in bridging communication gaps between people from different cultures?

   Can reduce the influence of cultural bias in the direct conversations.

CS CamScanner

No (82) E1 P3

3. Have you experienced and observed any instances where AI has been successful in facilitating cross-cultural understanding and collaboration in mediated communication?

Real-time translation.

4. In what ways do you think AI can help overcome language barriers and promote more inclusive communication among diverse groups?

Analyze the cultural context.

5. Are there any cultural biases or stereotypes that you have noticed in AI-powered language tools or communication platforms?

No.

6. How can universities and educational institutions leverage AI to create more inclusive and culturally diverse online learning environments?

Provide multiple content and translation service.

Thank you for your time and participation

CamScanner

Participant No (83) Ec PD

UNIVERSITI MALAYA

Depart of Media and Communication Studies

AI and Mediated Intercultural Communication Questionnaire

Dear UM Student/ Researcher,

Through this survey, we want to assess the probable effects of Artificial Intelligence (AI) on mediated communication among people from different cultures. This instrument which been developed by the help of the existing literature and ChatGPT, has 15 structured items with five (5) options per item and six (6) open-ended questions. Your participation in the survey is voluntary and the personal identifications of the participants will be treated as strictly confidential.

Thank you for your cooperation.

The researchers

A. Demographic Information

1. Gender: ☐ Male ☑ Female

2. Age: 25

3. Nationality: MALAYSIAN

4. Level of education: PHD

5. AI/ ChatGPT experience: ☑ Yes ☑ No

B. Intercultural Sensitivity Scale

**Directions:** The current AI-ICC questionnaire includes 15 items/ statements with five (5) options per item, as: 1 – Strongly agree; 2 – Agree, 3 – Uncertain; 4 – Disagree, and 5 – Strongly disagree.	Strongly agree	Agree	Uncertain	Disagree	Strongly disagree
1. AI and AI-powered machines can be helpful in bridging communication gaps between people from different cultures.	1	2 ✓	3	4	5
2. So far, AI has been successful in facilitating cross-cultural understanding and collaboration in mediated communication.	1	2 ✓	3	4	5
3. AI can help overcome language barriers and promote more inclusive communication among diverse groups.	1	2 ✓	3	4	5

CamScanner

No (83) [illegible]

4. There some cultural biases and stereotypes in AI-powered language tools and communication platforms.	1	2	3 ✓	4	5
5. AI has affected the localization and adaptation of content for different cultural audiences in digital communication.	1	2 ✓	3	4	5
6. I encountered any challenges in using AI-powered translation tools when communicating with people from different cultural backgrounds.	1	2 ✓	3	4	5
7. AI has the potential to promote cultural exchange and appreciation by enabling easier access to diverse perspectives and information.	1	2 ✓	3	4	5
8. AI-powered machines and chatbots should be programmed to respect and adapt to cultural norms and communication styles while interacting with users from different cultures.	1	2 ✓	3	4	5
9. There are some potential risks and concerns regarding AI's role in cross-cultural communication, such as perpetuating cultural stereotypes and misinterpretations.	1	2 ✓	3	4	5
10. I have participated in some cross-cultural virtual collaborations that relied on AI for communication and coordination.	1	2	3	4 ✓	5
11. I think AI can enhance intercultural learning experiences and foster empathy among individuals from different cultural backgrounds.	1	2 ✓	3	4	5
12. AI-powered virtual reality (VR) and augmented reality (AR) technologies can impact cross-cultural communication and understanding.	1	2 ✓	3	4	5
13. Somehow AI-generated content may unintentionally offend and misled individuals from specific cultural backgrounds.	1	2	3 ✓	4	5
14. Ethical considerations should be taken into account when designing AI-powered communication tools that cater to diverse cultural contexts.	1	2 ✓	3	4	5
15. Universities and educational institutions leverage AI to create more inclusive and culturally diverse online learning environments.	1	2	3 ✓	4	5

In the case of your agreement on sharing your answer/ points of views for questions below, please write down your comment/ point of view for all questions or any question that you prefer:

1. Did you experience the use of AI and AI-powered chatbots, such as ChatGPT and for what purpose?

   I have never use it . But I think it work to find some information quickly

2. How do you perceive the role of AI and AI-powered machines in bridging communication gaps between people from different cultures?

   I think as intermediate because we can programe AI and AI powered in multi culture.

CS CamScanner

No (B3) & P3

3. Have you experienced and observed any instances where AI has been successful in facilitating cross-cultural understanding and collaboration in mediated communication?

not yet

4. In what ways do you think AI can help overcome language barriers and promote more inclusive communication among diverse groups?

online communication

5. Are there any cultural biases or stereotypes that you have noticed in AI-powered language tools or communication platforms?

not sure.

6. How can universities and educational institutions leverage AI to create more inclusive and culturally diverse online learning environments?

to make easy for the lecturer to get and give online notes for student.

Thank you for your time and participation

CamScanner

Partecipant No (84) & 10

UNIVERSITI MALAYA

Depart of Media and Communication Studies

AI and Mediated Intercultural Communication Questionnaire

Dear UM Student/ Researcher,

Through this survey, we want to assess the probable effects of Artificial Intelligence (AI) on mediated communication among people from different cultures. This instrument which been developed by the help of the existing literature and ChatGPT, has 15 structured items with five (5) options per item and six (6) open-ended questions. Your participation in the survey is voluntary and the personal identifications of the participants will be treated as strictly confidential.

Thank you for your cooperation.

The researchers

A. Demographic Information

1. Gender: ✓ Male ☐ Female

2. Age: 24

3. Nationality: China

4. Level of education: Master

5. AI/ ChatGPT experience: ✓ Yes ☐ No

B. Intercultural Sensitivity Scale

**Directions:** The current AI-ICC questionnaire includes 15 items/ statements with five (5) options per item, as: 1 – Strongly agree; 2 – Agree, 3 – Uncertain; 4 – Disagree, and 5 – Strongly disagree.	Strongly agree	Agree	Uncertain	Disagree	Strongly disagree
1. AI and AI-powered machines can be helpful in bridging communication gaps between people from different cultures.	1	2 ✓	3	4	5
2. So far, AI has been successful in facilitating cross-cultural understanding and collaboration in mediated communication.	1	2 ✓	3	4	5
3. AI can help overcome language barriers and promote more inclusive communication among diverse groups.	1 ✓	2	3	4	5

CamScanner

Participant No (84) S1
PO

4. There some cultural biases and stereotypes in AI-powered language tools and communication platforms.	1	2 ✓	3	4	5
5. AI has affected the localization and adaptation of content for different cultural audiences in digital communication.	1	2	3 ✓	4	5
6. I encountered any challenges in using AI-powered translation tools when communicating with people from different cultural backgrounds.	1 ✓	2	3	4	5
7. AI has the potential to promote cultural exchange and appreciation by enabling easier access to diverse perspectives and information.	1 ✓	2	3	4	5
8. AI-powered machines and chatbots should be programmed to respect and adapt to cultural norms and communication styles while interacting with users from different cultures.	1	2 ✓	3	4	5
9. There are some potential risks and concerns regarding AI's role in cross-cultural communication, such as perpetuating cultural stereotypes and misinterpretations.	1	2 ✓	3	4	5
10. I have participated in some cross-cultural virtual collaborations that relied on AI for communication and coordination.	1	2	3	4 ✓	5
11. I think AI can enhance intercultural learning experiences and foster empathy among individuals from different cultural backgrounds.	1	2 ✓	3	4	5
12. AI-powered virtual reality (VR) and augmented reality (AR) technologies can impact cross-cultural communication and understanding.	1	2 ✓	3	4	5
13. Somehow AI-generated content may unintentionally offend and misled individuals from specific cultural backgrounds.	1	2	3 ✓	4	5
14. Ethical considerations should be taken into account when designing AI-powered communication tools that cater to diverse cultural contexts.	1 ✓	2	3	4	5
15. Universities and educational institutions leverage AI to create more inclusive and culturally diverse online learning environments.	1	2 ✓	3	4	5

In the case of your agreement on sharing your answer/ points of views for questions below, please write down your comment/ point of view for all questions or any question that you prefer:

1. Did you experience the use of AI and AI-powered chatbots, such as ChatGPT and for what purpose?

   Yes. ChatGPT helps me solve some problems.

2. How do you perceive the role of AI and AI-powered machines in bridging communication gaps between people from different cultures?

   AI as an aid.

CamScanner

No (84) Ec P3
(84)

3. Have you experienced and observed any instances where AI has been successful in facilitating cross-cultural understanding and collaboration in mediated communication?

Not Yet.

4. In what ways do you think AI can help overcome language barriers and promote more inclusive communication among diverse groups?

More idiomatic translation

5. Are there any cultural biases or stereotypes that you have noticed in AI-powered language tools or communication platforms?

Chat GPT

6. How can universities and educational institutions leverage AI to create more inclusive and culturally diverse online learning environments?

Apply it reasonablly

Thank you for your time and participation

CS CamScanner

Participant No 85 E, P①

UNIVERSITI MALAYA

Depart of Media and Communication Studies

AI and Mediated Intercultural Communication Questionnaire

Dear UM Student/ Researcher,

Through this survey, we want to assess the probable effects of Artificial Intelligence (AI) on mediated communication among people from different cultures. This instrument which been developed by the help of the existing literature and ChatGPT, has 15 structured items with five (5) options per item and six (6) open-ended questions. Your participation in the survey is voluntary and the personal identifications of the participants will be treated as strictly confidential.

Thank you for your cooperation.

The researchers

A. Demographic Information

1. Gender: ☐ Male ☑ Female

2. Age: 31

3. Nationality: China

4. Level of education: PHD

5. AI/ ChatGPT experience: ☑ Yes ☐ No

B. Intercultural Sensitivity Scale

Directions: The current AI-ICC questionnaire includes 15 items/ statements with five (5) options per item, as: 1 – Strongly agree; 2 – Agree, 3 – Uncertain; 4 – Disagree, and 5 – Strongly disagree.	Strongly agree	Agree	Uncertain	Disagree	Strongly disagree
1. AI and AI-powered machines can be helpful in bridging communication gaps between people from different cultures.	1	2	3 ✓	4	5
2. So far, AI has been successful in facilitating cross-cultural understanding and collaboration in mediated communication.	1	2	3 ✓	4	5
3. AI can help overcome language barriers and promote more inclusive communication among diverse groups.	1	2	3 ✓	4	5

CamScanner

Participant No (85) & P2

4. There some cultural biases and stereotypes in AI-powered language tools and communication platforms.	1	2 ✓	3	4	5
5. AI has affected the localization and adaptation of content for different cultural audiences in digital communication.	1	2 ✓	3	4	5
6. I encountered any challenges in using AI-powered translation tools when communicating with people from different cultural backgrounds.	1	2 ✓	3	4	5
7. AI has the potential to promote cultural exchange and appreciation by enabling easier access to diverse perspectives and information.	1	2 ✓	3	4	5
8. AI-powered machines and chatbots should be programmed to respect and adapt to cultural norms and communication styles while interacting with users from different cultures.	1 ✓	2	3	4	5
9. There are some potential risks and concerns regarding AI's role in cross-cultural communication, such as perpetuating cultural stereotypes and misinterpretations.	1	2	3 ✓	4	5
10. I have participated in some cross-cultural virtual collaborations that relied on AI for communication and coordination.	1	2	3 ✓	4	5
11. I think AI can enhance intercultural learning experiences and foster empathy among individuals from different cultural backgrounds.	1	2 ✓	3	4	5
12. AI-powered virtual reality (VR) and augmented reality (AR) technologies can impact cross-cultural communication and understanding.	1	2 ✓	3	4	5
13. Somehow AI-generated content may unintentionally offend and misled individuals from specific cultural backgrounds.	1	2 ✓	3	4	5
14. Ethical considerations should be taken into account when designing AI-powered communication tools that cater to diverse cultural contexts.	1	2 ✓	3	4	5
15. Universities and educational institutions leverage AI to create more inclusive and culturally diverse online learning environments.	1 ✓	2	3	4	5

In the case of your agreement on sharing your answer/ points of views for questions below, please write down your comment/ point of view for all questions or any question that you prefer:

1. Did you experience the use of AI and AI-powered chatbots, such as ChatGPT and for what purpose?

   Yes in order to write thesis and paiting.

2. How do you perceive the role of AI and AI-powered machines in bridging communication gaps between people from different cultures?

   I have no idea.

CamScanner

No (85) E P3

3. Have you experienced and observed any instances where AI has been successful in facilitating cross-cultural understanding and collaboration in mediated communication?

No

4. In what ways do you think AI can help overcome language barriers and promote more inclusive communication among diverse groups?

I have no idea.

5. Are there any cultural biases or stereotypes that you have noticed in AI-powered language tools or communication platforms?

No.

6. How can universities and educational institutions leverage AI to create more inclusive and culturally diverse online learning environments?

I don't know.

Thank you for your time and participation

CS CamScanner

Participant No (86) Er PO

UNIVERSITI MALAYA

Depart of Media and Communication Studies

AI and Mediated Intercultural Communication Questionnaire

Dear UM Student/ Researcher,

Through this survey, we want to assess the probable effects of Artificial Intelligence (AI) on mediated communication among people from different cultures. This instrument which been developed by the help of the existing literature and ChatGPT, has 15 structured items with five (5) options per item and six (6) open-ended questions. Your participation in the survey is voluntary and the personal identifications of the participants will be treated as strictly confidential.

Thank you for your cooperation.

The researchers

A. Demographic Information

1. Gender: ☐ Male ☑ Female

2. Age: 23

3. Nationality: Oman.

4. Level of education: Master

5. AI/ ChatGPT experience: ☐ Yes ☑ No

B. Intercultural Sensitivity Scale

**Directions:** The current AI-ICC questionnaire includes 15 items/ statements with five (5) options per item, as: 1 – Strongly agree; 2 – Agree, 3 – Uncertain; 4 – Disagree, and 5 – Strongly disagree.	Strongly agree	Agree	Uncertain	Disagree	Strongly disagree
1. AI and AI-powered machines can be helpful in bridging communication gaps between people from different cultures.	1 ✓	2	3	4	5
2. So far, AI has been successful in facilitating cross-cultural understanding and collaboration in mediated communication.	1 ✓	2	3	4	5
3. AI can help overcome language barriers and promote more inclusive communication among diverse groups.	1 ✓	2	3	4	5

CamScanner

Partcipant No (86) E 12

4. There some cultural biases and stereotypes in AI-powered language tools and communication platforms.	1	2 ✓	3	4	5
5. AI has affected the localization and adaptation of content for different cultural audiences in digital communication.	1	2	3 ✓	4	5
6. I encountered any challenges in using AI-powered translation tools when communicating with people from different cultural backgrounds.	1	2 ✓	3	4	5
7. AI has the potential to promote cultural exchange and appreciation by enabling easier access to diverse perspectives and information.	1	2 ✓	3	4	5
8. AI-powered machines and chatbots should be programmed to respect and adapt to cultural norms and communication styles while interacting with users from different cultures.	1	2 ✓	3	4	5
9. There are some potential risks and concerns regarding AI's role in cross-cultural communication, such as perpetuating cultural stereotypes and misinterpretations.	1	2 ✓	3	4	5
10. I have participated in some cross-cultural virtual collaborations that relied on AI for communication and coordination.	1	2	3	4 ✓	5
11. I think AI can enhance intercultural learning experiences and foster empathy among individuals from different cultural backgrounds.	1	2	3 ✓	4	5
12. AI-powered virtual reality (VR) and augmented reality (AR) technologies can impact cross-cultural communication and understanding.	1 ✓	2	3	4	5
13. Somehow AI-generated content may unintentionally offend and misled individuals from specific cultural backgrounds.	1 ✓	2	3	4	5
14. Ethical considerations should be taken into account when designing AI-powered communication tools that cater to diverse cultural contexts.	1 ✓	2	3	4	5
15. Universities and educational institutions leverage AI to create more inclusive and culturally diverse online learning environments.	1	2 ✓	3	4	5

In the case of your agreement on sharing your answer/ points of views for questions below, please write down your comment/ point of view for all questions or any question that you prefer:

1. Did you experience the use of AI and AI-powered chatbots, such as ChatGPT and for what purpose?

   NO

2. How do you perceive the role of AI and AI-powered machines in bridging communication gaps between people from different cultures?

   It is good. can help to understand more culture

CS CamScanner

Participant no 86 P3

3. Have you experienced and observed any instances where AI has been successful in facilitating cross-cultural understanding and collaboration in mediated communication?

No

4. In what ways do you think AI can help overcome language barriers and promote more inclusive communication among diverse groups?

more knowledge.

5. Are there any cultural biases or stereotypes that you have noticed in AI-powered language tools or communication platforms?

Yes.

6. How can universities and educational institutions leverage AI to create more inclusive and culturally diverse online learning environments?

No idea

Thank you for your time and participation

CamScanner

Participant No (87) E P(1)

UNIVERSITI MALAYA

Depart of Media and Communication Studies

AI and Mediated Intercultural Communication Questionnaire

Dear UM Student/ Researcher,

Through this survey, we want to assess the probable effects of Artificial Intelligence (AI) on mediated communication among people from different cultures. This instrument which been developed by the help of the existing literature and ChatGPT, has 15 structured items with five (5) options per item and six (6) open-ended questions. Your participation in the survey is voluntary and the personal identifications of the participants will be treated as strictly confidential.

Thank you for your cooperation.

The researchers

A. Demographic Information

1. Gender: ☐ Male ☑ Female

2. Age: 30

3. Nationality: China.

4. Level of education: PhD

5. AI/ ChatGPT experience: ☑ Yes ☐ No

B. Intercultural Sensitivity Scale

Directions: The current AI-ICC questionnaire includes 15 items/ statements with five (5) options per item, as: 1 – Strongly agree; 2 – Agree, 3 – Uncertain; 4 – Disagree, and 5 – Strongly disagree.	Strongly agree	Agree	Uncertain	Disagree	Strongly
1. AI and AI-powered machines can be helpful in bridging communication gaps between people from different cultures.	1	2 ✓	3	4	5
2. So far, AI has been successful in facilitating cross-cultural understanding and collaboration in mediated communication.	1	2 ✓	3	4	5
3. AI can help overcome language barriers and promote more inclusive communication among diverse groups.	1	2 ✓	3	4	5

CamScanner

No (87) & P(2)

4. There some cultural biases and stereotypes in AI-powered language tools and communication platforms.	1	2	3 ✓	4	5
5. AI has affected the localization and adaptation of content for different cultural audiences in digital communication.	1	2	3 ✓	4	5
6. I encountered any challenges in using AI-powered translation tools when communicating with people from different cultural backgrounds.	1	2	3 ✓	4	5
7. AI has the potential to promote cultural exchange and appreciation by enabling easier access to diverse perspectives and information.	1	2 ✓	3	4	5
8. AI-powered machines and chatbots should be programmed to respect and adapt to cultural norms and communication styles while interacting with users from different cultures.	1	2	3 ✓	4	5
9. There are some potential risks and concerns regarding AI's role in cross-cultural communication, such as perpetuating cultural stereotypes and misinterpretations.	1	2	3 ✓	4	5
10. I have participated in some cross-cultural virtual collaborations that relied on AI for communication and coordination.	1	2	3 ✓	4	5
11. I think AI can enhance intercultural learning experiences and foster empathy among individuals from different cultural backgrounds.	1	2 ✓	3	4	5
12. AI-powered virtual reality (VR) and augmented reality (AR) technologies can impact cross-cultural communication and understanding.	1	2 ✓	3	4	5
13. Somehow AI-generated content may unintentionally offend and misled individuals from specific cultural backgrounds.	1	2	3 ✓	4	5
14. Ethical considerations should be taken into account when designing AI-powered communication tools that cater to diverse cultural contexts.	1 ✓	2	3	4	5
15. Universities and educational institutions leverage AI to create more inclusive and culturally diverse online learning environments.	1	2 ✓	3	4	5

In the case of your agreement on sharing your answer/ points of views for questions below, please write down your comment/ point of view for all questions or any question that you prefer:

1. Did you experience the use of AI and AI-powered chatbots, such as ChatGPT and for what purpose?

Yes
Information searching

2. How do you perceive the role of AI and AI-powered machines in bridging communication gaps between people from different cultures?

No idea.

CamScanner

NO (87) E P3

3. Have you experienced and observed any instances where AI has been successful in facilitating cross-cultural understanding and collaboration in mediated communication?

No

4. In what ways do you think AI can help overcome language barriers and promote more inclusive communication among diverse groups?

No idea.

5. Are there any cultural biases or stereotypes that you have noticed in AI-powered language tools or communication platforms?

No

6. How can universities and educational institutions leverage AI to create more inclusive and culturally diverse online learning environments?

By different tools of methodology to support.

Thank you for your time and participation

CS CamScanner

Participant No (88) & PO

UNIVERSITI MALAYA

Depart of Media and Communication Studies

AI and Mediated Intercultural Communication Questionnaire

Dear UM Student/ Researcher,

Through this survey, we want to assess the probable effects of Artificial Intelligence (AI) on mediated communication among people from different cultures. This instrument which been developed by the help of the existing literature and ChatGPT, has 15 structured items with five (5) options per item and six (6) open-ended questions. Your participation in the survey is voluntary and the personal identifications of the participants will be treated as strictly confidential.

Thank you for your cooperation.

The researchers

A. Demographic Information

1. Gender: ☐ Male ✓ Female

2. Age: 25

3. Nationality: China

4. Level of education: PHD

5. AI/ ChatGPT experience: ✓ Yes ☐ No

B. Intercultural Sensitivity Scale

Directions: The current AI-ICC questionnaire includes 15 items/ statements with five (5) options per item, as: 1 – Strongly agree; 2 – Agree, 3 – Uncertain; 4 – Disagree, and 5 – Strongly disagree.	Strongly agree	Agree	Uncertain	Disagree	Strongly disagree
1. AI and AI-powered machines can be helpful in bridging communication gaps between people from different cultures.	1	2	3	4 ✓	5
2. So far, AI has been successful in facilitating cross-cultural understanding and collaboration in mediated communication.	1	2	3	4 ✓	5
3. AI can help overcome language barriers and promote more inclusive communication among diverse groups.	1	2	3	4 ✓	5

CamScanner

No (88) & P0

4. There some cultural biases and stereotypes in AI-powered language tools and communication platforms.	1	2	3 ✓	4	5
5. AI has affected the localization and adaptation of content for different cultural audiences in digital communication.	1	2	3 ✓	4	5
6. I encountered any challenges in using AI-powered translation tools when communicating with people from different cultural backgrounds.	1	2 ✓	3	4	5
7. AI has the potential to promote cultural exchange and appreciation by enabling easier access to diverse perspectives and information.	1	2	3	4 ✓	5
8. AI-powered machines and chatbots should be programmed to respect and adapt to cultural norms and communication styles while interacting with users from different cultures.	1 ✓	2	3	4	5
9. There are some potential risks and concerns regarding AI's role in cross-cultural communication, such as perpetuating cultural stereotypes and misinterpretations.	1	2 ✓	3	4	5
10. I have participated in some cross-cultural virtual collaborations that relied on AI for communication and coordination.	1	2	3	4	5 ✓
11. I think AI can enhance intercultural learning experiences and foster empathy among individuals from different cultural backgrounds.	1	2 ✓	3	4	5
12. AI-powered virtual reality (VR) and augmented reality (AR) technologies can impact cross-cultural communication and understanding.	1	2 ✓	3	4	5
13. Somehow AI-generated content may unintentionally offend and misled individuals from specific cultural backgrounds.	1	2 ✓	3	4	5
14. Ethical considerations should be taken into account when designing AI-powered communication tools that cater to diverse cultural contexts.	1 ✓	2	3	4	5
15. Universities and educational institutions leverage AI to create more inclusive and culturally diverse online learning environments.	1	2	3	4 ✓	5

In the case of your agreement on sharing your answer/ points of views for questions below, please write down your comment/ point of view for all questions or any question that you prefer:

1. Did you experience the use of AI and AI-powered chatbots, such as ChatGPT and for what purpose?

Yes

2. How do you perceive the role of AI and AI-powered machines in bridging communication gaps between people from different cultures?

Not very useful

CamScanner

No (88) E P3

3. Have you experienced and observed any instances where AI has been successful in facilitating cross-cultural understanding and collaboration in mediated communication?

No

4. In what ways do you think AI can help overcome language barriers and promote more inclusive communication among diverse groups?

In language learning

5. Are there any cultural biases or stereotypes that you have noticed in AI-powered language tools or communication platforms?

Not yet.

6. How can universities and educational institutions leverage AI to create more inclusive and culturally diverse online learning environments?

Not sure.

Thank you for your time and participation

CamScanner

Partipant no (R3) = P10

UNIVERSITI MALAYA

Depart of Media and Communication Studies

AI and Mediated Intercultural Communication Questionnaire

Dear UM Student/ Researcher,

Through this survey, we want to assess the probable effects of Artificial Intelligence (AI) on mediated communication among people from different cultures. This instrument which been developed by the help of the existing literature and ChatGPT, has 15 structured items with five (5) options per item and six (6) open-ended questions. Your participation in the survey is voluntary and the personal identifications of the participants will be treated as strictly confidential.

Thank you for your cooperation.

The researchers

A. Demographic Information

1. Gender: Male ✓ Female

2. Age: 40

3. Nationality: MALAYSIA

4. Level of education: ~~SPM (Bachelor)~~ post-grado

5. AI/ ChatGPT experience: ✓ Yes No

B. Intercultural Sensitivity Scale

Directions: The current AI-ICC questionnaire includes 15 items/ statements with five (5) options per item, as: 1 – Strongly agree; 2 – Agree, 3 – Uncertain; 4 – Disagree, and 5 – Strongly disagree.	Strongly agree	Agree	Uncertain	Disagree	Strongly disagree
1. AI and AI-powered machines can be helpful in bridging communication gaps between people from different cultures.	1	2	(3)	4	5
2. So far, AI has been successful in facilitating cross-cultural understanding and collaboration in mediated communication.	1	2	(3)	4	5
3. AI can help overcome language barriers and promote more inclusive communication among diverse groups.	1	2	(3)	4	5

CamScanner

No (89) & P(2)

4. There some cultural biases and stereotypes in AI-powered language tools and communication platforms.	1	**2**	3	4	5
5. AI has affected the localization and adaptation of content for different cultural audiences in digital communication.	1	**2**	3	4	5
6. I encountered any challenges in using AI-powered translation tools when communicating with people from different cultural backgrounds.	1	2	**3**	4	5
7. AI has the potential to promote cultural exchange and appreciation by enabling easier access to diverse perspectives and information.	1	2	**3**	4	5
8. AI-powered machines and chatbots should be programmed to respect and adapt to cultural norms and communication styles while interacting with users from different cultures.	1	2	**3**	4	5
9. There are some potential risks and concerns regarding AI's role in cross-cultural communication, such as perpetuating cultural stereotypes and misinterpretations.	1	2	**3**	4	5
10. I have participated in some cross-cultural virtual collaborations that relied on AI for communication and coordination.	1	2	**3**	4	5
11. I think AI can enhance intercultural learning experiences and foster empathy among individuals from different cultural backgrounds.	1	**2**	3	4	5
12. AI-powered virtual reality (VR) and augmented reality (AR) technologies can impact cross-cultural communication and understanding.	1	**2**	3	4	5
13. Somehow AI-generated content may unintentionally offend and misled individuals from specific cultural backgrounds.	1	2	**3**	4	5
14. Ethical considerations should be taken into account when designing AI-powered communication tools that cater to diverse cultural contexts.	1	2	**3**	4	5
15. Universities and educational institutions leverage AI to create more inclusive and culturally diverse online learning environments.	1	**2**	3	4	5

In the case of your agreement on sharing your answer/ points of views for questions below, please write down your comment/ point of view for all questions or any question that you prefer:

1. Did you experience the use of AI and AI-powered chatbots, such as ChatGPT and for what purpose?

   No, I did not

2. How do you perceive the role of AI and AI-powered machines in bridging communication gaps between people from different cultures?

   I am not sure since I am ~~not~~ using AI

CamScanner

No (89) E, P3

3. Have you experienced and observed any instances where AI has been successful in facilitating cross-cultural understanding and collaboration in mediated communication?

Never exprienced

4. In what ways do you think AI can help overcome language barriers and promote more inclusive communication among diverse groups?

From others inf, they can help thn any understandnt on particlar subject ~~and~~ even from other languge.

5. Are there any cultural biases or stereotypes that you have noticed in AI-powered language tools or communication platforms?

I am not sure

6. How can universities and educational institutions leverage AI to create more inclusive and culturally diverse online learning environments?

Not sure.

Thank you for your time and participation

CS CamScanner

Participant No (90) &c P(1)

UNIVERSITI MALAYA

Depart of Media and Communication Studies

AI and Mediated Intercultural Communication Questionnaire

Dear UM Student/ Researcher,

Through this survey, we want to assess the probable effects of Artificial Intelligence (AI) on mediated communication among people from different cultures. This instrument which been developed by the help of the existing literature and ChatGPT, has 15 structured items with five (5) options per item and six (6) open-ended questions. Your participation in the survey is voluntary and the personal identifications of the participants will be treated as strictly confidential.

Thank you for your cooperation.

The researchers

A. Demographic Information

1. Gender: ☑ Male ☐ Female

2. Age: 35

3. Nationality: Malaysian

4. Level of education: Master

5. AI/ ChatGPT experience: ☑ Yes ☐ No

B. Intercultural Sensitivity Scale

**Directions:** The current AI-ICC questionnaire includes 15 items/ statements with five (5) options per item, as: 1 – Strongly agree; 2 – Agree, 3 – Uncertain; 4 – Disagree, and 5 – Strongly disagree.	Strongly agree	Agree	Uncertain	Disagree	Strongly disagree
1. AI and AI-powered machines can be helpful in bridging communication gaps between people from different cultures.	(1)	2	3	4	5
2. So far, AI has been successful in facilitating cross-cultural understanding and collaboration in mediated communication.	1	(2)	3	4	5
3. AI can help overcome language barriers and promote more inclusive communication among diverse groups.	1	(2)	3	4	5

CamScanner

No (30) [illegible]

4. There some cultural biases and stereotypes in AI-powered language tools and communication platforms.	1	**2**	3	4	5
5. AI has affected the localization and adaptation of content for different cultural audiences in digital communication.	1	**2**	3	4	5
6. I encountered any challenges in using AI-powered translation tools when communicating with people from different cultural backgrounds.	1	2	**3**	4	5
7. AI has the potential to promote cultural exchange and appreciation by enabling easier access to diverse perspectives and information.	1	**2**	3	4	5
8. AI-powered machines and chatbots should be programmed to respect and adapt to cultural norms and communication styles while interacting with users from different cultures.	**1**	2	3	4	5
9. There are some potential risks and concerns regarding AI's role in cross-cultural communication, such as perpetuating cultural stereotypes and misinterpretations.	1	**2**	3	4	5
10. I have participated in some cross-cultural virtual collaborations that relied on AI for communication and coordination.	1	2	**3**	4	5
11. I think AI can enhance intercultural learning experiences and foster empathy among individuals from different cultural backgrounds.	1	**2**	3	4	5
12. AI-powered virtual reality (VR) and augmented reality (AR) technologies can impact cross-cultural communication and understanding.	1	**2**	3	4	5
13. Somehow AI-generated content may unintentionally offend and misled individuals from specific cultural backgrounds.	1	**2**	3	4	5
14. Ethical considerations should be taken into account when designing AI-powered communication tools that cater to diverse cultural contexts.	**1**	2	3	4	5
15. Universities and educational institutions leverage AI to create more inclusive and culturally diverse online learning environments.	**1**	2	3	4	5

In the case of your agreement on sharing your answer/ points of views for questions below, please write down your comment/ point of view for all questions or any question that you prefer:

1. Did you experience the use of AI and AI-powered chatbots, such as ChatGPT and for what purpose?

   Yes, for learning and educational purposes.

2. How do you perceive the role of AI and AI-powered machines in bridging communication gaps between people from different cultures?

   It can definitely help to overcome the language barriers, but whether it is sensitive to cultural differences is highly in doubt at the moment.

CamScanner

No (90) E, P (3)

3. Have you experienced and observed any instances where AI has been successful in facilitating cross-cultural understanding and collaboration in mediated communication?

No so far.

4. In what ways do you think AI can help overcome language barriers and promote more inclusive communication among diverse groups?

- Real-time translation
- Translation of knowledge & literature.

5. Are there any cultural biases or stereotypes that you have noticed in AI-powered language tools or communication platforms?

- As it is developed by Western countries, I think some of the content or answers may be biased in a pro-Western manner / colonial mindset.

6. How can universities and educational institutions leverage AI to create more inclusive and culturally diverse online learning environments?

First of all, universities should regulate the use of AI, rather than banning it outright. Secondly, AI could be used to enhance communication between students & lecturers, especially foreign students.

Thank you for your time and participation

CamScanner

Participant No (91) & P(1)

UNIVERSITI MALAYA

Depart of Media and Communication Studies

AI and Mediated Intercultural Communication Questionnaire

Dear UM Student/ Researcher,

Through this survey, we want to assess the probable effects of Artificial Intelligence (AI) on mediated communication among people from different cultures. This instrument which been developed by the help of the existing literature and ChatGPT, has 15 structured items with five (5) options per item and six (6) open-ended questions. Your participation in the survey is voluntary and the personal identifications of the participants will be treated as strictly confidential.

Thank you for your cooperation.

The researchers

A. Demographic Information

1. Gender: ☐ Male ☑ Female

2. Age: 26

3. Nationality: Chinese

4. Level of education: Master

5. AI/ ChatGPT experience: ☑ Yes ☐ No

B. Intercultural Sensitivity Scale

**Directions:** The current AI-ICC questionnaire includes 15 items/ statements with five (5) options per item, as: 1 – Strongly agree; 2 – Agree, 3 – Uncertain; 4 – Disagree, and 5 – Strongly disagree.	Strongly agree	Agree	Uncertain	Disagree	Strongly disagree
1. AI and AI-powered machines can be helpful in bridging communication gaps between people from different cultures.	1	2 ✓	3	4	5
2. So far, AI has been successful in facilitating cross-cultural understanding and collaboration in mediated communication.	1	2 ✓	3	4	5
3. AI can help overcome language barriers and promote more inclusive communication among diverse groups.	1	2 ✓	3	4	5

CamScanner

No 91 & P2

4. There some cultural biases and stereotypes in AI-powered language tools and communication platforms.	1 ✓	2	3	4	5
5. AI has affected the localization and adaptation of content for different cultural audiences in digital communication.	1	2 ✓	3	4	5
6. I encountered any challenges in using AI-powered translation tools when communicating with people from different cultural backgrounds.	1	2 ✓	3	4	5
7. AI has the potential to promote cultural exchange and appreciation by enabling easier access to diverse perspectives and information.	1 ✓	2	3	4	5
8. AI-powered machines and chatbots should be programmed to respect and adapt to cultural norms and communication styles while interacting with users from different cultures.	1 ✓	2	3	4	5
9. There are some potential risks and concerns regarding AI's role in cross-cultural communication, such as perpetuating cultural stereotypes and misinterpretations.	1	2 ✓	3	4	5
10. I have participated in some cross-cultural virtual collaborations that relied on AI for communication and coordination.	1	2	3 ✓	4	5
11. I think AI can enhance intercultural learning experiences and foster empathy among individuals from different cultural backgrounds.	1 ✓	2	3	4	5
12. AI-powered virtual reality (VR) and augmented reality (AR) technologies can impact cross-cultural communication and understanding.	1 ✓	2	3	4	5
13. Somehow AI-generated content may unintentionally offend and misled individuals from specific cultural backgrounds.	1 ✓	2	3	4	5
14. Ethical considerations should be taken into account when designing AI-powered communication tools that cater to diverse cultural contexts.	1 ✓	2	3	4	5
15. Universities and educational institutions leverage AI to create more inclusive and culturally diverse online learning environments.	1 ✓	2	3	4	5

In the case of your agreement on sharing your answer/ points of views for questions below, please write down your comment/ point of view for all questions or any question that you prefer:

1. Did you experience the use of AI and AI-powered chatbots, such as ChatGPT and for what purpose?

Yes, for searching information and entertainment.

2. How do you perceive the role of AI and AI-powered machines in bridging communication gaps between people from different cultures?

It's smart and embrace diverse cultures so it would help to reduce misunderstanding

CamScanner

No (31) & P(3)

3. Have you experienced and observed any instances where AI has been successful in facilitating cross-cultural understanding and collaboration in mediated communication?

Yes, I saw my son chat with an AI device to ask information about other countries and learn the culture.

4. In what ways do you think AI can help overcome language barriers and promote more inclusive communication among diverse groups?

to include more interesting culture-related stories and conversations.

5. Are there any cultural biases or stereotypes that you have noticed in AI-powered language tools or communication platforms?

Probably yes.

6. How can universities and educational institutions leverage AI to create more inclusive and culturally diverse online learning environments?

Promote the advantage of AI and run more workshop to encourage people to discuss about AI and learn more about it. Besides, more technologies advances are importan

Thank you for your time and participation

CS CamScanner

Perticipants No 92 & PO

UNIVERSITI MALAYA

Depart of Media and Communication Studies

AI and Mediated Intercultural Communication Questionnaire

Dear UM Student/ Researcher,

Through this survey, we want to assess the probable effects of Artificial Intelligence (AI) on mediated communication among people from different cultures. This instrument which been developed by the help of the existing literature and ChatGPT, has 15 structured items with five (5) options per item and six (6) open-ended questions. Your participation in the survey is voluntary and the personal identifications of the participants will be treated as strictly confidential.

Thank you for your cooperation.

The researchers

A. Demographic Information

1. Gender: ☑ Male ☐ Female

2. Age: 35

3. Nationality: China

4. Level of education: Phd

5. AI/ ChatGPT experience: ☑ Yes ☐ No

B. Intercultural Sensitivity Scale

**Directions:** The current AI-ICC questionnaire includes 15 items/ statements with five (5) options per item, as: 1 – Strongly agree; 2 – Agree, 3 – Uncertain; 4 – Disagree, and 5 – Strongly disagree.	Strongly agree	Agree	Uncertain	Disagree	Strongly disagree
1. AI and AI-powered machines can be helpful in bridging communication gaps between people from different cultures.	1	2	3 ✓	4	5
2. So far, AI has been successful in facilitating cross-cultural understanding and collaboration in mediated communication.	1	2 ✓	3	4	5
3. AI can help overcome language barriers and promote more inclusive communication among diverse groups.	1 ✓	2	3	4	5

CamScanner

No (92) E P(2)

4. There some cultural biases and stereotypes in AI-powered language tools and communication platforms.	1	2	3 ✓	4	5
5. AI has affected the localization and adaptation of content for different cultural audiences in digital communication.	1	2	3 ✓	4	5
6. I encountered any challenges in using AI-powered translation tools when communicating with people from different cultural backgrounds.	1	2 ✓	3	4	5
7. AI has the potential to promote cultural exchange and appreciation by enabling easier access to diverse perspectives and information.	1	2 ✓	3	4	5
8. AI-powered machines and chatbots should be programmed to respect and adapt to cultural norms and communication styles while interacting with users from different cultures.	1	2 ✓	3	4	5
9. There are some potential risks and concerns regarding AI's role in cross-cultural communication, such as perpetuating cultural stereotypes and misinterpretations.	1	2	3	4 ✓	5
10. I have participated in some cross-cultural virtual collaborations that relied on AI for communication and coordination.	1	2 ✓	3	4	5
11. I think AI can enhance intercultural learning experiences and foster empathy among individuals from different cultural backgrounds.	1 ✓	2	3	4	5
12. AI-powered virtual reality (VR) and augmented reality (AR) technologies can impact cross-cultural communication and understanding.	1 ✓	2	3	4	5
13. Somehow AI-generated content may unintentionally offend and misled individuals from specific cultural backgrounds.	1 ✓	2	3	4	5
14. Ethical considerations should be taken into account when designing AI-powered communication tools that cater to diverse cultural contexts.	1 ✓	2	3	4	5
15. Universities and educational institutions leverage AI to create more inclusive and culturally diverse online learning environments.	1	2 ✓	3	4	5

In the case of your agreement on sharing your answer/ points of views for questions below, please write down your comment/ point of view for all questions or any question that you prefer:

1. Did you experience the use of AI and AI-powered chatbots, such as ChatGPT and for what purpose?

   I have used gpt to collect information and solve unknow problems

2. How do you perceive the role of AI and AI-powered machines in bridging communication gaps between people from different cultures?

   Now AI may just be a tool for cross-cultural learning and communicat. But the future may be all-encompassing.

CS CamScanner

No (92) 2 P(3)

3. Have you experienced and observed any instances where AI has been successful in facilitating cross-cultural understanding and collaboration in mediated communication?

Maybe just GPT.

4. In what ways do you think AI can help overcome language barriers and promote more inclusive communication among diverse groups?

In the design of audio products. such as. Siri.

5. Are there any cultural biases or stereotypes that you have noticed in AI-powered language tools or communication platforms?

None.

6. How can universities and educational institutions leverage AI to create more inclusive and culturally diverse online learning environments?

Improvements should be made in teaching language and content

Thank you for your time and participation

CamScanner

Partcipants No (93) [illegible]

UNIVERSITI MALAYA

Depart of Media and Communication Studies

AI and Mediated Intercultural Communication Questionnaire

Dear UM Student/ Researcher,

Through this survey, we want to assess the probable effects of Artificial Intelligence (AI) on mediated communication among people from different cultures. This instrument which been developed by the help of the existing literature and ChatGPT, has 15 structured items with five (5) options per item and six (6) open-ended questions. Your participation in the survey is voluntary and the personal identifications of the participants will be treated as strictly confidential.

Thank you for your cooperation.

The researchers

A. Demographic Information

1. Gender: ☑ Male ☐ Female

2. Age: 36

3. Nationality: CHINA

4. Level of education: PHD

5. AI/ ChatGPT experience: ☑ Yes ☐ No

B. Intercultural Sensitivity Scale

**Directions:** The current AI-ICC questionnaire includes 15 items/ statements with five (5) options per item, as: 1 – Strongly agree; 2 – Agree, 3 – Uncertain; 4 – Disagree, and 5 – Strongly disagree.	Strongly agree	Agree	Uncertain	Disagree	Strongly disagree
1. AI and AI-powered machines can be helpful in bridging communication gaps between people from different cultures.	1	2	3	4	5 ✓
2. So far, AI has been successful in facilitating cross-cultural understanding and collaboration in mediated communication.	1	2	3	4 ✓	5
3. AI can help overcome language barriers and promote more inclusive communication among diverse groups.	1	2	3	4 ✓	5

CamScanner

Participant no. [illegible] 28

4. There some cultural biases and stereotypes in AI-powered language tools and communication platforms.	1	2	3 ✓	4	5
5. AI was effective for localization and adaptation of content for different cultural audiences in digital communication.	1	2	3	4 ✓	5
6. I encountered any challenges in using AI-powered translation tools when communicating with people from different cultural backgrounds.	1	2	3 ✓	4	5
7. AI has the potential to promote cultural exchange and appreciation by enabling easier access to diverse perspectives and information.	1	2	3	4 ✓	5
8. AI-powered machines and chatbots should be programmed to respect and adapt to cultural norms and communication styles while interacting with users from different cultures.	1	2	3	4 ✓	5
9. There are some potential risks and concerns regarding AI's role in cross-cultural communication, such as perpetuating cultural stereotypes and misinterpretations.	1	2	3	4 ✓	5
10. I have participated in some cross-cultural virtual collaborations that relied on AI for communication and coordination.	1	2	3	4 ✓	5
11. I think AI can enhance intercultural learning experiences and foster empathy among individuals from different cultural backgrounds.	1	2	3	4	5 ✓
12. AI-powered virtual reality (VR) and augmented reality (AR) technologies can impact cross-cultural communication and understanding.	1	2	3	4 ✓	5
13. Sometimes AI-generated content may unintentionally offend and mislead individuals from specific cultural backgrounds.	1	2	3	4 ✓	5
14. Ethical considerations should be taken into account when designing AI-powered communication tools that cater to diverse cultural contexts.	1	2	3	4 ✓	5
15. Universities and educational institutions leverage AI to create more inclusive and culturally diverse online learning environments.	1	2	3 ✓	4	5

In the case of your agreement on sharing your answer points of views for questions below, please write down your comment/ point of view for all questions or any question that you prefer.

1. Did you experience the use of AI and AI-powered chatbots, such as ChatGPT and for what purpose?

Yes, find relevant knowledge quickly.

2. How do you perceive the role of AI and AI-powered machines in bridging communication gaps between people from different cultures?

It's very important. The use of AI can continue to expand for cross-cultural communication.

CamScanner

No 93 & P3

3. Have you experienced and observed any instances where AI has been successful in facilitating cross-cultural understanding and collaboration in mediated communication?

No.

4. In what ways do you think AI can help overcome language barriers and promote more inclusive communication among diverse groups?

AI can speed up communication between people through rapid translation in different languages.

5. Are there any cultural biases or stereotypes that you have noticed in AI-powered language tools or communication platforms?

No.

6. How can universities and educational institutions leverage AI to create more inclusive and culturally diverse online learning environments?

They should ~~increase~~ increase the use of AI, but should prevent such phenomena as academic cheating.

Thank you for your time and participation

CS CamScanner

Participant No (99) & 10

UNIVERSITI MALAYA

Depart of Media and Communication Studies

AI and Mediated Intercultural Communication Questionnaire

Dear UM Student/ Researcher,

Through this survey, we want to assess the probable effects of Artificial Intelligence (AI) on mediated communication among people from different cultures. This instrument which been developed by the help of the existing literature and ChatGPT, has 15 structured items with five (5) options per item and six (6) open-ended questions. Your participation in the survey is voluntary and the personal identifications of the participants will be treated as strictly confidential.

Thank you for your cooperation.

The researchers

A. Demographic Information

1. Gender: ☐ Male ☑ Female

2. Age: 29

3. Nationality: Malaysian

4. Level of education: ~~[illegible]~~ Post Degree

5. AI/ ChatGPT experience: ☑ Yes ☐ No

B. Intercultural Sensitivity Scale

Directions: The current AI-ICC questionnaire includes 15 items/ statements with five (5) options per item, as: 1 – Strongly agree; 2 – Agree, 3 – Uncertain; 4 – Disagree, and 5 – Strongly disagree.	Strongly agree	Agree	Uncertain	Disagree	Strongly disagree
1. AI and AI-powered machines can be helpful in bridging communication gaps between people from different cultures.	1	(2)	3	4	5
2. So far, AI has been successful in facilitating cross-cultural understanding and collaboration in mediated communication.	1	2	(3)	4	5
3. AI can help overcome language barriers and promote more inclusive communication among diverse groups.	1	(2)	3	4	5

CamScanner

No 94 & P2

4. There some cultural biases and stereotypes in AI-powered language tools and communication platforms.	1	(2)	3	4	5
5. AI has affected the localization and adaptation of content for different cultural audiences in digital communication.	1	(2)	3	4	5
6. I encountered any challenges in using AI-powered translation tools when communicating with people from different cultural backgrounds.	1	2	(3)	4	5
7. AI has the potential to promote cultural exchange and appreciation by enabling easier access to diverse perspectives and information.	1	(2)	3	4	5
8. AI-powered machines and chatbots should be programmed to respect and adapt to cultural norms and communication styles while interacting with users from different cultures.	1	(2)	3	4	5
9. There are some potential risks and concerns regarding AI's role in cross-cultural communication, such as perpetuating cultural stereotypes and misinterpretations.	(1)	2	3	4	5
10. I have participated in some cross-cultural virtual collaborations that relied on AI for communication and coordination.	1	2	3	(4)	5
11. I think AI can enhance intercultural learning experiences and foster empathy among individuals from different cultural backgrounds.	1	(2)	3	4	5
12. AI-powered virtual reality (VR) and augmented reality (AR) technologies can impact cross-cultural communication and understanding.	1	(2)	3	4	5
13. Somehow AI-generated content may unintentionally offend and misled individuals from specific cultural backgrounds.	(1)	2	3	4	5
14. Ethical considerations should be taken into account when designing AI-powered communication tools that cater to diverse cultural contexts.	(1)	2	3	4	5
15. Universities and educational institutions leverage AI to create more inclusive and culturally diverse online learning environments.	1	(2)	3	4	5

In the case of your agreement on sharing your answer/ points of views for questions below, please write down your comment/ point of view for all questions or any question that you prefer:

1. Did you experience the use of AI and AI-powered chatbots, such as ChatGPT and for what purpose?

   yes, more information on how to answer / techwiques to answer questions

2. How do you perceive the role of AI and AI-powered machines in bridging communication gaps between people from different cultures?

   I think it is possible if wied in the right manner and if it can propery give the right information

CS CamScanner

No (34) Er P(3)

3. Have you experienced and observed any instances where AI has been successful in facilitating cross-cultural understanding and collaboration in mediated communication?

yes

4. In what ways do you think AI can help overcome language barriers and promote more inclusive communication among diverse groups?

- provide tools that facilitate translation, interpretation & language learning

5. Are there any cultural biases or stereotypes that you have noticed in AI-powered language tools or communication platforms?

yes

6. How can universities and educational institutions leverage AI to create more inclusive and culturally diverse online learning environments?

- teach students to use AI for the right purposes :-

a) for automated captioning & subtitling

b) better understanding of concepts

Thank you for your time and participation

CamScanner

Partcipant No (95) & PO

UNIVERSITI MALAYA

Depart of Media and Communication Studies

AI and Mediated Intercultural Communication Questionnaire

Dear UM Student/ Researcher,

Through this survey, we want to assess the probable effects of Artificial Intelligence (AI) on mediated communication among people from different cultures. This instrument which been developed by the help of the existing literature and ChatGPT, has 15 structured items with five (5) options per item and six (6) open-ended questions. Your participation in the survey is voluntary and the personal identifications of the participants will be treated as strictly confidential.

Thank you for your cooperation.

The researchers

A. Demographic Information

1. Gender: ☐ Male ✓ Female

2. Age: 27

3. Nationality: CHINA

4. Level of education: PhD

5. AI/ ChatGPT experience: ✓ Yes ☐ No

B. Intercultural Sensitivity Scale

Directions: The current AI-ICC questionnaire includes 15 items/ statements with five (5) options per item, as: 1 – Strongly agree; 2 – Agree, 3 – Uncertain; 4 – Disagree, and 5 – Strongly disagree.	Strongly agree	Agree	Uncertain	Disagree	Strongly disagree
1. AI and AI-powered machines can be helpful in bridging communication gaps between people from different cultures.	1	2 ✓	3	4	5
2. So far, AI has been successful in facilitating cross-cultural understanding and collaboration in mediated communication.	1	2	3 ✓	4	5
3. AI can help overcome language barriers and promote more inclusive communication among diverse groups.	1	2	3 ✓	4	5

CamScanner

No (95) & R2

4. There some cultural biases and stereotypes in AI-powered language tools and communication platforms.	1	2	3	4	5
5. AI has affected the localization and adaptation of content for different cultural audiences in digital communication.	1	2	3	4	5
6. I encountered any challenges in using AI-powered translation tools when communicating with people from different cultural backgrounds.	1	2	3	4	5
7. AI has the potential to promote cultural exchange and appreciation by enabling easier access to diverse perspectives and information.	1	2	3	4	5
8. AI-powered machines and chatbots should be programmed to respect and adapt to cultural norms and communication styles while interacting with users from different cultures.	1	2	3	4	5
9. There are some potential risks and concerns regarding AI's role in cross-cultural communication, such as perpetuating cultural stereotypes and misinterpretations.	1	2	3	4	5
10. I have participated in some cross-cultural virtual collaborations that relied on AI for communication and coordination.	1	2	3	4	5
11. I think AI can enhance intercultural learning experiences and foster empathy among individuals from different cultural backgrounds.	1	2	3	4	5
12. AI-powered virtual reality (VR) and augmented reality (AR) technologies can impact cross-cultural communication and understanding.	1	2	3	4	5
13. Somehow AI-generated content may unintentionally offend and misled individuals from specific cultural backgrounds.	1	2	3	4	5
14. Ethical considerations should be taken into account when designing AI-powered communication tools that cater to diverse cultural contexts.	1	2	3	4	5
15. Universities and educational institutions leverage AI to create more inclusive and culturally diverse online learning environments.	1	2	3	4	5

In the case of your agreement on sharing your answer/ points of views for questions below, please write down your comment/ point of view for all questions or any question that you prefer:

1. Did you experience the use of AI and AI-powered chatbots, such as ChatGPT and for what purpose?

   Yes. For help me compelete my work.

2. How do you perceive the role of AI and AI-powered machines in bridging communication gaps between people from different cultures?

   It can help, but It cannot find the differences between different cultures specific.

CS CamScanner

No (95) ♀ P(3)

3. Have you experienced and observed any instances where AI has been successful in facilitating cross-cultural understanding and collaboration in mediated communication?

No

4. In what ways do you think AI can help overcome language barriers and promote more inclusive communication among diverse groups?

I think ChatGPT is good at English language probelmies
It still have long way to go to promote diverse groups.

5. Are there any cultural biases or stereotypes that you have noticed in AI-powered language tools or communication platforms?

No.

6. How can universities and educational institutions leverage AI to create more inclusive and culturally diverse online learning environments?

Use AI to promoto your work. not use it just to finish your work. ~~And put in~~ more

Thank you for your time and participation

CamScanner

Participant No (96) & P[illegible]

UNIVERSITI MALAYA

Depart of Media and Communication Studies

AI and Mediated Intercultural Communication Questionnaire

Dear UM Student/ Researcher,

Through this survey, we want to assess the probable effects of Artificial Intelligence (AI) on mediated communication among people from different cultures. This instrument which been developed by the help of the existing literature and ChatGPT, has 15 structured items with five (5) options per item and six (6) open-ended questions. Your participation in the survey is voluntary and the personal identifications of the participants will be treated as strictly confidential.

Thank you for your cooperation.

The researchers

A. Demographic Information

1. Gender: ☑ Male ☐ Female

2. Age: 24

3. Nationality: China

4. Level of education: master

5. AI/ ChatGPT experience: ☑ Yes ☐ No

B. Intercultural Sensitivity Scale

Directions: The current AI-ICC questionnaire includes 15 items/ statements with five (5) options per item, as: 1 – Strongly agree; 2 – Agree, 3 – Uncertain; 4 – Disagree, and 5 – Strongly disagree.	Strongly agree	Agree	Uncertain	Disagree	Strongly disagree
1. AI and AI-powered machines can be helpful in bridging communication gaps between people from different cultures.	1	2 ✓	3	4	5
2. So far, AI has been successful in facilitating cross-cultural understanding and collaboration in mediated communication.	1	2 ✓	3 ✓	4	5
3. AI can help overcome language barriers and promote more inclusive communication among diverse groups.	1	2	3 ✓	4	5

No (36) E, Po

4. There some cultural biases and stereotypes in AI-powered language tools and communication platforms.	1	2 ✓	3	4	5
5. AI has affected the localization and adaptation of content for different cultural audiences in digital communication.	1	2	3 ✓	4	5
6. I encountered any challenges in using AI-powered translation tools when communicating with people from different cultural backgrounds.	1	2 ✓	3	4	5
7. AI has the potential to promote cultural exchange and appreciation by enabling easier access to diverse perspectives and information.	1	2	3 ✓	4	5
8. AI-powered machines and chatbots should be programmed to respect and adapt to cultural norms and communication styles while interacting with users from different cultures.	1 ✓	2	3	4	5
9. There are some potential risks and concerns regarding AI's role in cross-cultural communication, such as perpetuating cultural stereotypes and misinterpretations.	1	2 ✓	3	4	5
10. I have participated in some cross-cultural virtual collaborations that relied on AI for communication and coordination.	1	2	3 ✓	4	5
11. I think AI can enhance intercultural learning experiences and foster empathy among individuals from different cultural backgrounds.	1 ✓	2	3	4	5
12. AI-powered virtual reality (VR) and augmented reality (AR) technologies can impact cross-cultural communication and understanding.	1	2 ✓	3	4	5
13. Somehow AI-generated content may unintentionally offend and misled individuals from specific cultural backgrounds.	1	2	3 ✓	4	5
14. Ethical considerations should be taken into account when designing AI-powered communication tools that cater to diverse cultural contexts.	1 ✓	2	3	4	5
15. Universities and educational institutions leverage AI to create more inclusive and culturally diverse online learning environments.	1	2 ✓	3	4	5

In the case of your agreement on sharing your answer/ points of views for questions below, please write down your comment/ point of view for all questions or any question that you prefer:

1. Did you experience the use of AI and AI-powered chatbots, such as ChatGPT and for what purpose?

   language polish and translation

2. How do you perceive the role of AI and AI-powered machines in bridging communication gaps between people from different cultures?

   AI don't have any role in bridging gaps in my opinion

CS CamScanner

No (98) & P(3)

3. Have you experienced and observed any instances where AI has been successful in facilitating cross-cultural understanding and collaboration in mediated communication?

I don't have any experience ... sorry a.

4. In what ways do you think AI can help overcome language barriers and promote more inclusive communication among diverse groups?

It's really ~~[illegible]~~ help overcome language for people that not very good at anothe ~~[illegible]~~ language and compare to other translation AI more precise.

5. Are there any cultural biases or stereotypes that you have noticed in AI-powered language tools or communication platforms?

In my opinion, no.

6. How can universities and educational institutions leverage AI to create more inclusive and culturally diverse online learning environments?

I think AI dont have role in culture, it is just a tool for efficiay studying.

Thank you for your time and participation

CamScanner

Partpant No (97) E, P(1)

UNIVERSITI MALAYA

Depart of Media and Communication Studies

AI and Mediated Intercultural Communication Questionnaire

Dear UM Student/ Researcher,

Through this survey, we want to assess the probable effects of Artificial Intelligence (AI) on mediated communication among people from different cultures. This instrument which been developed by the help of the existing literature and ChatGPT, has 15 structured items with five (5) options per item and six (6) open-ended questions. Your participation in the survey is voluntary and the personal identifications of the participants will be treated as strictly confidential.

Thank you for your cooperation.

The researchers

A. Demographic Information

1. Gender: ☐ Male ✓ Female

2. Age: 38

3. Nationality: chinese

4. Level of education: master

5. AI/ ChatGPT experience: ✓ Yes ☐ No

B. Intercultural Sensitivity Scale

**Directions:** The current AI-ICC questionnaire includes 15 items/ statements with five (5) options per item, as: 1 – Strongly agree; 2 – Agree, 3 – Uncertain; 4 – Disagree, and 5 – Strongly disagree.	Strongly agree	Agree	Uncertain	Disagree	Strongly [illegible]
1. AI and AI-powered machines can be helpful in bridging communication gaps between people from different cultures.	1	2 ✓	3	4	5
2. So far, AI has been successful in facilitating cross-cultural understanding and collaboration in mediated communication.	1	2	3 ✓	4	5
3. AI can help overcome language barriers and promote more inclusive communication among diverse groups.	1	2 ✓	3	4	5

CS CamScanner

Rto (97) E1 P2

4. There some cultural biases and stereotypes in AI-powered language tools and communication platforms.	1	2	3 ✓	4	5
5. AI has affected the localization and adaptation of content for different cultural audiences in digital communication.	1	2 ✓	3	4	5
6. I encountered any challenges in using AI-powered translation tools when communicating with people from different cultural backgrounds.	1	2	3 ✓	4	5
7. AI has the potential to promote cultural exchange and appreciation by enabling easier access to diverse perspectives and information.	1 ✓	2	3	4	5
8. AI-powered machines and chatbots should be programmed to respect and adapt to cultural norms and communication styles while interacting with users from different cultures.	1 ✓	2	3	4	5
9. There are some potential risks and concerns regarding AI's role in cross-cultural communication, such as perpetuating cultural stereotypes and misinterpretations.	1	2 ✓	3	4	5
10. I have participated in some cross-cultural virtual collaborations that relied on AI for communication and coordination.	1	2	3 ✓	4	5
11. I think AI can enhance intercultural learning experiences and foster empathy among individuals from different cultural backgrounds.	1	2 ✓	3	4	5
12. AI-powered virtual reality (VR) and augmented reality (AR) technologies can impact cross-cultural communication and understanding.	1	2	3 ✓	4	5
13. Somehow AI-generated content may unintentionally offend and misled individuals from specific cultural backgrounds.	1	2	3 ✓	4	5
14. Ethical considerations should be taken into account when designing AI-powered communication tools that cater to diverse cultural contexts.	1	2 ✓	3	4	5
15. Universities and educational institutions leverage AI to create more inclusive and culturally diverse online learning environments.	1	2	3 ✓	4	5

In the case of your agreement on sharing your answer/ points of views for questions below, please write down your comment/ point of view for all questions or any question that you prefer:

1. Did you experience the use of AI and AI-powered chatbots, such as ChatGPT and for what purpose?

It helps in study & education

2. How do you perceive the role of AI and AI-powered machines in bridging communication gaps between people from different cultures?

Help to understand

CS CamScanner

No (97) E, PG

3. Have you experienced and observed any instances where AI has been successful in facilitating cross-cultural understanding and collaboration in mediated communication?

No

4. In what ways do you think AI can help overcome language barriers and promote more inclusive communication among diverse groups?

speech - to speech translation

5. Are there any cultural biases or stereotypes that you have noticed in AI-powered language tools or communication platforms?

No

6. How can universities and educational institutions leverage AI to create more inclusive and culturally diverse online learning environments?

No idea

Thank you for your time and participation

CS CamScanner

Participant No (98) E, P(1)

UNIVERSITI MALAYA

Depart of Media and Communication Studies

AI and Mediated Intercultural Communication Questionnaire

Dear UM Student/ Researcher,

Through this survey, we want to assess the probable effects of Artificial Intelligence (AI) on mediated communication among people from different cultures. This instrument which been developed by the help of the existing literature and ChatGPT, has 15 structured items with five (5) options per item and six (6) open-ended questions. Your participation in the survey is voluntary and the personal identifications of the participants will be treated as strictly confidential.

Thank you for your cooperation.

The researchers

A. Demographic Information

1. Gender: ✓ Male ☐ Female
2. Age: 29
3. Nationality: CHINA
4. Level of education: PHD
5. AI/ ChatGPT experience: ✓Yes ☐ No

B. Intercultural Sensitivity Scale

**Directions:** The current AI-ICC questionnaire includes 15 items/ statements with five (5) options per item, as: 1 – Strongly agree; 2 – Agree, 3 – Uncertain; 4 – Disagree, and 5 – Strongly disagree.	Strongly agree	Agree	Uncertain	Disagree	Strongly disagree
1. AI and AI-powered machines can be helpful in bridging communication gaps between people from different cultures.	1 ✓	2	3	4	5
2. So far, AI has been successful in facilitating cross-cultural understanding and collaboration in mediated communication.	1	2 ✓	3	4	5
3. AI can help overcome language barriers and promote more inclusive communication among diverse groups.	1	2 ✓	3	4	5

CamScanner

No 98 & P2

4. There some cultural biases and stereotypes in AI-powered language tools and communication platforms.	1	2	3 ✓	4	5
5. AI has affected the localization and adaptation of content for different cultural audiences in digital communication.	1	2 ✓	3	4	5
6. I encountered any challenges in using AI-powered translation tools when communicating with people from different cultural backgrounds.	1	2	3 ✓	4	5
7. AI has the potential to promote cultural exchange and appreciation by enabling easier access to diverse perspectives and information.	1 ✓	2	3	4	5
8. AI-powered machines and chatbots should be programmed to respect and adapt to cultural norms and communication styles while interacting with users from different cultures.	1	2 ✓	3	4	5
9. There are some potential risks and concerns regarding AI's role in cross-cultural communication, such as perpetuating cultural stereotypes and misinterpretations.	1	2	3 ✓	4	5
10. I have participated in some cross-cultural virtual collaborations that relied on AI for communication and coordination.	1	2	3	4 ✓	5
11. I think AI can enhance intercultural learning experiences and foster empathy among individuals from different cultural backgrounds.	1 ✓	2	3	4	5
12. AI-powered virtual reality (VR) and augmented reality (AR) technologies can impact cross-cultural communication and understanding.	1 ✓	2	3	4	5
13. Somehow AI-generated content may unintentionally offend and misled individuals from specific cultural backgrounds.	1	2	3 ✓	4	5
14. Ethical considerations should be taken into account when designing AI-powered communication tools that cater to diverse cultural contexts.	1 ✓	2	3	4	5
15. Universities and educational institutions leverage AI to create more inclusive and culturally diverse online learning environments.	1 ✓	2	3	4	5

In the case of your agreement on sharing your answer/ points of views for questions below, please write down your comment/ point of view for all questions or any question that you prefer:

1. Did you experience the use of AI and AI-powered chatbots, such as ChatGPT and for what purpose?

   Used to find information and seek inspiration

2. How do you perceive the role of AI and AI-powered machines in bridging communication gaps between people from different cultures?

   It can give more opportunities to learn about some culture and provide some riche cases.

No (98) & P(3)

3. Have you experienced and observed any instances where AI has been successful in facilitating cross-cultural understanding and collaboration in mediated communication?

Yes!

4. In what ways do you think AI can help overcome language barriers and promote more inclusive communication among diverse groups?

- through some simple text description. or in the form of pictures. or other resources.

5. Are there any cultural biases or stereotypes that you have noticed in AI-powered language tools or communication platforms?

Didn't notice.

6. How can universities and educational institutions leverage AI to create more inclusive and culturally diverse online learning environments?

1. Set up relevant courses
2. Hold conferences.

Thank you for your time and participation

CamScanner

Partcipant No (99) Ep PO

UNIVERSITI MALAYA

Depart of Media and Communication Studies

AI and Mediated Intercultural Communication Questionnaire

Dear UM Student/ Researcher,

Through this survey, we want to assess the probable effects of Artificial Intelligence (AI) on mediated communication among people from different cultures. This instrument which been developed by the help of the existing literature and ChatGPT, has 15 structured items with five (5) options per item and six (6) open-ended questions. Your participation in the survey is voluntary and the personal identifications of the participants will be treated as strictly confidential.

Thank you for your cooperation.

The researchers

A. Demographic Information

1. Gender: ☐ Male ☑ Female

2. Age: 35

3. Nationality: Malaysian

4. Level of education: Postgraduate – MA

5. AI/ ChatGPT experience: ☑ Yes ☐ No

B. Intercultural Sensitivity Scale

**Directions:** The current AI-ICC questionnaire includes 15 items/ statements with five (5) options per item, as: 1 – Strongly agree; 2 – Agree, 3 – Uncertain; 4 – Disagree, and 5 – Strongly disagree.	Strongly agree	Agree	Uncertain	Disagree	Strongly disagree
1. AI and AI-powered machines can be helpful in bridging communication gaps between people from different cultures.	1	(2)	3	4	5
2. So far, AI has been successful in facilitating cross-cultural understanding and collaboration in mediated communication.	1	2	(3)	4	5
3. AI can help overcome language barriers and promote more inclusive communication among diverse groups.	1	(2)	3	4	5

CamScanner

No 99 [illegible]

4. There some cultural biases and stereotypes in AI-powered language tools and communication platforms.	1	2	(3)	4	5
5. AI has affected the localization and adaptation of content for different cultural audiences in digital communication.	1	2	(3)	4	5
6. I encountered any challenges in using AI-powered translation tools when communicating with people from different cultural backgrounds.	1	2	3	(4)	5
7. AI has the potential to promote cultural exchange and appreciation by enabling easier access to diverse perspectives and information.	1	(2)	3	4	5
8. AI-powered machines and chatbots should be programmed to respect and adapt to cultural norms and communication styles while interacting with users from different cultures.	1	(2)	3	4	5
9. There are some potential risks and concerns regarding AI's role in cross-cultural communication, such as perpetuating cultural stereotypes and misinterpretations.	1	(2)	3	4	5
10. I have participated in some cross-cultural virtual collaborations that relied on AI for communication and coordination.	1	2	3	(4)	5
11. I think AI can enhance intercultural learning experiences and foster empathy among individuals from different cultural backgrounds.	1	(2)	3	4	5
12. AI-powered virtual reality (VR) and augmented reality (AR) technologies can impact cross-cultural communication and understanding.	1	(2)	3	4	5
13. Somehow AI-generated content may unintentionally offend and misled individuals from specific cultural backgrounds.	1	(2)	3	4	5
14. Ethical considerations should be taken into account when designing AI-powered communication tools that cater to diverse cultural contexts.	(1)	2	3	4	5
15. Universities and educational institutions leverage AI to create more inclusive and culturally diverse online learning environments.	1	(2)	3	4	5

In the case of your agreement on sharing your answer/ points of views for questions below, please write down your comment/ point of view for all questions or any question that you prefer:

1. Did you experience the use of AI and AI-powered chatbots, such as ChatGPT and for what purpose?

   Yes- Learning, asking questions

2. How do you perceive the role of AI and AI-powered machines in bridging communication gaps between people from different cultures?

   Helpful

CamScanner

No (99) & P(3)

3. Have you experienced and observed any instances where AI has been successful in facilitating cross-cultural understanding and collaboration in mediated communication?

No.

4. In what ways do you think AI can help overcome language barriers and promote more inclusive communication among diverse groups?

Translating apps

5. Are there any cultural biases or stereotypes that you have noticed in AI-powered language tools or communication platforms?

Not sure.

6. How can universities and educational institutions leverage AI to create more inclusive and culturally diverse online learning environments?

Distribute AI software for free to student

Thank you for your time and participation

CS CamScanner

Partipant No (100) & P(1)

UNIVERSITI MALAYA

Depart of Media and Communication Studies

AI and Mediated Intercultural Communication Questionnaire

Dear UM Student/ Researcher,

Through this survey, we want to assess the probable effects of Artificial Intelligence (AI) on mediated communication among people from different cultures. This instrument which been developed by the help of the existing literature and ChatGPT, has 15 structured items with five (5) options per item and six (6) open-ended questions. Your participation in the survey is voluntary and the personal identifications of the participants will be treated as strictly confidential.

Thank you for your cooperation.

The researchers

A. Demographic Information

1. Gender: ☐ Male ☒ Female

2. Age: 28

3. Nationality: Malaysian

4. Level of education: Pst Degree (Post graduation)

5. AI/ ChatGPT experience: ☑ Yes ☒ No

B. Intercultural Sensitivity Scale

**Directions:** The current AI-ICC questionnaire includes 15 items/ statements with five (5) options per item, as: 1 – Strongly agree; 2 – Agree, 3 – Uncertain; 4 – Disagree, and 5 – Strongly disagree.	Strongly agree	Agree	Uncertain	Disagree	Strongly disagree
1. AI and AI-powered machines can be helpful in bridging communication gaps between people from different cultures.	1	2	3	4	(5)
2. So far, AI has been successful in facilitating cross-cultural understanding and collaboration in mediated communication.	1	2	3	4	(5)
3. AI can help overcome language barriers and promote more inclusive communication among diverse groups.	1	2	3	4	(5)

CamScanner

No (100)

4. There some cultural biases and stereotypes in AI-powered language tools and communication platforms.	1	2	3	4	5
5. AI has affected the localization and adaptation of content for different cultural audiences in digital communication.	1	2	3	4	5
6. I encountered any challenges in using AI-powered translation tools when communicating with people from different cultural backgrounds.	1	2	3	4	5
7. AI has the potential to promote cultural exchange and appreciation by enabling easier access to diverse perspectives and information.	1	2	3	4	5
8. AI-powered machines and chatbots should be programmed to respect and adapt to cultural norms and communication styles while interacting with users from different cultures.	1	2	3	4	5
9. There are some potential risks and concerns regarding AI's role in cross-cultural communication, such as perpetuating cultural stereotypes and misinterpretations.	1	2	3	4	5
10. I have participated in some cross-cultural virtual collaborations that relied on AI for communication and coordination.	1	2	3	4	5
11. I think AI can enhance intercultural learning experiences and foster empathy among individuals from different cultural backgrounds.	1	2	3	4	5
12. AI-powered virtual reality (VR) and augmented reality (AR) technologies can impact cross-cultural communication and understanding.	1	2	3	4	5
13. Somehow AI-generated content may unintentionally offend and misled individuals from specific cultural backgrounds.	1	2	3	4	5
14. Ethical considerations should be taken into account when designing AI-powered communication tools that cater to diverse cultural contexts.	1	2	3	4	5
15. Universities and educational institutions leverage AI to create more inclusive and culturally diverse online learning environments.	1	2	3	4	5

In the case of your agreement on sharing your answer/ points of views for questions below, please write down your comment/ point of view for all questions or any question that you prefer:

1. Did you experience the use of AI and AI-powered chatbots, such as ChatGPT and for what purpose?

Never

2. How do you perceive the role of AI and AI-powered machines in bridging communication gaps between people from different cultures?

Redundant

CamScanner

No (100) [illegible] P(3)

3. Have you experienced and observed any instances where AI has been successful in facilitating cross-cultural understanding and collaboration in mediated communication?

No. We had been doing fine with messaging b/w human to human

4. In what ways do you think AI can help overcome language barriers and promote more inclusive communication among diverse groups?

Just another dictionary, translator

5. Are there any cultural biases or stereotypes that you have noticed in AI-powered language tools or communication platforms?

depends on the creator, mostly westernised ideologies.

6. How can universities and educational institutions leverage AI to create more inclusive and culturally diverse online learning environments?

not sure, but should be limited

Thank you for your time and participation

CamScanner

Participant No (101) & P(1)

UNIVERSITI MALAYA

Depart of Media and Communication Studies

AI and Mediated Intercultural Communication Questionnaire

Dear UM Student/ Researcher,

Through this survey, we want to assess the probable effects of Artificial Intelligence (AI) on mediated communication among people from different cultures. This instrument which been developed by the help of the existing literature and ChatGPT, has 15 structured items with five (5) options per item and six (6) open-ended questions. Your participation in the survey is voluntary and the personal identifications of the participants will be treated as strictly confidential.

Thank you for your cooperation.

The researchers

A. Demographic Information

1. Gender: ☐ Male ☑ Female

2. Age: 30

3. Nationality: Malaysian

4. Level of education: PhD

5. AI/ ChatGPT experience: ☑ Yes ☐ No

B. Intercultural Sensitivity Scale

Directions: The current AI-ICC questionnaire includes 15 items/ statements with five (5) options per item, as: 1 – Strongly agree; 2 – Agree, 3 – Uncertain; 4 – Disagree, and 5 – Strongly disagree.	Strongly agree	Agree	Uncertain	Disagree	Strongly disagree
1. AI and AI-powered machines can be helpful in bridging communication gaps between people from different cultures.	1	(2)	3	4	5
2. So far, AI has been successful in facilitating cross-cultural understanding and collaboration in mediated communication.	1	(2)	3	4	5
3. AI can help overcome language barriers and promote more inclusive communication among diverse groups.	1	(2)	3	4	5

CamScanner

No (101) & P2

4. There some cultural biases and stereotypes in AI-powered language tools and communication platforms.	1	(2)	3	4	5
5. AI has affected the localization and adaptation of content for different cultural audiences in digital communication.	1	(2)	3	4	5
6. I encountered any challenges in using AI-powered translation tools when communicating with people from different cultural backgrounds.	1	2	(3)	4	5
7. AI has the potential to promote cultural exchange and appreciation by enabling easier access to diverse perspectives and information.	1	(2)	3	4	5
8. AI-powered machines and chatbots should be programmed to respect and adapt to cultural norms and communication styles while interacting with users from different cultures.	1	(2)	3	4	5
9. There are some potential risks and concerns regarding AI's role in cross-cultural communication, such as perpetuating cultural stereotypes and misinterpretations.	(1)	2	3	4	5
10. I have participated in some cross-cultural virtual collaborations that relied on AI for communication and coordination.	1	2	(3)	4	5
11. I think AI can enhance intercultural learning experiences and foster empathy among individuals from different cultural backgrounds.	1	(2)	3	4	5
12. AI-powered virtual reality (VR) and augmented reality (AR) technologies can impact cross-cultural communication and understanding.	1	(2)	3	4	5
13. Somehow AI-generated content may unintentionally offend and misled individuals from specific cultural backgrounds.	1	(2)	3	4	5
14. Ethical considerations should be taken into account when designing AI-powered communication tools that cater to diverse cultural contexts.	(1)	2	3	4	5
15. Universities and educational institutions leverage AI to create more inclusive and culturally diverse online learning environments.	1	(2)	3	4	5

In the case of your agreement on sharing your answer/ points of views for questions below, please write down your comment/ point of view for all questions or any question that you prefer:

1. Did you experience the use of AI and AI-powered chatbots, such as ChatGPT and for what purpose?

   Yes, for making sentence

2. How do you perceive the role of AI and AI-powered machines in bridging communication gaps between people from different cultures?

   Great

CamScanner

No (101) E, P3

3. Have you experienced and observed any instances where AI has been successful in facilitating cross-cultural understanding and collaboration in mediated communication?

Not yet

4. In what ways do you think AI can help overcome language barriers and promote more inclusive communication among diverse groups?

By bridging the understanding

5. Are there any cultural biases or stereotypes that you have noticed in AI-powered language tools or communication platforms?

lack of humane

6. How can universities and educational institutions leverage AI to create more inclusive and culturally diverse online learning environments?

By creating specific rules on how to impement A I

Thank you for your time and participation

CS CamScanner

Partecepant No (102) E, P(1)

UNIVERSITI MALAYA

Depart of Media and Communication Studies

AI and Mediated Intercultural Communication Questionnaire

Dear UM Student/ Researcher,

Through this survey, we want to assess the probable effects of Artificial Intelligence (AI) on mediated communication among people from different cultures. This instrument which been developed by the help of the existing literature and ChatGPT, has 15 structured items with five (5) options per item and six (6) open-ended questions. Your participation in the survey is voluntary and the personal identifications of the participants will be treated as strictly confidential.

Thank you for your cooperation.

The researchers

A. Demographic Information

1. Gender: ☐ Male ☒ Female

2. Age: 28

3. Nationality: Malaysia

4. Level of education: Phd

5. AI/ ChatGPT experience: ☐ Yes ☒ No

B. Intercultural Sensitivity Scale

Directions: The current AI-ICC questionnaire includes 15 items/ statements with five (5) options per item, as: 1 – Strongly agree; 2 – Agree, 3 – Uncertain; 4 – Disagree, and 5 – Strongly disagree.	Strongly agree	Agree	Uncertain	Disagree	Strongly disagree
1. AI and AI-powered machines can be helpful in bridging communication gaps between people from different cultures.	(1)	2	3	4	5
2. So far, AI has been successful in facilitating cross-cultural understanding and collaboration in mediated communication.	1	2	(3)	4	5
3. AI can help overcome language barriers and promote more inclusive communication among diverse groups.	1	(2)	3	4	5

CamScanner

No 102 Ee Pe

4. There some cultural biases and stereotypes in AI-powered language tools and communication platforms.	1	**2**	3	4	5
5. AI has affected the localization and adaptation of content for different cultural audiences in digital communication.	**1**	2	3	4	5
6. I encountered any challenges in using AI-powered translation tools when communicating with people from different cultural backgrounds.	1	2	**3**	4	5
7. AI has the potential to promote cultural exchange and appreciation by enabling easier access to diverse perspectives and information.	**1**	2	3	4	5
8. AI-powered machines and chatbots should be programmed to respect and adapt to cultural norms and communication styles while interacting with users from different cultures.	**1**	2	3	4	5
9. There are some potential risks and concerns regarding AI's role in cross-cultural communication, such as perpetuating cultural stereotypes and misinterpretations.	1	**2**	3	4	5
10. I have participated in some cross-cultural virtual collaborations that relied on AI for communication and coordination.	1	2	3	4	**5**
11. I think AI can enhance intercultural learning experiences and foster empathy among individuals from different cultural backgrounds.	1	2	3	**4**	5
12. AI-powered virtual reality (VR) and augmented reality (AR) technologies can impact cross-cultural communication and understanding.	1	**2**	3	4	5
13. Somehow AI-generated content may unintentionally offend and misled individuals from specific cultural backgrounds.	1	**2**	3	4	5
14. Ethical considerations should be taken into account when designing AI-powered communication tools that cater to diverse cultural contexts.	**1**	2	3	4	5
15. Universities and educational institutions leverage AI to create more inclusive and culturally diverse online learning environments.	**1**	2	3	4	5

In the case of your agreement on sharing your answer/ points of views for questions below, please write down your comment/ point of view for all questions or any question that you prefer:

1. Did you experience the use of AI and AI-powered chatbots, such as ChatGPT and for what purpose?

Yes for communication ~~[illegible]~~

2. How do you perceive the role of AI and AI-powered machines in bridging communication gaps between people from different cultures?

Different cultures have their own identity. AI-powered machines can learn to adapt with their own language / identity. or can have many different choices of communication that suitable for that cultures.

CamScanner

No (102) E_C P3

3. Have you experienced and observed any instances where AI has been successful in facilitating cross-cultural understanding and collaboration in mediated communication?

NO.

4. In what ways do you think AI can help overcome language barriers and promote more inclusive communication among diverse groups?

Language translators /

5. Are there any cultural biases or stereotypes that you have noticed in AI-powered language tools or communication platforms?

Cannot direct translate / have difficult translation for some accents / have to download the application.

6. How can universities and educational institutions leverage AI to create more inclusive and culturally diverse online learning environments?

- Students from different cultures take parts in creating of the AI.
- Not limits for only most use language / most interesting language.

Thank you for your time and participation

CS CamScanner

Partcipant No (103) Ec P(1)

UNIVERSITI MALAYA

Depart of Media and Communication Studies

AI and Mediated Intercultural Communication Questionnaire

Dear UM Student/ Researcher,

Through this survey, we want to assess the probable effects of Artificial Intelligence (AI) on mediated communication among people from different cultures. This instrument which been developed by the help of the existing literature and ChatGPT, has 15 structured items with five (5) options per item and six (6) open-ended questions. Your participation in the survey is voluntary and the personal identifications of the participants will be treated as strictly confidential.

Thank you for your cooperation.

The researchers

A. Demographic Information

1. Gender: ☑ Male ☐ Female

2. Age: 38

3. Nationality: Nigerian (Nigeria)

4. Level of education: Master

5. AI/ ChatGPT experience: ☑ Yes ☐ No

B. Intercultural Sensitivity Scale

Directions: The current AI-ICC questionnaire includes 15 items/ statements with five (5) options per item, as: 1 – Strongly agree; 2 – Agree, 3 – Uncertain; 4 – Disagree, and 5 – Strongly disagree.	Strongly agree	Agree	Uncertain	Disagree	Strongly disagree
1. AI and AI-powered machines can be helpful in bridging communication gaps between people from different cultures.	(1)	2	3	4	5
2. So far, AI has been successful in facilitating cross-cultural understanding and collaboration in mediated communication.	(1)	2	3	4	5
3. AI can help overcome language barriers and promote more inclusive communication among diverse groups.	(1)	2	3	4	5

CS CamScanner

Pa- No (103) Ec P2

4. There some cultural biases and stereotypes in AI-powered language tools and communication platforms.	1	2	3	4	5
5. AI has affected the localization and adaptation of content for different cultural audiences in digital communication.	1	2	3	4	5
6. I encountered any challenges in using AI-powered translation tools when communicating with people from different cultural backgrounds.	1	2	3	4	5
7. AI has the potential to promote cultural exchange and appreciation by enabling easier access to diverse perspectives and information.	1	2	3	4	5
8. AI-powered machines and chatbots should be programmed to respect and adapt to cultural norms and communication styles while interacting with users from different cultures.	1	2	3	4	5
9. There are some potential risks and concerns regarding AI's role in cross-cultural communication, such as perpetuating cultural stereotypes and misinterpretations.	1	2	3	4	5
10. I have participated in some cross-cultural virtual collaborations that relied on AI for communication and coordination.	1	2	3	4	5
11. I think AI can enhance intercultural learning experiences and foster empathy among individuals from different cultural backgrounds.	1	2	3	4	5
12. AI-powered virtual reality (VR) and augmented reality (AR) technologies can impact cross-cultural communication and understanding.	1	2	3	4	5
13. Somehow AI-generated content may unintentionally offend and misled individuals from specific cultural backgrounds.	1	2	3	4	5
14. Ethical considerations should be taken into account when designing AI-powered communication tools that cater to diverse cultural contexts.	1	2	3	4	5
15. Universities and educational institutions leverage AI to create more inclusive and culturally diverse online learning environments.	1	2	3	4	5

In the case of your agreement on sharing your answer/ points of views for questions below, please write down your comment/ point of view for all questions or any question that you prefer:

1. Did you experience the use of AI and AI-powered chatbots, such as ChatGPT and for what purpose?

for Research and proposal writing

2. How do you perceive the role of AI and AI-powered machines in bridging communication gaps between people from different cultures?

It helps to bring cultures of other regions closer.

CamScanner

No (103) E PC3

3. Have you experienced and observed any instances where AI has been successful in facilitating cross-cultural understanding and collaboration in mediated communication?

No.

4. In what ways do you think AI can help overcome language barriers and promote more inclusive communication among diverse groups?

More research should be carried out.

5. Are there any cultural biases or stereotypes that you have noticed in AI-powered language tools or communication platforms?

Yes - for issue of LGBT.

6. How can universities and educational institutions leverage AI to create more inclusive and culturally diverse online learning environments?

More research should be written on AI.

Thank you for your time and participation

CS CamScanner

Participant No (104) & PD

UNIVERSITI MALAYA

Depart of Media and Communication Studies

AI and Mediated Intercultural Communication Questionnaire

Dear UM Student/ Researcher,

Through this survey, we want to assess the probable effects of Artificial Intelligence (AI) on mediated communication among people from different cultures. This instrument which been developed by the help of the existing literature and ChatGPT, has 15 structured items with five (5) options per item and six (6) open-ended questions. Your participation in the survey is voluntary and the personal identifications of the participants will be treated as strictly confidential.

Thank you for your cooperation.

The researchers

A. Demographic Information

1. Gender: ☑ Male ☐ Female

2. Age: 32

3. Nationality: Malaysia

4. Level of education: Post Bachelor Degree (Post-Graduation)

5. AI/ ChatGPT experience: ☑ Yes ☐ No

B. Intercultural Sensitivity Scale

Directions: The current AI-ICC questionnaire includes 15 items/ statements with five (5) options per item, as: 1 – Strongly agree; 2 – Agree, 3 – Uncertain; 4 – Disagree, and 5 – Strongly disagree.	Strongly agree	Agree	Uncertain	Disagree	Strongly disagree
1. AI and AI-powered machines can be helpful in bridging communication gaps between people from different cultures.	1	(2)	3	4	5
2. So far, AI has been successful in facilitating cross-cultural understanding and collaboration in mediated communication.	1	(2)	3	4	5
3. AI can help overcome language barriers and promote more inclusive communication among diverse groups.	1	(2)	3	4	5

CamScanner

No 104 & 10

4. There some cultural biases and stereotypes in AI-powered language tools and communication platforms.	1	2	3	4	5
5. AI has affected the localization and adaptation of content for different cultural audiences in digital communication.	1	2	3	4	5
6. I encountered any challenges in using AI-powered translation tools when communicating with people from different cultural backgrounds.	1	2	3	4	5
7. AI has the potential to promote cultural exchange and appreciation by enabling easier access to diverse perspectives and information.	1	2	3	4	5
8. AI-powered machines and chatbots should be programmed to respect and adapt to cultural norms and communication styles while interacting with users from different cultures.	1	2	3	4	5
9. There are some potential risks and concerns regarding AI's role in cross-cultural communication, such as perpetuating cultural stereotypes and misinterpretations.	1	2	3	4	5
10. I have participated in some cross-cultural virtual collaborations that relied on AI for communication and coordination.	1	2	3	4	5
11. I think AI can enhance intercultural learning experiences and foster empathy among individuals from different cultural backgrounds.	1	2	3	4	5
12. AI-powered virtual reality (VR) and augmented reality (AR) technologies can impact cross-cultural communication and understanding.	1	2	3	4	5
13. Somehow AI-generated content may unintentionally offend and misled individuals from specific cultural backgrounds.	1	2	3	4	5
14. Ethical considerations should be taken into account when designing AI-powered communication tools that cater to diverse cultural contexts.	1	2	3	4	5
15. Universities and educational institutions leverage AI to create more inclusive and culturally diverse online learning environments.	1	2	3	4	5

In the case of your agreement on sharing your answer/ points of views for questions below, please write down your comment/ point of view for all questions or any question that you prefer:

1. Did you experience the use of AI and AI-powered chatbots, such as ChatGPT and for what purpose?

   for Study

2. How do you perceive the role of AI and AI-powered machines in bridging communication gaps between people from different cultures?

   very good

CamScanner

No (104) & P(3)

3. Have you experienced and observed any instances where AI has been successful in facilitating cross-cultural understanding and collaboration in mediated communication?

no

4. In what ways do you think AI can help overcome language barriers and promote more inclusive communication among diverse groups?

I am not sure

5. Are there any cultural biases or stereotypes that you have noticed in AI-powered language tools or communication platforms?

no

6. How can universities and educational institutions leverage AI to create more inclusive and culturally diverse online learning environments?

I am not sure.

Thank you for your time and participation

CS CamScanner

Participant No 105 & RO

UNIVERSITI MALAYA

Depart of Media and Communication Studies

AI and Mediated Intercultural Communication Questionnaire

Dear UM Student/ Researcher,

Through this survey, we want to assess the probable effects of Artificial Intelligence (AI) on mediated communication among people from different cultures. This instrument which been developed by the help of the existing literature and ChatGPT, has 15 structured items with five (5) options per item and six (6) open-ended questions. Your participation in the survey is voluntary and the personal identifications of the participants will be treated as strictly confidential.

Thank you for your cooperation.

The researchers

A. Demographic Information

1. Gender: ☑ Male ☐ Female
2. Age: 29
3. Nationality: Chinese
4. Level of education: PhD
5. AI/ ChatGPT experience: ☑ Yes ☐ No

B. Intercultural Sensitivity Scale

**Directions:** The current AI-ICC questionnaire includes 15 items/ statements with five (5) options per item, as: 1 – Strongly agree; 2 – Agree, 3 – Uncertain; 4 – Disagree, and 5 – Strongly disagree.	Strongly agree	Agree	Uncertain	Disagree	Strongly disagree
1. AI and AI-powered machines can be helpful in bridging communication gaps between people from different cultures.	1	2	3 ✓	4	5
2. So far, AI has been successful in facilitating cross-cultural understanding and collaboration in mediated communication.	1	2	3 ✓	4	5
3. AI can help overcome language barriers and promote more inclusive communication among diverse groups.	1	2	3 ✓	4	5

CamScanner

No (105) & P(2)

4. There some cultural biases and stereotypes in AI-powered language tools and communication platforms.	1	2	3 ✓	4	5
5. AI has affected the localization and adaptation of content for different cultural audiences in digital communication.	1	2	3 ✓	4	5
6. I encountered any challenges in using AI-powered translation tools when communicating with people from different cultural backgrounds.	1	2	3	4 ✓	5
7. AI has the potential to promote cultural exchange and appreciation by enabling easier access to diverse perspectives and information.	1	2	3 ✓	4	5
8. AI-powered machines and chatbots should be programmed to respect and adapt to cultural norms and communication styles while interacting with users from different cultures.	1	2	3 ✓	4	5
9. There are some potential risks and concerns regarding AI's role in cross-cultural communication, such as perpetuating cultural stereotypes and misinterpretations.	1	2	3 ✓	4	5
10. I have participated in some cross-cultural virtual collaborations that relied on AI for communication and coordination.	1	2	3 ✓	4	5
11. I think AI can enhance intercultural learning experiences and foster empathy among individuals from different cultural backgrounds.	1	2	3 ✓	4	5
12. AI-powered virtual reality (VR) and augmented reality (AR) technologies can impact cross-cultural communication and understanding.	1	2	3 ✓	4	5
13. Somehow AI-generated content may unintentionally offend and misled individuals from specific cultural backgrounds.	1	2	3	4 ✓	5
14. Ethical considerations should be taken into account when designing AI-powered communication tools that cater to diverse cultural contexts.	1	2	3	4 ✓	5
15. Universities and educational institutions leverage AI to create more inclusive and culturally diverse online learning environments.	1	2	3 ✓	4	5

In the case of your agreement on sharing your answer/ points of views for questions below, please write down your comment/ point of view for all questions or any question that you prefer:

1. Did you experience the use of AI and AI-powered chatbots, such as ChatGPT and for what purpose?

Yes

2. How do you perceive the role of AI and AI-powered machines in bridging communication gaps between people from different cultures?

havn't consider

CamScanner

No (105) & P(3)

3. Have you experienced and observed any instances where AI has been successful in facilitating cross-cultural understanding and collaboration in mediated communication?

no

4. In what ways do you think AI can help overcome language barriers and promote more inclusive communication among diverse groups?

via Chat GPT

5. Are there any cultural biases or stereotypes that you have noticed in AI-powered language tools or communication platforms?

no

6. How can universities and educational institutions leverage AI to create more inclusive and culturally diverse online learning environments?

digital tutor

Thank you for your time and participation

CS CamScanner

Participant No 106 & P①

UNIVERSITI MALAYA

Depart of Media and Communication Studies

AI and Mediated Intercultural Communication Questionnaire

Dear UM Student/ Researcher,

Through this survey, we want to assess the probable effects of Artificial Intelligence (AI) on mediated communication among people from different cultures. This instrument which been developed by the help of the existing literature and ChatGPT, has 15 structured items with five (5) options per item and six (6) open-ended questions. Your participation in the survey is voluntary and the personal identifications of the participants will be treated as strictly confidential.

Thank you for your cooperation.

The researchers

A. Demographic Information

1. Gender: ☐ Male ☑ Female

2. Age: 32

3. Nationality: Malaysia

4. Level of education: PhD

5. AI/ ChatGPT experience: ☑ Yes ☐ No

B. Intercultural Sensitivity Scale

**Directions:** The current AI-ICC questionnaire includes 15 items/ statements with five (5) options per item, as: 1 – Strongly agree; 2 – Agree, 3 – Uncertain; 4 – Disagree, and 5 – Strongly disagree.	Strongly agree	Agree	Uncertain	Disagree	Strongly disagree
1. AI and AI-powered machines can be helpful in bridging communication gaps between people from different cultures.	1	2 ✓	3	4	5
2. So far, AI has been successful in facilitating cross-cultural understanding and collaboration in mediated communication.	1	2	3 ✓	4	5
3. AI can help overcome language barriers and promote more inclusive communication among diverse groups.	1	2 ✓	3	4	5

CamScanner

No (106) & P2

4. There some cultural biases and stereotypes in AI-powered language tools and communication platforms.	1	2	3	4	5
5. AI has affected the localization and adaptation of content for different cultural audiences in digital communication.	1	2	3	4	5
6. I encountered any challenges in using AI-powered translation tools when communicating with people from different cultural backgrounds.	1	2	3	4	5
7. AI has the potential to promote cultural exchange and appreciation by enabling easier access to diverse perspectives and information.	1	2	3	4	5
8. AI-powered machines and chatbots should be programmed to respect and adapt to cultural norms and communication styles while interacting with users from different cultures.	1	2	3	4	5
9. There are some potential risks and concerns regarding AI's role in cross-cultural communication, such as perpetuating cultural stereotypes and misinterpretations.	1	2	3	4	5
10. I have participated in some cross-cultural virtual collaborations that relied on AI for communication and coordination.	1	2	3	4	5
11. I think AI can enhance intercultural learning experiences and foster empathy among individuals from different cultural backgrounds.	1	2	3	4	5
12. AI-powered virtual reality (VR) and augmented reality (AR) technologies can impact cross-cultural communication and understanding.	1	2	3	4	5
13. Somehow AI-generated content may unintentionally offend and misled individuals from specific cultural backgrounds.	1	2	3	4	5
14. Ethical considerations should be taken into account when designing AI-powered communication tools that cater to diverse cultural contexts.	1	2	3	4	5
15. Universities and educational institutions leverage AI to create more inclusive and culturally diverse online learning environments.	1	2	3	4	5

In the case of your agreement on sharing your answer/ points of views for questions below, please write down your comment/ point of view for all questions or any question that you prefer:

1. Did you experience the use of AI and AI-powered chatbots, such as ChatGPT and for what purpose?

No

2. How do you perceive the role of AI and AI-powered machines in bridging communication gaps between people from different cultures?

CamScanner

No (106) E PB

3. Have you experienced and observed any instances where AI has been successful in facilitating cross-cultural understanding and collaboration in mediated communication?

4. In what ways do you think AI can help overcome language barriers and promote more inclusive communication among diverse groups?

5. Are there any cultural biases or stereotypes that you have noticed in AI-powered language tools or communication platforms?

6. How can universities and educational institutions leverage AI to create more inclusive and culturally diverse online learning environments?

Thank you for your time and participation

CS CamScanner

Participant No (107) E P(1)

UNIVERSITI MALAYA

Depart of Media and Communication Studies

AI and Mediated Intercultural Communication Questionnaire

Dear UM Student/ Researcher,

Through this survey, we want to assess the probable effects of Artificial Intelligence (AI) on mediated communication among people from different cultures. This instrument which been developed by the help of the existing literature and ChatGPT, has 15 structured items with five (5) options per item and six (6) open-ended questions. Your participation in the survey is voluntary and the personal identifications of the participants will be treated as strictly confidential.

Thank you for your cooperation.

The researchers

A. Demographic Information

1. Gender: ☑ Male ☐ Female

2. Age: 29

3. Nationality: Palestinian

4. Level of education: PhD

5. AI/ ChatGPT experience: ☐ Yes ☑ No

B. Intercultural Sensitivity Scale

**Directions:** The current AI-ICC questionnaire includes 15 items/ statements with five (5) options per item, as: 1 – Strongly agree; 2 – Agree, 3 – Uncertain; 4 – Disagree, and 5 – Strongly disagree.	Strongly agree	Agree	Uncertain	Disagree	Strongly disagree
1. AI and AI-powered machines can be helpful in bridging communication gaps between people from different cultures.	1	2 ✓	3	4	5
2. So far, AI has been successful in facilitating cross-cultural understanding and collaboration in mediated communication.	1	2 ✓	3	4	5
3. AI can help overcome language barriers and promote more inclusive communication among diverse groups.	1	2 ✓	3	4	5

CamScanner

Participants 107 & [illegible]

4. There some cultural biases and stereotypes in AI-powered language tools and communication platforms.	1	2	3 ✓	4	5
5. AI has affected the localization and adaptation of content for different cultural audiences in digital communication.	1	2 ✓	3	4	5
6. I encountered any challenges in using AI-powered translation tools when communicating with people from different cultural backgrounds.	1	2	3 ✓	4	5
7. AI has the potential to promote cultural exchange and appreciation by enabling easier access to diverse perspectives and information.	1	2 ✓	3	4	5
8. AI-powered machines and chatbots should be programmed to respect and adapt to cultural norms and communication styles while interacting with users from different cultures.	1	2 ✓	3	4	5
9. There are some potential risks and concerns regarding AI's role in cross-cultural communication, such as perpetuating cultural stereotypes and misinterpretations.	1	2 ✓	3	4	5
10. I have participated in some cross-cultural virtual collaborations that relied on AI for communication and coordination.	1	2	3 ✓	4	5
11. I think AI can enhance intercultural learning experiences and foster empathy among individuals from different cultural backgrounds.	1	2	3 ✓	4	5
12. AI-powered virtual reality (VR) and augmented reality (AR) technologies can impact cross-cultural communication and understanding.	1	2	3 ✓	4	5
13. Somehow AI-generated content may unintentionally offend and misled individuals from specific cultural backgrounds.	1	2	3 ✓	4	5
14. Ethical considerations should be taken into account when designing AI-powered communication tools that cater to diverse cultural contexts.	1	2 ✓	3	4	5
15. Universities and educational institutions leverage AI to create more inclusive and culturally diverse online learning environments.	1	2 ✓	3	4	5

In the case of your agreement on sharing your answer/ points of views for questions below, please write down your comment/ point of view for all questions or any question that you prefer:

1. Did you experience the use of AI and AI-powered chatbots, such as ChatGPT and for what purpose?

2. How do you perceive the role of AI and AI-powered machines in bridging communication gaps between people from different cultures?

CamScanner

No (107) & P3

3. Have you experienced and observed any instances where AI has been successful in facilitating cross-cultural understanding and collaboration in mediated communication?

4. In what ways do you think AI can help overcome language barriers and promote more inclusive communication among diverse groups?

5. Are there any cultural biases or stereotypes that you have noticed in AI-powered language tools or communication platforms?

6. How can universities and educational institutions leverage AI to create more inclusive and culturally diverse online learning environments?

Thank you for your time and participation

CamScanner

Participant No (108) - P(1)

UNIVERSITI MALAYA

Depart of Media and Communication Studies

AI and Mediated Intercultural Communication Questionnaire

Dear UM Student/ Researcher,

Through this survey, we want to assess the probable effects of Artificial Intelligence (AI) on mediated communication among people from different cultures. This instrument which been developed by the help of the existing literature and ChatGPT, has 15 structured items with five (5) options per item and six (6) open-ended questions. Your participation in the survey is voluntary and the personal identifications of the participants will be treated as strictly confidential.

Thank you for your cooperation.

The researchers

A. Demographic Information

1. Gender: ☐ Male ☒ Female

2. Age: 35

3. Nationality: Malaysian

4. Level of education: Bachelor Degree

5. AI/ ChatGPT experience: ☒ Yes ☐ No

B. Intercultural Sensitivity Scale

**Directions:** The current AI-ICC questionnaire includes 15 items/ statements with five (5) options per item, as: 1 – Strongly agree; 2 – Agree, 3 – Uncertain; 4 – Disagree, and 5 – Strongly disagree.	Strongly agree	Agree	Uncertain	Disagree	Strongly disagree
1. AI and AI-powered machines can be helpful in bridging communication gaps between people from different cultures.	1	2 ✓	3	4	5
2. So far, AI has been successful in facilitating cross-cultural understanding and collaboration in mediated communication.	1	2 ✓	3	4	5
3. AI can help overcome language barriers and promote more inclusive communication among diverse groups.	1	2	3 ✓	4	5

CamScanner

No (108) & Pr

4. There some cultural biases and stereotypes in AI-powered language tools and communication platforms.	1	2	3 ✓	4	5
5. AI has affected the localization and adaptation of content for different cultural audiences in digital communication.	1	2	3 ✓	4	5
6. I encountered any challenges in using AI-powered translation tools when communicating with people from different cultural backgrounds.	1	2	3 ✓	4	5
7. AI has the potential to promote cultural exchange and appreciation by enabling easier access to diverse perspectives and information.	1	2	3 ✓	4	5
8. AI-powered machines and chatbots should be programmed to respect and adapt to cultural norms and communication styles while interacting with users from different cultures.	1	2	3 ✓	4	5
9. There are some potential risks and concerns regarding AI's role in cross-cultural communication, such as perpetuating cultural stereotypes and misinterpretations.	1	2	3 ✓	4	5
10. I have participated in some cross-cultural virtual collaborations that relied on AI for communication and coordination.	1	2	3 ✓	4	5
11. I think AI can enhance intercultural learning experiences and foster empathy among individuals from different cultural backgrounds.	1	2	3 ✓	4	5
12. AI-powered virtual reality (VR) and augmented reality (AR) technologies can impact cross-cultural communication and understanding.	1	2 ✓	3	4	5
13. Somehow AI-generated content may unintentionally offend and misled individuals from specific cultural backgrounds.	1	2	3 ✓	4	5
14. Ethical considerations should be taken into account when designing AI-powered communication tools that cater to diverse cultural contexts.	1	2	3 ✓	4	5
15. Universities and educational institutions leverage AI to create more inclusive and culturally diverse online learning environments.	1	2	3 ✓	4	5

In the case of your agreement on sharing your answer/ points of views for questions below, please write down your comment/ point of view for all questions or any question that you prefer:

1. Did you experience the use of AI and AI-powered chatbots, such as ChatGPT and for what purpose?

   for travelling purposes. (holiday)

2. How do you perceive the role of AI and AI-powered machines in bridging communication gaps between people from different cultures?

   –

CamScanner

No (08) & P3

3. Have you experienced and observed any instances where AI has been successful in facilitating cross-cultural understanding and collaboration in mediated communication?

—

4. In what ways do you think AI can help overcome language barriers and promote more inclusive communication among diverse groups?

5. Are there any cultural biases or stereotypes that you have noticed in AI-powered language tools or communication platforms?

—

6. How can universities and educational institutions leverage AI to create more inclusive and culturally diverse online learning environments?

—

Thank you for your time and participation

CamScanner

Participant No (109) & P(1)

UNIVERSITI MALAYA

Depart of Media and Communication Studies

AI and Mediated Intercultural Communication Questionnaire

Dear UM Student/ Researcher,

Through this survey, we want to assess the probable effects of Artificial Intelligence (AI) on mediated communication among people from different cultures. This instrument which been developed by the help of the existing literature and ChatGPT, has 15 structured items with five (5) options per item and six (6) open-ended questions. Your participation in the survey is voluntary and the personal identifications of the participants will be treated as strictly confidential.

Thank you for your cooperation.

The researchers

A. Demographic Information

1. Gender: ☑ Male ☐ Female

2. Age: 37

3. Nationality: Indonesia

4. Level of education: Ph.D

5. AI/ ChatGPT experience: ☑ Yes ☐ No

B. Intercultural Sensitivity Scale

Directions: The current AI-ICC questionnaire includes 15 items/ statements with five (5) options per item, as: 1 – Strongly agree; 2 – Agree, 3 – Uncertain; 4 – Disagree, and 5 – Strongly disagree.	Strongly agree	Agree	Uncertain	Disagree	Strongly disagree
1. AI and AI-powered machines can be helpful in bridging communication gaps between people from different cultures.	(1)	2	3	4	5
2. So far, AI has been successful in facilitating cross-cultural understanding and collaboration in mediated communication.	1	(2)	3	4	5
3. AI can help overcome language barriers and promote more inclusive communication among diverse groups.	1	(2)	3	4	5

CS CamScanner

Participant No (103) [illegible] P2

4. There some cultural biases and stereotypes in AI-powered language tools and communication platforms.	1	2	(3)	4	5
5. AI has affected the localization and adaptation of content for different cultural audiences in digital communication.	1	2	(3)	4	5
6. I encountered any challenges in using AI-powered translation tools when communicating with people from different cultural backgrounds.	1	(2)	3	4	5
7. AI has the potential to promote cultural exchange and appreciation by enabling easier access to diverse perspectives and information.	1	(2)	3	4	5
8. AI-powered machines and chatbots should be programmed to respect and adapt to cultural norms and communication styles while interacting with users from different cultures.	(1)	2	3	4	5
9. There are some potential risks and concerns regarding AI's role in cross-cultural communication, such as perpetuating cultural stereotypes and misinterpretations.	(1)	2	3	4	5
10. I have participated in some cross-cultural virtual collaborations that relied on AI for communication and coordination.	1	2	(3)	4	5
11. I think AI can enhance intercultural learning experiences and foster empathy among individuals from different cultural backgrounds.	1	(2)	3	4	5
12. AI-powered virtual reality (VR) and augmented reality (AR) technologies can impact cross-cultural communication and understanding.	1	(2)	3	4	5
13. Somehow AI-generated content may unintentionally offend and misled individuals from specific cultural backgrounds.	1	(2)	3	4	5
14. Ethical considerations should be taken into account when designing AI-powered communication tools that cater to diverse cultural contexts.	(1)	2	3	4	5
15. Universities and educational institutions leverage AI to create more inclusive and culturally diverse online learning environments.	(1)	2	3	4	5

In the case of your agreement on sharing your answer/ points of views for questions below, please write down your comment/ point of view for all questions or any question that you prefer:

1. Did you experience the use of AI and AI-powered chatbots, such as ChatGPT and for what purpose?

   yes, only for academic purposes.

2. How do you perceive the role of AI and AI-powered machines in bridging communication gaps between people from different cultures?

CamScanner

No (109) Ei B

3. Have you experienced and observed any instances where AI has been successful in facilitating cross-cultural understanding and collaboration in mediated communication?

4. In what ways do you think AI can help overcome language barriers and promote more inclusive communication among diverse groups?

5. Are there any cultural biases or stereotypes that you have noticed in AI-powered language tools or communication platforms?

6. How can universities and educational institutions leverage AI to create more inclusive and culturally diverse online learning environments?

Thank you for your time and participation

CS CamScanner

Participant No (110) & P(1)

UNIVERSITI MALAYA

Depart of Media and Communication Studies

AI and Mediated Intercultural Communication Questionnaire

Dear UM Student/ Researcher,

Through this survey, we want to assess the probable effects of Artificial Intelligence (AI) on mediated communication among people from different cultures. This instrument which been developed by the help of the existing literature and ChatGPT, has 15 structured items with five (5) options per item and six (6) open-ended questions. Your participation in the survey is voluntary and the personal identifications of the participants will be treated as strictly confidential.

Thank you for your cooperation.

The researchers

A. Demographic Information

1. Gender: ☐ Male ☑ Female

2. Age: 35

3. Nationality: Chinese

4. Level of education: Ph.D

5. AI/ ChatGPT experience: ☑ Yes ☐ No

B. Intercultural Sensitivity Scale

**Directions:** The current AI-ICC questionnaire includes 15 items/ statements with five (5) options per item, as: 1 – Strongly agree; 2 – Agree, 3 – Uncertain; 2 – Disagree, and 5 – Strongly disagree.	Strongly agree	Agree	Uncertain	Disagree	Strongly disagree
1. AI and AI-powered machines can be helpful in bridging communication gaps between people from different cultures.	1	2 ✓	3	4	5
2. So far, AI has been successful in facilitating cross-cultural understanding and collaboration in mediated communication.	1 ✓	2	3	4	5
3. AI can help overcome language barriers and promote more inclusive communication among diverse groups.	1	2	3 ✓	4	5

CamScanner

No (110) E P2

4. There some cultural biases and stereotypes in AI-powered language tools and communication platforms.	1	2	3 ✓	4	5
5. AI has affected the localization and adaptation of content for different cultural audiences in digital communication.	1	2 ✓	3	4	5
6. I encountered any challenges in using AI-powered translation tools when communicating with people from different cultural backgrounds.	1	2	3 ✓	4	5
7. AI has the potential to promote cultural exchange and appreciation by enabling easier access to diverse perspectives and information.	1	2 ✓	3	4	5
8. AI-powered machines and chatbots should be programmed to respect and adapt to cultural norms and communication styles while interacting with users from different cultures.	1 ✓	2	3	4	5
9. There are some potential risks and concerns regarding AI's role in cross-cultural communication, such as perpetuating cultural stereotypes and misinterpretations.	1	2 ✓	3	4	5
10. I have participated in some cross-cultural virtual collaborations that relied on AI for communication and coordination.	1	2 ✓	3	4	5
11. I think AI can enhance intercultural learning experiences and foster empathy among individuals from different cultural backgrounds.	1	2 ✓	3	4	5
12. AI-powered virtual reality (VR) and augmented reality (AR) technologies can impact cross-cultural communication and understanding.	1	2 ✓	3	4	5
13. Somehow AI-generated content may unintentionally offend and misled individuals from specific cultural backgrounds.	1	2	3 ✓	4	5
14. Ethical considerations should be taken into account when designing AI-powered communication tools that cater to diverse cultural contexts.	1	2 ✓	3	4	5
15. Universities and educational institutions leverage AI to create more inclusive and culturally diverse online learning environments.	1	2 ✓	3	4	5

In the case of your agreement on sharing your answer/ points of views for questions below, please write down your comment/ point of view for all questions or any question that you prefer:

1. Did you experience the use of AI and AI-powered chatbots, such as ChatGPT and for what purpose?

   Yes

2. How do you perceive the role of AI and AI-powered machines in bridging communication gaps between people from different cultures?

   AI can ~~[illegible]~~ contribute to promoting cultural diversity

CamScanner

3. Have you experienced and observed any instances where AI has been successful in facilitating cross-cultural understanding and collaboration in mediated communication?

4. In what ways do you think AI can help overcome language barriers and promote more inclusive communication among diverse groups?

5. Are there any cultural biases or stereotypes that you have noticed in AI-powered language tools or communication platforms?

6. How can universities and educational institutions leverage AI to create more inclusive and culturally diverse online learning environments?

Thank you for your time and participation

CS CamScanner

Participant No (111) & P(1)

UNIVERSITI MALAYA

Depart of Media and Communication Studies

AI and Mediated Intercultural Communication Questionnaire

Dear UM Student/ Researcher,

Through this survey, we want to assess the probable effects of Artificial Intelligence (AI) on mediated communication among people from different cultures. This instrument which been developed by the help of the existing literature and ChatGPT, has 15 structured items with five (5) options per item and six (6) open-ended questions. Your participation in the survey is voluntary and the personal identifications of the participants will be treated as strictly confidential.

Thank you for your cooperation.

The researchers

A. Demographic Information

1. Gender: ☐ Male ☑ Female

2. Age: 26

3. Nationality: Malaysian

4. Level of education: Masters

5. AI/ ChatGPT experience: ☐ Yes ☑ No

B. Intercultural Sensitivity Scale

**Directions:** The current AI-ICC questionnaire includes 15 items/ statements with five (5) options per item, as: 1 – Strongly agree; 2 – Agree, 3 – Uncertain; 4 – Disagree, and 5 – Strongly disagree.	Strongly agree	Agree	Uncertain	Disagree	Strongly disagree
1. AI and AI-powered machines can be helpful in bridging communication gaps between people from different cultures.	1	(2)	3	4	5
2. So far, AI has been successful in facilitating cross-cultural understanding and collaboration in mediated communication.	1	2	(3)	4	5
3. AI can help overcome language barriers and promote more inclusive communication among diverse groups.	1	2	(3)	4	5

CamScanner

Participant No (111) & P2

4. There some cultural biases and stereotypes in AI-powered language tools and communication platforms.	1	2	(3)	4	5
5. AI has affected the localization and adaptation of content for different cultural audiences in digital communication.	1	(2)	3	4	5
6. I encountered any challenges in using AI-powered translation tools when communicating with people from different cultural backgrounds.	1	2	3	(4)	5
7. AI has the potential to promote cultural exchange and appreciation by enabling easier access to diverse perspectives and information.	1	2	(3)	4	5
8. AI-powered machines and chatbots should be programmed to respect and adapt to cultural norms and communication styles while interacting with users from different cultures.	1	(2)	3	4	5
9. There are some potential risks and concerns regarding AI's role in cross-cultural communication, such as perpetuating cultural stereotypes and misinterpretations.	1	2	(3)	4	5
10. I have participated in some cross-cultural virtual collaborations that relied on AI for communication and coordination.	1	2	3	(4)	5
11. I think AI can enhance intercultural learning experiences and foster empathy among individuals from different cultural backgrounds.	1	(2)	3	4	5
12. AI-powered virtual reality (VR) and augmented reality (AR) technologies can impact cross-cultural communication and understanding.	1	(2)	3	4	5
13. Somehow AI-generated content may unintentionally offend and misled individuals from specific cultural backgrounds.	1	2	3	(4)	5
14. Ethical considerations should be taken into account when designing AI-powered communication tools that cater to diverse cultural contexts.	(1)	2	3	4	5
15. Universities and educational institutions leverage AI to create more inclusive and culturally diverse online learning environments.	1	(2)	3	4	5

In the case of your agreement on sharing your answer/ points of views for questions below, please write down your comment/ point of view for all questions or any question that you prefer:

1. Did you experience the use of AI and AI-powered chatbots, such as ChatGPT and for what purpose?

2. How do you perceive the role of AI and AI-powered machines in bridging communication gaps between people from different cultures?

CamScanner

Participant No (111) & PC

3. Have you experienced and observed any instances where AI has been successful in facilitating cross-cultural understanding and collaboration in mediated communication?

4. In what ways do you think AI can help overcome language barriers and promote more inclusive communication among diverse groups?

5. Are there any cultural biases or stereotypes that you have noticed in AI-powered language tools or communication platforms?

6. How can universities and educational institutions leverage AI to create more inclusive and culturally diverse online learning environments?

Thank you for your time and participation

CamScanner

Participant No (112) & P(1)

UNIVERSITI MALAYA

Depart of Media and Communication Studies

AI and Mediated Intercultural Communication Questionnaire

Dear UM Student/ Researcher,

Through this survey, we want to assess the probable effects of Artificial Intelligence (AI) on mediated communication among people from different cultures. This instrument which been developed by the help of the existing literature and ChatGPT, has 15 structured items with five (5) options per item and six (6) open-ended questions. Your participation in the survey is voluntary and the personal identifications of the participants will be treated as strictly confidential.

Thank you for your cooperation.

The researchers

A. Demographic Information

1. Gender: ☐ Male ☑ Female

2. Age: 22

3. Nationality: China

4. Level of education: Master

5. AI/ ChatGPT experience: ☑ Yes ☐ No

B. Intercultural Sensitivity Scale

**Directions:** The current AI-ICC questionnaire includes 15 items/ statements with five (5) options per item, as: 1 – Strongly agree; 2 – Agree, 3 – Uncertain; 4 – Disagree, and 5 – Strongly disagree.	Strongly agree	Agree	Uncertain	Disagree	Strongly disagree
1. AI and AI-powered machines can be helpful in bridging communication gaps between people from different cultures.	1	(2)	3	4	5
2. So far, AI has been successful in facilitating cross-cultural understanding and collaboration in mediated communication.	1	2	(3)	4	5
3. AI can help overcome language barriers and promote more inclusive communication among diverse groups.	1	(2)	3	4	5

CamScanner

No (112) & PC

4. There some cultural biases and stereotypes in AI-powered language tools and communication platforms.	1	2	(3)	4	5
5. AI has affected the localization and adaptation of content for different cultural audiences in digital communication.	1	2	3	(4)	5
6. I encountered any challenges in using AI-powered translation tools when communicating with people from different cultural backgrounds.	1	2	(3)	4	5
7. AI has the potential to promote cultural exchange and appreciation by enabling easier access to diverse perspectives and information.	1	(2)	3	4	5
8. AI-powered machines and chatbots should be programmed to respect and adapt to cultural norms and communication styles while interacting with users from different cultures.	1	2	3	(4)	5
9. There are some potential risks and concerns regarding AI's role in cross-cultural communication, such as perpetuating cultural stereotypes and misinterpretations.	1	2	(3)	4	5
10. I have participated in some cross-cultural virtual collaborations that relied on AI for communication and coordination.	1	(2)	3	4	5
11. I think AI can enhance intercultural learning experiences and foster empathy among individuals from different cultural backgrounds.	1	2	3	(4)	5
12. AI-powered virtual reality (VR) and augmented reality (AR) technologies can impact cross-cultural communication and understanding.	1	2	(3)	4	5
13. Somehow AI-generated content may unintentionally offend and misled individuals from specific cultural backgrounds.	1	(2)	3	4	5
14. Ethical considerations should be taken into account when designing AI-powered communication tools that cater to diverse cultural contexts.	1	2	3	(4)	5
15. Universities and educational institutions leverage AI to create more inclusive and culturally diverse online learning environments.	1	(2)	3	4	5

In the case of your agreement on sharing your answer/ points of views for questions below, please write down your comment/ point of view for all questions or any question that you prefer:

1. Did you experience the use of AI and AI-powered chatbots, such as ChatGPT and for what purpose?

   Yes. For study

2. How do you perceive the role of AI and AI-powered machines in bridging communication gaps between people from different cultures?

CS CamScanner

No (112) & P(3)

3. Have you experienced and observed any instances where AI has been successful in facilitating cross-cultural understanding and collaboration in mediated communication?

Yes

4. In what ways do you think AI can help overcome language barriers and promote more inclusive communication among diverse groups?

5. Are there any cultural biases or stereotypes that you have noticed in AI-powered language tools or communication platforms?

No

6. How can universities and educational institutions leverage AI to create more inclusive and culturally diverse online learning environments?

Thank you for your time and participation

CamScanner

Participant No (113) E, PC

UNIVERSITI MALAYA

Depart of Media and Communication Studies

AI and Mediated Intercultural Communication Questionnaire

Dear UM Student/ Researcher,

Through this survey, we want to assess the probable effects of Artificial Intelligence (AI) on mediated communication among people from different cultures. This instrument which been developed by the help of the existing literature and ChatGPT, has 15 structured items with five (5) options per item and six (6) open-ended questions. Your participation in the survey is voluntary and the personal identifications of the participants will be treated as strictly confidential.

Thank you for your cooperation.

The researchers

A. Demographic Information

1. Gender: ☑ Male ☐ Female

2. Age: 32

3. Nationality: Malaysian

4. Level of education: Master

5. AI/ ChatGPT experience: ☑ Yes ☐ No

B. Intercultural Sensitivity Scale

**Directions:** The current AI-ICC questionnaire includes 15 items/ statements with five (5) options per item, as: 1 – Strongly agree; 2 – Agree, 3 – Uncertain; 4 – Disagree, and 5 – Strongly disagree.	Strongly agree	Agree	Uncertain	Disagree	Strongly disagree
1. AI and AI-powered machines can be helpful in bridging communication gaps between people from different cultures.	1	(2)	3	4	5
2. So far, AI has been successful in facilitating cross-cultural understanding and collaboration in mediated communication.	1	2	(3)	4	5
3. AI can help overcome language barriers and promote more inclusive communication among diverse groups.	1	2	(3)	4	5

CamScanner

No 113 E, P2

4. There some cultural biases and stereotypes in AI-powered language tools and communication platforms.	1	2	3	4	5
5. AI has affected the localization and adaptation of content for different cultural audiences in digital communication.	1	2	3	4	5
6. I encountered any challenges in using AI-powered translation tools when communicating with people from different cultural backgrounds.	1	2	3	4	5
7. AI has the potential to promote cultural exchange and appreciation by enabling easier access to diverse perspectives and information.	1	2	3	4	5
8. AI-powered machines and chatbots should be programmed to respect and adapt to cultural norms and communication styles while interacting with users from different cultures.	1	2	3	4	5
9. There are some potential risks and concerns regarding AI's role in cross-cultural communication, such as perpetuating cultural stereotypes and misinterpretations.	1	2	3	4	5
10. I have participated in some cross-cultural virtual collaborations that relied on AI for communication and coordination.	1	2	3	4	5
11. I think AI can enhance intercultural learning experiences and foster empathy among individuals from different cultural backgrounds.	1	2	3	4	5
12. AI-powered virtual reality (VR) and augmented reality (AR) technologies can impact cross-cultural communication and understanding.	1	2	3	4	5
13. Somehow AI-generated content may unintentionally offend and misled individuals from specific cultural backgrounds.	1	2	3	4	5
14. Ethical considerations should be taken into account when designing AI-powered communication tools that cater to diverse cultural contexts.	1	2	3	4	5
15. Universities and educational institutions leverage AI to create more inclusive and culturally diverse online learning environments.	1	2	3	4	5

In the case of your agreement on sharing your answer/ points of views for questions below, please write down your comment/ point of view for all questions or any question that you prefer:

1. Did you experience the use of AI and AI-powered chatbots, such as ChatGPT and for what purpose?

   Yes, when communicating with banking & insurance companies.

2. How do you perceive the role of AI and AI-powered machines in bridging communication gaps between people from different cultures?

CamScanner

No-(113) & P(3)

3. Have you experienced and observed any instances where AI has been successful in facilitating cross-cultural understanding and collaboration in mediated communication?

4. In what ways do you think AI can help overcome language barriers and promote more inclusive communication among diverse groups?

5. Are there any cultural biases or stereotypes that you have noticed in AI-powered language tools or communication platforms?

6. How can universities and educational institutions leverage AI to create more inclusive and culturally diverse online learning environments?

Thank you for your time and participation

CamScanner

Participant No: (M) & P(1)

UNIVERSITI MALAYA

Depart of Media and Communication Studies

AI and Mediated Intercultural Communication Questionnaire

Dear UM Student/ Researcher,

Through this survey, we want to assess the probable effects of Artificial Intelligence (AI) on mediated communication among people from different cultures. This instrument which been developed by the help of the existing literature and ChatGPT, has 15 structured items with five (5) options per item and six (6) open-ended questions. Your participation in the survey is voluntary and the personal identifications of the participants will be treated as strictly confidential.

Thank you for your cooperation.

The researchers

A. Demographic Information

1. Gender: ☐ Male ☑ Female

2. Age: 36

3. Nationality: Malaysian

4. Level of education: Phd

5. AI/ ChatGPT experience: ☑ Yes ☐ No

B. Intercultural Sensitivity Scale

**Directions:** The current AI-ICC questionnaire includes 15 items/ statements with five (5) options per item, as: 1 – Strongly agree; 2 – Agree, 3 – Uncertain; 4 – Disagree, and 5 – Strongly disagree.	Strongly agree	Agree	Uncertain	Disagree	Strongly disagree
1. AI and AI-powered machines can be helpful in bridging communication gaps between people from different cultures.	1	(2)	3	4	5
2. So far, AI has been successful in facilitating cross-cultural understanding and collaboration in mediated communication.	1	2	(3)	4	5
3. AI can help overcome language barriers and promote more inclusive communication among diverse groups.	1	(2)	3	4	5

CamScanner

4. There some cultural biases and stereotypes in AI-powered language tools and communication platforms.	1	2	3	4	5
5. AI has affected the localization and adaptation of content for different cultural audiences in digital communication.	1	2	3	4	5
6. I encountered any challenges in using AI-powered translation tools when communicating with people from different cultural backgrounds.	1	2	3	4	5
7. AI has the potential to promote cultural exchange and appreciation by enabling easier access to diverse perspectives and information.	1	2	3	4	5
8. AI-powered machines and chatbots should be programmed to respect and adapt to cultural norms and communication styles while interacting with users from different cultures.	1	2	3	4	5
9. There are some potential risks and concerns regarding AI's role in cross-cultural communication, such as perpetuating cultural stereotypes and misinterpretations.	1	2	3	4	5
10. I have participated in some cross-cultural virtual collaborations that relied on AI for communication and coordination.	1	2	3	4	5
11. I think AI can enhance intercultural learning experiences and foster empathy among individuals from different cultural backgrounds.	1	2	3	4	5
12. AI-powered virtual reality (VR) and augmented reality (AR) technologies can impact cross-cultural communication and understanding.	1	2	3	4	5
13. Somehow AI-generated content may unintentionally offend and misled individuals from specific cultural backgrounds.	1	2	3	4	5
14. Ethical considerations should be taken into account when designing AI-powered communication tools that cater to diverse cultural contexts.	1	2	3	4	5
15. Universities and educational institutions leverage AI to create more inclusive and culturally diverse online learning environments.	1	2	3	4	5

In the case of your agreement on sharing your answer/ points of views for questions below, please write down your comment/ point of view for all questions or any question that you prefer:

1. Did you experience the use of AI and AI-powered chatbots, such as ChatGPT and for what purpose?

2. How do you perceive the role of AI and AI-powered machines in bridging communication gaps between people from different cultures?

CS CamScanner

No : (114) & P3

3. Have you experienced and observed any instances where AI has been successful in facilitating cross-cultural understanding and collaboration in mediated communication?

4. In what ways do you think AI can help overcome language barriers and promote more inclusive communication among diverse groups?

5. Are there any cultural biases or stereotypes that you have noticed in AI-powered language tools or communication platforms?

6. How can universities and educational institutions leverage AI to create more inclusive and culturally diverse online learning environments?

Thank you for your time and participation

CamScanner

Participant No: (115) E PO

UNIVERSITI MALAYA

Depart of Media and Communication Studies

AI and Mediated Intercultural Communication Questionnaire

Dear UM Student/ Researcher,

Through this survey, we want to assess the probable effects of Artificial Intelligence (AI) on mediated communication among people from different cultures. This instrument which been developed by the help of the existing literature and ChatGPT, has 15 structured items with five (5) options per item and six (6) open-ended questions. Your participation in the survey is voluntary and the personal identifications of the participants will be treated as strictly confidential.

Thank you for your cooperation.

The researchers

A. Demographic Information

1. Gender: ☐ Male ☑ Female

2. Age: 48

3. Nationality: Malaysia

4. Level of education: PhD

5. AI/ ChatGPT experience: ☑ Yes ☐ No

B. Intercultural Sensitivity Scale

Directions: The current AI-ICC questionnaire includes 15 items/ statements with five (5) options per item, as: 1 – Strongly agree; 2 – Agree, 3 – Uncertain; 4 – Disagree, and 5 – Strongly disagree.	Strongly agree	Agree	Uncertain	Disagree	Strongly disagree
1. AI and AI-powered machines can be helpful in bridging communication gaps between people from different cultures.	1	(2)	3	4	5
2. So far, AI has been successful in facilitating cross-cultural understanding and collaboration in mediated communication.	1	2	(3)	4	5
3. AI can help overcome language barriers and promote more inclusive communication among diverse groups.	1	(2)	3	4	5

CamScanner

Participant (115), P=(2)

4. There some cultural biases and stereotypes in AI-powered language tools and communication platforms.	1	2	3	4	5
5. AI has affected the localization and adaptation of content for different cultural audiences in digital communication.	1	2	3	4	5
6. I encountered any challenges in using AI-powered translation tools when communicating with people from different cultural backgrounds.	1	2	3	4	5
7. AI has the potential to promote cultural exchange and appreciation by enabling easier access to diverse perspectives and information.	1	2	3	4	5
8. AI-powered machines and chatbots should be programmed to respect and adapt to cultural norms and communication styles while interacting with users from different cultures.	1	2	3	4	5
9. There are some potential risks and concerns regarding AI's role in cross-cultural communication, such as perpetuating cultural stereotypes and misinterpretations.	1	2	3	4	5
10. I have participated in some cross-cultural virtual collaborations that relied on AI for communication and coordination.	1	2	3	4	5
11. I think AI can enhance intercultural learning experiences and foster empathy among individuals from different cultural backgrounds.	1	2	3	4	5
12. AI-powered virtual reality (VR) and augmented reality (AR) technologies can impact cross-cultural communication and understanding.	1	2	3	4	5
13. Somehow AI-generated content may unintentionally offend and misled individuals from specific cultural backgrounds.	1	2	3	4	5
14. Ethical considerations should be taken into account when designing AI-powered communication tools that cater to diverse cultural contexts.	1	2	3	4	5
15. Universities and educational institutions leverage AI to create more inclusive and culturally diverse online learning environments.	1	2	3	4	5

In the case of your agreement on sharing your answer/ points of views for questions below, please write down your comment/ point of view for all questions or any question that you prefer:

1. Did you experience the use of AI and AI-powered chatbots, such as ChatGPT and for what purpose?

2. How do you perceive the role of AI and AI-powered machines in bridging communication gaps between people from different cultures?

CamScanner

Participant (115) & P(3)

3. Have you experienced and observed any instances where AI has been successful in facilitating cross-cultural understanding and collaboration in mediated communication?

4. In what ways do you think AI can help overcome language barriers and promote more inclusive communication among diverse groups?

5. Are there any cultural biases or stereotypes that you have noticed in AI-powered language tools or communication platforms?

6. How can universities and educational institutions leverage AI to create more inclusive and culturally diverse online learning environments?

Thank you for your time and participation

CamScanner
